# Network mapping of root–microbe interactions in *Arabidopsis thaliana*

**DOI:** 10.1038/s41522-021-00241-4

**Published:** 2021-09-07

**Authors:** Xiaoqing He, Qi Zhang, Beibei Li, Yi Jin, Libo Jiang, Rongling Wu

**Affiliations:** 1 Beijing Advanced Innovation Center for Tree Breeding by Molecular Design, Beijing Forestry University, Beijing, China; 2 Center for Computational Biology, College of Biological Sciences and Technology, Beijing Forestry University, Beijing, China; 3 Center for Statistical Genetics, Departments of Public Health Sciences and Statistics, The Pennsylvania State University, Hershey, PA USA

**Keywords:** Microbiome, Microbial ecology

## Abstract

Understanding how plants interact with their colonizing microbiota to determine plant phenotypes is a fundamental question in modern plant science. Existing approaches for genome-wide association studies (GWAS) are often focused on the association analysis between host genes and the abundance of individual microbes, failing to characterize the genetic bases of microbial interactions that are thought to be important for microbiota structure, organization, and function. Here, we implement a behavioral model to quantify various patterns of microbe-microbe interactions, i.e., mutualism, antagonism, aggression, and altruism, and map host genes that modulate microbial networks constituted by these interaction types. We reanalyze a root-microbiome data involving 179 accessions of *Arabidopsis thaliana* and find that the four networks differ structurally in the pattern of bacterial-fungal interactions and microbiome complexity. We identify several fungus and bacterial hubs that play a central role in mediating microbial community assembly surrounding *A. thaliana* root systems. We detect 1142 significant host genetic variants throughout the plant genome and then implement Bayesian networks (BN) to reconstruct epistatic networks involving all significant SNPs, of which 91 are identified as hub QTLs. Results from gene annotation analysis suggest that most of the hub QTLs detected are in proximity to candidate genes, executing a variety of biological functions in plant growth and development, resilience against pathogens, root development, and abiotic stress resistance. This study provides a new gateway to understand how genetic variation in host plants influences microbial communities and our results could help improve crops by harnessing soil microbes.

## Introduction

The microbiota has been widely thought to be an important determinant of various natural processes ranging from biogeographical cycling to human health. Many studies have characterized strong associations between the microbiota and a variety of human disorders, but research on how the microbiome impacts plant growth has not been conducted until recently^1^. Increasing evidence shows that the microbiota plays a pivotal role in promoting plants’ stress tolerance, determining plant productivity, improving the bioavailability of nutrients, and preventing invasion by bacterial pathogens^2–8^. Some bacteria can fix and preserve nitrogen in root nodules for plants^9,10^, whereas others can even modulate the timing of flowering of plants^11,12^ and may contribute to rescuing host populations at the risk of extinction^13^. Under drought stress, root microbiomes can help crop plants maintain production^4,14^.

While the microbiota affects the phenotypes of the hosts they colonize, the hosts can also shape the structure and function of the microbial communities^15–18^. It has been widely recognized that the microbiota and their hosts form complex but well-orchestrated interaction networks^19^. There is great variability among plant species or genotypes in their ability to recruit specific microbial communities^20,21^. Plant genes affect root metabolism, immune system functioning, and root exudate composition, which in turn influence the activity and structure of the root microbiome^22^. Recent studies provide a ‘cry-for-help’ hypothesis to explain that stressed plants assemble health-promoting soil microbiomes by changing their root exudation chemistry^23–25^. The overall influence of host genetic variation on the microbiome remains an open question.

Roots of healthy plants are colonized by multi-kingdom microbial consortia^26–28^. The whole microbiome structure and function are determined by the pattern and strength of how the constituent microbes interact with each other through cooperation or competition^27,29,30^. Interactions between microbiota members, particularly bacterial-fungal interactions, contribute to plant health^26,27^. Given that fungi have a strong influence on the structure of the root microbiome, characterizing both bacteria and fungi can enhance our understanding of the root microbiome^31^. Several studies have identified highly interconnected ‘hub species’ in microbial networks that act as mediators between a host and its associated microbiome^15,32^. Yet, we are still unclear in which way microbes interact with each other to shape polymicrobial communities^33^. We know little about how microbiota members contribute to the establishment, stability, and resilience of microbial communities essential for the maintenance of plant health.

Understanding the fundamental questions described above requires integrated systems approaches^34^. Recently, with the application of next-generation sequencing, the microbiome data and host genetic data measured at unprecedented resolution have been increasingly available^28,35–37^. From these data, genome-wide association studies (GWAS) have been developed to systematically characterize the genetic underpinnings of microbiota-host associations in plants^15,31,38^. However, traditional GWAS models can only detect the host QTLs responsible for the abundance of individual microbes, failing to disentangle the relationships of diverse microbial species and microbe–host interactions^31,38,39^. It is becoming increasingly clear that genetic variation in plants influences not only the relative abundance of individual microbes but also their interaction network. To overcome the complexity of internal workings within the root microbiome that contains a highly dense microbial community, we introduce behavioral ecology theory to derive simple mathematical descriptors of pairwise interactions that encode microbial networks at any dimension^33,40^. These descriptors can discern and quantify common types of ecological interactions, including mutualism, antagonism, aggression, and altruism, which occur in biological communities. The biological relevance of these descriptors has been validated by an in vitro growth assay using diverse strains of two bacterial species^40^. We further integrated these mathematical descriptors into a GWAS setting to unveil the genetic and molecular mechanisms underlying microbial interactions in the host gut that contains a dense and highly diverse microbial community^40^_._

In this article, we report the application of our ecology-based network model to root-microbiota interactions in Arabidopsis. As a model system, Arabidopsis has been extensively studied, aimed to explore the interactions between microbial communities and hosts. In a GWAS including 179 accessions of *A. thaliana*, Bergelson et al.^31^ identified associations between the abundance of individual microbes within root microbiomes and plant genotypes. By reanalyzing this dataset, we further reveal the intricate relationship between *A. thaliana* and its colonizing microorganisms. We identify hub microbes within the root microbiome, characterize how microbes interact across kingdoms, and illustrate how this process is governed by the host genes.

## Results

### Co-occurrence networks of the root microbiota

We developed a behavioral ecology model to define the strengths of mutualism, antagonism, aggression, and altruism between each pair of microbes, quantitatively described by *Z*_mu_, *Z*_an_, *Z*_ag_, and *Z*_al_, respectively (see Experimental Procedures). Validation of these descriptors through in vitro growth assays^40,41^ shows their usefulness as a proxy to measure mutualism, antagonism, aggression, and altruism strengths. We use these descriptors to reconstruct mutualism, antagonism, aggression, and altruism networks for the root microbiota of the *A. thaliana*. To reduce the complexity of the networks, we chose the most abundant 100 OTUs in bacteria and fungi, respectively, for the reconstruction of four types of bacterial-fungal networks (OTU1-100 are listed as bacteria and OTU100-200 as fungi). We calculated node-level topological properties (i.e., degree, betweenness, closeness and eigencentrality) using the “igraph” R package. Bacterial and fungal co-occurrence network characteristics are listed in Supplementary Table 1. We are aware of that rare microbes may have an over-proportional role in regulating the functioning of host-associated environments and including rare microbes in future investigations will improve our understanding of microbial community function^42^.

**Fig. 1:**
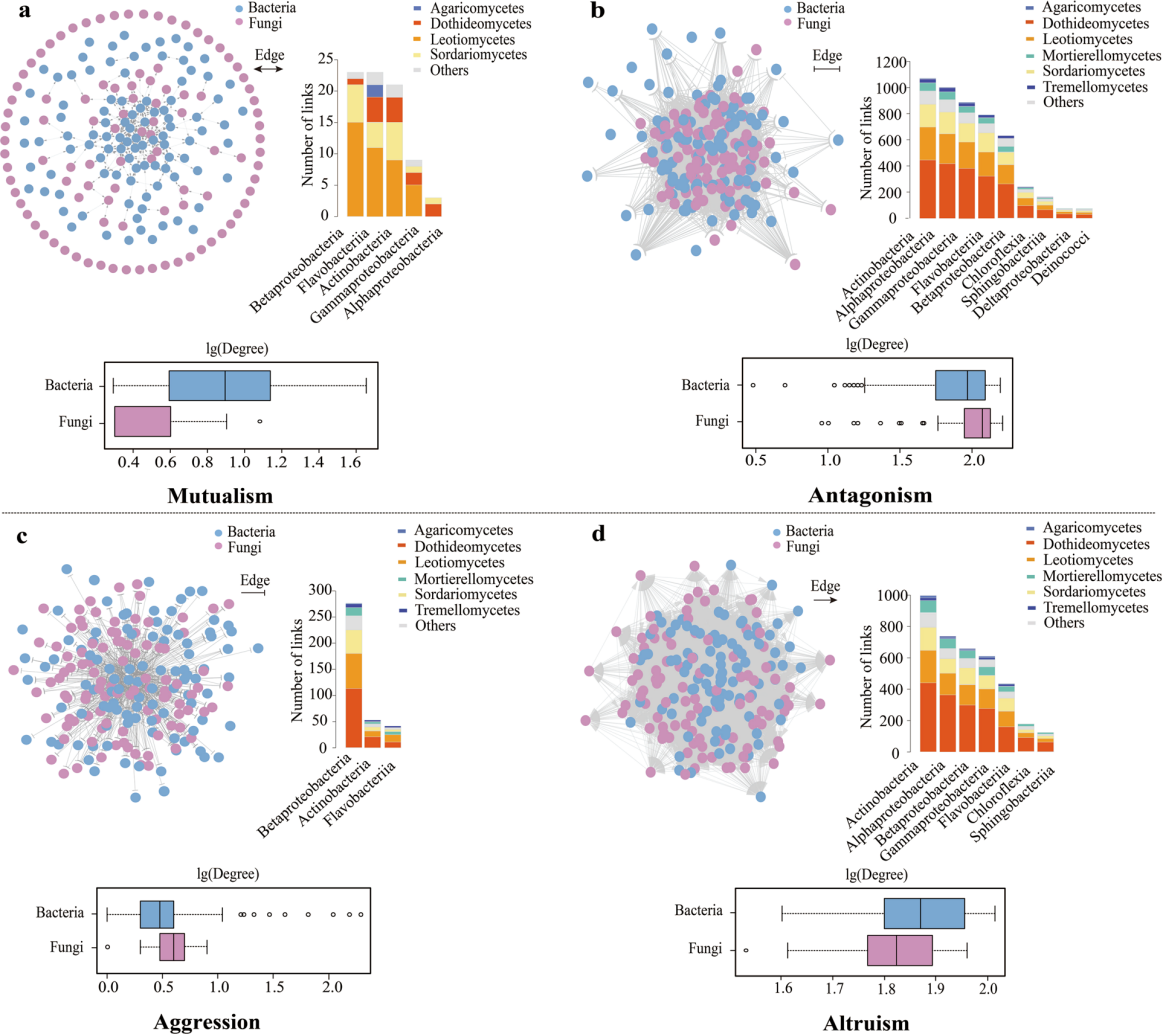
Microbial descriptor-based network at the OTU level of the root microbiome in *Arabidopsis thaliana*. **a** Z_mu_-based mutualism network. **b** Z_an_-based antagonism network. **c** Z_ag_-based aggression network. **d** Z_al_-based altruism network. In each network, bacteria and fungi are distinguished by different colors. The network analysis was performed in the “igraph” R package and visualized in Cytoscape v3.7.1. The number of links between root inter-kingdom microbes was given at the right. Bacterial and fungal OTUs were grouped at the class level and sorted according to the number of edges between bacteria and fungi within each network. In boxplots, the fungal and bacterial degrees were calculated to determine the relative importance of bacteria and fungi in each network.

Interkingdom functional diversity among fungi and bacteria is important for maintaining ecosystem functioning^28^ and microbial interkingdom interactions in roots can promote Arabidopsis survival^27^. We calculated degree-centrality parameters to determine the relative importance of bacteria and fungi in each network. It indicates that bacteria are more central to the structure of the mutualism and altruism networks than fungi (Fig. 1a, d), as bacteria tend to have a higher number (i.e., degree) of network connections than fungi (Wilcoxon test, *P* = 0.00038 and *P* = 0.0001029 for the mutualism and altruism networks, respectively) (Supplementary Table 2). In contrast, fungi in the antagonism and aggression networks appear to have a higher number of network connections than bacteria (Fig. 1b, c; *P* = 0.0002128 and *P* = 0.01961 for the antagonism and aggression networks, respectively). We also calculated and compared interkingdom microbial OTU relationships (the number of links; edges information) among bacterial and fungal taxonomic groups in four interaction networks (Fig. 1; Supplementary Table 3). Bacterial OTUs belonging to classes Betaproteobacteria, Flavobacteriia, Actinobacteria, Gammaproteobacteria, and Alphaproteobacteria displayed a strong mutualistic relationship with fungal OTUs belonging to classes Leotiomycetes, Dothideomycetes, Sordariomycetes, Agaricomycetes, and others, respectively (Fig. 1a). Bacterial classes such as Actinobacterial, Alphaproteobacteria, Gammaproteobacteria all displayed antagnonistic relationships with fungal classes Leotiomycetes, Dothidemycetes, and Sodariomycetes (Fig. 1b). In the aggression network, there were three bacterial classes (Betaproteobacteria, Actinobacteria, and Flavobacteriia) which were aggressive to fungal classes (Leotiomycetes, Sordariomycetes, Dothideomycetes, etc.) (Fig. 1c). Bacterial classes including Actinobacteria, Alphaproteobacteria, Betaproteobacteria, and Gammaproteobacteria were altruistic to fungal classes, Dothideomycetes, Leotiomycetes, Sordariomycetes, Mortierellomycetes, etc. (Fig. 1d).

### Hubs of the co-occurrence network identification

**Fig. 2:**
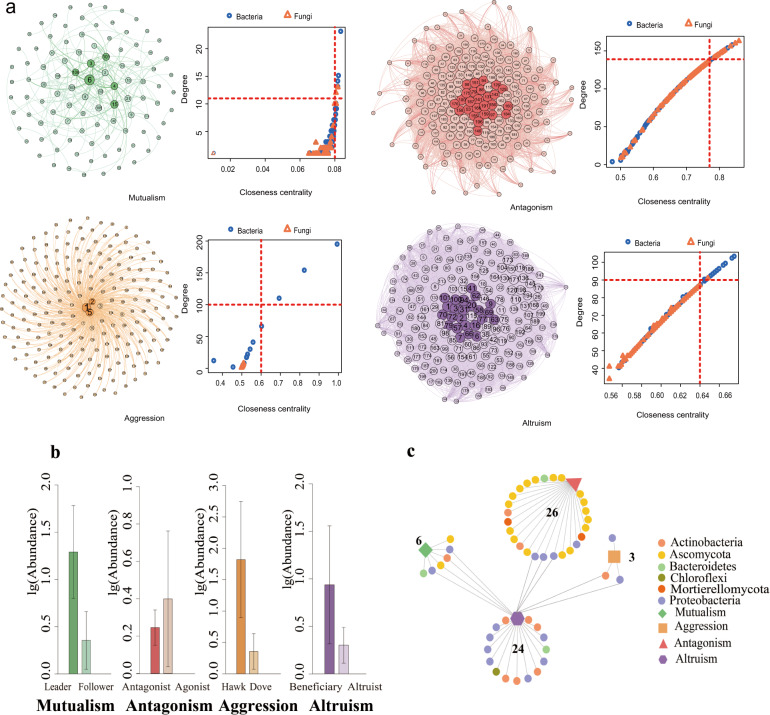
Hubs of the co-occurrence network. **a** The identity of each OTU is labeled by a number, one to 100 for bacteria and 101 to 200 for fungi. In each network, hub microbes are highlighted in border colors. The distribution of ‘Hub microbes’ in four different microbial networks was based on degree and closeness centrality values. These two values of each OTU within each network were given at the right. The red dotted line represents the screening cutoffs of ‘Hub microbes’ corresponding to each network. **b** The abundance of hub microbes within each network. **c** The shared hub microbes within each microbial network. This ‘shared network’ was represented in Cytoscape. Circle shapes represented hub microbes from each microbial network and irregular shapes represented different microbial network types. The edges were connected to hub microbes and microbial network. The distribution of ‘Hub microbes’ in four different microbial networks was based on degree and closeness centrality values. The red dotted line represents the screening cutoffs of ‘Hub microbes’ corresponding to each network. Visualization was done with Gephi for four microbial networks.

Hub microbes are important in shaping microbial communities due to their critical roles in maintaining network function^15^. The four networks differed structurally in the pattern of social links and the number of hub microbes. Fungal and bacterial OTUs that display the highest degree and the highest closeness centrality scores may serve as hub taxa to drive fungal-bacterial interaction equilibrium in *A. thaliana* roots^43^ (Fig. 2a; Supplementary Table 1).

We identified six hub microbes (leaders), four bacteria, and two fungi (nodes with degree >11 and closeness centrality values >0.08 in the network; *P* < 0.01), which dominate the mutualism network. The four hub bacteria are classes Betaproteobacteria (two OTUs), Sphingobacteriia, and Actinobacteria, and the two fungal hubs are phylum Ascomycota (2 OTUs). In the antagonism network, 26 hub microbes (‘antagonists’) that are more combative were found to act as ‘public enemies’, which were antagonistic to many more microbes than other microbes (degree >139 and closeness centrality values >0.78). The ‘agonists’ that are less combative were observed to be more abundant than the ‘antagonists’ (*P* < 0.01; Fig. 2b). In the aggression network, three OTUs belonging to the bacterial class Betaproteobacteria (two OTUs) and Actinobacteria might represent the hub taxa (degree >100 and closeness centrality values >0.60; *P* < 0.5). The ‘hawks’ which are considered to aggressively repress others are abundant than the ‘doves’ (those inhibited by others). The altruism network includes some ‘altruists’ (24 hub microbes; Fig. 2a; Supplementary Table 1) that sacrificed their own growth by providing resources to beneficiaries (degree >90 and closeness centrality values >0.64). The hub microbes (beneficiaries) are more abundant than the altruists (Fig. 2b; *P* < 0.01).

A total of 59 OTUs were identified as hub species, which were mainly from bacterial phyla proteobacteria (21 OTUs), Actinobacteria (12 OTUs), Chloroflexi (1 OTU), Bacteroidetes (3 OTUs) and fugal phyla Ascomycota (20 OTUs), Mortierellomycota (2 OTUs) (Supplementary Table 1). The altruism network shares 4, 3, and 2 phyla with the mutualism, antagonism, and aggression networks, respectively (Fig. 2c).

### Mapping root-microbe interactions

**Fig. 3:**
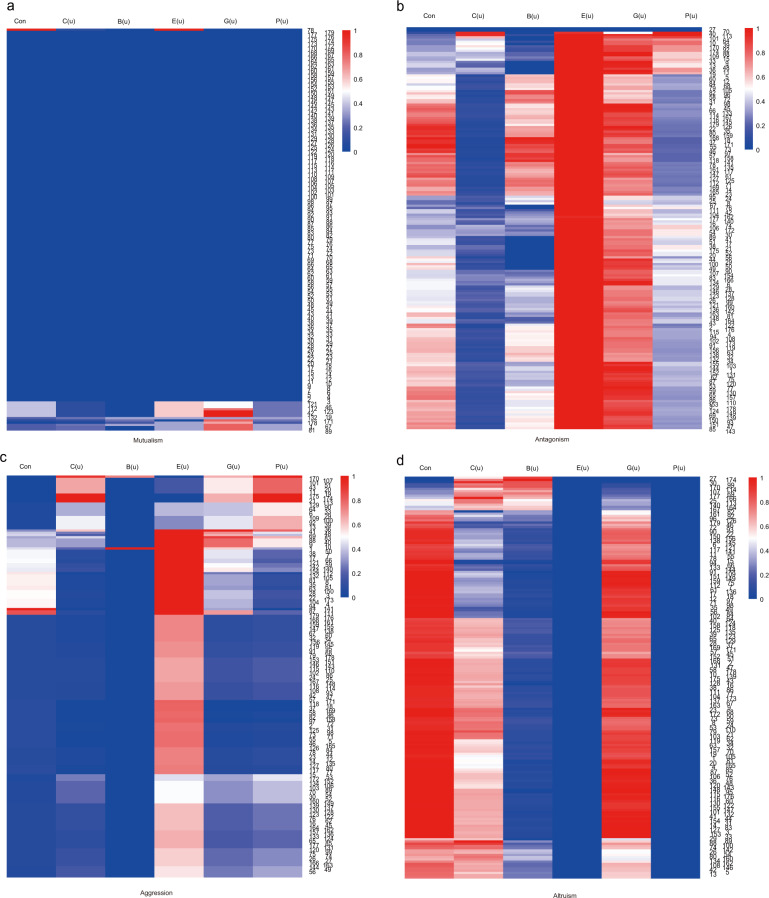
Heatmap of six emergent property indices. **a** Mutualism. **b** Antagonism. **c** Aggression. **d** Altruism.

We calculated six centrality indices namely connectivity (Con), closeness (C(u)), betweenness (B(u)), eccentricity (E(u)), eigencentrality (G(u)), and PageRank (P(u)) (Fig. 3) for each network using the formulas given in Jiang et al.^40^. In the same network type, these indices exhibit differences among hosts and, also, the same index varies among network types. All indices depend on network type which provides a basis for mapping microbial network QTLs. In our previous study, we developed a statistical procedure to test and estimate how individual SNPs are associated with network properties^40^. By treating each network index as a phenotype, we performed association mapping for the interaction networks (Supplementary Fig. 1). The chosen significant threshold is −log_10_ (*P*) ≥ 5. The population structure analysis was performed by Admixture software for 179 *A.thaliana* accessions. The results indicated that the 179 accessions were divided into six subgroups. We also considered population structure in Q GWAS and QK GWAS. The QQ plots results showed that the population structure and genetic relatedness among accessions have subtle impact on the results of association analysis (Supplementary Figs. 2–7). Our model identified 1142 significant host genetic variants throughout the plant genome, which are responsible for centrality indices of each network, including 225 acting through mutualism, 845 through antagonism, 49 through aggression, and 23 through altruism (19.70% for mutualism, 73.99% for antagonism, 4.29% for aggression, and 2.01% for altruism) (Supplementary Table 4). It appears that more variants control mutualism and antagonism than aggression and altruism. We also calculated heritability estimates for each network property as described in Li et al’s research^44^. SNP based heritability varied from 0 to 35.95% (Supplementary Table 5). We found that the total SNP-h^2^ of mutualism (C(u)) (96%), antagonism (E(u)) (83%), mutualism (P(u)) (82%) and aggression (Con) (72%) was higher than other topological features of the networks.

### QTL networks

**Fig. 4:**
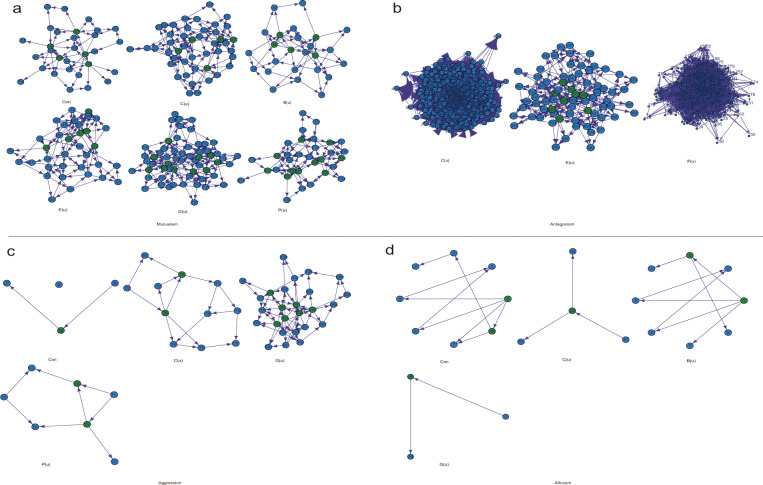
Bayesian QTL networks of significant SNPs mediating interaction networks of microbial network. **a** mutualism. **b** antagonism. **c** aggression. **d** altruism. Hub QTLs within each genetic network are highlighted in green circles. The emergent properties of each microbial network are described by connectivity (Con), closeness (C(u)), betweenness (B(u)), eccentricity (E(u)), eigencentrality (G(u)), and PageRank (P(u)).

We implemented Jiang et al.’s^41^ procedure to reconstruct Bayesian QTL networks among the significant SNPs detected to affect each type of microbial network and identified 91 hub QTLs (Fig. 4; Table 1). Through QTL network analysis, we can better characterize how a QTL mediates microbial cooperation or competition through its epistatic interactions with other QTLs. In the QTL network for the microbial mutualism network, we identify a hub QTL that affects connectivity QTL, annotated to gene *TMK3* (*AT2G01820*) that orchestrates plant growth by regulation of both cell expansion and cell proliferation and as a component of auxin signaling^45^. A hub QTL for the betweenness of microbial mutualism network is located in gene *IBR1(AT4G05530)*, encoding indole-3-butyric acid response 1(IBR1). IBR1 are involved in root hair elongation^46^. *AOC4*(*AT1G13280*, an eigencentrality hub QTL) encodes allene oxide cyclase. One of four genes in Arabidopsis that encode this enzyme, which catalyzes an essential step in jasmonic acid biosynthesisis, a hormone whose role in defense responses is well established^47^. A hub QTL for the PageRank of the mutualism network represents gene *NTRB (AT4G35460)* encoding NADPH-dependent thioredoxin reductase. Thioredoxin is a key regulator of intracellular redox status that determine plant development in response to biotic and abiotic stress. Thioredoxin reductase (ntra ntrb) mutant alters both auxin transport and metabolism, causing a loss of apical dominance and reduced secondary root production, etc., largely regulated by auxin^48^.

**Table 1:** Numbers of mutualism, antagonism, aggression, and altruism QTLs that affect the emergent properties of ecological networks. The names of genes to which QTLs are annotated are given below.

	Emergent property					
	Con	C(u)	B(u)	E(u)	G(u)	P(u)
Mutualism QTLs	5	5	5	6	11	11
	—	*AT2G20635*	–	*AT5G35602*	–	*AT2G20635*
	*AT5G35602*	*AT4G05594*	*SRF1*	*TMK3*	*AT1G04867*	*AT1G04867*
	*TMK3*	–	*ARPC3*	*RAD23A*	*AT4G05594*	–
	—	*AT1G11700*	*AT4G10201*	*CYP721A1*	*IBR1*	*AT4G05594*
	*EMB2296*	*TNPO3*	*AT3G22421*	*WSS1/WSS1B*	*VIIIA*	–
Gene				–	*AT4G36190*	*AT4G03824*
					*AOC4*	*IBR1*
					*AT5G46850*	*AT4G36190*
					*AT2G38260*	*NTRB*
					–	*AT4G35410*
					*NTRB*	*AT1G50330*
Antagonism QTLs	0	25	1	9	1	6
		–	*Hrd1A*	*AT1G05380*	*SS5*	*AT5G11430*
		*AT5G35555*		–		*AT1G72230*
		*UBP22*		*JAZ6*		*CSTF77*
		–		*AT3G01850*		–
		*AT3G12350*		*IGPD*		–
		*DELTA-VPE*		*AT4G15640*		–
		*DEL2*		*AT4G16050*		
		*MAIN*		*AT5G19930*		
		*CAP1*		*SBT5.4*		
		*DEL2*				
Gene		–				
		*AT4G31360*				
		*AT4G25610*				
		*NAC058*				
		*AT5G20660*				
		–				
		*AT2G34185*				
		*AT5G19480*				
		*AT4G00320*				
		*UPL4*				
		*AT3G13662*				
		–				
		*PEPR2*				
		*AT5G11750*				
		–				
Aggression QTLs	1	2	0	1	7	2
	*GA2OX5*	*AT1G12760*		*AT5G19097*	*AT5G35525*	–
		*AT3G30405*			*AT5G35495*	*PDE320*
					*AT3G29175*	
					*UPL4*	
Gene					–	
					*AT1G14800*	
					*AT4G08280*	
Altruism QTLs	2	1	2	0	1	0
	*EGRET*	*RGLG5*	*AT5G60470*		–	
Gene						
	–		*PUM11*			

In the QTL network for the microbial antagonism network, a closeness hub QTL acts like gene *UBP22(AT5G10790)*. *UBP22* encodes a ubiquitin-specific protease, which plays role in regulating plant development and stress responses^49^. A hub QTL for the betweenness of the microbial antagonism network is located in gene *Hrd1A* which may be an important regulator of heat stress response in Arabidopsis^50^. A hub QTL for the closeness of the microbial antagonism network acts like gene *PEPR2(AT1G17750)* encoding PEP1 receptor 2, which is transcriptionally induced by wounding and pathogen-associated molecular patterns and contributes to defense responses in Arabidopsis^51^. *CHL1(AT5G40090)* is the hub QTL for the eigencentrality of the antagonism network, which encodes disease resistance protein (TIR-NBS class). TIR-NBS protein is involved in disease resistance in Arabidopsis^52^.

There are 12 pleiotropic genes including *UPL4(AT5G02880)*, which are detected to influence multiple types of microbial networks or properties (Table 1). *UPL4* encodes a ubiquitin-protein ligase, function additively in the regulation of plant growth and development, and positively modulate immune hormone salicylic acid (SA)-mediated basal and induced resistance responses^53^.

## Discussion

Plant rhizosphere is considered as the second genome of plants. Nowadays, research on rhizosphere interactions has become one of the hottest topics in modern biology and agriculture. Potentially beneficial bacteria and fungi may serve as a valuable foundation for bio-fertilizer development in agriculture and forestry. Knowledge about how plants communicate and crosstalk with their entire microbiota will be crucial for the choice of microbes that benefit sustainable plant growth^54,55^. However, our understanding of the intrinsic principles underlying the assembly of the root microbial community is still limited^56^. In this article, we demonstrate the potential of a new computational model to reveal these principles behind.

Currently, network analysis has emerged as an extremely promising approach for modeling complex biological systems and can potentially provide deep and unique perspectives on microbial interactions and ecological assembly rules beyond those of simple richness and composition^17^. Properties of co-occurrence networks can reveal the intrinsic mechanisms of microbial interactions in response to environmental disturbance^35,57^. The connection and strength of the network even are crucial for the resistance to the pathogens^17^. In this study, we calculated four descriptors between each pair of genera and reconstructed four corresponding 200-node networks accordingly. Each described root microbiome interactions according to a different ecological interaction metric and help us to explore co-occurrence patterns of bacterial and fungal taxa. The four networks differ structurally in the pattern of bacterial-fungal interactions and microbiome complexity and the number of hub microbes. These differences provide a basis for the following microbial network mapping. In our previous study, we quantified the internal workings of microbial community within the gut by mathematical descriptors of pairwise interactions and provided a critical starting point to investigate these higher order interactions more deeply^40^.

Different members of root microbiota affect plant health through a complex network of microbial interactions. It is important to understand the mechanistic details of how ecological interactions are generated and how they are at play within the root microbiota. In our study, the phylogenetic signal measurement of network property parameters is calculated by Pagel’s lambda (Supplementary Table 6). In mutualism, the signals in both bacterial and fungal groups are relatively strong while in the other three networks most phylogenetic signals are close to 0. As can be seen, the values of lambda vary in different network types and network property parameters. Take aggression as an example, bacteria show a much higher signal value than fungi in Con (connectivity) but less than fungi in C(u) (closeness). We also found that bacteria have a higher degree in the mutualism and altruism networks and are more central to the structure of networks. Fungi have more connections in the antagonism and aggression networks, however, based on the directions of edges, bacteria still are more antagonistic and aggressive to fungi (Supplementary Table 3). Understanding the interaction among different species within a community is one of the central goals in ecology^58^. Bacterial communities aid in maintaining the microbial balance and protect host plants against the detrimental effects of filamentous eukaryotic microbes^27^. In Bergelson et al.’s research, strong and significant cross-kingdom correlations for the top taxa were observed which implied that bacteria and fungi interacted in the root microbiome and variation within the root microbiome was influenced by members of both kingdoms communities^31^. A previous study showed that microbes tend to be positively related within kingdoms but negatively related between kingdoms^15^. Besides bacteria and fungi, rhizosphere bacteriophages and protists also play roles in plant health^59,60^, which should be included in further research.

The identification of network hubs and their importance in microbial community structure has crucial implications for studying microbe–microbe interactions and can facilitate the design of strategies for future targeted biocontrol^15^. Hub microorganisms have a regulatory influence on the network of microbial interactions, which can exert strong effects on microbiome assembly and serve as mediators between the plant and microbiome^15^. According to centrality measurements, such as degree, closeness centrality, and betweenness centrality, hub microorganisms are tightly connected within a co-occurrence network^15^. We identified hub microbes in four types of networks, mutualism, antagonism, aggression, and altruism. The most dominant taxa as hub microbes belong to bacterial phyla Proteobacteria (21 OTUs), Actinobacteria (12 OTUs), and fugal phyla Ascomycota (20 OTUs). This is consistent with the finding that Proteobacteria, Actinobacteria, and Ascomycota are the most abundant phyla in plants and soil^43,61^. Actinobacteria is one of the bacteria whose dysbiosis in abundance in tomato rhizosphere causes the incidence of bacterial wilt disease^6^. Some key taxa with the highest degree and betweenness centrality for the root microbiome identified in Bergelson et al.’s research^31^ such as *Massilia*, *Actinobacteria*, and *Actinoplanes*, are also considered as hub microbes in our study (Supplementary Table 1).

Plant phenotypes are inextricably shaped by their interactions with microbes^34^. In a well-designed GWAS study, Bergelson, et al.^31^ found a few significant QTLs that are associated with root microbial species richness and community structure, which are involved in plant immunity, cell-wall integrity, root, and root-hair development. In this study, we used a newly developed network mapping model^40^ to reanalyze Bergelson et al.’s^31^ data, characterizing previously undetected QTLs that mediate microbial interactions. We found that most of the QTLs detected by the new model can be annotated to candidate genes with known biological functions including plant growth and development, resilience against pathogens, root development, and improved resistance against abiotic stress conditions (Table 1; Supplementary Table 7). We also investigated candidate genes within ~10 kb windows on each side of associated SNPs by software PLINK. The genes of R^2^ > 0.8 were retained. Supplementary Table 8 lists the 135 genes within a 10 kb window around associated SNPs including genes such as *PEPR2, UBP22, UPL4* which were also identified as hub genes linked to the six network property parameters.

Understanding how microbes improve plant stress resistance will enhance our understanding of how plants survive in stress conditions. In the near future, it will be crucial to unravel the complex network of genetic, microbial, and metabolic interactions, including the signaling events mediating microbe–host interactions. Scientists have also linked the phyllosphere microbiome to plant Health^62^ and found host genes could affect bacterial communities in the phyllosphere^17^. Building synthetic microbiomes in plants has been proved to be useful for future research on plant–microbe interactions^30,63–66^. Studies have shown the potential of microbiome adjustment tailored to bring benefits for plant growth and resistance to biotic and abiotic challenges^67^. Bioorganic fertilizers promote indigenous soil plant-beneficial consortium to enhance plant disease suppression^68^. The design of more efficient biofertilizers to update soil function has important implications for the manipulation of crop microbiomes for sustainable agriculture. Our work provides a comprehensive exploration of microbial interkingdom interactions, hub microbes, and plant genes for the structure of the root microbiome. The results obtained could help design synthetic microbiomes beneficial for plant growth.

## Methods

### Root microbiome experiment

Bergelson et al.^31,38^ conducted a genome-wide association study (GWAS) for the root microbiome in *Arabidopsis thaliana*. The study included 179 accessions of *A. thaliana*, each measured for the bacterial and fungal abundance of the root microbiota using a 16S/ITS rRNA gene sequencing technique and genotyped for Arabidopsis SNPs by a high-throughput sequencing technology (Supplementary Table 9).

### Microbial interactions analysis

We chose the 100 most abundant bacterial OTUs and the 100 most abundant fungal OTUs (Supplementary Table 10) to reconstruct microbial interaction networks using a microbial behavioral network model^40^. This model is based on mathematical descriptors of four types of microbe-microbe interactions, mutualism, antagonism, aggression, and altruism, expressed as

1 $$\begin{array}{l}Zmu = \frac{{XuXv}}{{Xu\, - \,Xv}} \ldots \ldots \ldots \ldots \ldots \ldots \ldots \ldots \ldots \ldots \ldots \ldots .{{{\mathrm{Mutualism}}}}\\ Zan = \frac{1}{{\left( {XuXv} \right)\left( {Xu\, - \,Xv} \right)}} \ldots \ldots \ldots \ldots \ldots \ldots \ldots \ldots...... .{{{\mathrm{Antagonism}}}}\\ Zag = \frac{{Xu}}{{Xv}} \ldots \ldots \ldots \ldots \ldots \ldots \ldots \ldots \ldots \ldots \ldots \ldots \ldots \ldots {{{\mathrm{Aggression}}}}\\ Zal = 1 - \frac{{Xv}}{{Xu}} \ldots \ldots \ldots \ldots \ldots \ldots \ldots \ldots \ldots \ldots \ldots \ldots\ldots{{{\mathrm{Altruism}}}}\end{array}$$

where *x*_u_ and *x*_v_ (*x*_u_ > *x*_v_, *u* ≠ *v*, *u*, *v* = 1,…, m) are the abundance of two microbes u and v, and m is the number of microbes. Based on the above equations, we use the corrected microbial abundance (log10-transformed) to quantify four interactional relationships of two microbes *u* and *v*. The descriptor, Z_*mu*_, can be used to quantify a cooperative relationship (mutualism) between two microbes. The descriptor, Z_*an*_, can be used to quantify a competitive relationship (antagonism) between two microbes. The descriptor, Z_*ag*_, can represent the utilization extent (aggression) of a more abundant microbe to a less abundant microbe. The descriptor, Z_*al*_, can represent the sacrifice extent (altruism) of a more abundant microbe to a less abundant microbe. Microbial interaction relationships were quantified as interaction matrices. Each matrix was normalized to control the range of interaction relationship values within [0,1]. Next, we performed threshold filtering to obtain microbial interaction sparse matrix. The threshold is 0.95 in mutualism, antagonism, and aggression networks and that in altruism network is 0.99. In each network, the value of the interaction relationship above the threshold was retained. Then we obtained a sparse network of each interaction type.

### Microbial networks analysis

By microbial interactions analysis, we can reveal internal workings within the root microbial community. The interaction networks can be visualized using Gephi (https://gephi.org/). We constructed the corresponding network for mutualism, antagonism, aggression, and altruism, respectively. Emergent properties of each network can be calculated in the “igraph” R package^69^. We calculated six network indices to describe the features of various networks, including connectivity (Con), closeness (C(u)), betweenness(B(u)), eccentricity (E(u)), eigencentrality (G(u)) and Pagerank (P(u)). The specific calculation method was described by Jiang et al.^40^. The heat maps of each network index were generated by package *pheatmap* in R (https://CRAN.R-project.org/package=pheatmap). Meanwhile, microbial networks can be used to statistically identify hub taxa. We calculated the degree of each node for every network using the “igraph” R package^69^. It is generally believed that hub microbes with a high degree and closeness centrality value play crucial roles in microbial networks^15,43^. These hub microbes (called leaders, antagonists, hawks, and beneficiaries) in mutualism, antagonism, aggression, and altruism networks, respectively, are compared with other microbes (expressed as followers, agonists, doves, and altruists) from each network type^40^.

### Mapping microbial network properties

To study how host genes influence root microbiomes, we consider six network property parameters as phenotypic traits that are associated with host SNPs (Single Nucleotide Polymorphisms). We chose those SNPs with MAF > 5%) for association analysis. A regression model of log-transformed phenotypes at a SNP is expressed as

2 $$y_i = \mu + \mathop {\sum }\limits_{j = 1}^2 x_i\beta + e_i$$

where y_i_ represents the phenotype of the ith host, μ is the mean of the phenotypes over all hosts, x_i_ is the genotype indicator of the ith host which is 0 for high-frequency allele and 1 for low-frequency allele, β is the geneic effect of the SNP and e_i_ is a random error value. Then, we used lm function in R for association analysis from which to get the *P*-value of each SNP. Package *qqman* (https://CRAN.R-project.org/package=qqman) was used to draw the Manhattan plot. By statistical testing, we can find significant SNPs that are associated with each network property.

### QTL networks

Many existing approaches attempt to reveal the genetic architecture of complex traits by identifying key individual genes underlying the traits. However, epistatic interactions among different genes have been increasingly recognized to play an important role in genetic control. Several approaches have been developed to map epistatic interactions based on gene pairwise analysis, failing to systematically chart a network of epistasis involving all genes. More recently, Jiang et al.^41^ proposed an analytical procedure of reconstructing epistatic networks from mapping data. This procedure was used to infer QTL networks of the significant SNPs that mediate the emergent properties of microbial networks. At each SNP, we calculated the mean value of each genotype for a network parameter and assigned this value to each *Arabidopsis* accession, transforming the GWAS data structure from its SNP-phenotype illustration to SNP-based genotype representation. We implemented Bayesian networks (BN) to reconstruct genetic networks involving all significant SNPs for each network parameter. The BN-based QTL networks are directed acyclic graphs, encoded by casual SNP-SNP interactions. We identified hub QTLs that play a crucial role in the genetic architecture of plant microbiomes assembly.

### Reporting summary

Further information on research design is available in the Nature Research Reporting Summary linked to this article.

## Supplementary information

Reporting Summary

Supplementary Information

## Data Availability

The data used can be downloaded at 10.1038/s41598-018-37208-z.

## Code availability

The computer code can be freely downloaded at https://github.com/lenahe2006/npj-Biofilms-and-Microbiomes for any purpose of research. Also, all questions in data and computation can be addressed to the corresponding author.
