# Supplementary material for: Network mapping of root–microbe interactions in Arabidopsis thaliana
Source: NPJ Biofilms Microbiomes. 2021 Sep 7;7:72. doi: 10.1038/s41522-021-00241-4 (PMC8423736; doi:10.1038/s41522-021-00241-4)
Supplement: Supplementary file 2 — Supplementary Information [file 41522_2021_241_MOESM2_ESM.pdf]

Supplemental Material for:

Network mapping of root-microbe interactions in Arabidopsis thaliana

Xiaoqing He, Qi Zhang, Beibei Li, Yi Jin, Libo Jiang, and Rongling Wu

Table of Contents:

**Supplementary Table 1**	**Page 2**
**Supplementary Table 2**	**Page 11**
**Supplementary Table 3**	**Page 11**
**Supplementary Table 4**	**Page 16**
**Supplementary Table 5**	**Page 100**
**Supplementary Table 6**	**Page 105**
**Supplementary Table 7**	**Page 107**
**Supplementary Table 8**	**Page 118**
**Supplementary Table 9**	**Page 130**
**Supplementary Table 10**	**Page 134**
**Supplementary Figure 1**	**Page 218**
**Supplementary Figure 2**	**Page 219**
**Supplementary Figure 3**	**Page 220**
**Supplementary Figure 4**	**Page 221**
**Supplementary Figure 5**	**Page 222**
**Supplementary Figure 6**	**Page 223**
**Supplementary Figure 7**	**Page 224**

Supplementary Table 1-1 I

		Centralization of degree	Centralization of closeness	Centralizaton of betweenness	Eccentricity
Bacteria	OTU6	23	0.082984074	79.68095238	5
Bacteria	OTU15	15	0.081683168	64.3452381	5
Bacteria	OTU3	14	0.081280788	17.08333333	5
Bacteria	OTU4	13	0.081280788	53.16666667	5
Fungi	OTU101	13	0.081683168	59.03333333	6
Fungi	OTU106	12	0.080618893	56.48809524	6
Bacteria	OTU2	10	0.080161943	20.88333333	5
Bacteria	OTU21	10	0.07983871	24.33333333	5
Fungi	OTU103	10	0.080750408	81.86666667	6
Fungi	OTU105	10	0.07983871	26.5	6
Bacteria	OTU8	9	0.080553295	74.35238095	5
Bacteria	OTU9	9	0.07983871	42.54285714	6
Bacteria	OTU10	9	0.080553295	47.07619048	5
Bacteria	OTU13	8	0.080097087	14.59285714	5
Bacteria	OTU5	7	0.079010375	16.36666667	5
Bacteria	OTU7	7	0.079326923	24.36666667	6
Bacteria	OTU17	7	0.079903148	17.56904762	5
Bacteria	OTU1	6	0.079518072	0	5
Bacteria	OTU16	6	0.079136691	40.83333333	5
Bacteria	OTU19	6	0.078137332	14.33333333	6
Bacteria	OTU30	6	0.079454254	20.48571429	6
Bacteria	OTU35	6	0.079263411	1.333333333	5
Bacteria	OTU47	6	0.079326923	7.295238095	5
Fungi	OTU102	6	0.079073482	0	5
Fungi	OTU108	6	0.0792	0	5
Bacteria	OTU11	5	0.077708006	11.91666667	6
Bacteria	OTU12	5	0.078633836	28.03333333	6
Bacteria	OTU18	5	0.079010375	31	5
Bacteria	OTU20	5	0.077464789	18	6
Bacteria	OTU33	5	0.079136691	0	6
Bacteria	OTU34	5	0.07850912	16.0952381	6
Bacteria	OTU14	4	0.077283372	6	6
Bacteria	OTU24	4	0.078199052	26	6
Bacteria	OTU25	4	0.076684741	0	6
Bacteria	OTU28	4	0.078137332	6.676190476	5
Bacteria	OTU37	4	0.078137332	13	6
Bacteria	OTU65	4	0.078821656	0	6
Fungi	OTU113	4	0.078137332	0	5
Bacteria	OTU27	3	0.07443609	5.333333333	6
Bacteria	OTU43	3	0.078137332	3.833333333	6
Bacteria	OTU45	3	0.076803724	0	6
Bacteria	OTU53	3	0.076388889	0	7

Bacteria	OTU56	3	0.075399848	12	6
Fungi	OTU111	3	0.077464789	0	6
Fungi	OTU140	3	0.069085834	2	7
Bacteria	OTU22	2	0.075056861	1.5	6
Bacteria	OTU26	2	0.075342466	10	6
Bacteria	OTU29	2	0.076329992	3	6
Bacteria	OTU36	2	0.074660633	7	7
Bacteria	OTU40	2	0.073935773	3	6
Bacteria	OTU46	2	0.076684741	0	6
Bacteria	OTU49	2	0.077952756	0	6
Bacteria	OTU52	2	0.073224852	1	7
Bacteria	OTU54	2	0.076036866	1.083333333	6
Bacteria	OTU84	2	0.073224852	9	7
Fungi	OTU104	2	0.071895425	1	7
Fungi	OTU114	2	0.077830189	0	6
Fungi	OTU116	2	0.075227964	0	6
Fungi	OTU119	2	0.076566125	0	6
Fungi	OTU128	2	0.077647059	0	6
Fungi	OTU130	2	0.074380165	1	6
Fungi	OTU138	2	0.074943225	0	7
Fungi	OTU164	2	0.073496659	0	7
Fungi	OTU167	2	0.075572519	0	7
Fungi	OTU169	2	0.073387695	0	6
Bacteria	OTU23	1	0.068989547	0	8
Bacteria	OTU32	1	0.072474378	0	7
Bacteria	OTU39	1	0.072687225	0	7
Bacteria	OTU44	1	0.073062731	0	7
Bacteria	OTU48	1	0.01010101	0	1
Bacteria	OTU50	1	0.01010101	0	1
Bacteria	OTU51	1	0.01010101	0	1
Bacteria	OTU59	1	0.071531792	0	8
Bacteria	OTU60	1	0.071531792	0	8
Bacteria	OTU64	1	0.01010101	0	1
Bacteria	OTU66	1	0.01010101	0	1
Bacteria	OTU67	1	0.01010101	0	1
Bacteria	OTU78	1	0.075803982	0	6
Bacteria	OTU87	1	0.065088757	0	8
Bacteria	OTU90	1	0.070663812	0	7
Bacteria	OTU92	1	0.01010101	0	1
Bacteria	OTU95	1	0.073062731	0	7
Fungi	OTU109	1	0.01010101	0	1
Fungi	OTU110	1	0.069376314	0	7
Fungi	OTU112	1	0.074773414	0	6
Fungi	OTU115	1	0.01010101	0	1
Fungi	OTU118	1	0.073496659	0	7
Fungi	OTU122	1	0.01010101	0	1
Fungi	OTU123	1	0.076153846	0	6
Fungi	OTU124	1	0.075803982	0	6
Fungi	OTU125	1	0.06875	0	8
Fungi	OTU126	1	0.076153846	0	6
Fungi	OTU129	1	0.075342466	0	7

Fungi	OTU131	1	0.067576792	0	8
Fungi	OTU134	1	0.070663812	0	7
Fungi	OTU136	1	0.065088757	0	8
Fungi	OTU145	1	0.070014144	0	8
Fungi	OTU163	1	0.074548193	0	6
Fungi	OTU180	1	0.073825503	0	6
Fungi	OTU186	1	0.069767442	0	7

Bacterial and fungal co-occurrence network characteristics and hub microbes (Mutualism

Centralization of eigenvector	PageRank	Clustering coefficient	Hub	Hub node(degree>11,closeness centrality>0.08)
1	0.007532611	0.081028	0.626661	Yes
0.536881144	0.008824134	0.033333	0.32322	Yes
0.67221681	0.00645572	0.087912	0.416021	Yes
0.599967415	0.007703371	0.096154	0.207305	Yes
0.517721171	0.023396015	0.044872	0.038097	Yes
0.541444169	0.022840919	0.068182	0.0181	Yes
0.501905145	0.006652934	0.133333	0.188285	
0.361985792	0.00680231	0.1	0	
0.468482955	0.023944975	0.033333	0.143854	
0.441577224	0.027380303	0.1	0.113641	
0.309324074	0.008142094	0.069444	0.15266	
0.346803411	0.007299418	0.055556	0.076558	
0.349588893	0.00911486	0.055556	0.142972	
0.399660205	0.008957801	0.125	0.16063	
0.2128679	0.007281266	0.047619	0.097189	
0.317602253	0.006775856	0.119048	0.062596	
0.371828598	0.008617387	0.119048	0.100071	
0.259458567	0.005827387	0.133333	0.117965	
0.183605785	0.00698085	0.233333	0	
0.174017545	0.007014132	0.233333	0.143396	
0.266400754	0.008010886	0.066667	0.044156	
0.3742212	0.006569628	0.066667	0.017764	
0.255684435	0.011942178	0.033333	0.103603	
0.353733041	0.013630509	0.033333	0	
0.215773405	0.036011249	0.033333	0.161051	
0.209306361	0.007607615	0.1	0.0872	
0.237465738	0.00713585	0.05	0	
0.188445916	0.007300986	0.1	0.078658	
0.119761906	0.008166027	0.05	0.018922	
0.283716875	0.009111971	0.1	0.000531	
0.237820893	0.009214159	0.05	0.033172	
0.141199079	0.007719989	0.25	0	
0.13647351	0.007896	0.083333	0.039845	
0.063261929	0.005827387	0.166667	0.024835	
0.138523222	0.007635637	0.083333	0	
0.148419856	0.008724701	0	0	
0.301455561	0.007917416	0	0.116533	
0.142864586	0.015608448	0	0.041602	
0.037704196	0.01142209	0	0	
0.143806429	0.00743649	0	0	
0.060614261	0.01904373	0	0.062094	
0.062418149	0.005827387	0	0.004025	

0.037293117	0.00698085	0.166667	0
0.128798596	0.023207122	0	0.065734
0.001986221	0.015733945	0	0.000707
0.049188929	0.006550132	0	0
0.043355387	0.007376913	0.5	0
0.073301733	0.007014132	0	0
0.041228942	0.006713745	0.5	0
0.02530632	0.007065202	0	0
0.083351763	0.007644832	0	0.007865
0.196064814	0.006569628	0	0
0.020184786	0.012097763	0	0
0.062537464	0.006979283	0	0
0.016224346	0.008064587	0	0
0.007520752	0.007065707	0	0.051213
0.139343431	0.008216136	0	0
0.049859632	0.007788452	0	0
0.1065448	0.008894023	0	0
0.137651727	0.007339925	0	0
0.030845147	0.006652934	0	0
0.057541566	0.034668257	0	0
0.028272801	0.014696684	0	0.009719
0.091367614	0.016214998	0	0
0.016474513	0.022446386	0	0
0.003314942	0.005827387	0	0
0.014041897	0.008141095	0	0
0.024540844	0.007982878	0	0
0.017401997	0.013243383	0	0
1.47E-17	0.005827387	0	0
2.22E-17	0.005827387	0	0
2.00E-17	0.010780666	0	0
0.007318431	0.00747848	0	0
0.007318431	0.00747848	0	0
1.67E-17	0.005827387	0	0
2.19E-17	0.005827387	0	0
1.58E-17	0.010780666	0	0.000015
0.070345244	0.006482174	0	0
0.000232881	0.005827387	0	0
0.00437256	0.008794248	0	0
1.80E-17	0.005827387	0	0
0.020403286	0.007019789	0	0
2.27E-17	0.010780666	0	0
0.002967127	0.011832809	0	0
0.046859536	0.007350213	0	0
2.22E-17	0.010780666	0	0
0.027842488	0.007849211	0	0
2.33E-17	0.010780666	0	0
0.062948477	0.00645243	0	0
0.078816373	0.006249492	0	0
0.001902279	0.012682286	0	0
0.062948477	0.00645243	0	0
0.054928896	0.012611797	0	0

0.000881796	0.011833238	0	0
0.00437256	0.008794248	0	0
0.000232881	0.005827387	0	0
0.004834029	0.01153407	0	0
0.04244227	0.006550132	0	0
0.02209499	0.007896	0	0
0.003616545	0.011482381	0	0

)

Consensus_Lineage
Bacteria;Proteobacteria;Betaproteobacteria;Burkholderiales;Comamonadaceae
Bacteria;Bacteroidetes;Sphingobacteriia;Sphingobacteriales;env.OPS 17
Bacteria;Proteobacteria;Betaproteobacteria;Burkholderiales;Oxalobacteraceae
Bacteria;Actinobacteria;Actinobacteria;Micrococcales;Microbacteriaceae
Fungi;Ascomycota;Leotiomycetes;Helotiales;Helotiaceae;Tetracladium;
Fungi;Ascomycota

Supplementary Table 2-1 The inter-kingdom connections in each network

	Actinobacteria	Alphaproteobacteria	Betaproteobacteria	Flavobacteriia	Gammaproteobacteria
Agaricomycetes	0	0	0	2	0
Dothideomycetes	4	2	1	4	2
Leotiomycetes	9	0	15	11	5
Sordariomycetes	6	1	6	4	1
others	2	0	1	2	1

Supplementary Table 3-1 The edge information in the mutualism network

Source	Target	Type
OTU1	OTU2	Directed
OTU1	OTU4	Directed
OTU1	OTU5	Directed
OTU1	OTU10	Directed
OTU1	OTU101	Directed
OTU1	OTU130	Directed
OTU2	OTU3	Directed
OTU2	OTU4	Directed
OTU2	OTU5	Directed
OTU2	OTU6	Directed
OTU2	OTU7	Directed
OTU2	OTU11	Directed
OTU2	OTU17	Directed
OTU2	OTU33	Directed
OTU2	OTU102	Directed
OTU3	OTU4	Directed
OTU3	OTU6	Directed
OTU3	OTU8	Directed
OTU3	OTU10	Directed
OTU3	OTU12	Directed
OTU3	OTU33	Directed
OTU3	OTU35	Directed
OTU3	OTU49	Directed
OTU3	OTU65	Directed
OTU3	OTU101	Directed
OTU3	OTU105	Directed
OTU3	OTU106	Directed
OTU3	OTU124	Directed
OTU4	OTU6	Directed
OTU4	OTU8	Directed
OTU4	OTU13	Directed
OTU4	OTU14	Directed
OTU4	OTU15	Directed
OTU4	OTU17	Directed
OTU4	OTU21	Directed
OTU4	OTU28	Directed
OTU4	OTU78	Directed
OTU4	OTU102	Directed
OTU5	OTU8	Directed
OTU5	OTU14	Directed
OTU5	OTU40	Directed
OTU5	OTU45	Directed
OTU5	OTU101	Directed
OTU6	OTU7	Directed
OTU6	OTU9	Directed
OTU6	OTU13	Directed
OTU6	OTU15	Directed
OTU6	OTU17	Directed

OTU6	OTU18	Directed
OTU6	OTU21	Directed
OTU6	OTU30	Directed
OTU6	OTU34	Directed
OTU6	OTU35	Directed
OTU6	OTU47	Directed
OTU6	OTU49	Directed
OTU6	OTU65	Directed
OTU6	OTU101	Directed
OTU6	OTU102	Directed
OTU6	OTU103	Directed
OTU6	OTU105	Directed
OTU6	OTU106	Directed
OTU6	OTU114	Directed
OTU6	OTU128	Directed
OTU7	OTU9	Directed
OTU7	OTU11	Directed
OTU7	OTU20	Directed
OTU7	OTU54	Directed
OTU7	OTU103	Directed
OTU8	OTU10	Directed
OTU8	OTU16	Directed
OTU8	OTU18	Directed
OTU8	OTU28	Directed
OTU8	OTU47	Directed
OTU8	OTU56	Directed
OTU9	OTU10	Directed
OTU9	OTU12	Directed
OTU9	OTU33	Directed
OTU9	OTU36	Directed
OTU9	OTU43	Directed
OTU9	OTU105	Directed
OTU9	OTU113	Directed
OTU10	OTU26	Directed
OTU10	OTU37	Directed
OTU10	OTU101	Directed
OTU10	OTU106	Directed
OTU10	OTU113	Directed
OTU11	OTU13	Directed
OTU11	OTU39	Directed
OTU11	OTU106	Directed
OTU12	OTU15	Directed
OTU12	OTU105	Directed
OTU12	OTU118	Directed
OTU13	OTU102	Directed
OTU13	OTU105	Directed
OTU13	OTU106	Directed
OTU13	OTU108	Directed
OTU13	OTU112	Directed
OTU14	OTU27	Directed
OTU14	OTU102	Directed

OTU15	OTU30	Directed
OTU15	OTU33	Directed
OTU15	OTU34	Directed
OTU15	OTU37	Directed
OTU15	OTU46	Directed
OTU15	OTU65	Directed
OTU15	OTU101	Directed
OTU15	OTU106	Directed
OTU15	OTU111	Directed
OTU15	OTU119	Directed
OTU15	OTU123	Directed
OTU15	OTU126	Directed
OTU16	OTU17	Directed
OTU16	OTU19	Directed
OTU16	OTU20	Directed
OTU16	OTU29	Directed
OTU16	OTU101	Directed
OTU17	OTU34	Directed
OTU17	OTU106	Directed
OTU17	OTU119	Directed
OTU18	OTU24	Directed
OTU18	OTU114	Directed
OTU18	OTU180	Directed
OTU19	OTU46	Directed
OTU19	OTU95	Directed
OTU19	OTU101	Directed
OTU19	OTU106	Directed
OTU19	OTU128	Directed
OTU20	OTU27	Directed
OTU20	OTU32	Directed
OTU20	OTU103	Directed
OTU21	OTU22	Directed
OTU21	OTU33	Directed
OTU21	OTU37	Directed
OTU21	OTU43	Directed
OTU21	OTU65	Directed
OTU21	OTU103	Directed
OTU21	OTU116	Directed
OTU21	OTU163	Directed
OTU22	OTU138	Directed
OTU23	OTU164	Directed
OTU24	OTU84	Directed
OTU24	OTU101	Directed
OTU24	OTU105	Directed
OTU25	OTU30	Directed
OTU25	OTU104	Directed
OTU25	OTU108	Directed
OTU25	OTU116	Directed
OTU26	OTU52	Directed
OTU27	OTU45	Directed
OTU28	OTU47	Directed

OTU28	OTU169	Directed
OTU29	OTU105	Directed
OTU30	OTU45	Directed
OTU30	OTU103	Directed
OTU30	OTU113	Directed
OTU34	OTU164	Directed
OTU34	OTU167	Directed
OTU35	OTU47	Directed
OTU35	OTU102	Directed
OTU35	OTU103	Directed
OTU35	OTU105	Directed
OTU36	OTU145	Directed
OTU37	OTU44	Directed
OTU40	OTU110	Directed
OTU43	OTU101	Directed
OTU47	OTU108	Directed
OTU47	OTU113	Directed
OTU48	OTU67	Directed
OTU50	OTU51	Directed
OTU52	OTU111	Directed
OTU53	OTU59	Directed
OTU53	OTU60	Directed
OTU53	OTU101	Directed
OTU54	OTU108	Directed
OTU56	OTU90	Directed
OTU56	OTU134	Directed
OTU64	OTU122	Directed
OTU66	OTU109	Directed
OTU84	OTU125	Directed
OTU87	OTU140	Directed
OTU92	OTU115	Directed
OTU101	OTU103	Directed
OTU101	OTU108	Directed
OTU103	OTU105	Directed
OTU103	OTU106	Directed
OTU103	OTU129	Directed
OTU104	OTU131	Directed
OTU105	OTU138	Directed
OTU106	OTU108	Directed
OTU106	OTU111	Directed
OTU106	OTU167	Directed
OTU130	OTU186	Directed
OTU136	OTU140	Directed
OTU140	OTU169	Directed

Supplementary Table 4 Significant QTLs associated with the microbial interaction network

Number	Position	CHR	P	microbial network properties	gene	gene description	GO
1	2.4E+07	5	4.56E-07	Bu_altruism	AT5G60470	C2H2 and C2HC zinc fingers superfamily protein	nucleus；(GO:0005634)； DNA-binding transcription factor activity；(GO:0003700)； regulation of transcription, DNA-
2	2.4E+07	5	4.56E-07	Bu_altruism	AT5G6047	-	-
3	5633733	4	6.07E-07	Bu_altruism	PUM11	pumilio 11	cytoplasm；(GO:0005737)； mRNA binding；(GO:0003729)； posttranscriptional regulation of gene expression；(
4	1.3E+07	4	9.21E-07	Bu_altruism	-	-	-
5	2.4E+07	5	1.70E-06	Bu_altruism	AT5G60520	Late embryogenesis abundant (LEA) protein-like	molecular_function_unknown；(GO:0003674)； extracellular region；(GO:0005576
6	2.4E+07	5	2.10E-06	Bu_altruism	-	-	-
7	4865340	4	6.26E-06	Bu_altruism	-	-	-
8	1.4E+07	5	8.09E-06	Bu_altruism	-	-	-
9	5456536	3	8.09E-06	Bu_antagonism	Hrd1A	RING/U-box superfamily protein	ubiquitin-dependent protein catabolic process；(GO:0006511)； protein binding；(GO:0005515)； ubiquitin protein ligase activity；(GO:0061630)； ubiquitin-dependent ERAD pathway；(GO:0030433)； protein ubiquitination；(
10	7746139	1	1.09E-08	Bu_mutualis	-	-	-
11	1.4E+07	4	2.28E-08	Bu_mutualis	-	-	-
12	1.7E+07	5	2.28E-08	Bu_mutualis	-	-	-
13	8289541	2	2.28E-08	Bu_mutualism	AT2G19120	P-loop containing nucleoside triphosphate hydrolases superfamily protein	plasmodesma；(GO:0009506)； cytoplasm；(GO:0005737)； RNA binding；(GO:0003723)； biological_process_unknown；(GO:0008150)

14	4036563	1	2.53E-08	Bu_mutualism	AT1G11950	Transcription factor jumonji (jmjC) domain-containing protein	[illegible] activity (H3-K9 specific); (GO:0032454); nucleus; (GO:0005634); histone H3-K9 demethylation; (GO:0033169); regulation of transcription by RNA polymerase II; (GO:0006357); histone deacetylase complex; (GO:0000118); transcription coregulator activity; (GO:0003712); [illegible]
15	9696393	3	7.83E-08	Bu_mutualis	AT3G2648	hypothetical	-
16	8977674	2	1.07E-07	Bu_mutualis	SRF1	STRUBBELIG-	-
17	7009363	4	1.40E-07	Bu_mutualism	AT4G11590	F-box associated ubiquitination effector family protein	molecular_function_unknown; (GO:0003674); protein ubiquitination; (GO:0016567)
18	2.2E+07	1	2.01E-07	Bu_mutualism	ARPC3	This gene encodes one of seven	-
19	1.6E+07	1	2.30E-07	Bu_mutualism	PMI1	plastid movement impaired1	intracellular anatomical structure; (GO:0005622); response to osmotic stress; (GO:0006970); regulation of seed germination; (GO:0010029); cytosol; (GO:0005829); response to blue light; (GO:0009637); nucleus; (GO:0005634); regulation of abscisic acid-activated signaling pathway; (GO:0009787); chloroplast relocation; (GO:0009902);
20	1.5E+07	3	5.77E-07	Bu_mutualism	AT3G42850	Mevalonate/galactokinase family protein	galactose metabolic process; (GO:0006012); chloroplast; (GO:0009507); L-
21	1.7E+07	1	5.95E-07	Bu_mutualis	-	-	-
22	7747171	1	9.57E-07	Bu_mutualis	-	-	-

23	7749852	1	9.57E-07	Bu_mutualism	AT1G22010	hypothetical protein	molecular_function_unknown; (GO:0003674);
24	1.5E+07	5	9.57E-07	Bu_mutualis	-	-	-
25	3688388	2	1.38E-06	Bu_mutualis	AT2G0983	hypothetical	-
26	9405110	1	2.94E-06	Bu_mutualism	AT1G27090	glycine-rich protein	chloroplast; (GO:0009507); nucleus; (
27	2.9E+07	1	2.94E-06	Bu_mutualis	-	-	-
28	6358289	4	3.23E-06	Bu_mutualis	AT4G1020	F-box family	-
29	6221857	4	3.79E-06	Bu_mutualism	MES12	methyl esterase 12	methyl indole-3-acetate esterase activity; (GO:0080030); jasmonic acid metabolic process; (GO:0009694); salicylic acid metabolic process; (
30	2E+07	3	4.06E-06	Bu_mutualism	AT3G54750	downstream neighbor of Son	molecular_function_unknown; (GO:0003674); nucleus; (
31	8152846	1	4.63E-06	Bu_mutualism	FRO3	ferric reduction oxidase 3	oxidation-reduction process; (GO:0055114); ferric-chelate
32	1.3E+07	1	5.27E-06	Bu_mutualism	PUM17	pumilio 17	cytoplasm; (GO:0005737); posttranscriptional regulation of gene
33	7949361	3	5.79E-06	Bu_mutualism	AT3G22421	F-box/associated interaction domain	biological_process_unknown; (GO:0008150
34	2.2E+07	1	8.75E-06	Bu_mutualism	ARPC3	This gene encodes one of seven	-
35	5880477	3	4.10E-06	Con_aggressi	UPL6	ubiquitin protein	-
36	1.4E+07	5	5.92E-06	Con_aggressi	AT5G3622	hypothetical	-
37	6983342	4	6.16E-06	Con_aggressi	AT4G1150	-	-
38	5872184	3	9.52E-06	Con_aggressi	GA20X5	-	-
1	2.4E+07	5	1.28E-06	Con_altruism	AT5G60470	C2H2 and C2HC zinc fingers superfamily protein	nucleus; (GO:0005634); DNA-binding transcription factor activity; (
2	2.4E+07	5	1.28E-06	Con_altruism	AT5G60470	C2H2 and C2HC zinc fingers superfamily protein	nucleus; (GO:0005634); DNA-binding transcription factor activity; (
3	5633733	4	1.37E-06	Con_altruism	PUM11	pumilio 11	cytoplasm; (GO:0005737); mRNA binding; (
4	1.3E+07	4	2.86E-07	Con_altruism	-	-	-
5	2.4E+07	5	4.11E-06	Con_altruism	AT5G60520	Late embryogenesis	molecular_function_unknown; (GO:0003674
6	2.4E+07	5	5.23E-06	Con_altruism	-	-	-
7	4865340	4	7.42E-06	Con_altruism	-	-	-
39	1.7E+07	4	7.93E-06	Con_altruism	-	-	-
40	9967780	5	2.03E-08	Con_mutuali	-	-	-
41	1.4E+07	5	8.04E-08	Con_mutuali	AT5G3560	hypothetical	-
42	1.4E+07	5	8.04E-08	Con_mutuali	AT5G3561	-	-
43	2.1E+07	3	1.19E-07	Con_mutuali	-	-	-

44	1.9E+07	1	1.19E-07	Con_mutualism	DWF5	Ergosterol biosynthesis ERG4/ERG24 family	7-dehydrocholesterol reductase activity; (GO:0047598); plasma membrane; (GO:0005886); oxidoreductase activity, acting on the CH-CH group of donors; (GO:0016627); unidimensional cell growth; (GO:0009826); sterol biosynthetic process; (GO:0016126
45	5543599	1	1.24E-07	Con_mutualism	RAD23A	The protein encoded by this gene is one of two human homologs of Saccharomyces cerevisiae Rad23	nucleotide-excision repair; (GO:0006289); proteasome-mediated ubiquitin-dependent protein catabolic process; (GO:0043161); damaged DNA binding; (
46	2866886	4	1.24E-07	Con_mutuali	-	-	-
47	1.8E+07	1	1.24E-07	Con_mutualism	AT1G49450	Transducin/WD40 repeat-like	nucleus; (GO:0005634);
48	358189	2	1.24E-07	Con_mutualism	AT2G01820	Leucine-rich repeat protein kinase family protein	plasmodesma; (GO:0009506); plant-type cell wall; (GO:0009505); plasma membrane; (GO:0005886); protein phosphorylation; (GO:0006468); pollen
49	1.4E+07	5	4.46E-07	Con_mutuali	AT5G3561	-	-
50	5545724	1	5.62E-07	Con_mutualism	RAD23A	The protein encoded by this gene is one of two human homologs of Saccharomyces cerevisiae Rad23	nucleotide-excision repair; (GO:0006289); proteasome-mediated ubiquitin-dependent protein catabolic process; (GO:0043161); damaged DNA binding; (
51	358617	2	5.62E-07	Con_mutualism	AT2G01820	Leucine-rich repeat protein kinase family protein	plasmodesma; (GO:0009506); plant-type cell wall; (GO:0009505); plasma membrane; (GO:0005886); protein phosphorylation; (GO:0006468); pollen
52	6522489	4	5.62E-07	Con_mutuali	MEE53	Cysteine/Histidine	-
53	9472315	5	6.48E-07	Con_mutuali	-	-	-

54	5520641	1	2.01E-06	Con_mutualism	WAKL6	wall associated kinase-like 6	kinase activity; (GO:0016301); plant-type cell wall; (GO:0009505); polysaccharide binding; (GO:0030247); cell
55	1.7E+07	2	2.01E-06	Con_mutualism	AT2G41590	Ta11-like non-LTR retrotransposon	nucleus; (GO:0005634); molecular_function_un
56	1.2E+07	1	2.01E-06	Con_mutuali	HAF01	HAC13 protein	-
57	1.4E+07	5	2.04E-06	Con_mutuali	-	-	-
58	1.5E+07	4	3.23E-06	Con_mutualism	AT4G30730	hypothetical protein	chloroplast; (GO:0009507); biological_process_unk
59	7837282	2	3.23E-06	Con_mutuali	EMB2296	Ribosomal protein	-
60	1602807	2	3.23E-06	Con_mutuali	-	-	-
61	2.4E+07	5	4.75E-06	Con_mutuali	-	-	-
62	9293684	4	4.91E-06	Con_mutualism	AT4G16490	ARM repeat superfamily	cytoplasm; (GO:0005737);
63	2.8E+07	1	5.71E-06	Con_mutuali	CYP721A1	cytochrome P450,	-
64	6001018	1	6.02E-06	Con_mutuali	TRFL3	TRF-like 3	-
65	1.5E+07	4	6.02E-06	Con_mutuali	-	-	-
66	465269	5	6.02E-06	Con_mutualism	EXPA9	expansin A9	unidimensional cell growth; (GO:0009826); extracellular region; (GO:0005576
67	1.3E+07	1	6.02E-06	Con_mutuali	-	-	-
68	1475536	3	8.79E-06	Con_mutualism	AT3G05190	D-aminoacid aminotransferase-	chloroplast; (GO:0009507);
69	1406662	4	9.33E-06	Con_mutualism	GRH1	GRR1-like protein 1	protein binding; (GO:0005515); response to auxin; (GO:0009733); carbon catabolite repression of transcription by glucose; (GO:0045014); ubiquitin-dependent protein catabolic process; (GO:0006511); inositol hexakisphosphate binding; (GO:0000822); auxin binding; (GO:0010011); stamen
70	1.3E+07	3	1.82E-07	Cu_aggressio	-	-	-
71	1.3E+07	1	7.23E-07	Cu_aggressio	-	-	-
72	1.1E+07	5	1.46E-06	Cu_aggressio	-	-	-
73	1.4E+07	5	1.58E-06	Cu_aggressio	-	-	-
74	4653504	1	3.62E-06	Cu_aggressio	-	-	-
75	263618	3	4.71E-06	Cu_aggression	AT3G01720	peptidyl serine alpha-galactosyltransfera	transferase activity, transferring glycosyl groups; (GO:0016757

76	2194772	3	5.53E-06	Cu_aggression	PDE320	pigment defective 320	nucleus; (GO:0005634); acylglycerol transport; (GO:0034196); chloroplast envelope; (GO:0009941); cytosol; (GO:0005829); chloroplast outer membrane; (GO:0009707); chloroplast; (GO:0009507); ER to
77	2.7E+07	1	6.18E-06	Cu_aggressio	AT1G7207	Chaperone DnaJ-	cytoplasm; (
78	4348611	1	6.45E-06	Cu_aggression	AT1G12760	Zinc finger, C3HC4 type (RING finger) family protein	protein ubiquitination; (GO:0016567); ubiquitin-protein transferase activity; (GO:0004842);
79	1.2E+07	3	7.40E-06	Cu_aggressio	AT3G3040	hypothetical	-
80	1.1E+07	5	7.88E-06	Cu_aggressio	AT5G2852	hypothetical	-
81	2.5E+07	1	3.04E-06	Cu_altruism	AT1G67800	Copine (Calcium-dependent phospholipid-binding protein) family	nucleus; (GO:0005634); protein binding; (GO:0005515); extracellular region; (GO:0005576);
82	2.5E+07	1	3.08E-06	Cu_altruism	AT1G67800	Copine (Calcium-dependent phospholipid-binding protein) family	nucleus; (GO:0005634); protein binding; (GO:0005515); extracellular region; (GO:0005576);
83	2.5E+07	1	9.79E-06	Cu_altruism	AT1G67792	Natural antisense transcript overlaps with	biological_process_unknown; (GO:0008150);
84	2.5E+07	1	9.79E-06	Cu_altruism	AT1G67792	Natural antisense transcript overlaps with	biological_process_unknown; (GO:0008150);
73	1.4E+07	5	4.61E-09	Cu_antagonis	-	-	-
74	4653504	1	9.70E-07	Cu_antagonis	-	-	-
76	2194772	3	1.47E-08	Cu_antagonism	PDE320	pigment defective 320	nucleus; (GO:0005634); acylglycerol transport; (GO:0034196); chloroplast envelope; (GO:0009941); cytosol; (GO:0005829); chloroplast outer membrane; (GO:0009707); chloroplast; (GO:0009507); ER to

85	3556389	5	9.39E-13	Cu_antagonism	UAP56a	DEAD/DEAH box RNA helicase family protein	ATPase activity; (GO:0016887); mRNA binding; (GO:0003729); nucleus; (GO:0005634); response to cadmium ion; (GO:0046686); nucleolus; (GO:0005730); protein binding; (
86	1.7E+07	3	2.38E-11	Cu_antagonism	AT3G46420	Leucine-rich repeat protein kinase family protein	protein phosphorylation; (GO:0006468); extracellular region; (
87	9984166	5	7.27E-11	Cu_antagonism	AT5G27950	P-loop containing nucleoside triphosphate hydrolases superfamily protein	ATP-dependent microtubule motor activity, plus-end-directed; (GO:0008574); microtubule-based movement; (GO:0007018); cytoskeleton-dependent
88	3280058	5	7.69E-11	Cu_antagonis	-	-	-
89	3564738	5	1.30E-10	Cu_antagonis	AT5G0185	-	-
90	1.3E+07	5	1.75E-10	Cu_antagonism	PTM	PHD type transcription factor with transmembrane domain protein (PTM)	chloroplast envelope; (GO:0009941); nucleus; (GO:0005634); protein binding; (GO:0005515); methylated histone
91	3509204	5	2.31E-10	Cu_antagonis	-	-	-
92	1.4E+07	5	3.10E-10	Cu_antagonis	AT5G3534	hypothetical	-
93	6328771	4	3.38E-10	Cu_antagonism	AT4G10150	RING/U-box superfamily protein	ubiquitin-dependent protein catabolic process; (GO:0006511); ubiquitin protein
94	1.4E+07	5	3.55E-10	Cu_antagonism	AT5G35525	PLAC8 family protein	cytoplasm; (GO:0005737); biological_process_unk
95	1.4E+07	5	3.60E-10	Cu_antagonis	-	-	-
96	1.4E+07	5	9.89E-10	Cu_antagonis	AT5G3533	hypothetical	-
97	1.4E+07	5	1.10E-09	Cu_antagonism	AT5G35475	General transcription factor	molecular_function_unknown; (GO:0003674
98	3408362	5	1.43E-09	Cu_antagonism	AT5G10780	ER membrane protein complex subunit-like protein	nucleus; (GO:0005634); ER membrane protein complex; (
99	3409353	5	1.43E-09	Cu_antagonism	AT5G10780	ER membrane protein complex subunit-like protein	nucleus; (GO:0005634); ER membrane protein complex; (
100	3410949	5	1.43E-09	Cu_antagonis	UBP22	ubiquitin-specific	-

101	3416182	5	1.43E-09	Cu_antagonism	AT5G10800	RNA recognition motif (RRM)-	cytoplasm； (GO:0005737)；
102	3419070	5	1.43E-09	Cu_antagonism	ER	Leucine-rich receptor-like protein kinase family protein	regulation of plant organ morphogenesis； (GO:1905421)； plasma membrane； (GO:0005886)； regulation of cell division； (GO:0051302)； protein binding； (GO:0005515)； regulation of cell adhesion； (GO:0030155)； polarity specification of adaxial/abaxial axis； (GO:0009944)； signaling receptor binding； (GO:0005102)； receptor serine/threonine kinase binding； (GO:0033612)；
103	3421461	5	1.43E-09	Cu_antagonis	AT5G1082	Major facilitator	chloroplast； (
104	3745460	5	2.13E-09	Cu_antagonism	AT5G11650	alpha/beta-Hydrolases superfamily protein	Golgi apparatus； (GO:0005794)； lipase activity； (GO:0016298)； endoplasmic
105	4514041	3	2.20E-09	Cu_antagonism	BGAL1	beta galactosidase 1	plant-type cell wall； (GO:0009505)； vacuole； (GO:0005773)； beta-galactosidase activity； (GO:0004565)； plasmodesma； (
106	3670243	5	2.65E-09	Cu_antagonism	AT5G11480	P-loop containing nucleoside	nucleus； (GO:0005634)； GTP
107	8074644	3	5.30E-09	Cu_antagonis	-	-	-
108	1.4E+07	5	5.37E-09	Cu_antagonis	AT5G3555	hypothetical	-
109	5109002	4	6.23E-09	Cu_antagonis	-	-	-
110	2E+07	1	7.03E-09	Cu_antagonism	AT1G52510	alpha/beta-Hydrolases superfamily protein	chloroplast envelope； (GO:0009941)； chloroplast stroma； (GO:0009570)；
111	2E+07	1	7.03E-09	Cu_antagonism	FRS6	FAR1-related sequence 6	nucleus； (GO:0005634)； regulation of
112	2E+07	1	7.03E-09	Cu_antagonis	-	-	-
113	3410142	5	7.13E-09	Cu_antagonis	UBP22	ubiquitin-specific	-

114	3603933	3	7.17E-09	Cu_antagonism	MYB65	myb domain protein 65	nucleus; (GO:0005634); anther development; (GO:0048653); DNA-binding transcription factor activity; (GO:0003700); positive regulation of programmed cell death; (GO:0043068); anther wall tapetum morphogenesis; (GO:0048655); transcription regulatory region sequence-
115	3604005	3	7.17E-09	Cu_antagonism	MYB65	myb domain protein 65	nucleus; (GO:0005634); anther development; (GO:0048653); DNA-binding transcription factor activity; (GO:0003700); positive regulation of programmed cell death; (GO:0043068); anther wall tapetum morphogenesis; (GO:0048655); transcription regulatory region sequence-
116	3562004	5	7.77E-09	Cu_antagonis	-	-	-
117	1.3E+07	5	9.83E-09	Cu_antagonism	AT5G35230	hypothetical protein	molecular_function_unknown; (GO:0003674);
118	2E+07	1	1.03E-08	Cu_antagonism	AT1G52710	Rubredoxin-like superfamily protein	cytochrome-c oxidase activity; (GO:0004129); mitochondrial envelope; (GO:0005740); mitochondrial electron
119	1.3E+07	5	1.28E-08	Cu_antagonism	AT5G35230	hypothetical protein	molecular_function_unknown; (GO:0003674);
120	1E+07	5	1.54E-08	Cu_antagonis	AT5G2797	ARM repeat	plasma membrane; (
121	1E+07	5	1.54E-08	Cu_antagonis	AT5G2797	ARM repeat	plasma membrane; (
122	7078163	1	1.71E-08	Cu_antagonis	-		-
123	1.6E+07	4	1.75E-08	Cu_antagonis	EDA39	calmodulin-	-
124	3605408	3	1.88E-08	Cu_antagonism	AT3G11450	DnaJ and Myb-like DNA-binding domain-containing protein	cytosol; (GO:0005829); embryo development ending in seed dormancy; (GO:0009793); chromatin silencing; (GO:0006342);
125	6115036	2	2.26E-08	Cu_antagonis	AT2G1440	hypothetical	-

126	3408858	5	2.28E-08	Cu_antagonism	AT5G10780	ER membrane protein complex subunit-like protein	nucleus；(GO:0005634)； ER membrane protein complex；(
127	3422619	5	2.28E-08	Cu_antagonis	AT5G1082	Major facilitator	chloroplast；(
128	6106176	1	2.52E-08	Cu_antagonis	-	-	-
129	2082367	4	2.63E-08	Cu_antagonis	AT4G0429	hypothetical	-
130	3635394	3	2.66E-08	Cu_antagonis	ACLB-1	ATP citrate lyase,	-
131	3638338	3	2.66E-08	Cu_antagonism	CASP2	This gene encodes a member of the	-
132	9957213	5	2.70E-08	Cu_antagonis	SADHU5-2	hypothetical	-
133	3829977	3	2.76E-08	Cu_antagonism	AT3G12020	P-loop containing nucleoside triphosphate hydrolases superfamily protein	chloroplast；(GO:0009507)； microtubule binding；(GO:0008017)； microtubule；(GO:0005874)；
134	6782787	1	2.84E-08	Cu_antagonis	-	-	-
135	6616766	5	2.85E-08	Cu_antagonis	SUTLR3;5	-	-
136	1.4E+07	5	2.97E-08	Cu_antagonis	AT5G3549	hypothetical	-
137	3277274	5	3.16E-08	Cu_antagonis	-	-	-
138	2E+07	2	3.47E-08	Cu_antagonis	CRP6	-	-
139	2E+07	1	3.49E-08	Cu_antagonism	AT1G52510	alpha/beta-Hydrolases superfamily protein	chloroplast envelope；(GO:0009941)； chloroplast stroma；(GO:0009570)；
140	2E+07	5	3.98E-08	Cu_antagonis	-	-	-
141	1.3E+07	5	4.61E-08	Cu_antagonism	AT5G35200	ENTH/ANTH/VHS superfamily protein	clathrin-coated vesicle；(GO:0030136)； vesicle budding from membrane；(GO:0006900)； SNARE binding；(GO:0000149)； clathrin heavy chain binding；(GO:0032050)； plasma
142	7049722	5	4.83E-08	Cu_antagonis	-	-	-
143	2E+07	1	4.91E-08	Cu_antagonism	CDT1	The protein encoded by this gene is involved in	nucleus；(GO:0005634)； detoxification of
144	3557596	5	5.20E-08	Cu_antagonis	GLR2.6	glutamate receptor	-
145	2E+07	1	5.21E-08	Cu_antagonism	AT1G52730	Transducin/WD40 repeat-like superfamily protein	cytoplasm；(GO:0005737)； Cul4-RING E3 ubiquitin ligase complex；(GO:0080008)；

146	6598438	5	5.34E-08	Cu_antagonism	ASP2	-	2-oxoglutarate metabolic process; (GO:0006103); mitochondrion; (GO:0005739); copper ion binding; (GO:0005507); L-aspartate:2-oxoglutarate aminotransferase activity; (GO:0004069); plasma membrane; (GO:0005886); pyridoxal phosphate binding; (GO:0030170); cellular
147	1.2E+07	4	6.07E-08	Cu_antagonism	AT4G22850	SNARE associated Golgi protein family	plasma membrane; (GO:0005886); biological_process_unk
148	1.2E+07	2	6.35E-08	Cu_antagonis	-	-	-
149	1.2E+07	2	6.35E-08	Cu_antagonism	EXO70H5	exocyst subunit exo70 family	pollen tube; (GO:0090406);
150	1.4E+07	1	6.58E-08	Cu_antagonis	-	-	-
151	2E+07	5	6.74E-08	Cu_antagonism	AT5G50310	Galactose oxidase/kelch	biological_process_unknown; (GO:0008150
152	2E+07	5	6.74E-08	Cu_antagonism	ELO3	radical SAM domain-containing protein / GCN5-related N-acetyltransferase (GNAT) family protein	sphingolipid biosynthetic process; (GO:0030148); mitochondrion; (GO:0005739); fatty acid elongation, polyunsaturated fatty acid; (GO:0034626); endoplasmic reticulum; (
153	2E+07	5	6.74E-08	Cu_antagonism	ELO3	radical SAM domain-containing protein / GCN5-related N-acetyltransferase (GNAT) family protein	sphingolipid biosynthetic process; (GO:0030148); mitochondrion; (GO:0005739); fatty acid elongation, polyunsaturated fatty acid; (GO:0034626); endoplasmic reticulum; (
154	6830174	5	7.39E-08	Cu_antagonis	P1	movement protein	-
155	1.3E+07	5	7.57E-08	Cu_antagonis	-	-	-
156	6109521	1	7.63E-08	Cu_antagonism	PEPR2	PEP1 receptor 2	protein serine kinase activity; (GO:0106310); peptide binding; (GO:0042277); kinase activity; (GO:0016301); protein binding; (GO:0005515); plasma membrane; (GO:0005886);

157	2E+07	1	7.96E-08	Cu_antagonis	-	-	-
158	2E+07	1	7.96E-08	Cu_antagonis	-	-	-
159	2E+07	1	7.96E-08	Cu_antagonis	-	-	-
160	2E+07	1	7.96E-08	Cu_antagonism	AT1G53025	Ubiquitin-conjugating enzyme family protein	nucleus; (GO:0005634); postreplication repair; (GO:0006301); protein K63-linked
161	2E+07	1	7.96E-08	Cu_antagonism	AT1G53025	Ubiquitin-conjugating enzyme family protein	nucleus; (GO:0005634); postreplication repair; (GO:0006301); protein K63-linked
162	2E+07	1	7.96E-08	Cu_antagonis	-	-	-
163	2E+07	1	7.96E-08	Cu_antagonism	AT1G53035	transmembrane protein	molecular_function_unknown; (GO:0003674); chloroplast; (
164	2E+07	1	7.96E-08	Cu_antagonis	-	-	-
165	2E+07	1	7.96E-08	Cu_antagonis	-	-	-
166	2E+07	1	7.96E-08	Cu_antagonism	ATMAP4K ALPHA1	Protein kinase superfamily	-
167	2E+07	1	8.16E-08	Cu_antagonism	AT1G53860	Remorin family protein	biological_process_unknown; (GO:0008150);
168	6370884	5	9.68E-08	Cu_antagonism	AT5G19050	alpha/beta-Hydrolases	biological_process_unknown; (GO:0008150
169	3648231	5	1.03E-07	Cu_antagonism	AT5G11430	SPOC domain / Transcription	nucleus; (GO:0005634);
170	3417902	5	1.06E-07	Cu_antagonism	AT5G10800	AT5G10800	cytoplasm; (GO:0005737);
171	1.9E+07	2	1.11E-07	Cu_antagonism	UNE5	thioredoxin family protein	plant-type cell wall; (GO:0009505); vacuolar membrane; (GO:0005774); response to cadmium ion; (GO:0046686); extracellular region; (GO:0005576); pollen tube development; (GO:0048868); endoplasmic reticulum; (GO:0005783); embryo development
172	3487466	5	1.30E-07	Cu_antagonism	AT5G11020	Protein kinase superfamily protein	protein phosphorylation; (GO:0006468); phosphorylation; (GO:0016310);
173	7898106	3	1.33E-07	Cu_antagonis	AT3G2234	hypothetical	0

174	5150464	4	1.34E-07	Cu_antagonism	KNAT1	homeobox knotted-like protein	transcription regulatory region sequence-specific DNA binding; (GO:0000976); protein binding; (GO:0005515); DNA-binding transcription factor activity; (GO:0003700); xylem and phloem pattern formation; (GO:0010051); nucleus; (
175	7886932	3	1.38E-07	Cu_antagonis	-	-	-
176	2E+07	1	1.40E-07	Cu_antagonis	SRF6	STRUBBELIG-	-
177	2E+07	1	1.40E-07	Cu_antagonis	RPT1A	regulatory particle	-
178	2E+07	1	1.40E-07	Cu_antagonism	AT1G53780	26S proteasome regulatory complex ATPase	cytoplasm; (GO:0005737); ATPase activity; (GO:0016887); protein catabolic process; (GO:0030163);
179	6824998	5	1.43E-07	Cu_antagonism	AT5G20220	zinc knuckle (CCHC-type) family protein	nucleus; (GO:0005634); nucleic acid binding; (GO:0003676); mRNA binding; (GO:0003729); cytoplasm; (
180	6825667	5	1.43E-07	Cu_antagonism	AT5G20220	zinc knuckle (CCHC-type) family protein	nucleus; (GO:0005634); nucleic acid binding; (GO:0003676); mRNA binding; (GO:0003729); cytoplasm; (
181	2E+07	1	1.58E-07	Cu_antagonis	PAP5	purple acid	-
182	1.3E+07	5	1.59E-07	Cu_antagonis	-	-	-
183	2E+07	1	1.64E-07	Cu_antagonism	AT1G53930	Ubiquitin-like superfamily protein	protein ubiquitination; (GO:0016567); nucleus; (GO:0005634); cytoplasm; (GO:0005737);
184	1.5E+07	4	1.68E-07	Cu_antagonis	PKR2	chromatin	-
185	3415727	5	1.79E-07	Cu_antagonism	AT5G10800	RNA recognition motif (RRM)-	cytoplasm; (GO:0005737);
186	2E+07	1	2.09E-07	Cu_antagonism	AT1G53025	Ubiquitin-conjugating enzyme family protein	nucleus; (GO:0005634); postreplication repair; (GO:0006301); protein K63-linked
187	3572047	5	2.09E-07	Cu_antagonis	GLR2.5	glutamate receptor	-
188	3278886	5	2.13E-07	Cu_antagonis	-	-	-

189	1.3E+07	5	2.15E-07	Cu_antagonism	AT5G35180	ENHANCED DISEASE RESISTANCE protein (DUF1336)	plasma membrane; (GO:0005886); lipid binding; (GO:0008289); nucleus; (
190	9968053	5	2.19E-07	Cu_antagonis	-	-	-
191	1.2E+07	4	2.23E-07	Cu_antagonism	AT4G22860	TPX2-LIKE Group A family with aurora binding andTPX2 domains. Activator of aurora kinase activity	nucleus; (GO:0005634); nuclear microtubule; (GO:0005880); mitochondrion; (GO:0005739); activation of protein kinase activity; (GO:0032147); protein kinase activator activity; (GO:0030295
192	3649416	5	2.33E-07	Cu_antagonism	AT5G11430	SPOC domain / Transcription	nucleus; (GO:0005634);
193	2E+07	5	2.33E-07	Cu_antagonism	AT5G50310	Galactose oxidase/kelch	biological_process_unknown; (GO:0008150
194	2.5E+07	5	2.36E-07	Cu_antagonism	RMI1		resolution of meiotic recombination intermediates; (GO:0000712); nucleus; (GO:0005634); double-strand break repair via homologous
195	2E+07	1	2.40E-07	Cu_antagonism	AT1G53030	encodes a copper chaperone, can functional complements the	copper chaperone activity; (GO:0016531); cytosol; (GO:0005829); copper
196	3678558	5	2.65E-07	Cu_antagonism	AT5G11500	coiled-coil protein	biological_process_unknown; (GO:0008150); nucleus; (
197	4452230	3	2.65E-07	Cu_antagonis	PUT4	Amino acid	-
198	1.6E+07	3	2.70E-07	Cu_antagonism	DCL3	dicer-like 3	ribonuclease III activity; (GO:0004525); protein binding; (GO:0005515); nucleolus; (GO:0005730); RNA binding; (GO:0003723); nucleus; (GO:0005634); maintenance of DNA methylation; (GO:0010216); nucleic
199	3737307	5	2.70E-07	Cu_antagonism	AT5G11610	Exostosin family protein	transferase activity, transferring glycosyl groups; (GO:0016757); protein
200	3741348	5	2.70E-07	Cu_antagonis	NOXY2	hypothetical	-

201	3744717	5	2.70E-07	Cu_antagonism	AT5G11650	alpha/beta-Hydrolases superfamily protein	Golgi apparatus; (GO:0005794); lipase activity; (GO:0016298); endoplasmic
202	2E+07	1	2.73E-07	Cu_antagonism	AT1G53120	RNA-binding S4 domain-containing	biological_process_unknown; (GO:0008150
203	3646210	3	2.74E-07	Cu_antagonism	TBL8	TRICHOME BIREFRINGENCE-LIKE 8	Golgi apparatus; (GO:0005794); chloroplast; (
204	6107495	1	2.78E-07	Cu_antagonism	PEPR2	PEP1 receptor 2	protein serine kinase activity; (GO:0106310); peptide binding; (GO:0042277); kinase activity; (GO:0016301); protein binding; (GO:0005515); plasma membrane; (GO:0005886);
205	6111419	1	2.78E-07	Cu_antagonism	CSTF77	Tetratricopeptide repeat (TPR)-like superfamily protein	gene silencing by RNA; (GO:0031047); protein binding; (GO:0005515); nucleus; (GO:0005634); mRNA binding; (GO:0003729); regulation of gene
206	6113415	1	2.78E-07	Cu_antagonism	CSTF77	Tetratricopeptide repeat (TPR)-like superfamily protein	gene silencing by RNA; (GO:0031047); protein binding; (GO:0005515); nucleus; (GO:0005634); mRNA binding; (GO:0003729); regulation of gene
207	6114594	1	2.78E-07	Cu_antagonism	CSTF77	Tetratricopeptide repeat (TPR)-like superfamily protein	gene silencing by RNA; (GO:0031047); protein binding; (GO:0005515); nucleus; (GO:0005634); mRNA binding; (GO:0003729); regulation of gene
208	6118737	1	2.78E-07	Cu_antagonis	-	-	-
209	6120859	1	2.78E-07	Cu_antagonism	SUVH7	histone-lysine N-methyltransferase, H3 lysine-9 specific SUVH3-	histone lysine methylation; (GO:0034968); zinc ion binding; (
210	2430191	5	2.85E-07	Cu_antagonism	AT5G07670	RNI-like superfamily protein	SCF ubiquitin ligase complex; (GO:0019005); SCF-
211	2E+07	1	3.01E-07	Cu_antagonis	-	-	-

212	6779815	5	3.13E-07	Cu_antagonism	NUDX19	nudix hydrolase homolog 19	chloroplast; (GO:0009507); peroxisome; (GO:0005777); cytosol; (GO:0005829); NADH metabolic process; (GO:0006734
213	1.5E+07	4	3.14E-07	Cu_antagonis	UTr7	UDP-N-	-
214	2E+07	5	3.27E-07	Cu_antagonism	ELO3	radical SAM domain-containing protein / GCN5-related N-acetyltransferase (GNAT) family protein	sphingolipid biosynthetic process; (GO:0030148); mitochondrion; (GO:0005739); fatty acid elongation, polyunsaturated fatty acid; (GO:0034626); endoplasmic reticulum; (
215	2.7E+07	1	3.39E-07	Cu_antagonis	-	-	-
216	3683036	5	3.62E-07	Cu_antagonism	MYB3R-4	myb domain protein 3r-4	negative regulation of cellular response to caffeine; (GO:1901181); nucleus; (GO:0005634); protein binding; (GO:0005515); positive regulation of transcription, DNA-templated; (GO:0045893); DNA-binding transcription factor activity, RNA polymerase II-specific; (GO:0000981); DNA-binding transcription
217	3686139	5	3.62E-07	Cu_antagonism	ASP3	-	cytosol; (GO:0005829); L-aspartate:2-oxoglutarate aminotransferase activity; (GO:0004069); glutamate metabolic process; (GO:0006536); plastid; (GO:0009536); nitrogen compound metabolic process; (GO:0006807); leaf

218	3686741	5	3.62E-07	Cu_antagonism	ASP3	-	cytosol； (GO:0005829)； L-aspartate:2-oxoglutarate aminotransferase activity； (GO:0004069)； glutamate metabolic process； (GO:0006536)； plastid； (GO:0009536)； nitrogen compound metabolic process； (GO:0006807)； leaf
219	3687178	5	3.62E-07	Cu_antagonism	ASP3	-	cytosol； (GO:0005829)； L-aspartate:2-oxoglutarate aminotransferase activity； (GO:0004069)； glutamate metabolic process； (GO:0006536)； plastid； (GO:0009536)； nitrogen compound metabolic process； (GO:0006807)； leaf
220	3688241	5	3.62E-07	Cu_antagonis	-	-	-
221	1.9E+07	1	3.84E-07	Cu_antagonism	AT1G50140	P-loop containing nucleoside triphosphate hydrolases	cellular_component_unknown； (GO:0005575)； ATP binding； (GO:0005524)；
222	1.4E+07	5	3.85E-07	Cu_antagonis	-	-	-
223	6785001	1	3.86E-07	Cu_antagonis	CYP722A1	cytochrome P450,	-
224	1.3E+07	4	3.90E-07	Cu_antagonis	ACYB-2	Cytochrome	-
225	6509352	5	3.93E-07	Cu_antagonism	ARIA	ARM repeat protein interacting with ABF2	plasma membrane； (GO:0005886)； protein ubiquitination； (GO:0016567)； response to salt stress； (GO:0009651)； response to abscisic
226	3547479	5	4.02E-07	Cu_antagonis	VAMP713	vesicle-associated	-
227	2E+07	1	4.16E-07	Cu_antagonism	SPA4	SPA1-related 4	response to blue light； (GO:0009637)； chloroplast； (GO:0009507)； protein binding； (GO:0005515)； Cul4-RING E3 ubiquitin ligase complex； (
228	1.3E+07	5	4.19E-07	Cu_antagonis	-	-	-
229	2.5E+07	5	4.33E-07	Cu_antagonis	FLS4	flavonol synthase	-

230	9976088	5	4.51E-07	Cu_antagonism	AT5G27944	MADS-box transcription factor family protein	RNA polymerase II cis-regulatory region sequence-specific DNA binding；(GO:0000978)； protein dimerization activity；(GO:0046983)； DNA-binding transcription factor activity, RNA polymerase II-
231	2.7E+07	1	4.62E-07	Cu_antagonis	AT1G7222	RING/U-box	protein ubiquitination；
232	2.7E+07	1	4.62E-07	Cu_antagonism	AT1G72230	Cupredoxin superfamily protein	electron transfer activity；(GO:0009055)； anchored component of membrane；(
233	3692122	5	4.62E-07	Cu_antagonis	-	-	-
234	3549811	5	4.78E-07	Cu_antagonis	-	-	-
235	1.3E+07	5	4.94E-07	Cu_antagonis	-	-	-
236	2E+07	1	5.05E-07	Cu_antagonis	-	-	-
237	8103005	3	5.20E-07	Cu_antagonis	-	-	-
238	2E+07	1	5.49E-07	Cu_antagonism	SPA4	flavonol synthase 4	response to blue light；(GO:0009637)； chloroplast；(GO:0009507)； protein binding；(GO:0005515)； Cul4-RING E3 ubiquitin ligase complex；(
239	6800581	5	5.59E-07	Cu_antagonism	HBP5	SOUL heme-binding family protein	chloroplast thylakoid membrane；(GO:0009535)； chloroplast；(GO:0009507)； cytoplasm；(GO:0005737)； protein binding；(
240	3931991	3	5.75E-07	Cu_antagonism	AT3G12350	F-box family protein	chloroplast；(GO:0009507)； biological_process_unk
241	6107419	1	6.13E-07	Cu_antagonism	PEPR2	PEP1 receptor 2	protein serine kinase activity；(GO:0106310)； peptide binding；(GO:0042277)； kinase activity；(GO:0016301)； protein binding；(GO:0005515)； plasma membrane；(GO:0005886)；
242	2E+07	1	6.15E-07	Cu_antagonism	AT1G53705	putative aminoacyl-tRNA ligase	biological_process_unknown；(GO:0008150)；

243	2E+07	1	6.22E-07	Cu_antagonism	NAM	NAC (No Apical Meristem) domain transcriptional regulator superfamily protein	nucleus; (GO:0005634); integument development; (GO:0080060); DNA-binding transcription factor activity; (GO:0003700); regulation of embryonic
244	1E+07	3	6.28E-07	Cu_antagonis	AT3G2814	RNA ligase/cyclic	0
245	6123757	1	6.65E-07	Cu_antagonism	AT1G17780	ATG8A/F interacting protein containing a WxxL LIR motif at the C terminus which is essential for interaction with ATG8. Stress (abiotic or biotic)	heat acclimation; (GO:0010286); nucleus; (GO:0005634); phagophore; (GO:0061908); protein binding; (GO:0005515); autophagosome; (
246	3989691	3	6.81E-07	Cu_antagonis	FYD	FYD	chloroplast envelope; (
247	2.1E+07	5	6.85E-07	Cu_antagonism	KEA5	K+ efflux antiporter 5	potassium:proton antiporter activity; (GO:0015386); potassium ion transmembrane transport; (GO:0071805);
248	7052493	3	6.90E-07	Cu_antagonism	DELTA-VPE	delta vacuolar processing enzyme	cysteine-type endopeptidase activity; (GO:0004197); plant-type cell wall; (GO:0009505); vacuolar protein processing; (GO:0006624); protein storage vacuole; (
249	7083996	1	6.98E-07	Cu_antagonis	-	-	-
250	1.2E+07	4	7.03E-07	Cu_antagonism	AT4G22860	TPX2-LIKE Group A family with aurora binding andTPX2 domains. Activator of aurora kinase activity	nucleus; (GO:0005634); nuclear microtubule; (GO:0005880); mitochondrion; (GO:0005739); activation of protein kinase activity; (GO:0032147); protein kinase activator activity; (GO:0030295
251	2.6E+07	5	7.06E-07	Cu_antagonis	CHR24	chromatin	-
252	2.5E+07	5	7.13E-07	Cu_antagonis	-	-	-
253	356583	2	7.13E-07	Cu_antagonis	-	-	-
254	2E+07	1	7.17E-07	Cu_antagonism	AT1G53200	TAF RNA polymerase I subunit A	nucleus; (GO:0005634); biological_process_unknown; (GO:0008150

255	1.5E+07	4	7.63E-07	Cu_antagonism	AT4G31360	selenium binding protein	nucleus； (GO:0005634)； Golgi
256	1.5E+07	4	8.84E-07	Cu_antagonism	AT4G31880	One of 5 PO76/PDS5 cohesion cofactor orthologs of Arabidopsis	chromatin； (GO:0000785)； nucleus； (GO:0005634)； mitotic sister chromatid
257	4453610	3	8.86E-07	Cu_antagonis	-	-	-
258	2.5E+07	5	9.02E-07	Cu_antagonism	AT5G63640	ENTH/VHS/GAT family protein	nucleus； (GO:0005634)； plasma membrane； (
259	2.5E+07	5	9.02E-07	Cu_antagonism	AT5G63640	ENTH/VHS/GAT family protein	nucleus； (GO:0005634)； plasma membrane； (
260	6111687	1	9.35E-07	Cu_antagonism	CSTF77	Tetratricopeptide repeat (TPR)-like superfamily protein	gene silencing by RNA； (GO:0031047)； protein binding； (GO:0005515)； nucleus； (GO:0005634)； mRNA binding； (GO:0003729)； regulation of gene
261	1.1E+07	3	9.50E-07	Cu_antagonis	-	-	-
262	1E+07	5	9.56E-07	Cu_antagonism	DES1	L-cysteine desulfhydrase 1	cytoplasm； (GO:0005737)； chloroplast； (GO:0009507)； L-cysteine desulfhydrase activity； (GO:0080146)； cysteine homeostasis； (GO:0080145)； cystathionine gamma-
263	3704802	5	9.56E-07	Cu_antagonis	GuILO3	-	-
264	944214	1	9.97E-07	Cu_antagonis	-	-	-
265	1.8E+07	5	1.02E-06	Cu_antagonism	AT5G43950	vacuolar protein sorting-associated protein	cellular_component_unknown； (GO:0005575)；
266	4345705	4	1.02E-06	Cu_antagonism	AT4G07524	Ras-related small GTP-binding	biological_process_unknown； (GO:0008150
267	2.7E+07	1	1.03E-06	Cu_antagonis	-	-	-
268	4846040	5	1.04E-06	Cu_antagonis	DEL2	DP-E2F-like 2	-
269	6131358	1	1.05E-06	Cu_antagonis	BETA-TIP	beta-tonoplast	-
270	8685294	5	1.07E-06	Cu_antagonis	-	-	-

271	6985904	5	1.11E-06	Cu_antagonism	COPT5	copper transporter 5	copper ion transmembrane transport；(GO:0035434)；response to abscisic acid；(GO:0009737)；high-affinity copper ion transmembrane transporter activity；(GO:0015089)；copper ion transmembrane transporter activity；(
272	6986306	5	1.11E-06	Cu_antagonism	AT5G20660	Zn-dependent exopeptidases superfamily	endoplasmic reticulum；(GO:0005783)；
273	6990330	5	1.11E-06	Cu_antagonism	AT5G20660	Zn-dependent exopeptidases superfamily	endoplasmic reticulum；(GO:0005783)；
274	6991854	5	1.11E-06	Cu_antagonis	-	-	-
275	6995926	5	1.11E-06	Cu_antagonis	-	-	-
276	1.9E+07	2	1.15E-06	Cu_antagonis	AT2G4748	hypothetical	mitochondrion；(
277	5137011	4	1.15E-06	Cu_antagonis	-	-	-
278	2E+07	1	1.18E-06	Cu_antagonis	AT1G5320	-	-
279	6898572	5	1.20E-06	Cu_antagonism	MGD2	monogalactosyldiacylglycerol synthase 2	UDP-galactosyltransferase activity；(GO:0035250)；1,2-diacylglycerol 3-beta-galactosyltransferase activity；(GO:0046509)；glycolipid biosynthetic process；(GO:0009247)；fatty

280	6929612	5	1.20E-06	Cu_antagonism	XIK	myosin family protein with Dil	actin cytoskeleton; (GO:0015629); mitochondrion localization; (GO:0051646); motor activity; (GO:0003774); trichome morphogenesis; (GO:0010090); plasmodesma; (GO:0009506); transport vesicle; (GO:0030133); root hair elongation; (GO:0048767); vesicle transport along actin filament; (GO:0030050); cell division; (GO:0051301); actin filament organization; (GO:0007015); actin
281	6955406	5	1.20E-06	Cu_antagonism	AT5G20560	Glycosyl hydrolase superfamily protein	anchored component of plasma membrane; (GO:0046658); extracellular region; (GO:0005576); glucan
282	6965654	5	1.20E-06	Cu_antagonism	TBL5	TRICHOME BIREFRINGENCE-LIKE 5	chloroplast; (GO:0009507); Golgi apparatus; (GO:0005794); O-
283	7040060	5	1.20E-06	Cu_antagonism	AT5G20790	transmembrane protein	extracellular region; (GO:0005576); molecular function un
284	2.6E+07	5	1.21E-06	Cu_antagonis	-	-	-
285	6162427	1	1.22E-06	Cu_antagonism	HDG12	homeodomain GLABROUS 12	DNA-binding transcription factor activity, RNA polymerase II-specific; (GO:0000981); DNA-binding transcription
286	1.3E+07	5	1.27E-06	Cu_antagonism	AT5G35205	ransposable_element_gene;(source:Araport11);non-LTR	-

287	6050051	4	1.30E-06	Cu_antagonism	CPK4	calcium-dependent protein kinase 4	protein binding； (GO:0005515)； calmodulin-dependent protein kinase activity； (GO:0004683)； cytoplasm； (GO:0005737)； calcium-dependent protein serine/threonine kinase activity； (GO:0009931)； peptidyl-serine phosphorylation； (GO:0018105)； positive regulation of abscisic acid-activated
288	1.3E+07	1	1.34E-06	Cu_antagonis	-	-	-
289	2.5E+07	5	1.35E-06	Cu_antagonism	FLS2	Leucine-rich receptor-like protein kinase family protein	defense response to bacterium； (GO:0042742)； protein binding； (GO:0005515)； membrane； (GO:0016020)； identical protein binding； (GO:0042802)； transmembrane receptor protein serine/threonine kinase activity； (GO:0004675)； plasma
290	2.6E+07	5	1.35E-06	Cu_antagonism	AT5G64160	plant/protein	molecular_function_unknown； (GO:0003674)；
291	6114793	1	1.35E-06	Cu_antagonism	CSTF77	Tetratricopeptide repeat (TPR)-like superfamily protein	gene silencing by RNA； (GO:0031047)； protein binding； (GO:0005515)； nucleus； (GO:0005634)； mRNA binding； (GO:0003729)； regulation of gene
292	6612615	5	1.36E-06	Cu_antagonis	-	-	-
293	6785478	5	1.36E-06	Cu_antagonism	AT5G20080	FAD/NAD(P)-binding oxidoreductase	plant-type cell wall； (GO:0009505)； mitochondrion； (GO:0005739)； cytochrome-b5 reductase activity, acting on NAD(P)H； (
294	2.6E+07	5	1.37E-06	Cu_antagonism	MC3	metacaspase 3	cysteine-type endopeptidase
295	1.6E+07	1	1.38E-06	Cu_antagonism	AT1G42515	ransposable_element_gene;(source:Ar	-

296	6168473	1	1.42E-06	Cu_antagonism	AT1G17930	Mobile domain protein involved in silencing of	nucleus; (GO:0005634); meristem
297	6618416	5	1.42E-06	Cu_antagonism	GNL2	Ubiquitous expression in testis	-
298	3284053	5	1.46E-06	Cu_antagonism	GRF6	G-box regulating factor 6	nucleus; (GO:0005634); transcription, DNA-templated; (GO:0006351); protein binding; (
299	1.6E+07	4	1.57E-06	Cu_antagonism	AT4G32285	Putative clathrin assembly protein, component of TPLATE complex that functions in clathrin-mediated endocytosis	1-phosphatidylinositol binding; (GO:0005545); clathrin-coated pit; (GO:0005905); SNARE binding; (GO:0000149); plasma membrane; (GO:0005886); clathrin heavy chain binding; (
300	1.5E+07	4	1.60E-06	Cu_antagonism	AT4G31880	One of 5 PO76/PDS5 cohesion cofactor orthologs of Arabidopsis	chromatin; (GO:0000785); nucleus; (GO:0005634); mitotic sister chromatid
301	4845899	5	1.66E-06	Cu_antagonis	DEL2	DP-E2F-like 2	-
302	1.6E+07	1	1.66E-06	Cu_antagonism	PMI1	plastid movement impaired1	intracellular anatomical structure; (GO:0005622); response to osmotic stress; (GO:0006970); regulation of seed germination; (GO:0010029); cytosol; (GO:0005829); response to blue light; (GO:0009637); nucleus; (GO:0005634);
303	6456342	5	1.67E-06	Cu_antagonis	-	-	-
304	1982674	3	1.72E-06	Cu_antagonism	AT3G06455	ubiquitin family protein	nucleus; (GO:0005634);
305	3577735	5	1.73E-06	Cu_antagonism	AT5G11220	hypothetical protein	biological_process_unknown; (GO:0008150

306	7053611	5	1.73E-06	Cu_antagonism	SUS1	sucrose synthase 1	nucleus; (GO:0005634); production of siRNA involved in RNA interference; (GO:0030422); regulation of seed maturation; (GO:2000034); ribonuclease III activity; (GO:0004525); protein binding; (GO:0005515); RNA processing; (GO:0006396); mRNA cleavage involved in gene silencing by miRNA; (GO:0035279); nuclear dicing body; (GO:0010445); production of lsiRNA involved in RNA interference; (
307	1.5E+07	4	1.78E-06	Cu_antagonism	AT4G31880	One of 5 PO76/PDS5 cohesion cofactor orthologs of Arabidopsis	chromatin; (GO:0000785); nucleus; (GO:0005634); mitotic sister chromatid
308	3689078	5	1.79E-06	Cu_antagonis	-	-	-
309	2E+07	1	1.82E-06	Cu_antagonis	-	-	-
310	2E+07	1	1.83E-06	Cu_antagonism	AT1G52990	thioredoxin family protein	cell redox homeostasis; (GO:0045454); protein-disulfide reductase activity; (GO:0047134); oxidoreductase activity, acting on a sulfur group of donors, disulfide as
311	3869355	3	1.87E-06	Cu_antagonism	EML1	Human echinoderm	-
312	6122693	1	1.90E-06	Cu_antagonism	SUVH7	histone-lysine N-methyltransferase, H3 lysine-9 specific SUVH3-	histone lysine methylation; (GO:0034968); zinc ion binding; (
313	2E+07	1	1.91E-06	Cu_antagonism	AT1G54920	hypothetical protein	molecular_function_unknown; (GO:0003674);
314	2.7E+07	1	1.93E-06	Cu_antagonis	-	-	-

315	1.9E+07	5	1.94E-06	Cu_antagonism	AT5G47690	One of 5 PO76/PDS5 cohesion cofactor orthologs of Arabidopsis	DNA repair; (GO:0006281); cytosol; (GO:0005829); mitotic sister chromatid cohesion; (GO:0007064); plastid; (GO:0009536
316	1.2E+07	2	1.94E-06	Cu_antagonism	AT2G28660	Chloroplast-targeted copper	nucleus; (GO:0005634); metal
317	1.6E+07	3	1.94E-06	Cu_antagonism	DCL3	dicer-like 3	ribonuclease III activity; (GO:0004525); protein binding; (GO:0005515); nucleolus; (GO:0005730); RNA binding; (GO:0003723); nucleus; (GO:0005634); maintenance of DNA methylation; (GO:0010216); nucleic
318	1.5E+07	4	2.01E-06	Cu_antagonism	AT4G30990	ARM repeat superfamily protein	Golgi apparatus; (GO:0005794); nucleolus; (GO:0005730); biological_process unk
319	6810909	5	2.03E-06	Cu_antagonism	AT5G20170	RNA polymerase II transcription mediator	mediator complex; (GO:0016592); transcription coregulator activity; (GO:0003712); core mediator complex; (GO:0070847);
320	3498140	5	2.07E-06	Cu_antagonis	TRS120	TRS120	-
321	2E+07	1	2.11E-06	Cu_antagonis	AT1G5301	RING/U-box	protein ubiquitination;
322	2E+07	1	2.27E-06	Cu_antagonism	AT1G53025	Ubiquitin-conjugating enzyme family protein	nucleus; (GO:0005634); postreplication repair; (GO:0006301); protein K63-linked
323	7968454	3	2.35E-06	Cu_antagonism	PDF2	protodermal factor 2	cytoplasm; (GO:0005737); protein folding; (GO:0006457); prefoldin complex;

324	5033389	1	2.37E-06	Cu_antagonism	NHX8	Na+/H+ exchanger 8	lithium ion transport; (GO:0010351); plasma membrane; (GO:0005886); solute:proton antiporter activity; (GO:0015299); potassium ion transmembrane transport; (GO:0071805); potassium:proton antiporter activity; (
325	1.6E+07	1	2.37E-06	Cu_antagonis	-	-	-
326	3614061	5	2.38E-06	Cu_antagonis	-	-	-
327	1.5E+07	4	2.39E-06	Cu_antagonism	AT4G31360	selenium binding protein	nucleus; (GO:0005634); Golgi
328	6107227	1	2.40E-06	Cu_antagonism	PEPR2	PEP1 receptor 2	protein serine kinase activity; (GO:0106310); peptide binding; (GO:0042277); kinase activity; (GO:0016301); protein binding; (GO:0005515); plasma membrane; (GO:0005886);
329	1785724	3	2.42E-06	Cu_antagonis	-	-	-
330	3575247	5	2.43E-06	Cu_antagonis	-	-	-
331	1.5E+07	4	2.47E-06	Cu_antagonis	UBP18	ubiquitin-specific	-
332	1.3E+07	4	2.47E-06	Cu_antagonism	AT4G25610	C2H2-like zinc finger protein	biological_process_unknown; (GO:0008150); nucleus; (
333	1.4E+07	5	2.48E-06	Cu_antagonis	-	-	-
334	6571559	5	2.50E-06	Cu_antagonism	AT5G19480	mediator of RNA polymerase II transcription subunit	transcription factor binding; (GO:0008134); mediator complex; (GO:0016592); nucleus; (
335	6572245	5	2.50E-06	Cu_antagonism	AT5G19480	mediator of RNA polymerase II transcription subunit	transcription factor binding; (GO:0008134); mediator complex; (GO:0016592); nucleus; (
336	6572576	5	2.50E-06	Cu_antagonism	AT5G19480	mediator of RNA polymerase II transcription subunit	transcription factor binding; (GO:0008134); mediator complex; (GO:0016592); nucleus; (
337	6572726	5	2.50E-06	Cu_antagonism	AT5G19480	mediator of RNA polymerase II transcription subunit	transcription factor binding; (GO:0008134); mediator complex; (GO:0016592); nucleus; (

338	6573441	5	2.50E-06	Cu_antagonism	AT5G19480	mediator of RNA polymerase II transcription subunit	transcription factor binding; (GO:0008134); mediator complex; (GO:0016592);
339	6586217	5	2.50E-06	Cu_antagonis	MSL9	mechanosensitive	-
340	9671970	3	2.53E-06	Cu_antagonism	ATRZ-1A	-	nucleus; (nucleus; (GO:0005634); zinc ion binding; (GO:0008270); cold acclimation; (GO:0009631); mRNA
341	6172451	1	2.55E-06	Cu_antagonism	AT1G17940	Endosomal targeting BRO1-like domain-	nucleus; (GO:0005634); mitochondrion; (
342	2.5E+07	5	2.58E-06	Cu_antagonism	emb2746	RNA-metabolising metallo-beta-lactamase family protein	mRNA binding; (GO:0003729); nucleus; (GO:0005634); chloroplast organization; (GO:0009658); embryo development ending in seed
343	3548045	5	2.60E-06	Cu_antagonis	VAMP713	vesicle-associated	-
344	6955554	5	2.70E-06	Cu_antagonism	AT5G20560	Glycosyl hydrolase superfamily protein	anchored component of plasma membrane; (GO:0046658); extracellular region; (GO:0005576); glucan
345	9852873	5	2.88E-06	Cu_antagonis	-	-	-
346	9863967	5	2.88E-06	Cu_antagonism	TOPP8	Calcineurin-like metallo-phosphoesterase superfamily protein	cytoplasm; (GO:0005737); protein dephosphorylation; (GO:0006470); root hair cell tip growth; (GO:0048768); nucleus; (GO:0005634); protein serine phosphatase
347	2E+07	1	2.89E-06	Cu_antagonis	-	-	-
348	6108407	1	2.91E-06	Cu_antagonism	PEPR2	PEP1 receptor 2	protein serine kinase activity; (GO:0106310); peptide binding; (GO:0042277); kinase activity; (GO:0016301); protein binding; (GO:0005515); plasma membrane; (GO:0005886);

349	6108654	1	2.91E-06	Cu_antagonism	PEPR2	PEP1 receptor 2	[illegible] activity; (GO:0106310); peptide binding; (GO:0042277); kinase activity; (GO:0016301); protein binding; (GO:0005515); plasma membrane; ([illegible]
350	6116096	1	2.91E-06	Cu_antagonism	CSTF77	Tetratricopeptide repeat (TPR)-like superfamily protein	[illegible] RNA; (GO:0031047); protein binding; (GO:0005515); nucleus; (GO:0005634); mRNA binding; (GO:0003729); [illegible]
351	2E+07	1	2.95E-06	Cu_antagonis	-	-	-
352	6319036	3	2.97E-06	Cu_antagonis	NAC058	NAC domain	-
353	6134713	1	3.00E-06	Cu_antagonis	AT1G0533	Natural antisense	-
354	1.5E+07	4	3.06E-06	Cu_antagonis	-	-	-
355	6990737	5	3.06E-06	Cu_antagonism	AT5G20660	Zn-dependent exopeptidases superfamily	endoplasmic reticulum; (GO:0005783);
356	4886932	4	3.09E-06	Cu_antagonis	-	-	-
357	6966349	5	3.24E-06	Cu_antagonis	-	-	-
358	6971527	5	3.24E-06	Cu_antagonism	AT5G20610	Encodes a member of a plant specific C2 domain containing gene family. Along with PMI, it appears to be involved in [illegible]	intracellular anatomical structure; (GO:0005622); nucleus; (GO:0005634); chloroplast relocation; (GO:0009902); [illegible]
359	1.5E+07	4	3.29E-06	Cu_antagonism	AT4G30990	ARM repeat superfamily protein	Golgi apparatus; (GO:0005794); nucleolus; (GO:0005730); [illegible]
360	6121342	1	3.34E-06	Cu_antagonism	SUVH7	histone-lysine N-methyltransferase, H3 lysine-9 specific SUVH3-	histone lysine methylation; (GO:0034968); zinc ion binding; (
361	1.5E+07	4	3.39E-06	Cu_antagonism	AT4G31180	The IBI1 gene encodes an aspartyl tRNA synthetase (AspRS). In addition, the IBI1 protein acts as a receptor protein of [illegible]	[illegible]); RNA binding; (GO:0003723); aspartate-tRNA ligase activity; (GO:0004815); defense response to fungus; (GO:0050832); response to cadmium [illegible]

362	805706	2	3.41E-06	Cu_antagonism	MYB88	myb domain protein 88	nucleus; (GO:0005634); sequence-specific DNA binding; (GO:0043565); stomatal complex formation; (GO:0010376); RNA polymerase II cis-regulatory region sequence-specific DNA binding; (GO:0000978); guard mother cell differentiation; (GO:0010444); regulation of DNA endoreduplication; (GO:0032875); response to abscisic acid; (GO:0009737); lateral root development; (GO:0048527); DNA-binding transcription
363	2E+07	1	3.46E-06	Cu_antagonism	ATMAP4K ALPHA1	Protein kinase superfamily	-
364	2E+07	1	3.46E-06	Cu_antagonism	ATMAP4K ALPHA1	Protein kinase superfamily	-
365	1.3E+07	4	3.50E-06	Cu_antagonis	SLK3	SEUSS-like 3	-
366	1.3E+07	4	3.50E-06	Cu_antagonism	AT4G25550	Cleavage/polyadenylation specificity factor, 25kDa subunit	hydrolase activity; (GO:0016787); mRNA processing; (GO:0006397); rRNA processing; (GO:0006364); nucleus; (GO:0005634); mRNA binding; (
367	1.3E+07	4	3.50E-06	Cu_antagonism	AT4G25580	CAP160 protein	response to stress; (GO:0006950); response to abscisic
368	1.4E+07	5	3.61E-06	Cu_antagonis	-	-	-
369	6110680	1	3.67E-06	Cu_antagonism	CSTF77	Tetratricopeptide repeat (TPR)-like superfamily protein	gene silencing by RNA; (GO:0031047); protein binding; (GO:0005515); nucleus; (GO:0005634); mRNA binding; (GO:0003729); regulation of gene
370	6992629	5	3.71E-06	Cu_antagonis	-	-	-
371	3704279	5	3.71E-06	Cu_antagonis	GuILO3	-	-

372	2.6E+07	5	3.72E-06	Cu_antagonism	PI-4KBETA1	phosphatidylinositol 4-OH kinase beta1	plasma membrane; (GO:0005886); root hair tip; (GO:0035619); root hair cell tip growth; (GO:0048768); pollen tube growth; (GO:0009860); phosphatidylinositol phosphorylation; (GO:0046854); protein binding; (GO:0005515); cytoplasmic vesicle membrane; (GO:0030659);
373	1.1E+07	5	3.74E-06	Cu_antagonism	AT5G28776	transposable_element_gene;(source: Araport11);copia-like	-
374	9940571	5	3.76E-06	Cu_antagonis	-	-	-
375	1.7E+07	5	3.78E-06	Cu_antagonism	GLA1	Folylpolyglutamate synthetase family protein	tetrahydrofolylpolyglutamate synthase activity; (GO:0004326); mitochondrion; (GO:0005739); cytosol; (GO:0005829); one-carbon metabolic process; (GO:0006730); dihydrofolate synthase
376	5108439	4	3.89E-06	Cu_antagonis	-	-	-
377	2.7E+07	1	3.93E-06	Cu_antagonism	AT1G72210	basic helix-loop-helix (bHLH) DNA-binding superfamily protein	protein binding; (GO:0005515); regulation of transcription by RNA polymerase II; (GO:0006357); protein dimerization activity; (GO:0046983); DNA-binding transcription
378	6256850	4	3.93E-06	Cu_antagonis	-	-	-
379	1.1E+07	3	4.04E-06	Cu_antagonis	-	-	-

380	1.6E+07	1	4.06E-06	Cu_antagonism	PMI1	plastid movement impaired1	intracellular anatomical structure; (GO:0005622); response to osmotic stress; (GO:0006970); regulation of seed germination; (GO:0010029); cytosol; (GO:0005829); response to blue light; (GO:0009637); nucleus; (GO:0005634);
381	3769610	5	4.12E-06	Cu_antagonism	AT5G11700	ephrin type-B receptor	plasmodesma; (GO:0009506); extracellular region; (
382	2E+07	2	4.13E-06	Cu_antagonis	SETH6	Phototropic-	-
383	5127092	4	4.13E-06	Cu_antagonis	-	-	-
384	2E+07	1	4.15E-06	Cu_antagonism	TCP3	-	nucleus; (GO:0005634); leaf development; (GO:0048366); response to auxin; (GO:0009733); leaf morphogenesis; (GO:0009965); sequence-specific DNA binding; (GO:0043565); protein
385	1.1E+07	3	4.15E-06	Cu_antagonism	AT3G29175	transposable_element_gene;(source: Araport11);Mutator-like transposase	-
386	3729221	5	4.25E-06	Cu_antagonis	-	-	-
387	6995739	5	4.29E-06	Cu_antagonis	-	-	-
388	9726633	5	4.40E-06	Cu_antagonism	MIRO1	MIRO-related GTP-ase 1	mitochondrion; (GO:0005739); calcium ion binding; (GO:0005509); plastid; (GO:0009536); regulation of mitochondrion organization; (GO:0010821);
389	1344131	5	4.41E-06	Cu_antagonis	-	-	-
390	1.3E+07	2	4.44E-06	Cu_antagonism	SAG13	senescence-associated gene 13	aging; (GO:0007568); chloroplast; (GO:0009507); defense response to insect; (GO:0002213); regulation of defense response; (
391	613022	5	4.47E-06	Cu_antagonism	AT5G02710	zinc/iron-chelating domain protein	molecular_function_unknown; (GO:0003674);

392	3459373	5	4.52E-06	Cu_antagonism	AT5G10950	Tudor/PWWP/MBT superfamily protein	nucleus; (GO:0005634); DNA repair; (GO:0006281); mitotic sister chromatid cohesion; (GO:0007064);
393	6131950	1	4.68E-06	Cu_antagonis	AT1G0533	Natural antisense	-
394	2.7E+07	1	4.74E-06	Cu_antagonis	-		-
395	1.9E+07	2	4.77E-06	Cu_antagonism	UNE5		plant-type cell wall; (GO:0009505); vacuolar membrane; (GO:0005774); response to cadmium ion; (GO:0046686); extracellular region; (GO:0005576); pollen tube development; (GO:0048868); endoplasmic reticulum; (GO:0005783); embryo development
396	3890092	3	4.78E-06	Cu_antagonism	Nek7		cytoplasm; (GO:0005737); protein threonine kinase activity; (GO:0106311); protein kinase activity; (GO:0004672); protein serine/threonine kinase activity; (GO:0004674
397	2E+07	1	4.80E-06	Cu_antagonis	-		-
398	1.4E+07	2	4.87E-06	Cu_antagonism	AT2G34185	hypothetical protein	molecular_function_unknown; (GO:0003674); mitochondrion; (
399	1.4E+07	5	4.95E-06	Cu_antagonis	-		-
400	3483194	5	4.96E-06	Cu_antagonis	-		-
401	6779063	5	4.99E-06	Cu_antagonism	AT5G20060	alpha/beta-Hydrolases	cytoplasm; (GO:0005737);
402	6367261	5	5.02E-06	Cu_antagonis	-		-
403	2.6E+07	5	5.05E-06	Cu_antagonism	AT5G64220	CAMTA2 proteins bind to the AtALMT1 promoter at in vitro. The gene itself is Al inducible, and AtALMT1 expression is partially repressed in camta2 mutant. The mRNA is cell-	transcription coregulator activity; (GO:0003712); nucleus; (GO:0005634); DNA-binding transcription factor activity; (GO:0003700); regulation of transcription, DNA-templated; (GO:0006355);
404	3477147	5	5.27E-06	Cu_antagonism	AT5G10990	SAUR-like auxin-responsive protein family	response to auxin; (GO:0009733); molecular_function_un

405	6572794	5	5.31E-06	Cu_antagonism	AT5G19480	mediator of RNA polymerase II transcription subunit	transcription factor binding; (GO:0008134); mediator complex; (GO:0016592); nucleus; (
406	5215287	4	5.33E-06	Cu_antagonism	AT4G08264	transposable_element_gene;(source:	-
407	140430	4	5.38E-06	Cu_antagonism	AT4G00320	F-box/RNI-like superfamily	biological_process_unknown; (GO:0008150
408	156611	4	5.40E-06	Cu_antagonis	AT4G0035	Encodes a	-
409	1.4E+07	5	5.42E-06	Cu_antagonis	-	-	-
410	6528991	5	5.48E-06	Cu_antagonism	CLT1	CRT (chloroquine-resistance transporter)-like transporter 1	glutathione transport; (GO:0034635); plastid; (GO:0009536); response to cadmium
411	6129263	1	5.52E-06	Cu_antagonis	ENODL22	early nodulin-like	-
412	3345652	5	5.54E-06	Cu_antagonis	-	-	-
413	1.4E+07	5	5.55E-06	Cu_antagonis	-	-	-
414	1.6E+07	4	5.60E-06	Cu_antagonism	AT4G33060	Cyclophilin-like peptidyl-prolyl cis-trans isomerase family protein	peptidyl-prolyl cis-trans isomerase activity; (GO:0003755); protein folding; (GO:0006457); protein peptidyl-
415	667064	5	5.62E-06	Cu_antagonism	UPL4	ubiquitin-protein ligase 4	nucleus; (GO:0005634); ubiquitin-protein transferase activity; (GO:0004842); protein
416	1.5E+07	3	5.62E-06	Cu_antagonis	-	-	-
417	6122042	1	5.64E-06	Cu_antagonism	SUVH7	histone-lysine N-methyltransferase, H3 lysine-9 specific SUVH3-	histone lysine methylation; (GO:0034968); zinc ion binding; (
418	1.3E+07	5	5.66E-06	Cu_antagonism	AT5G34866	transposable_element_gene;(source: Araport11);gypsy-	-
419	4467408	3	5.67E-06	Cu_antagonis	AT3G1366	Disease resistance-	extracellular region; (
420	6645051	5	5.68E-06	Cu_antagonism	S1P	SITE-1 protease	Golgi apparatus; (GO:0005794); extracellular region; (GO:0005576); endoplasmic reticulum; (GO:0005783); proteolysis; (GO:0006508); serine
421	1.4E+07	1	5.69E-06	Cu_antagonis	-	-	-
422	3590592	3	5.70E-06	Cu_antagonis	-	-	-
423	2E+07	2	5.76E-06	Cu_antagonism	AT2G47610	Ribosomal protein L7Ae/L30e/S12e/Gadd45 family protein	cytoplasm; (GO:0005737); cytosolic ribosome; (GO:0022626); maturation of LSU-rRNA; (GO:0000470); nucleolus; (GO:0005730); cytosolic large

424	3728453	5	5.88E-06	Cu_antagonism	TINY2	Integrase-type DNA-binding superfamily protein	nucleus; (GO:0005634); positive regulation of transcription, DNA-templated; (
425	3521242	5	5.90E-06	Cu_antagonis	-	-	-
426	6121816	1	5.93E-06	Cu_antagonism	SUVH7	histone-lysine N-methyltransferase, H3 lysine-9 specific SUVH3-like protein	nucleus; (GO:0005634); positive regulation of transcription, DNA-templated; (
427	3668768	5	5.93E-06	Cu_antagonis	-	-	-
428	6108971	1	5.94E-06	Cu_antagonism	PEPR2	PEP1 receptor 2	protein serine kinase activity; (GO:0106310); peptide binding; (GO:0042277); kinase activity; (GO:0016301); protein binding; (GO:0005515); plasma membrane; (GO:0005886);
429	1E+07	4	5.96E-06	Cu_antagonis	-	-	-
430	3666881	5	5.97E-06	Cu_antagonism	AT5G11470	SG1 is a Bromo-Adjacent Homology (BAH) domain containing protein involved in CHG methylation within genebodies. Loss of function results in	regulation of histone methylation; (GO:0031060); nucleus; (GO:0005634); negative regulation of chromatin silencing; (GO:0031936); chromatin binding; (
431	1.8E+07	2	6.01E-06	Cu_antagonism	AT2G43140	bHLH129 is a nuclear localized basic helix loop helix protein. It has been shown to function as a transcriptional	cellular response to abscisic acid stimulus; (GO:0071215); nucleus; (GO:0005634); negative regulation of gene expression; (
432	6987427	5	6.02E-06	Cu_antagonism	AT5G20660	Zn-dependent exopeptidases superfamily	endoplasmic reticulum; (GO:0005783);
433	1.5E+07	4	6.14E-06	Cu_antagonis	-	-	-
434	6114974	1	6.20E-06	Cu_antagonism	CSTF77	Tetratricopeptide repeat (TPR)-like superfamily protein	gene silencing by RNA; (GO:0031047); protein binding; (GO:0005515); nucleus; (GO:0005634); mRNA binding; (GO:0003729); regulation of gene
435	1.7E+07	3	6.20E-06	Cu_antagonism	AT3G45775	transposable_element_gene;(source: Araport11);copia-like	-

436	2E+07	5	6.22E-06	Cu_antagonism	AT5G48650	Negative regulator of defense response to Pseudomonas syringae pv. tomato through	cytosol; (GO:0005829); cytoplasm; (GO:0005737); ribonucleoprotein complex; (GO:1990904);
437	6109751	1	6.23E-06	Cu_antagonism	PEPR2	PEP1 receptor 2	protein serine kinase activity; (GO:0106310); peptide binding; (GO:0042277); kinase activity; (GO:0016301); protein binding; (GO:0005515); plasma membrane; (GO:0005886);
438	5007555	5	6.34E-06	Cu_antagonis	-	-	-
439	9749437	5	6.36E-06	Cu_antagonis	-	-	-
440	6381285	5	6.45E-06	Cu_antagonis	-	-	-
441	1.4E+07	1	6.48E-06	Cu_antagonism	AT1G36915	transposable_element_gene;(source: Araport11);pseudogene, similar to putative	-
442	3720137	5	6.71E-06	Cu_antagonism	AT5G11580	Regulator of chromosome condensation	cytosol; (GO:0005829); Golgi apparatus; (GO:0005794); plasma
443	3455417	5	6.81E-06	Cu_antagonism	AT5G10946	hypothetical protein	biological_process_unknown; (GO:0008150
444	3455923	5	6.81E-06	Cu_antagonism	AT5G10946	hypothetical protein	biological_process_unknown; (GO:0008150
445	2.6E+07	5	6.88E-06	Cu_antagonism	TOP3A	This gene encodes a DNA topoisomerase, an enzyme that controls and alters the topologic states of DNA during transcription	chromosome; (GO:0005694); nucleus; (GO:0005634); DNA repair; (GO:0006281); meiotic cell cycle; (GO:0051321); DNA topoisomerase activity; (GO:0003916); DNA topological
446	3764090	5	6.88E-06	Cu_antagonism	AT5G11700	ephrin type-B receptor	plasmodesma; (GO:0009506); extracellular region; (
447	3543061	5	7.01E-06	Cu_antagonism	AT5G11130	Exostosin family protein	transferase activity, transferring glycosyl groups; (GO:0016757
448	1.4E+07	5	7.04E-06	Cu_antagonism	AT5G35380	kinase with adenine nucleotide alpha hydrolases-like domain-containing protein	kinase activity; (GO:0016301); phosphorylation; (GO:0016310); protein phosphorylation; (GO:0006468); ATP
449	3787697	5	7.05E-06	Cu_antagonism	AT5G11750	Ribosomal protein L19 family protein	chloroplast; (GO:0009507); structural constituent of

450	1.3E+07	4	7.12E-06	Cu_antagonism	ABCB28	member of NAP subfamily	chloroplast；（ GO:0009507)；integral component of membrane；（ GO:0016021)；transmembrane transport；（ GO:0055085)；
451	1.3E+07	4	7.12E-06	Cu_antagonism	ABCB28	member of NAP subfamily	chloroplast；（ GO:0009507)；integral component of membrane；（ GO:0016021)；transmembrane transport；（ GO:0055085)；
452	4859052	1	7.19E-06	Cu_antagonism	AT1G14220	Ribonuclease T2 family protein	extracellular region；（ GO:0005576)；endoribonuclease activity；（ GO:0004521)；RNA catabolic
453	3744415	5	7.30E-06	Cu_antagonism	-	-	-
454	3502475	5	7.34E-06	Cu_antagonism	MYB64	myb domain	-
455	122676	4	7.36E-06	Cu_antagonism	AT4G00280	ER protein carbohydrate-	molecular_function_unknown；（ GO:0003674
456	2.1E+07	5	7.44E-06	Cu_antagonism	RGP4	reversibly glycosylated polypeptide 4	plant-type cell wall biogenesis；（ GO:0009832)；Golgi apparatus；（ GO:0005794)；protein binding；（
457	2E+07	1	7.56E-06	Cu_antagonism	FRS6	FAR1-related sequence 6	nucleus；（ GO:0005634)；regulation of
458	2E+07	2	7.60E-06	Cu_antagonism	AT2G48000	Pentatricopeptide repeat (PPR) superfamily	biological_process_unknown；（ GO:0008150)；
459	1.9E+07	5	7.64E-06	Cu_antagonism	AT5G47700	Co-orthologous gene of large ribosomal subunit protein RPP1	cytoplasmic translation；（ GO:0002181)；translational elongation；（ GO:0006414)；nucleus；（ GO:0005634)；cytosolic ribosome；（ GO:0022626)；ribonucleoprotein

460	2E+07	1	7.68E-06	Cu_antagonism	Mmdh1	Lactate/malate dehydrogenase family protein	L-malate dehydrogenase activity; (GO:0030060); malate metabolic process; (GO:0006108); carbohydrate metabolic process; (GO:0005975); response to cadmium ion; (GO:0046686); mitochondrion; (GO:0005739);
461	2.1E+07	5	7.75E-06	Cu_antagonis	NF-YC6	nuclear factor Y,	-
462	9328704	1	7.80E-06	Cu_antagonis	-	-	-
463	6128210	1	7.82E-06	Cu_antagonis	-	-	-
464	2E+07	1	8.02E-06	Cu_antagonism	AT1G53330	encodes a member of the pentatricopeptide repeat (PPR) gene family. T-DNA	root development; (GO:0048364); chloroplast; (GO:0009507); embryo development
465	2.1E+07	5	8.03E-06	Cu_antagonis	AT5G5042	Encodes a	extracellular region; (
466	3774465	5	8.03E-06	Cu_antagonism	AT5G11710	EPSIN1 plays an important role in the vacuolar trafficking of soluble proteins at the trans-Golgi network via its interaction with gamma-ADR, VTI11, VSR1, and clathrin. Associated with actin filaments and	clathrin vesicle coat; (GO:0030125); plasma membrane; (GO:0005886); thylakoid; (GO:0009579); endosome; (GO:0005768); protein targeting to vacuole; (GO:0006623); protein binding; (GO:0005515); endocytosis; (
467	6996618	5	8.10E-06	Cu_antagonis	-	-	-
468	1.4E+07	1	8.15E-06	Cu_antagonis	AT1G0922	novel transcribed	-
469	1.4E+07	1	8.15E-06	Cu_antagonis	-	-	-
470	1.8E+07	2	8.15E-06	Cu_antagonism	AT2G44830	AGCVIII kinase involved in the pulse-induced first positive phototropism. Plasma-membrane-associated element of a molecular	cytoplasm; (GO:0005737); plasma membrane; (GO:0005886); phototropism; (GO:0009638); sieve cell differentiation; (GO:0048756); auxin
471	1.3E+07	5	8.27E-06	Cu_antagonis	-	-	-
472	9891509	3	8.35E-06	Cu_antagonis	-	-	-
473	6400482	5	8.36E-06	Cu_antagonis	-	-	-
474	9880110	5	8.46E-06	Cu_antagonism	AT5G27870	Plant invertase/pectin methylesterase inhibitor superfamily	pectinesterase inhibitor activity; (GO:0046910); pectinesterase activity; (GO:0030599); nucleus; (
475	9950039	5	8.48E-06	Cu_antagonism	AT5G55896	transposable_element_gene;(source: Araport11);non-LTR	-

476	1.4E+07	1	8.83E-06	Cu_antagonis	-		-
477	2064891	3	8.88E-06	Cu_antagonism	AT3G06620	PAS domain-containing protein tyrosine kinase family protein	protein phosphorylation； (GO:0006468)； cytoplasm； (GO:0005737)； ATP binding； (
478	1.3E+07	4	8.94E-06	Cu_antagonism	AT4G25250	PMEI4 pectin methyleseterase inhibitor. Expressed in roots	pectinesterase inhibitor activity； (GO:0046910)； negative regulation of catalytic activity； (GO:0043086)； regulation of root
479	6844133	5	9.06E-06	Cu_antagonis	-	-	-
480	6860063	5	9.06E-06	Cu_antagonis	-	-	-
481	3741193	5	9.13E-06	Cu_antagonis	NOXY2	hypothetical	-
482	6118371	1	9.21E-06	Cu_antagonis	-	-	-
483	6122117	1	9.21E-06	Cu_antagonism	SUVH7	histone-lysine N-methyltransferase, H3 lysine-9 specific SUVH3-like protein	nucleus； (GO:0005634)； positive regulation of transcription, DNA-templated； (
484	384888	4	9.27E-06	Cu_antagonism	ECA2	ER-type Ca2+-ATPase 2	calcium transmembrane transporter activity, phosphorylative mechanism； (GO:0005388)； proton-exporting ATPase activity, phosphorylative mechanism； (
485	6124241	1	9.28E-06	Cu_antagonism	AT1G17780	ATG8A/F interacting protein containing a WxxL LIR motif at the C terminus which is essential for interaction with ATG8. Stress (abiotic or biotic)	heat acclimation； (GO:0010286)； nucleus； (GO:0005634)； phagophore； (GO:0061908)； protein binding； (GO:0005515)； autophagosome； (
486	1.2E+07	4	9.28E-06	Cu_antagonis	-	-	-
487	112272	4	9.30E-06	Cu_antagonis	-	-	-
488	2E+07	1	9.37E-06	Cu_antagonism	AT1G54920	hypothetical protein	molecular_function_unknown； (GO:0003674)；
489	6765501	5	9.44E-06	Cu_antagonism	AT5G20030	Plant Tudor-like RNA-binding	biological_process_unknown； (GO:0008150
490	2.1E+07	5	9.50E-06	Cu_antagonism	AT5G50350	hypothetical protein	nucleus； (GO:0005634)； molecular_function_un
491	2E+07	1	9.56E-06	Cu_antagonis	-	-	-
492	2.5E+07	5	9.64E-06	Cu_antagonism	AT5G60470	C2H2 and C2HC zinc fingers superfamily protein	nucleus； (GO:0005634)； DNA-binding transcription factor activity； (

493	1.5E+07	3	9.65E-06	Cu_antagonism	AT3G43151	transposable_element_gene;(source: Araport11);gypsy-like retrotransposon	-
494	3890072	3	9.69E-06	Cu_antagonism	Nek7	NIMA-related kinases share high amino acid sequence identity with the gene product of the Aspergillus nidulans 'never in mitosis A' gene	cytoplasm; (GO:0005737); protein threonine kinase activity; (GO:0106311); protein kinase activity; (GO:0004672); protein serine/threonine kinase activity; (GO:0004674
495	915673	5	9.80E-06	Cu_antagonism	AT5G03610	GDSL-motif esterase/acyltransferase/lipase.	hydrolase activity, acting on ester bonds; (GO:0016788);
496	2E+07	1	9.87E-06	Cu_antagonism	AT1G53860	Encodes a protein that is highly methylated in a	biological_process_unknown; (GO:0008150);
10	7746139	1	1.83E-08	Cu_mutualis	-	-	-
22	7747171	1	2.09E-09	Cu_mutualis	-	-	-
23	7749852	1	2.09E-09	Cu_mutualism	AT1G22010	hypothetical protein	molecular_function_unknown; (GO:0003674);
46	2866886	4	4.14E-06	Cu_mutualis	-	-	-
497	8900611	2	4.33E-09	Cu_mutualism	AT2G20635	protein kinase and Mad3-BUB1-I domain-containing protein	protein serine kinase activity; (GO:0106310); protein phosphorylation; (GO:0006468); protein threonine kinase activity; (GO:0106311); protein kinase activity; (GO:0004672
498	3943227	1	9.80E-09	Cu_mutualis	AT1G0486	None	-
499	1783257	4	1.02E-07	Cu_mutualism	AT4G03824	transposable_element_gene;(source: Araport11);Mariner-like transposase	-
500	4382479	1	4.33E-07	Cu_mutualism	AT1G12855	F-box family protein	nucleus; (GO:0005634); DNA-binding transcription factor activity; (GO:0003700);
501	3947434	1	4.33E-07	Cu_mutualis	-	-	-
502	3941461	1	4.33E-07	Cu_mutualism	AT1G11684	hypothetical protein	cellular_component_unknown; (GO:0005575);
503	9551569	4	5.97E-07	Cu_mutualism	AT4G16970	Protein kinase superfamily protein;(source:Araport11)	protein serine/threonine kinase activity; (GO:0004674); protein autophosphorylation; (GO:0046777); double-strand break repair via break-induced
504	464186	2	6.44E-07	Cu_mutualis	-	-	-

505	2903965	4	9.79E-07	Cu_mutualism	AT4G05594	transposable_element_gene;(source: Araport11);copia-like	-
506	2.8E+07	1	1.75E-06	Cu_mutualis	-	-	-
507	3946688	1	1.79E-06	Cu_mutualism	AT1G11700	senescence regulator (Protein	molecular_function_unknown; (GO:0003674
508	4404315	1	1.79E-06	Cu_mutualism	AT1G12930	Ran effector.	cytoplasm; (GO:0005737); protein
509	1.5E+07	4	2.03E-06	Cu_mutualism	AT4G31210	DNA topoisomerase, type IA, core	chromosome; (GO:0005694); DNA topoisomerase type I (single strand cut, ATP-independent) activity; (GO:0003917);
510	3309721	1	2.07E-06	Cu_mutualis	-	-	-
511	1140210	5	2.07E-06	Cu_mutualis	-	-	-
512	3314951	1	2.29E-06	Cu_mutualism	ECA3	endoplasmic reticulum-type calcium-transporting ATPase 3	calcium transmembrane transporter activity, phosphorylative mechanism; (GO:0005388); calcium ion transmembrane transport; (GO:0070588); Golgi apparatus; (GO:0005794); manganese ion homeostasis; (GO:0055071); calcium ion transport; (GO:0006816);
513	1.9E+07	1	2.31E-06	Cu_mutualism	VIIIA	P-loop containing nucleoside triphosphate hydrolases superfamily protein	microfilament motor activity; (GO:0000146); vesicle; (GO:0031982); motor activity; (GO:0003774); actin cytoskeleton; (GO:0015629); plasma membrane; (GO:0005886); actin filament-based
514	1.7E+07	4	2.51E-06	Cu_mutualism	MLH3	This gene is a member of the MutL-homolog (MLH) family of DNA mismatch repair (MMR) genes. MLH genes	nucleus; (GO:0005634); mismatch repair complex; (GO:0032300); reciprocal meiotic recombination; (

515	1.7E+07	4	2.51E-06	Cu_mutualism	MLH3	This gene is a member of the MutL-homolog (MLH) family of DNA mismatch repair (MMR) genes. MLH genes	nucleus; (GO:0005634); mismatch repair complex; (GO:0032300); reciprocal meiotic recombination; (
516	2.2E+07	5	3.10E-06	Cu_mutualism	AT5G53090	NAD(P)-binding Rossmann-fold	oxidation-reduction process; (GO:0055114
517	1783331	4	3.27E-06	Cu_mutualism	AT4G03824	transposable_element_gene;(source:Araport11);Mariner-like transposase	-
518	2.1E+07	5	3.27E-06	Cu_mutualism	ABCG8	The protein encoded by this gene is a member of the superfamily of ATP-binding	-
519	2.5E+07	5	3.99E-06	Cu_mutualism	ABCA12	The membrane-associated protein	-
520	1640779	4	4.08E-06	Cu_mutualis	-	-	-
521	3386231	3	4.11E-06	Cu_mutualism	AT3G10815	RING/U-box superfamily protein;(source:Ar	ubiquitin protein ligase activity; (GO:0061630); mitochondrion; (
522	1652367	4	4.14E-06	Cu_mutualism	AT4G03728	hypothetical protein;(source:Araport11)	molecular_function_unknown; (GO:0003674);
523	2817901	4	4.14E-06	Cu_mutualism	IBR1	indole-3-butyric acid response 1	peroxisome; (GO:0005777); indolebutyric acid metabolic process; (GO:0080024); chloroplast; (
524	2803877	4	4.15E-06	Cu_mutualis	-	-	-
525	1.8E+07	5	4.84E-06	Cu_mutualism	YUC5	Flavin-binding monooxygenase family protein	NADP binding; (GO:0050661); flavin adenine dinucleotide binding; (GO:0050660); monooxygenase activity; (GO:0004497); auxin biosynthetic process; (GO:0009851
526	1E+07	2	4.84E-06	Cu_mutualis	AT2G2459	hypothetical	-
527	8034073	4	5.00E-06	Cu_mutualis	-	-	-
528	1.6E+07	4	5.46E-06	Cu_mutualis	-	-	-
529	4394432	1	5.98E-06	Cu_mutualis	GAPA-2	glyceraldehyde 3-	-
530	4405073	1	5.98E-06	Cu_mutualism	AT1G12930	Ran effector.	cytoplasm; (GO:0005737); protein
531	557339	5	5.98E-06	Cu_mutualis	-	-	-
532	1.8E+07	5	6.21E-06	Cu_mutualism	AT5G44010	fanconi anemia group F protein (FANCF);(source:Araport11)	cellular response to DNA damage stimulus; (GO:0006974); Fanconi anaemia nuclear complex; (
533	7747197	1	6.69E-06	Cu_mutualis	-	-	-

534	114805	5	6.90E-06	Cu_mutualism	AT5G01280	Encodes a microtubule-associated protein.	plasma membrane; (GO:0005886); microtubule associated complex; (GO:0005875); cortical microtubule; (
535	2.7E+07	5	7.32E-06	Cu_mutualis	-	-	-
536	2.4E+07	1	7.35E-06	Cu_mutualism	AT1G65200	Ubiquitin carboxyl-terminal	nucleus; (GO:0005634)
537	1.3E+07	5	7.93E-06	Cu_mutualism	AT5G33392	transposable_element_gene;(source: Araport11);CACTA-like transposase	-
624	1284986	1	2.47E-08	Eu_antagonism	HCAR	7-hydroxymethyl chlorophyll a reductase, chloroplastic (EC 1.17.7.2)	chloroplast [GO:0009507]; extracellular region [GO:0005576]; 7-hydroxymethyl chlorophyll a reductase activity [GO:0090415]; iron-sulfur cluster binding [GO:0051536];
834	1568258	1	4E-07	Eu_antagonis	-	-	-
539	1579057	1	6.93E-13	Eu_antagonis	AT1G0538	Uncharacterized	metal ion binding
835	1582248	1	9.04E-06	Eu_antagonis	AT1G0538	Uncharacterized	metal ion binding
836	1.1E+07	1	9.04E-06	Eu_antagonis	AT1G3009	F-box/kelch-repeat	chloroplast
837	1.1E+07	1	3.39E-06	Eu_antagonis	-	-	-
838	2E+07	1	3.07E-06	Eu_antagonism	AT1G54820	Protein kinase superfamily protein	integral component of membrane [GO:0016021]; ATP
572	2.3E+07	1	4.32E-10	Eu_antagonism	BAN	Anthocyanidin reductase (AtANR) (EC 1.3.1.77) (Anthocyanin spotted testa) (ast) (Protein	anthocyanidin reductase activity [GO:0033729]; oxidoreductase activity [GO:0016491]; oxidoreductase activity, acting on the CH-OH group of donors, NAD
542	2.3E+07	1	3.39E-06	Eu_antagonis	KAS III	-	-
541	2.3E+07	1	3.39E-06	Eu_antagonis	KAS III	-	-
605	2.6E+07	1	7.66E-09	Eu_antagonis	-	-	-
543	2.7E+07	1	3.39E-06	Eu_antagonis	JAZ6	Jasmonate-zim-	nucleus [GO:0005634]
545	2.7E+07	1	3.39E-06	Eu_antagonis	AT1G7249	Uncharacterized	-
839	2.7E+07	1	5.39E-06	Eu_antagonis	-	-	-
840	2.7E+07	1	1.66E-06	Eu_antagonis	AT1G7250	Inter alpha-trypsin	T10D10_3
841	2.8E+07	1	3.39E-06	Eu_antagonis	-	-	-
546	7142207	2	3.39E-06	Eu_antagonism	NERD	Zinc finger CCCH domain-containing	
842	7391611	2	4E-07	Eu_antagonism	AT2G17000	Mechanosensitive ion channel protein 7 (Mechanosensitive	integral component of membrane [GO:0016021]; plasma membrane

843	9165015	2	3.39E-06	Eu_antagonism	VHA-A2	V-type proton ATPase subunit a2 (V-ATPase subunit a2) (V-type proton ATPase 95 kDa subunit a isoform 2) (V-ATPase 95 kDa isoform a2) (Vacuolar H(+)-ATPase subunit a isoform 2) (Vacuolar proton pump subunit a2) (Vacuolar proton translocating ATPase 95 kDa subunit a isoform	Golgi apparatus [GO:0005794]; integral component of membrane [GO:0016021]; mitochondrion [GO:0005739]; plant-type vacuole [GO:0000325]; plant-type vacuole membrane [GO:0009705]; vacuolar membrane [GO:0005774]; vacuolar proton-transporting V-type ATPase complex [GO:0016471]; vacuolar proton
844	1.2E+07	2	8.43E-07	Eu_antagonism	SKD1	Protein SUPPRESSOR OF K(+) TRANSPORT GROWTH DEFECT 1 (AtSKD1) (EC 3.6.4.6) (Protein VACUOLAR PROTEIN SORTING 4)	cytoplasm [GO:0005737]; multivesicular body [GO:0005771]; multivesicular body membrane [GO:0032585]; nucleus [GO:0005634]; peroxisome [GO:0005777]; plasmodesma [GO:0009506]; ATP
845	1.2E+07	2	5.39E-06	Eu_antagonis	-	-	-
846	241756	3	3.39E-06	Eu_antagonism	RGLG1	E3 ubiquitin-protein ligase RGLG1 (EC 2.3.2.27) (RING domain ligase 1)	nucleus [GO:0005634]; plasma membrane [GO:0005886]; metal ion binding [GO:0046872]; ubiquitin protein ligase activity [GO:0061630]; ubiquitin-protein transferase activity
604	296722	3	7.66E-09	Eu_antagonism	AT3G01830	Probable calcium-binding protein	calcium ion binding [GO:0005509]
847	296892	3	9.04E-06	Eu_antagonism	AT3G01830	Probable calcium-binding protein	calcium ion binding [GO:0005509]
540	301029	3	6.93E-13	Eu_antagonism	AT3G01850	(thale cress) hypothetical protein	racemase and epimerase activity, acting on carbohydrates and
848	335460	3	1.66E-06	Eu_antagonis	-	-	-
849	338522	3	5.39E-06	Eu_antagonism	AK3	Aspartokinase 3, chloroplastic (EC 2.7.2.4) (Aspartate kinase 3)	chloroplast stroma [GO:0009570]; aspartate kinase activity [GO:0004072]; ATP binding [GO:0005524]; homoserine biosynthetic

850	341030	3	5.39E-06	Eu_antagonism	AK3	Aspartokinase 3, chloroplastic (EC 2.7.2.4) (Aspartate kinase 3)	chloroplast stroma [GO:0009570]; aspartate kinase activity [GO:0004072]; ATP binding [GO:0005524]; homoserine biosynthetic
851	3103010	3	9.04E-06	Eu_antagonism	AT3G10060	Peptidyl-prolyl cis-trans isomerase FKBP16-4, chloroplastic (PPIase FKBP16-4) (EC 5.2.1.8)	chloroplast [GO:0009507]; chloroplast thylakoid [GO:0009534]; chloroplast thylakoid lumen [GO:0009543];
852	4710016	3	5.39E-06	Eu_antagonis	-	-	-
625	5084973	3	2.47E-08	Eu_antagonism	AT3G15110	(Transmembrane protein)	chloroplast thylakoid membrane
853	5111859	3	3.39E-06	Eu_antagonism	AT3G15180	ARM repeat superfamily protein	plasmodesma [GO:0009506]; vacuolar membrane
854	7951936	3	3.39E-06	Eu_antagonis	IGPD	-	-
855	7957545	3	3.39E-06	Eu_antagonis	AT3G2243	Uncharacterized	integral component of
856	7964502	3	3.39E-06	Eu_antagonism	OASA2	Putative inactive cysteine synthase 2 (Beta-substituted Ala synthase 1;2) (ARAth-Bsas1;2) (O-acetylserine	cytoplasm [GO:0005737]; cytosol [GO:0005829]; cysteine synthase activity [GO:0004124]; lyase activity [GO:0016829];
857	8594541	3	3.39E-06	Eu_antagonis	-	-	-
858	8837646	3	3.07E-06	Eu_antagonism	SYP32	Syntaxin-32 (AtSYP32)	endomembrane system [GO:0012505]; Golgi membrane [GO:0000139]; integral component of membrane [GO:0016021]; SNARE complex [GO:0031201]; SNAP
859	1.5E+07	3	1.66E-06	Eu_antagonis	-	-	-
860	1.6E+07	3	1.66E-06	Eu_antagonis	AT3G4383	-	
544	1.7E+07	3	3.39E-06	Eu_antagonis	AT3G4591	Uncharacterized	
861	2.2E+07	3	1.66E-06	Eu_antagonism	AT3G60200	Uncharacterized protein At3g60200	
862	1157252	4	5.39E-06	Eu_antagonism	AT4G02650	Putative clathrin assembly protein At4g02650	clathrin-coated pit [GO:0005905]; clathrin-coated vesicle [GO:0030136]; Golgi apparatus [GO:0005794]; pollen tube [GO:0090406]; pollen tube tip [GO:0090404]; 1-phosphatidylinositol binding [GO:0005545]; clathrin heavy chain
650	1497879	4	1.75E-07	Eu_antagonism	DFL2	Indole-3-acetic acid-amido synthetase GH3.10 (EC 6.3.2.-)	chloroplast [GO:0009507]; cytoplasm [GO:0005737]; acid-

863	5752995	4	1.66E-06	Eu_antagonis	-	-	
864	8919093	4	1.66E-06	Eu_antagonism	AT4G15640	Uncharacterized protein	-
865	8919172	4	3.07E-06	Eu_antagonism	AT4G15640	Uncharacterized protein	-
603	8921069	4	7.66E-09	Eu_antagonism	AT4G15640	Uncharacterized protein	-
866	8921350	4	3.07E-06	Eu_antagonism	AT4G15640	Uncharacterized protein	-
867	9093999	4	3.39E-06	Eu_antagonism	AT4G16050	Uncharacterized protein	-
868	1.2E+07	4	3.39E-06	Eu_antagonism	CHX17	Cation/H(+) antiporter 17 (Protein CATION/H+ EXCHANGER 17) (AtCHX17)	endomembrane system [GO:0012505]; integral component of membrane [GO:0016021]; late endosome [GO:0005770]; solute:proton antiporter
869	1.2E+07	4	3.39E-06	Eu_antagonism	CHX17	Cation/H(+) antiporter 17 (Protein CATION/H+ EXCHANGER 17) (AtCHX17)	endomembrane system [GO:0012505]; integral component of membrane [GO:0016021]; late endosome [GO:0005770]; solute:proton antiporter
870	1.4E+07	4	3.39E-06	Eu_antagonism	TPS4	Trehalose-6-phosphatase	catalytic activity [GO:0003824];
606	1.7E+07	4	7.66E-09	Eu_antagonis	-	-	-
871	1.8E+07	4	3.39E-06	Eu_antagonism	GH9B18	Cellulase (EC 3.2.1.4)	cellulase activity [GO:0008810]; cell wall organization
872	180671	5	4E-07	Eu_antagonis	AT5G0144	Uncharacterized	metal ion binding
873	4420488	5	3.39E-06	Eu_antagonism	PAO1	Polyamine oxidase 1 (AtPAO1) (EC 1.5.3.16) (EC 1.5.3.17) (N(1)-acetylpolyamine oxidase) (Spermine oxidase)	cytoplasm [GO:0005737]; flavin adenine dinucleotide binding [GO:0050660]; N1-acetylspermidine:oxygen oxidoreductase (3-acetamidopropanal-forming) activity [GO:0052904]; N1-acetylspermine:oxygen oxidoreductase (3-acetamidopropanal-forming) activity [GO:0052903]; N1-acetylspermine:oxygen

874	5255835	5	3.39E-06	Eu_antagonism	AT5G16090	Ubiquitin receptor RAD23 (DNA repair protein RAD23)	cytosol [GO:0005829]; nucleoplasm [GO:0005654]; plastid [GO:0009536]; damaged DNA binding [GO:0003684]; polyubiquitin modification-dependent protein binding
875	6738187	5	9.04E-06	Eu_antagonism	AT5G19930	Protein PGR (AtPGR) (Plasma membrane	integral component of membrane [GO:0016021];
602	9703001	5	7.66E-09	Eu_antagonism	AT5G27490	Protein YIP	Golgi apparatus [GO:0005794]; integral component of membrane
876	1.4E+07	5	3.39E-06	Eu_antagonism	YAK1	Dual specificity protein kinase YAK1 homolog (AtYAK1) (EC 2.7.12.1) (Dual specificity tyrosine-phosphorylation-regulated kinase YAK1)	cytoplasm [GO:0005737]; cytosol [GO:0005829]; plasmodesma [GO:0009506]; ATP binding [GO:0005524]; protein serine kinase activity [GO:0106310]; protein serine/threonine kinase activity [GO:0004674]; protein serine/threonine/tyrosine kinase activity [GO:0004712]; protein threonine kinase activity [GO:0106311];
547	1.6E+07	5	3.39E-06	Eu_antagonis	-	-	-
607	1.7E+07	5	7.66E-09	Eu_antagonis	-	-	-
877	1.7E+07	5	4E-07	Eu_antagonism	BKI1	BRI1 kinase inhibitor 1	cytosol [GO:0005829]; plasma membrane [GO:0005886]; protein heterodimerization activity [GO:0046982]; protein kinase inhibitor activity [GO:0004860];
878	1.7E+07	5	5.39E-06	Eu_antagonism	AT5G43440	1-aminocyclopropane-1-carboxylate	1-aminocyclopropane-1-carboxylate oxidase activity [GO:0009815];
879	1.8E+07	5	3.39E-06	Eu_antagonis	AT5G4366	Uncharacterized	-
880	1.8E+07	5	3.39E-06	Eu_antagonis	-	-	-
881	1.9E+07	5	9.04E-06	Eu_antagonis	-	-	-
585	2E+07	5	2.01E-09	Eu_antagonis	MUG6	-	-
586	2E+07	5	2.01E-09	Eu_antagonism	AT5G48970	Mitochondrial thiamine diphosphate carrier 2	integral component of mitochondrial inner membrane [GO:0031305]; mitochondrion [GO:0005739]; thiamine pyrophosphate
882	2.3E+07	5	3.39E-06	Eu_antagonis	AT5G5644	Putative FBD-	-

883	2.4E+07	5	9.04E-06	Eu_antagonism	AT5G59700	Probable receptor-like protein kinase At5g59700 (EC 2.7.11.-)	integral component of membrane [GO:0016021]; plasma membrane [GO:0005886]; plasmodesma
884	2.4E+07	5	9.04E-06	Eu_antagonism	VIP2	Protein PAF1 homolog (Protein EARLY FLOWERING 7) (Protein VERNALIZATION INDEPENDENCE 2)	Cdc73/Paf1 complex [GO:0016593]; nucleus [GO:0005634]; transcriptionally active chromatin [GO:0035327]; chromatin binding [GO:0003682]; RNA polymerase II complex
571	2.4E+07	5	4.32E-10	Eu_antagonism	SBT5.4	Subtilisin-like protease SBT5.4 (EC 3.4.21.-) (Subtilase subfamily 5	endoplasmic reticulum [GO:0005783]; plasma membrane [GO:0005886]; serine-type endopeptidase
538	6405664	5	9.83E-06	Eu_aggression	AT5G19097	transposable_element_gene;(source: Araport11);copia-like	-
40	9967780	5	1.67E-07	Eu_mutualis	-	-	-
41	1.4E+07	5	2.54E-07	Eu_mutualism	AT5G35602	transposable_element_gene;(source: Araport11);gypsy-like	-
42	1.4E+07	5	2.54E-07	Eu_mutualism	AT5G35615	pseudogene of Ribonuclease H-	-
43	2.1E+07	3	3.34E-06	Eu_mutualis	-	-	-
44	1.9E+07	1	3.34E-06	Eu_mutualism	DWF5	Ergosterol biosynthesis ERG4/ERG24 family	7-dehydrocholesterol reductase activity；(GO:0047598)；plasma membrane；(GO:0005886)；oxidoreductase activity, acting on the CH-CH group of donors；(GO:0016627)；unidimensional cell growth；(GO:0009826)；sterol biosynthetic process；(GO:0016126
45	5543599	1	8.56E-07	Eu_mutualism	RAD23A	The protein encoded by this gene is one of two human homologs of Saccharomyces cerevisiae Rad23, a protein involved in nucleotide excision repair.	nucleotide-excision repair；(GO:0006289)；proteasome-mediated ubiquitin-dependent protein catabolic process；(GO:0043161)；damaged DNA binding；(
46	2866886	4	3.92E-07	Eu_mutualis	-	-	-

47	1.8E+07	1	8.56E-07	Eu_mutualism	AT1G49450	Transducin/WD40 repeat-like	nucleus； (GO:0005634)；
48	358189	2	8.56E-07	Eu_mutualism	AT2G01820	Transmembrane kinase (TMK), member of the plant receptor-like kinase (RLK) family. TMKs are characterized by an extracellular	plasmodesma； (GO:0009506)； plant-type cell wall； (GO:0009505)； plasma membrane； (GO:0005886)； protein phosphorylation； (GO:0006468)； pollen
49	1.4E+07	5	2.67E-08	Eu_mutualism	AT5G35615	pseudogene of Ribonuclease H-	-
50	5545724	1	3.34E-06	Eu_mutualism	RAD23A	The protein encoded by this gene is one of two human homologs of Saccharomyces cerevisiae Rad23, a protein involved in nucleotide excision repair.	nucleotide-excision repair； (GO:0006289)； proteasome-mediated ubiquitin-dependent protein catabolic process； (GO:0043161)； damaged DNA binding； (
51	358617	2	3.34E-06	Eu_mutualism	AT2G01820	Transmembrane kinase (TMK), member of the plant receptor-like kinase (RLK) family. TMKs are characterized by an extracellular	plasmodesma； (GO:0009506)； plant-type cell wall； (GO:0009505)； plasma membrane； (GO:0005886)； protein phosphorylation； (GO:0006468)； pollen
52	6522489	4	3.34E-06	Eu_mutualis	MEE53	Cysteine/Histidine	-
53	9472315	5	4.10E-06	Eu_mutualis	-	-	-
56	1.2E+07	1	5.57E-06	Eu_mutualism	HAF01	HAC13 protein (HAC13)	nucleus； (GO:0005634)； RNA polymerase II general transcription initiation factor activity； (GO:0016251)； DNA mediated transformation； (GO:0009294)； TBP
57	1.4E+07	5	5.63E-06	Eu_mutualis	-	-	-
62	9293684	4	3.53E-07	Eu_mutualism	AT4G16490	ARM repeat superfamily	cytoplasm； (GO:0005737)；
63	2.8E+07	1	4.34E-06	Eu_mutualis	CYP721A1	cytochrome P450,	-
505	2903965	4	8.50E-06	Eu_mutualism	AT4G05594	transposable_element_gene;(source: Araport11);copia-like	-

513	1.9E+07	1	1.82E-06	Eu_mutualism	VIIIA	P-loop containing nucleoside triphosphate hydrolases superfamily protein	microfilament motor activity；(GO:0000146)；vesicle；(GO:0031982)；motor activity；(GO:0003774)；actin cytoskeleton；(GO:0015629)；plasma membrane；(GO:0005886)；actin filament-based
514	1.7E+07	4	4.69E-06	Eu_mutualism	MLH3	This gene is a member of the MutL-homolog (MLH) family of DNA mismatch repair (MMR) genes. MLH genes are implicated in maintaining	nucleus；(GO:0005634)；mismatch repair complex；(GO:0032300)；reciprocal meiotic recombination；(GO:0007131)；ATP binding；(
515	1.7E+07	4	4.69E-06	Eu_mutualism	MLH3	This gene is a member of the MutL-homolog (MLH) family of DNA mismatch repair (MMR) genes. MLH genes are implicated in maintaining	nucleus；(GO:0005634)；mismatch repair complex；(GO:0032300)；reciprocal meiotic recombination；(GO:0007131)；ATP binding；(
524	2803877	4	2.39E-06	Eu_mutualis	-	-	-
677	1.7E+07	4	1.82E-06	Eu_mutualism	AT4G36190	Serine carboxypeptidase S28 family protein;(source:Araport11)	proteolysis；(GO:0006508)；serine-type peptidase activity；(GO:0008236)；vacuole；(
678	1.9E+07	5	3.16E-06	Eu_mutualis	AT5G4655	DNA-binding	nucleus；(
679	1.4E+07	5	3.68E-06	Eu_mutualism	AT5G35690	WT-like growth phenotype mutants of WSS1B do not	nucleus；(GO:0005634)
680	1.4E+07	5	3.68E-06	Eu_mutualism	AT5G03545	Expressed in roots in response to phosphate starvation. this	cellular response to phosphate starvation；(GO:0016036)；mitochondrion；(
681	1.4E+07	5	3.68E-06	Eu_mutualism	AT5G35690	WT-like growth phenotype mutants of WSS1B do not	nucleus；(GO:0005634)
682	2.4E+07	1	4.10E-06	Eu_mutualism	EGL3	basic helix-loop-helix (bHLH) DNA-binding superfamily protein	protein dimerization activity；(GO:0046983)；DNA-binding transcription factor activity；(GO:0003700)；regulation of transcription, DNA-templated；(GO:0006355)；

683	2.1E+07	5	4.10E-06	Eu_mutualism	AT5G51400	PLAC8 family protein;(source:Araport11)	Golgi apparatus; (GO:0005794); nucleus; (GO:0005634);
684	2E+07	3	4.10E-06	Eu_mutualism	PTR1	peptide transporter 1	transmembrane transport; (GO:0055085); tripeptide transmembrane transporter activity; (GO:0042937); plasma
685	2126066	3	5.36E-06	Eu_mutualis	TPXz	-	-
686	1.4E+07	5	5.57E-06	Eu_mutualis	-	-	-
687	4548634	1	6.61E-06	Eu_mutualism	AOC4	allene oxide cyclase 4	mitochondrion; (GO:0005739); jasmonic acid biosynthetic process; (GO:0009695); chloroplast; (
688	5533737	1	8.37E-06	Eu_mutualism	WAKL4	wall associated kinase-like 4	plant-type cell wall; (GO:0009505); protein serine/threonine kinase activity; (GO:0004674); response to cadmium ion; (GO:0046686); response to zinc ion; (GO:0010043); polysaccharide binding; (GO:0030247); plasma
74	4653504	1	1.51E-06	Gu_aggressio	-	-	-
94	1.4E+07	5	6.80E-06	Gu_aggression	AT5G35525	PLAC8 family protein;(source:Araport11)	cytoplasm; (GO:0005737); biological_process_unk
96	1.4E+07	5	1.29E-06	Gu_aggression	AT5G35332	transposable_element_gene;(source: Araport11);pseudo	-
97	1.4E+07	5	3.82E-06	Gu_aggression	AT5G35475	General transcription factor	molecular_function_unknown; (GO:0003674
109	5109002	4	2.90E-07	Gu_aggressio	-	-	-
136	1.4E+07	5	1.70E-06	Gu_aggression	AT5G35495	transposable_element_gene;(source: Araport11);non-LTR	-
385	1.1E+07	3	9.86E-06	Gu_aggression	AT3G29175	transposable_element_gene;(source: Araport11);Mutator-like transposase	-
415	667064	5	6.59E-07	Gu_aggression	UPL4	ubiquitin-protein ligase 4	nucleus; (GO:0005634); ubiquitin-protein transferase activity; (GO:0004842); protein

689	2432431	4	3.10E-08	Gu_aggression	MED21	This gene encodes a member of the mediator complex subunit 21 family. The encoded protein interacts with the human RNA polymerase II holoenzyme and	nucleus；(GO:0005634)；regulation of transcription by RNA polymerase II；(GO:0006357)；mediator complex；(GO:0016592)；defense response to
690	4845891	3	7.70E-07	Gu_aggressio	-	-	-
691	578069	5	1.41E-06	Gu_aggressio	-	-	-
692	579375	5	1.55E-06	Gu_aggressio	-	-	-
693	955304	1	1.78E-06	Gu_aggression	SOM	Zinc finger C-x8-C-x5-C-x3-H type family protein	cytoplasm；(GO:0005737)； protein binding；(GO:0005515)； DNA-binding transcription factor activity；(
694	623749	5	1.90E-06	Gu_aggressio	-	-	-
695	5214077	4	2.08E-06	Gu_aggressio	-	-	-
696	690838	5	2.30E-06	Gu_aggression	AT5G02950	Tudor/PWWP/MBT superfamily protein;(source:Ar	nucleus；(GO:0005634)；molecular_function_un
697	2.7E+07	1	2.77E-06	Gu_aggressio	-	-	-
698	1.8E+07	2	3.73E-06	Gu_aggression	AT2G42240	RNA-binding (RRM/RBD/RNP	nucleus；(GO:0005634)； mRNA
699	5216649	4	3.84E-06	Gu_aggressio	-	-	-
700	4654714	1	3.99E-06	Gu_aggressio	-	-	-
701	5102442	1	4.59E-06	Gu_aggression	AT1G14800	Nucleic acid-binding, OB-fold-like	molecular_function_unknown；(GO:0003674)；
702	1.4E+07	5	5.48E-06	Gu_aggression	AT5G35400	Pseudouridine synthase family protein;(source:Araport11)	pseudouridine synthase activity；(GO:0009982)； RNA binding；(GO:0003723)； tRNA pseudouridine synthesis；(
703	668716	5	5.60E-06	Gu_aggression	UPL4	ubiquitin-protein ligase 4	nucleus；(GO:0005634)；ubiquitin-protein transferase activity；(GO:0004842)； protein
704	5775050	3	5.63E-06	Gu_aggression	AAE7	acyl-activating enzyme 7	acetate-CoA ligase activity；(GO:0003987)； AMP binding；(GO:0016208)；peroxisome；(GO:0005777)； acetate metabolic process；(GO:0006083)；
705	2386376	4	8.41E-06	Gu_aggressio	CPK27	calcium-dependent	-
706	5230364	4	9.23E-06	Gu_aggression	AT4G08280	Thioredoxin superfamily protein;(source:Ar	molecular_function_unknown；(GO:0003674)； nucleus；(
4	1.3E+07	4	5.64E-06	Gu_altruism	-	-	-
8	1.4E+07	5	8.42E-06	Gu_altruism	-	-	-
39	1.7E+07	4	9.20E-06	Gu_altruism	-	-	-

670	2.6E+07	5	1.67E-06	Gu_antagonis	AT5G6558	-	-
10	7746139	1	4.24E-06	Gu_mutualis	-	-	-
46	2866886	4	4.24E-06	Gu_mutualis	-	-	-
49	1.4E+07	5	6.37E-06	Gu_mutualism	AT5G35615	pseudogene of Ribonuclease H-	-
498	3943227	1	1.08E-06	Gu_mutualis	AT1G0486	None;(source:Arap	-
505	2903965	4	7.60E-09	Gu_mutualism	AT4G05594	transposable_element_gene;(source: Araport11);copia-like	-
513	1.9E+07	1	2.59E-09	Gu_mutualism	VIIIA	P-loop containing nucleoside triphosphate hydrolases superfamily protein	microfilament motor activity; (GO:0000146); vesicle; (GO:0031982); motor activity; (GO:0003774); actin cytoskeleton; (GO:0015629); plasma membrane; (GO:0005886); actin filament-based
514	1.7E+07	4	2.95E-09	Gu_mutualism	MLH3	This gene is a member of the MutL-homolog (MLH) family of DNA mismatch repair (MMR) genes. MLH genes are implicated in maintaining	nucleus; (GO:0005634); mismatch repair complex; (GO:0032300); reciprocal meiotic recombination; (GO:0007131); ATP binding; (
515	1.7E+07	4	2.95E-09	Gu_mutualism	MLH3	This gene is a member of the MutL-homolog (MLH) family of DNA mismatch repair (MMR) genes. MLH genes are implicated in maintaining	nucleus; (GO:0005634); mismatch repair complex; (GO:0032300); reciprocal meiotic recombination; (GO:0007131); ATP binding; (
517	1783331	4	2.12E-06	Gu_mutualism	AT4G03824	transposable_element_gene;(source: Araport11);Mariner-like transposase	-
518	2.1E+07	5	9.01E-06	Gu_mutualism	ABCG8	The protein encoded by this	-
519	2.5E+07	5	5.03E-06	Gu_mutualism	ABCA12	The membrane-associated protein	-
520	1640779	4	6.93E-06	Gu_mutualis	-	-	-
522	1652367	4	5.46E-08	Gu_mutualism	AT4G03728	hypothetical protein;(source:Araport11)	molecular_function_unknown; (GO:0003674);

523	2817901	4	2.87E-10	Gu_mutualism	IBR1	indole-3-butyric acid response 1	peroxisome；(GO:0005777)；indolebutyric acid metabolic process；(GO:0080024)；chloroplast；(
524	2803877	4	7.05E-10	Gu_mutualis	-	-	-
532	1.8E+07	5	8.37E-07	Gu_mutualism	AT5G44010	fanconi anemia group F protein (FANCF);(source: Araport11)	cellular response to DNA damage stimulus；(GO:0006974)；Fanconi anaemia nuclear complex；(
677	1.7E+07	4	7.88E-09	Gu_mutualism	AT4G36190	Serine carboxypeptidase S28 family protein;(source:Araport11)	proteolysis；(GO:0006508)；serine-type peptidase activity；(GO:0008236)；vacuole；(
687	4548634	1	9.47E-06	Gu_mutualism	AOC4	allene oxide cyclase 4	mitochondrion；(GO:0005739)；jasmonic acid biosynthetic process；(GO:0009695)；chloroplast；(
707	1.6E+07	2	1.65E-07	Gu_mutualis	-	-	-
708	1993791	4	1.65E-07	Gu_mutualism	AT4G04145	transposable_element_gene;(source: Araport11);copia-like	-
709	1761978	4	2.38E-07	Gu_mutualism	AT4G03813	transposable_element_gene;(source: Araport11);non-LTR	-
710	7074687	1	3.77E-07	Gu_mutualism	AT1G20400	hypothetical protein (DUF1204);(sourc	biological_process_unknown；(GO:0008150)；
711	1.6E+07	2	8.55E-07	Gu_mutualism	FAC1	AMP deaminase, putative / myoadenylate deaminase	protein binding；(GO:0005515)；cytosol；(GO:0005829)；embryo development ending in seed dormancy；(GO:0009793)；integral component of mitochondrial outer membrane；(GO:0031307)；response to abscisic acid；(GO:0009737

712	1.6E+07	2	8.55E-07	Gu_mutualism	FAC1	AMP deaminase, putative / myoadenylate deaminase	protein binding; (GO:0005515); cytosol; (GO:0005829); embryo development ending in seed dormancy; (GO:0009793); integral component of mitochondrial outer membrane; (GO:0031307); response to abscisic acid; (GO:0009737
713	1.9E+07	5	8.78E-07	Gu_mutualism	AT5G46850	phosphatidylinositol-glycan biosynthesis class X-like protein;(source:Ar	nucleus; (GO:0005634); molecular_function_unknown; (GO:0003674); GPI anchor
714	1.9E+07	5	8.78E-07	Gu_mutualism	AT5G46850	phosphatidylinositol-glycan biosynthesis class X-like protein;(source:Ar	nucleus; (GO:0005634); molecular_function_unknown; (GO:0003674); GPI anchor
715	1.9E+07	5	8.78E-07	Gu_mutualism	VAM3	Syntaxin/t-SNARE family protein	SNARE binding; (GO:0000149); SNAP receptor activity; (GO:0005484); plant-type vacuole membrane; (GO:0009705); endomembrane system; (GO:0012505); stomatal movement; (GO:0010118); protein binding; (GO:0005515); late endosome; (GO:0005770); negative gravitropism; (GO:0009959);

716	1.9E+07	5	8.78E-07	Gu_mutualism	VAM3	Syntaxin/t-SNARE family protein	SNARE binding; (GO:0000149); SNAP receptor activity; (GO:0005484); plant-type vacuole membrane; (GO:0009705); endomembrane system; (GO:0012505); stomatal movement; (GO:0010118); protein binding; (GO:0005515); late endosome; (GO:0005770); negative gravitropism; (GO:0009959);
717	1247406	3	8.97E-07	Gu_mutualis	MUG1	-	-
718	2853130	4	1.12E-06	Gu_mutualism	AT4G05582	transposable_element_gene;(source: Araport11);gypsy-like	-
719	1.6E+07	2	1.16E-06	Gu_mutualism	AT2G38260	Probably not a pseudogene based	-
720	1.5E+07	5	1.49E-06	Gu_mutualis	-	-	-
721	1.9E+07	5	1.90E-06	Gu_mutualism	VAM3	Syntaxin/t-SNARE family protein	SNARE binding; (GO:0000149); SNAP receptor activity; (GO:0005484); plant-type vacuole membrane; (GO:0009705); endomembrane system; (GO:0012505); stomatal movement; (GO:0010118); protein binding; (GO:0005515); late endosome; (GO:0005770); negative gravitropism; (GO:0009959);

722	1.9E+07	5	1.90E-06	Gu_mutualism	VAM3	Syntaxin/t-SNARE family protein	SNARE binding； (GO:0000149)； SNAP receptor activity； (GO:0005484)； plant-type vacuole membrane； (GO:0009705)； endomembrane system； (GO:0012505)； stomatal movement； (GO:0010118)； protein binding； (GO:0005515)； late endosome； (GO:0005770)； negative gravitropism； [illegible]
723	4469268	1	2.07E-06	Gu_mutualism	GLE1	This gene encodes a predicted 75-kDa polypeptide with high sequence and structure homology to yeast Gle1p	poly(A)+ mRNA export from nucleus； (GO:0016973)； protein binding； (GO:0005515)； regulation of translational initiation； (GO:0006446)； mRNA export from nucleus； (GO:0006406)； embryo development ending in seed dormancy； [illegible]
724	716012	4	2.09E-06	Gu_mutualis	MYB55	myb domain	-
725	1.7E+07	4	2.57E-06	Gu_mutualism	NTRB	NADPH-dependent thioredoxin reductase B	pollen germination； (GO:0009846)； positive regulation of cell division； (GO:0051781)； cell redox homeostasis； (GO:0045454)； mitochondrion； (GO:0005739)； removal of superoxide radicals； (GO:0019430)；
726	1509997	4	2.63E-06	Gu_mutualis	-	-	-
727	1235552	3	2.89E-06	Gu_mutualism	EIN4	Signal transduction histidine kinase, hybrid-type, ethylene sensor	negative regulation of ethylene-activated signaling pathway； (GO:0010105)； protein histidine kinase activity； (GO:0004673)； ethylene receptor activity； (GO:0038199)； ethylene binding； [illegible]

728	1238302	3	2.89E-06	Gu_mutualism	EIN4	Signal transduction histidine kinase, hybrid-type, ethylene sensor	negative regulation of ethylene-activated signaling pathway; (GO:0010105); protein histidine kinase activity; (GO:0004673); ethylene receptor activity; (GO:0038199); ethylene binding; (
729	1.7E+07	1	3.56E-06	Gu_mutualism	AT1G47360	transposable_element_gene;(source:Araport11);copia-like	-
730	9877773	5	3.64E-06	Gu_mutualis	-	-	-
731	9799098	1	3.67E-06	Gu_mutualism	AT1G28090	Polynucleotide adenylyltransferas	RNA processing; (GO:0006396);
732	3477652	5	3.70E-06	Gu_mutualism	AT5G10990	SAUR-like auxin-responsive protein family;(source:Ara	response to auxin; (GO:0009733); molecular_function_un
733	1.8E+07	5	3.70E-06	Gu_mutualism	AT5G44000	Glutathione S-transferase family protein;(source:Ar	cytoplasm; (GO:0005737); glutathione transferase
734	2804637	4	4.24E-06	Gu_mutualism	EHD2	This gene encodes a member of the EH domain-containing protein family. These proteins are characterized by a C-terminal EF-hand domain, a	intracellular membrane-bounded organelle; (GO:0043231); plasma membrane; (GO:0005886); endosomal transport; (GO:0016197); calcium ion binding; (GO:0005509); protein
735	2841947	4	4.24E-06	Gu_mutualism	AT4G05580	transposable_element_gene;(source:Araport11);similar	-
736	1.7E+07	4	4.91E-06	Gu_mutualism	AT4G35410	Clathrin adaptor complex small chain family protein;(source:Araport11)	intracellular protein transport; (GO:0006886); mitochondrion; (GO:0005739);
737	1.7E+07	2	6.03E-06	Gu_mutualism	TBL30	trichome birefringence-like	Golgi apparatus; (GO:0005794); O-
738	2796884	4	6.20E-06	Gu_mutualis	-	-	-
739	1.7E+07	4	7.00E-06	Gu_mutualism	AT4G36790	Major facilitator superfamily protein;(source:Araport11)	transmembrane transporter activity; (GO:0022857); Golgi medial cisterna; (

740	1.9E+07	5	7.35E-06	Gu_mutualism	VAM3	Syntaxin/t-SNARE family protein	[illegible] (GO:0000149); SNAP receptor activity; (GO:0005484); plant-type vacuole membrane; (GO:0009705); endomembrane system; (GO:0012505); stomatal movement; (GO:0010118); protein binding; (GO:0005515); late endosome; (GO:0005770); negative gravitropism; [illegible]
741	1.5E+07	5	7.56E-06	Gu_mutualis	-	-	-
742	996502	4	8.48E-06	Gu_mutualism	SUS3	sucrose synthase 3	response to mannitol; (GO:0010555); transferase activity, transferring glycosyl groups; (GO:0016757); response to water deprivation; [illegible]
743	5601018	2	8.50E-06	Gu_mutualism	AT2G13460	transposable_element_gene;(source: Araport11);non-LTR	-
744	1.7E+07	4	8.94E-06	Gu_mutualism	DRL1		purine nucleotide binding; (GO:0017076); calmodulin binding; (GO:0005516); tRNA modification; (GO:0006400); regulation of transcription by RNA polymerase II; (
745	1.9E+07	1	9.10E-06	Gu_mutualism	AT1G50350	E3 ubiquitin-protein ligase	ubiquitin protein ligase activity; (GO:0061630
746	1.6E+07	2	9.65E-06	Gu_mutualis	-	-	-
70	1.3E+07	3	4.34E-07	Pu_aggressio	-	-	-
71	1.3E+07	1	3.13E-06	Pu_aggressio	-	-	-
72	1.1E+07	5	2.88E-06	Pu_aggressio	-	-	-
73	1.4E+07	5	6.27E-06	Pu_aggressio	-	-	-
74	4653504	1	1.69E-06	Pu_aggressio	-	-	-

76	2194772	3	5.23E-06	Pu_aggression	PDE320	pigment defective 320	nucleus; (GO:0005634); acylglycerol transport; (GO:0034196); chloroplast envelope; (GO:0009941); cytosol; (GO:0005829); chloroplast outer membrane; (GO:0009707); chloroplast; (GO:0009507); ER to
77	2.7E+07	1	7.15E-06	Pu_aggressio	AT1G7207	Chaperone DnaJ-	cytoplasm; (
73	1.4E+07	5	3.52E-08	Pu_antagonis	-	-	-
74	4653504	1	2.63E-07	Pu_antagonis	-	-	-
76	2194772	3	2.61E-09	Pu_antagonism	PDE320	pigment defective 320	nucleus; (GO:0005634); acylglycerol transport; (GO:0034196); chloroplast envelope; (GO:0009941); cytosol; (GO:0005829); chloroplast outer membrane; (GO:0009707); chloroplast; (GO:0009507); ER to
85	3556389	5	4.94E-12	Pu_antagonism	UAP56a	DEAD/DEAH box RNA helicase family protein	ATPase activity; (GO:0016887); mRNA binding; (GO:0003729); nucleus; (GO:0005634); response to cadmium ion; (GO:0046686); nucleolus; (GO:0005730); protein binding; (
86	1.7E+07	3	3.33E-09	Pu_antagonism	AT3G46420	Leucine-rich repeat protein kinase family protein;(source:Ar	protein phosphorylation; (GO:0006468); extracellular region; (
87	9984166	5	3.77E-09	Pu_antagonism	AT5G27950	P-loop containing nucleoside triphosphate hydrolases superfamily protein;(source:Araport11)	ATP-dependent microtubule motor activity, plus-end-directed; (GO:0008574); microtubule-based movement; (GO:0007018); cytoskeleton-dependent
88	3280058	5	2.44E-09	Pu_antagonis	-	-	-
89	3564738	5	9.68E-10	Pu_antagonis	AT5G0185	None;(source:Arap	0

90	1.3E+07	5	3.25E-10	Pu_antagonism	PTM	PHD type transcription factor	-
91	3509204	5	2.05E-10	Pu_antagonis	-	-	-
92	1.4E+07	5	6.98E-09	Pu_antagonism	AT5G35348	transposable_element_gene;(source:	-
93	6328771	4	1.76E-08	Pu_antagonism	AT4G10150	RING/U-box superfamily protein;(source:Araport11)	ubiquitin-dependent protein catabolic process; (GO:0006511); ubiquitin protein
94	1.4E+07	5	6.25E-09	Pu_antagonism	AT5G35525	PLAC8 family protein;(source:Araport11)	cytoplasm; (GO:0005737); biological_process_unk
95	1.4E+07	5	4.76E-09	Pu_antagonis	-	-	-
96	1.4E+07	5	5.14E-09	Pu_antagonism	AT5G35332	transposable_element_gene;(source:Araport11);pseudo	-
97	1.4E+07	5	3.79E-09	Pu_antagonism	AT5G35475	General transcription factor	molecular_function_unknown; (GO:0003674
98	3408362	5	2.36E-08	Pu_antagonism	AT5G10780	ER membrane protein complex subunit-like protein;(source:Ar	nucleus; (GO:0005634); ER membrane protein complex; (
99	3409353	5	2.36E-08	Pu_antagonism	AT5G10780	ER membrane protein complex subunit-like protein;(source:Ar	nucleus; (GO:0005634); ER membrane protein complex; (
100	3410949	5	2.36E-08	Pu_antagonis	UBP22	ubiquitin-specific	-
101	3416182	5	2.36E-08	Pu_antagonism	AT5G10800	RNA recognition motif (RRM)-	cytoplasm; (GO:0005737);
102	3419070	5	2.36E-08	Pu_antagonism	ER	Leucine-rich receptor-like protein kinase family protein	regulation of plant organ morphogenesis; (GO:1905421); plasma membrane; (GO:0005886); regulation of cell division; (GO:0051302); protein binding; (GO:0005515); regulation of cell adhesion; (GO:0030155); polarity specification of adaxial/abaxial axis; (GO:0009944); signaling receptor binding; (GO:0005102); receptor serine/threonine kinase binding; (GO:0033612);
103	3421461	5	2.36E-08	Pu_antagonis	AT5G1082	Major facilitator	chloroplast; (

104	3745460	5	2.38E-08	Pu_antagonism	AT5G11650	alpha/beta-Hydrolases superfamily protein;(source:Ar	Golgi apparatus; (GO:0005794); lipase activity; (GO:0016298); endoplasmic
105	4514041	3	1.20E-07	Pu_antagonism	BGAL1	beta galactosidase 1	plant-type cell wall; (GO:0009505); vacuole; (GO:0005773); beta-galactosidase activity; (GO:0004565); plasmodesma; (
106	3670243	5	2.21E-09	Pu_antagonism	AT5G11480	P-loop containing nucleoside	nucleus; (GO:0005634); GTP
107	8074644	3	2.54E-07	Pu_antagonis	-	-	-
108	1.4E+07	5	5.75E-08	Pu_antagonism	AT5G35555	transposable_element_gene;(source:Araport11);copia-like	-
109	5109002	4	2.72E-08	Pu_antagonis	-	-	-
110	2E+07	1	1.21E-07	Pu_antagonism	AT1G52510	alpha/beta-Hydrolases superfamily protein;(source:Ar	chloroplast envelope; (GO:0009941); chloroplast stroma; (GO:0009570);
111	2E+07	1	1.21E-07	Pu_antagonism	FRS6	FAR1-related sequence 6	nucleus; (GO:0005634); regulation of
112	2E+07	1	1.21E-07	Pu_antagonis	-	-	-
113	3410142	5	9.15E-08	Pu_antagonis	UBP22	ubiquitin-specific	-
114	3603933	3	7.69E-08	Pu_antagonism	MYB65	myb domain protein 65	nucleus; (GO:0005634); anther development; (GO:0048653); DNA-binding transcription factor activity; (GO:0003700); positive regulation of programmed cell death; (GO:0043068); anther wall tapetum morphogenesis; (GO:0048655); transcription regulatory region sequence-
115	3604005	3	7.69E-08	Pu_antagonism	MYB66	myb domain protein 65	protein binding; (GO:0005515); DNA binding; (GO:0003677); nucleus; (GO:0005634); transcription regulatory region sequence-specific DNA
116	3562004	5	2.59E-09	Pu_antagonis	-	-	-
117	1.3E+07	5	1.01E-07	Pu_antagonism	AT5G35230	hypothetical protein;(source:Araport11)	molecular_function_unknown; (GO:0003674);

118	2E+07	1	1.03E-07	Pu_antagonism	AT1G52710	Rubredoxin-like superfamily protein;(source:Araport11)	cytochrome-c oxidase activity; (GO:0004129); mitochondrial envelope; (GO:0005740); mitochondrial electron
119	1.3E+07	5	2.47E-07	Pu_antagonism	AT5G35230	hypothetical protein;(source:Araport11)	molecular_function_unknown; (GO:0003674);
120	1E+07	5	3.32E-07	Pu_antagonis	AT5G2797	ARM repeat	plasma membrane; (
121	1E+07	5	3.32E-07	Pu_antagonis	AT5G2797	ARM repeat	plasma membrane; (
122	7078163	1	3.90E-07	Pu_antagonis	-	-	-
123	1.6E+07	4	2.75E-08	Pu_antagonis	EDA39	calmodulin-	-
124	3605408	3	3.95E-08	Pu_antagonism	AT3G11450	Encodes a ZRF1 chromatin regulator. Functions in regulating plant growth and development	cytosol; (GO:0005829); embryo development ending in seed dormancy; (GO:0009793); chromatin silencing; (GO:0006342);
125	6115036	2	1.55E-06	Pu_antagonism	AT2G14400	transposable_element_gene;(source:Araport11);gypsy-like retrotransposon family, has a 5.4e-	-
126	3408858	5	4.06E-07	Pu_antagonism	AT5G10780	ER membrane protein complex subunit-like protein;(source:Ar	nucleus; (GO:0005634); ER membrane protein complex; (
127	3422619	5	4.06E-07	Pu_antagonis	AT5G1082	Major facilitator	chloroplast; (
128	6106176	1	3.53E-07	Pu_antagonis	-	-	-
129	2082367	4	4.56E-07	Pu_antagonism	AT4G04296	transposable_element_gene;(source:Araport11);non-LTR	-
130	3635394	3	2.02E-07	Pu_antagonism	SPY	Tetratricopeptide repeat (TPR)-like superfamily protein	nucleus; (GO:0005634); cytoplasm; (GO:0005737); protein N-acetylglucosaminyltransferase activity; (GO:0016262); regulation of reactive oxygen species metabolic process; (GO:2000377); cytosol; (GO:0005829); protein binding; (
131	3638338	3	2.02E-07	Pu_antagonism	CASP2	This gene encodes a member of the	-
132	9957213	5	6.70E-07	Pu_antagonis	SADHU5-2	hypothetical	-

133	3829977	3	1.28E-06	Pu_antagonism	AT3G12020	P-loop containing nucleoside triphosphate hydrolases superfamily protein;(source:Ar	chloroplast；(GO:0009507)； microtubule binding；(GO:0008017)； microtubule；(GO:0005874)；
134	6782787	1	1.43E-07	Pu_antagonis	-	-	-
135	6616766	5	2.32E-07	Pu_antagonis	SULTR3;5	sulfate transporter	-
136	1.4E+07	5	4.10E-07	Pu_antagonism	AT5G35495	transposable_element_gene;(source:Araport11);non-LTR	-
137	3277274	5	4.94E-07	Pu_antagonis	-	-	-
138	2E+07	2	1.01E-07	Pu_antagonis	CRP6	-	-
139	2E+07	1	3.77E-07	Pu_antagonism	AT1G52510	alpha/beta-Hydrolases superfamily protein;(source:Ar	chloroplast envelope；(GO:0009941)； chloroplast stroma；(GO:0009570)；
140	2E+07	5	8.48E-07	Pu_antagonis	-	-	-
141	1.3E+07	5	1.73E-07	Pu_antagonism	AT5G35200	ENTH/ANTH/VHS superfamily protein;(source:Araport11)	clathrin-coated vesicle；(GO:0030136)； vesicle budding from membrane；(GO:0006900)； SNARE binding；(GO:0000149)； clathrin heavy chain binding；(GO:0032050)； plasma
142	7049722	5	4.54E-07	Pu_antagonis	-	-	-
143	2E+07	1	2.52E-07	Pu_antagonism	CDT1		nucleus；(GO:0005634)； detoxification of
144	3557596	5	1.02E-07	Pu_antagonis	GLR2.6		-
145	2E+07	1	6.46E-07	Pu_antagonism	AT1G52730	Transducin/WD40 repeat-like superfamily protein;(source:Araport11)	cytoplasm；(GO:0005737)； Cul4-RING E3 ubiquitin ligase complex；(GO:0080008)；
146	6598438	5	2.36E-07	Pu_antagonism	ASP2	-	2-oxoglutarate metabolic process；(GO:0006103)； mitochondrion；(GO:0005739)； copper ion binding；(GO:0005507)； L-aspartate:2-oxoglutarate aminotransferase activity；(GO:0004069)； plasma membrane；(GO:0005886)； pyridoxal phosphate binding；(GO:0030170)； cellular
148	1.2E+07	2	6.58E-06	Pu_antagonis	-	-	-

149	1.2E+07	2	6.58E-06	Pu_antagonism	EXO70H5	exocyst subunit exo70 family	pollen tube; (GO:0090406);
150	1.4E+07	1	4.15E-06	Pu_antagonis	-	-	-
151	2E+07	5	8.48E-07	Pu_antagonism	AT5G50310	Galactose oxidase/kelch	biological_process_unknown; (GO:0008150
152	2E+07	5	8.48E-07	Pu_antagonism	ELO3	radical SAM domain-containing protein / GCN5-related N-acetyltransferase (GNAT) family protein	sphingolipid biosynthetic process; (GO:0030148); mitochondrion; (GO:0005739); fatty acid elongation, polyunsaturated fatty acid; (GO:0034626); endoplasmic reticulum; (
153	2E+07	5	8.48E-07	Pu_antagonism	ELO3	radical SAM domain-containing protein / GCN5-related N-acetyltransferase (GNAT) family protein	sphingolipid biosynthetic process; (GO:0030148); mitochondrion; (GO:0005739); fatty acid elongation, polyunsaturated fatty acid; (GO:0034626); endoplasmic reticulum; (
154	6830174	5	4.33E-06	Pu_antagonism	PI	K-box region and MADS-box transcription factor family protein	regulation of transcription by RNA polymerase II; (GO:0006357); cytoplasm; (GO:0005737); RNA polymerase II cis-regulatory region sequence-specific DNA binding; (GO:0000978); nucleus; (GO:0005634); protein binding; (GO:0005515);
155	1.3E+07	5	6.18E-07	Pu_antagonis	-	-	-
156	6109521	1	5.32E-07	Pu_antagonism	PEPR2	PEP1 receptor 2	protein serine kinase activity; (GO:0106310); peptide binding; (GO:0042277); kinase activity; (GO:0016301); protein binding; (GO:0005515); plasma membrane; (GO:0005886);
157	2E+07	1	1.44E-06	Pu_antagonis	-	-	-
158	2E+07	1	1.44E-06	Pu_antagonis	-	-	-
159	2E+07	1	1.44E-06	Pu_antagonis	-	-	-

160	2E+07	1	1.44E-06	Pu_antagonism	AT1G53025	Ubiquitin-conjugating enzyme family protein;(source:Araport11)	nucleus； (GO:0005634)； postreplication repair； (GO:0006301)； protein K63-linked
161	2E+07	1	1.44E-06	Pu_antagonism	AT1G53025	Ubiquitin-conjugating enzyme family protein;(source:Araport11)	nucleus； (GO:0005634)； postreplication repair； (GO:0006301)； protein K63-linked
162	2E+07	1	1.44E-06	Pu_antagonis	-	-	-
163	2E+07	1	1.44E-06	Pu_antagonism	AT1G53035	transmembrane protein;(source:Araport11)	molecular_function_unknown； (GO:0003674)； chloroplast； (
164	2E+07	1	1.44E-06	Pu_antagonis	-	-	-
165	2E+07	1	1.44E-06	Pu_antagonis	-	-	-
166	2E+07	1	1.44E-06	Pu_antagonism	ATMAP4KALPHA1	-	-
167	2E+07	1	4.47E-07	Pu_antagonism	AT1G53860	Encodes a protein that is highly methylated in a	biological_process_unknown； (GO:0008150)；
168	6370884	5	1.34E-06	Pu_antagonism	AT5G19050	alpha/beta-Hydrolases	biological_process_unknown； (GO:0008150
169	3648231	5	8.72E-08	Pu_antagonism	AT5G11430	SPOC domain / Transcription	nucleus； (GO:0005634)；
170	3417902	5	8.26E-08	Pu_antagonism	AT5G10800	RNA recognition motif (RRM)-	cytoplasm； (GO:0005737)；
172	3487466	5	8.72E-08	Pu_antagonism	AT5G11020	Protein kinase superfamily protein;(source:Araport11)	protein phosphorylation； (GO:0006468)； phosphorylation； (GO:0016310)；
173	7898106	3	5.14E-07	Pu_antagonism	AT3G22340	transposable_element_gene;(source:Araport11);copia-like	-
174	5150464	4	1.29E-06	Pu_antagonis	KNAT1	homeobox	-
175	7886932	3	1.13E-06	Pu_antagonis	-	-	-
176	2E+07	1	1.50E-06	Pu_antagonis	SRF6	STRUBBELIG-	-
177	2E+07	1	1.50E-06	Pu_antagonis	RPT1A	regulatory particle	-
178	2E+07	1	1.50E-06	Pu_antagonism	AT1G53780	26S proteasome regulatory complex ATPase;(source:Araport11)	cytoplasm； (GO:0005737)； ATPase activity； (GO:0016887)； protein catabolic process； (GO:0030163)；
179	6824998	5	1.98E-06	Pu_antagonism	AT5G20220	zinc knuckle (CCHC-type) family protein;(source:Araport11)	nucleus； (GO:0005634)； nucleic acid binding； (GO:0003676)； mRNA binding； (GO:0003729)； cytoplasm； (

180	6825667	5	1.98E-06	Pu_antagonism	AT5G20220	zinc knuckle (CCHC-type) family protein;(source:Araport11)	nucleus; (GO:0005634); nucleic acid binding; (GO:0003676); mRNA binding; (GO:0003729); cytoplasm; (
181	2E+07	1	3.35E-06	Pu_antagonis	PAP5	purple acid	-
182	1.3E+07	5	1.86E-06	Pu_antagonis	-	-	-
183	2E+07	1	2.11E-06	Pu_antagonism	AT1G53930	Ubiquitin-like superfamily protein;(source:Araport11)	protein ubiquitination; (GO:0016567); nucleus; (GO:0005634); cytoplasm; (GO:0005737);
184	1.5E+07	4	9.70E-07	Pu_antagonis	PKR2	chromatin	-
185	3415727	5	1.58E-06	Pu_antagonism	AT5G10800	RNA recognition motif (RRM)-	cytoplasm; (GO:0005737);
186	2E+07	1	1.33E-06	Pu_antagonism	AT1G53025	Ubiquitin-conjugating enzyme family protein;(source:Araport11)	nucleus; (GO:0005634); postreplication repair; (GO:0006301); protein K63-linked
187	3572047	5	2.55E-06	Pu_antagonis	GLR2.5	glutamate receptor	-
188	3278886	5	4.47E-07	Pu_antagonis	-	-	-
189	1.3E+07	5	7.60E-07	Pu_antagonism	AT5G35180	ENHANCED DISEASE RESISTANCE protein (DUF1336);(sourc	plasma membrane; (GO:0005886); lipid binding; (GO:0008289); nucleus; (
190	9968053	5	4.19E-06	Pu_antagonis	-	-	-
192	3649416	5	1.14E-07	Pu_antagonism	AT5G11430	SPOC domain / Transcription	nucleus; (GO:0005634);
193	2E+07	5	2.83E-06	Pu_antagonism	AT5G50310	Galactose oxidase/kelch	biological_process_unknown; (GO:0008150
194	2.5E+07	5	5.94E-06	Pu_antagonism	RMI1	RMI1 is a component of protein complexes that limit DNA crossover formation via the dissolution of double Holliday	resolution of meiotic recombination intermediates; (GO:0000712); nucleus; (GO:0005634); double-strand break repair via homologous
195	2E+07	1	3.46E-06	Pu_antagonism	AT1G53030	encodes a copper chaperone, can functional complements the	copper chaperone activity; (GO:0016531); cytosol; (GO:0005829); copper
196	3678558	5	3.55E-07	Pu_antagonism	AT5G11500	coiled-coil protein;(source:Araport11)	biological_process_unknown; (GO:0008150); nucleus; (
197	4452230	3	3.22E-06	Pu_antagonis	PUT4	Amino acid	-

198	1.6E+07	3	4.30E-06	Pu_antagonism	DCL3	dicer-like 3	ribonuclease III activity; (GO:0004525); protein binding; (GO:0005515); nucleolus; (GO:0005730); RNA binding; (GO:0003723); nucleus; (GO:0005634); maintenance of DNA methylation; (GO:0010216); nucleic
199	3737307	5	2.13E-07	Pu_antagonism	AT5G11610	Exostosin family protein;(source:Araport11)	transferase activity, transferring glycosyl groups; (GO:0016757); protein
200	3741348	5	2.13E-07	Pu_antagonis	NOXY2	hypothetical	-
201	3744717	5	2.13E-07	Pu_antagonism	AT5G11650	alpha/beta-Hydrolases superfamily protein;(source:Ar	Golgi apparatus; (GO:0005794); lipase activity; (GO:0016298); endoplasmic
202	2E+07	1	2.28E-06	Pu_antagonism	AT1G53120	RNA-binding S4 domain-containing	biological_process_unknown; (GO:0008150
203	3646210	3	7.27E-07	Pu_antagonism	TBL8	TRICHOME BIREFRINGENCE-LIKE 8	Golgi apparatus; (GO:0005794); chloroplast; (
204	6107495	1	1.85E-06	Pu_antagonism	PEPR2	PEP1 receptor 2	protein serine kinase activity; (GO:0106310); peptide binding; (GO:0042277); kinase activity; (GO:0016301); protein binding; (GO:0005515); plasma membrane; (GO:0005886);
205	6111419	1	1.85E-06	Pu_antagonism	CSTF77	Tetratricopeptide repeat (TPR)-like superfamily protein	gene silencing by RNA; (GO:0031047); protein binding; (GO:0005515); nucleus; (GO:0005634); mRNA binding; (GO:0003729); regulation of gene
206	6113415	1	1.85E-06	Pu_antagonism	CSTF77	Tetratricopeptide repeat (TPR)-like superfamily protein	gene silencing by RNA; (GO:0031047); protein binding; (GO:0005515); nucleus; (GO:0005634); mRNA binding; (GO:0003729); regulation of gene

207	6114594	1	1.85E-06	Pu_antagonism	CSTF77	Tetratricopeptide repeat (TPR)-like superfamily protein	gene silencing by RNA; (GO:0031047); protein binding; (GO:0005515); nucleus; (GO:0005634); mRNA binding; (GO:0003729); regulation of gene
208	6118737	1	1.85E-06	Pu_antagonis	-	-	-
209	6120859	1	1.85E-06	Pu_antagonism	SUVH7	histone-lysine N-methyltransferase, H3 lysine-9 specific SUVH3-like protein	nucleus; (GO:0005634); positive regulation of transcription, DNA-templated; (
210	2430191	5	1.77E-06	Pu_antagonism	AT5G07670	RNI-like superfamily protein;(source:Ar	SCF ubiquitin ligase complex; (GO:0019005); SCF-
211	2E+07	1	1.31E-06	Pu_antagonis	-	-	-
212	6779815	5	2.26E-06	Pu_antagonism	NUDX19	nudix hydrolase homolog 19	chloroplast; (GO:0009507); peroxisome; (GO:0005777); cytosol; (GO:0005829); NADH metabolic process; (GO:0006734
213	1.5E+07	4	1.39E-06	Pu_antagonis	UTr7	UDP-N-	-
214	2E+07	5	3.69E-06	Pu_antagonism	ELO3	radical SAM domain-containing protein / GCN5-related N-acetyltransferase (GNAT) family protein	sphingolipid biosynthetic process; (GO:0030148); mitochondrion; (GO:0005739); fatty acid elongation, polyunsaturated fatty acid; (GO:0034626); endoplasmic reticulum; (
215	2.7E+07	1	5.41E-06	Pu_antagonis	-	-	-

216	3683036	5	6.14E-07	Pu_antagonism	MYB3R-4	myb domain protein 3r-4	negative regulation of cellular response to caffeine; (GO:1901181); nucleus; (GO:0005634); protein binding; (GO:0005515); positive regulation of transcription, DNA-templated; (GO:0045893); DNA-binding transcription factor activity, RNA polymerase II-specific; (GO:0000981); DNA-binding transcription
217	3686139	5	6.14E-07	Pu_antagonism	ASP3	-	cytosol; (GO:0005829); L-aspartate:2-oxoglutarate aminotransferase activity; (GO:0004069); glutamate metabolic process; (GO:0006536); plastid; (GO:0009536); nitrogen compound metabolic process; (GO:0006807); leaf
218	3686741	5	6.14E-07	Pu_antagonism	ASP3	-	cytosol; (GO:0005829); L-aspartate:2-oxoglutarate aminotransferase activity; (GO:0004069); glutamate metabolic process; (GO:0006536); plastid; (GO:0009536); nitrogen compound metabolic process; (GO:0006807); leaf

219	3687178	5	6.14E-07	Pu_antagonism	ASP3	-	cytosol; (GO:0005829); L-aspartate:2-oxoglutarate aminotransferase activity; (GO:0004069); glutamate metabolic process; (GO:0006536); plastid; (GO:0009536); nitrogen compound metabolic process; (GO:0006807); leaf
220	3688241	5	6.14E-07	Pu_antagonis	-	-	-
221	1.9E+07	1	2.74E-06	Pu_antagonism	AT1G50140	P-loop containing nucleoside triphosphate hydrolases	cellular_component_unknown; (GO:0005575); ATP binding; (GO:0005524);
222	1.4E+07	5	3.25E-07	Pu_antagonis	-	-	-
223	6785001	1	6.50E-07	Pu_antagonis	CYP722A1	cytochrome P450,	-
224	1.3E+07	4	4.83E-06	Pu_antagonis	ACYB-2	Cytochrome	-
226	3547479	5	1.24E-07	Pu_antagonis	VAMP713	vesicle-associated	-
227	2E+07	1	6.29E-06	Pu_antagonism	SPA4	SPA1-related 4	response to blue light; (GO:0009637); chloroplast; (GO:0009507); protein binding; (GO:0005515); Cul4-RING E3 ubiquitin ligase complex; (
228	1.3E+07	5	4.16E-06	Pu_antagonis	-	-	-
229	2.5E+07	5	2.63E-06	Pu_antagonis	FLS4	flavonol synthase	-
230	9976088	5	8.65E-06	Pu_antagonism	AT5G27944	MADS-box transcription factor family protein;(source:Araport11)	RNA polymerase II cis-regulatory region sequence-specific DNA binding; (GO:0000978); protein dimerization activity; (GO:0046983); DNA-binding transcription factor activity, RNA polymerase II-
231	2.7E+07	1	5.42E-06	Pu_antagonis	AT1G7222	RING/U-box	protein ubiquitination;
232	2.7E+07	1	5.42E-06	Pu_antagonism	AT1G72230	Cupredoxin superfamily protein;(source:Araport11)	electron transfer activity; (GO:0009055); anchored component of membrane; (
233	3692122	5	7.04E-07	Pu_antagonis	-	-	-
234	3549811	5	2.16E-07	Pu_antagonis	-	-	-
235	1.3E+07	5	6.69E-06	Pu_antagonis	-	-	-
236	2E+07	1	4.93E-06	Pu_antagonis	-	-	-

238	2E+07	1	7.07E-06	Pu_antagonism	SPA4	SPA1-related 4	response to blue light; (GO:0009637); chloroplast; (GO:0009507); protein binding; (GO:0005515); Cul4-RING E3 ubiquitin ligase complex; (
239	6800581	5	1.03E-06	Pu_antagonism	HBP5	SOUL heme-binding family protein	chloroplast thylakoid membrane; (GO:0009535); chloroplast; (GO:0009507); cytoplasm; (GO:0005737); protein binding; (
240	3931991	3	8.15E-06	Pu_antagonism	AT3G12350	F-box family protein;(source:Araport11)	chloroplast; (GO:0009507); biological_process_unk
241	6107419	1	1.57E-06	Pu_antagonism	PEPR2	PEP1 receptor 2	protein serine kinase activity; (GO:0106310); peptide binding; (GO:0042277); kinase activity; (GO:0016301); protein binding; (GO:0005515); plasma membrane; (GO:0005886);
242	2E+07	1	7.20E-06	Pu_antagonism	AT1G53705	putative aminoacyl-tRNA ligase;(source:Ara	biological_process_unknown; (GO:0008150);
243	2E+07	1	5.81E-06	Pu_antagonism	NAM	NAC (No Apical Meristem) domain transcriptional regulator superfamily protein	nucleus; (GO:0005634); integument development; (GO:0080060); DNA-binding transcription factor activity; (GO:0003700); regulation of embryonic
245	6123757	1	1.25E-06	Pu_antagonism	AT1G17780	ATG8A/F interacting protein containing a WxxL LIR motif at the C terminus which is essential for interaction with ATG8. Stress (abiotic or biotic)	heat acclimation; (GO:0010286); nucleus; (GO:0005634); phagophore; (GO:0061908); protein binding; (GO:0005515); autophagosome; (
248	7052493	3	7.75E-08	Pu_antagonis	DELTA-	delta vacuolar	-
249	7083996	1	3.76E-07	Pu_antagonis	-	-	-
251	2.6E+07	5	9.93E-06	Pu_antagonis	CHR24	chromatin	-
252	2.5E+07	5	3.31E-06	Pu_antagonis	-	-	-
255	1.5E+07	4	4.24E-06	Pu_antagonism	AT4G31360	selenium binding protein;(source:Ar	nucleus; (GO:0005634); Golgi

256	1.5E+07	4	9.10E-06	Pu_antagonism	AT4G31880	One of 5 PO76/PDS5 cohesion cofactor orthologs of Arabidopsis.	chromatin; (GO:0000785); nucleus; (GO:0005634); mitotic sister chromatid
257	4453610	3	2.42E-06	Pu_antagonis	-	-	-
258	2.5E+07	5	4.26E-06	Pu_antagonism	AT5G63640	ENTH/VHS/GAT family protein;(source:Ar	nucleus; (GO:0005634); plasma membrane; (
259	2.5E+07	5	4.26E-06	Pu_antagonism	AT5G63640	ENTH/VHS/GAT family protein;(source:Ar	nucleus; (GO:0005634); plasma membrane; (
260	6111687	1	6.22E-06	Pu_antagonism	CSTF77	Tetratricopeptide repeat (TPR)-like superfamily protein	gene silencing by RNA; (GO:0031047); protein binding; (GO:0005515); nucleus; (GO:0005634); mRNA binding; (GO:0003729); regulation of gene
262	1E+07	5	5.52E-06	Pu_antagonism	DES1	L-cysteine desulfhydrase 1	cytoplasm; (GO:0005737); chloroplast; (GO:0009507); L-cysteine desulfhydrase activity; (GO:0080146); cysteine homeostasis; (GO:0080145); cystathionine gamma-
263	3704802	5	8.01E-07	Pu_antagonis	GuILO3	-	-
264	944214	1	1.86E-06	Pu_antagonis	-	-	-
269	6131358	1	6.94E-07	Pu_antagonis	BETA-TIP	beta-tonoplast	-
277	5137011	4	5.82E-06	Pu_antagonis	-	-	-
278	2E+07	1	9.41E-06	Pu_antagonism	AT1G53210	Encodes a Na+/Ca 2+ exchanger-like protein that participates in the maintenance of Ca 2+ homeostasis. The mRNA is cell-to-cell mobile.	vacuole; (GO:0005773); calcium:sodium antiporter activity; (GO:0005432); plasmodesma; (GO:0009506); calcium ion binding; (GO:0005509); mRNA binding; (GO:0003729); chloroplast; (
285	6162427	1	7.22E-07	Pu_antagonism	HDG12	homeodomain GLABROUS 12	DNA-binding transcription factor activity, RNA polymerase II-specific; (GO:0000981); DNA-binding transcription

286	1.3E+07	5	4.35E-06	Pu_antagonism	AT5G35205	transposable_element_gene;(source: Araport11);non-LTR	-
290	2.6E+07	5	7.94E-06	Pu_antagonism	AT5G64160	plant/protein;(source:Araport11)	molecular_function_unknown；（GO:0003674）；
292	6612615	5	1.43E-06	Pu_antagonis	-	-	-
293	6785478	5	3.70E-06	Pu_antagonism	AT5G20080	FAD/NAD(P)-binding oxidoreductase;(source:Araport11)	plant-type cell wall；（GO:0009505）；mitochondrion；（GO:0005739）；cytochrome-b5 reductase activity, acting on NAD(P)H；（
296	6168473	1	5.25E-07	Pu_antagonism	AT1G17930	Mobile domain protein involved in silencing of	nucleus；（GO:0005634）；meristem
297	6618416	5	9.06E-06	Pu_antagonis	GNL2	-	-
298	3284053	5	1.41E-06	Pu_antagonism	GRF6	G-box regulating factor 6	nucleus；（GO:0005634）；transcription, DNA-templated；（GO:0006351）；protein binding；（
299	1.6E+07	4	3.46E-06	Pu_antagonism	AT4G32285	Putative clathrin assembly protein, component of TPLATE complex that functions in clathrin-mediated endocytosis.	1-phosphatidylinositol binding；（GO:0005545）；clathrin-coated pit；（GO:0005905）；SNARE binding；（GO:0000149）；plasma membrane；（GO:0005886）；clathrin heavy chain binding；（
300	1.5E+07	4	6.39E-06	Pu_antagonism	AT4G31880	One of 5 PO76/PDS5 cohesion cofactor orthologs of Arabidopsis.	chromatin；（GO:0000785）；nucleus；（GO:0005634）；mitotic sister chromatid
305	3577735	5	7.83E-07	Pu_antagonism	AT5G11220	hypothetical protein;(source:Ar	biological_process_unknown；（GO:0008150

306	7053611	5	7.80E-06	Pu_antagonism	SUS1	sucrose synthase 1	nucleus; (GO:0005634); production of siRNA involved in RNA interference; (GO:0030422); regulation of seed maturation; (GO:2000034); ribonuclease III activity; (GO:0004525); protein binding; (GO:0005515); RNA processing; (GO:0006396); mRNA cleavage involved in gene silencing by miRNA; (GO:0035279); nuclear dicing body; (GO:0010445); production of lsiRNA involved in RNA interference; (
307	1.5E+07	4	7.69E-06	Pu_antagonism	AT4G31880	One of 5 PO76/PDS5 cohesion cofactor orthologs of Arabidopsis.	chromatin; (GO:0000785); nucleus; (GO:0005634); mitotic sister chromatid
308	3689078	5	2.92E-06	Pu_antagonis	-	-	-
309	2E+07	1	2.99E-06	Pu_antagonis	-	-	-
312	6122693	1	3.69E-06	Pu_antagonism	SUVH7	histone-lysine N-methyltransferase, H3 lysine-9 specific SUVH3-like protein	nucleus; (GO:0005634); positive regulation of transcription, DNA-templated; (
313	2E+07	1	1.16E-06	Pu_antagonism	AT1G54920	hypothetical protein;(source:Araport11)	molecular_function_unknown; (GO:0003674);
318	1.5E+07	4	4.87E-06	Pu_antagonism	AT4G30990	ARM repeat superfamily protein;(source:Araport11)	Golgi apparatus; (GO:0005794); nucleolus; (GO:0005730); biological_process_unk
319	6810909	5	3.04E-06	Pu_antagonism	AT5G20170	RNA polymerase II transcription mediator;(source:Araport11)	mediator complex; (GO:0016592); transcription coregulator activity; (GO:0003712); core mediator complex; (GO:0070847);
320	3498140	5	3.18E-07	Pu_antagonis	TRS120	TRS120	-
321	2E+07	1	4.87E-06	Pu_antagonis	AT1G5301	RING/U-box	protein ubiquitination;
326	3614061	5	5.81E-07	Pu_antagonis	-	-	-
327	1.5E+07	4	7.43E-06	Pu_antagonism	AT4G31360	selenium binding protein;(source:Ar	nucleus; (GO:0005634); Golgi

328	6107227	1	3.11E-06	Pu_antagonism	PEPR2	PEP1 receptor 2	protein serine kinase activity; (GO:0106310); peptide binding; (GO:0042277); kinase activity; (GO:0016301); protein binding; (GO:0005515); plasma membrane; (GO:0005886);
330	3575247	5	7.13E-06	Pu_antagonis	-	-	-
334	6571559	5	7.96E-06	Pu_antagonism	AT5G19480	mediator of RNA polymerase II transcription subunit;(source:Araport11)	transcription factor binding; (GO:0008134); mediator complex; (GO:0016592); nucleus; (
335	6572245	5	7.96E-06	Pu_antagonism	AT5G19480	mediator of RNA polymerase II transcription subunit;(source:Araport11)	transcription factor binding; (GO:0008134); mediator complex; (GO:0016592); nucleus; (
336	6572576	5	7.96E-06	Pu_antagonism	AT5G19480	mediator of RNA polymerase II transcription subunit;(source:Araport11)	transcription factor binding; (GO:0008134); mediator complex; (GO:0016592); nucleus; (
337	6572726	5	7.96E-06	Pu_antagonism	AT5G19480	mediator of RNA polymerase II transcription subunit;(source:Araport11)	transcription factor binding; (GO:0008134); mediator complex; (GO:0016592); nucleus; (
338	6573441	5	7.96E-06	Pu_antagonism	AT5G19480	mediator of RNA polymerase II transcription subunit;(source:Araport11)	transcription factor binding; (GO:0008134); mediator complex; (GO:0016592); nucleus; (
339	6586217	5	7.96E-06	Pu_antagonism	MSL9	mechanosensitive channel of small conductance-like 9	mechanosensitive ion channel activity; (GO:0008381); response to karrikin; (GO:0080167); detection of mechanical
341	6172451	1	9.54E-07	Pu_antagonism	AT1G17940	Endosomal targeting BRO1-like domain-	nucleus; (GO:0005634); mitochondrion; (
343	3548045	5	5.39E-07	Pu_antagonis	VAMP713	vesicle-associated	-
351	2E+07	1	6.76E-06	Pu_antagonis	-	-	-
352	6319036	3	1.20E-07	Pu_antagonis	NAC058	NAC domain	-
359	1.5E+07	4	3.76E-06	Pu_antagonism	AT4G30990	ARM repeat superfamily protein;(source:Araport11)	Golgi apparatus; (GO:0005794); nucleolus; (GO:0005730); biological_process_unk

360	6121342	1	8.39E-06	Pu_antagonism	SUVH7	histone-lysine N-methyltransferase, H3 lysine-9 specific SUVH3-like protein	nucleus; (GO:0005634); positive regulation of transcription, DNA-templated; (
369	6110680	1	9.47E-06	Pu_antagonism	CSTF77	Tetratricopeptide repeat (TPR)-like superfamily protein	gene silencing by RNA; (GO:0031047); protein binding; (GO:0005515); nucleus; (GO:0005634); mRNA binding; (GO:0003729); regulation of gene
371	3704279	5	1.61E-06	Pu_antagonis	GuILO3	-	-
375	1.7E+07	5	8.11E-06	Pu_antagonis	GLA1	Folylpolyglutamat	-
376	5108439	4	1.22E-06	Pu_antagonis	-	-	-
379	1.1E+07	3	8.89E-06	Pu_antagonis	-	-	-
381	3769610	5	3.52E-06	Pu_antagonism	AT5G11700	ephrin type-B receptor;(source:Araport11)	plasmodesma; (GO:0009506); extracellular region; (
382	2E+07	2	8.55E-06	Pu_antagonism	SETH6	Phototropic-responsive NPH3 family protein	nucleus; (GO:0005634); protein ubiquitination; (GO:0016567); plasma
386	3729221	5	1.02E-06	Pu_antagonis	-	-	-
389	1344131	5	1.65E-06	Pu_antagonis	-	-	-
391	613022	5	1.64E-06	Pu_antagonism	AT5G02710	zinc/iron-chelating domain protein;(source:Ar	molecular_function_unknown; (GO:0003674);
392	3459373	5	3.44E-06	Pu_antagonism	AT5G10950	Tudor/PWWP/MBT superfamily protein;(source:Araport11)	nucleus; (GO:0005634); DNA repair; (GO:0006281); mitotic sister chromatid cohesion; (GO:0007064);
397	2E+07	1	9.59E-07	Pu_antagonis	-	-	-
400	3483194	5	1.39E-06	Pu_antagonis	-	-	-
404	3477147	5	2.17E-06	Pu_antagonism	AT5G10990	SAUR-like auxin-responsive protein family;(source:Ara	response to auxin; (GO:0009733); molecular_function_un
405	6572794	5	6.89E-06	Pu_antagonism	AT5G19480	mediator of RNA polymerase II transcription subunit;(source:Araport11)	transcription factor binding; (GO:0008134); mediator complex; (GO:0016592); nucleus; (
406	5215287	4	6.65E-06	Pu_antagonism	AT4G08264	transposable_element_gene;(source:Araport11);pseudo	0
415	667064	5	2.94E-07	Pu_antagonism	UPL4	ubiquitin-protein ligase 4	nucleus; (GO:0005634); ubiquitin-protein transferase activity; (GO:0004842); protein

423	2E+07	2	7.95E-06	Pu_antagonism	AT2G47610	Ribosomal protein L7Ae/L30e/S12e/Gadd45 family protein;(source:Araport11)	cytoplasm; (GO:0005737); cytosolic ribosome; (GO:0022626); maturation of LSU-rRNA; (GO:0000470); nucleolus; (GO:0005730); cytosolic large
424	3728453	5	2.35E-06	Pu_antagonism	TINY2	Integrase-type DNA-binding superfamily protein	nucleus; (GO:0005634); positive regulation of transcription, DNA-templated; (
425	3521242	5	7.18E-07	Pu_antagonis	-	-	-
428	6108971	1	4.85E-06	Pu_antagonism	PEPR2	PEP1 receptor 2	protein serine kinase activity; (GO:0106310); peptide binding; (GO:0042277); kinase activity; (GO:0016301); protein binding; (GO:0005515); plasma membrane; (GO:0005886);
430	3666881	5	1.12E-06	Pu_antagonism	AT5G11470	SG1 is a Bromo-Adjacent Homology (BAH) domain containing protein involved in CHG methylation within genebodies. Loss of function results in	regulation of histone methylation; (GO:0031060); nucleus; (GO:0005634); negative regulation of chromatin silencing; (GO:0031936); chromatin binding; (
434	6114974	1	2.55E-06	Pu_antagonism	CSTF77	Tetratricopeptide repeat (TPR)-like superfamily protein	gene silencing by RNA; (GO:0031047); protein binding; (GO:0005515); nucleus; (GO:0005634); mRNA binding; (GO:0003729); regulation of gene
435	1.7E+07	3	6.87E-06	Pu_antagonism	AT3G45775	transposable_element_gene;(source:Araport11);copia-like	-
440	6381285	5	9.65E-06	Pu_antagonis	-	-	-
442	3720137	5	2.45E-06	Pu_antagonism	AT5G11580	Regulator of chromosome condensation	cytosol; (GO:0005829); Golgi apparatus; (GO:0005794); plasma
443	3455417	5	8.78E-06	Pu_antagonism	AT5G10946	hypothetical protein;(source:Ar	biological_process_unknown; (GO:0008150
444	3455923	5	8.78E-06	Pu_antagonism	AT5G10946	hypothetical protein;(source:Ar	biological_process_unknown; (GO:0008150
446	3764090	5	8.44E-06	Pu_antagonism	AT5G11700	ephrin type-B receptor;(source:Araport11)	plasmodesma; (GO:0009506); extracellular region; (

448	1.4E+07	5	4.42E-06	Pu_antagonism	AT5G35380	kinase with adenine nucleotide alpha hydrolases-like domain-containing protein;(source:Ar	kinase activity; (GO:0016301); phosphorylation; (GO:0016310); protein phosphorylation; (GO:0006468); ATP
449	3787697	5	5.42E-06	Pu_antagonism	AT5G11750	Ribosomal protein L19 family protein;(source:Ar	chloroplast; (GO:0009507); structural constituent of
452	4859052	1	8.75E-06	Pu_antagonism	AT1G14220	Ribonuclease T2 family protein;(source:Araport11)	extracellular region; (GO:0005576); endoribonuclease activity; (GO:0004521); RNA catabolic
453	3744415	5	3.57E-06	Pu_antagonis	-	-	-
454	3502475	5	2.44E-06	Pu_antagonis	MYB64	myb domain	-
466	3774465	5	2.01E-06	Pu_antagonism	AT5G11710	EPSIN1 plays an important role in the vacuolar trafficking of soluble proteins at the trans-Golgi network via its interaction with gamma-ADR, VTI11, VSR1, and clathrin. Associated with actin filaments and	clathrin vesicle coat; (GO:0030125); plasma membrane; (GO:0005886); thylakoid; (GO:0009579); endosome; (GO:0005768); protein targeting to vacuole; (GO:0006623); protein binding; (GO:0005515); endocytosis; (
472	9891509	3	2.06E-06	Pu_antagonis	-	-	-
481	3741193	5	5.71E-06	Pu_antagonis	NOXY2	hypothetical	-
485	6124241	1	8.15E-06	Pu_antagonism	AT1G17780	ATG8A/F interacting protein containing a WxxL LIR motif at the C terminus which is essential for interaction with ATG8. Stress (abiotic or biotic)	heat acclimation; (GO:0010286); nucleus; (GO:0005634); phagophore; (GO:0061908); protein binding; (GO:0005515); autophagosome; (
488	2E+07	1	6.02E-06	Pu_antagonism	AT1G54920	hypothetical protein;(source:Araport11)	molecular_function_unknown; (GO:0003674);
689	2432431	4	7.11E-07	Pu_antagonism	MED21	This gene encodes a member of the mediator complex subunit 21 family	nucleus; (GO:0005634); regulation of transcription by RNA polymerase II; (GO:0006357); mediator complex; (GO:0016592); defense response to
691	578069	5	2.20E-06	Pu_antagonis	-	-	-
692	579375	5	1.33E-06	Pu_antagonis	-	-	-
694	623749	5	3.45E-06	Pu_antagonis	-	-	-

703	668716	5	2.37E-06	Pu_antagonism	UPL4	ubiquitin-protein ligase 4	nucleus；（ GO:0005634 ）； ubiquitin-protein transferase activity；（ GO:0004842 ）； protein
747	3520545	5	8.87E-07	Pu_antagonis	-	-	-
748	3784115	5	1.08E-06	Pu_antagonis	AGP15	arabinogalactan	-
749	3480589	5	1.34E-06	Pu_antagonism	AT5G11000	hypothetical protein (DUF868);(source: Araport11)	chloroplast；（ GO:0009507 ）； biological_process_unknown；（ GO:0008150
750	3480966	5	1.34E-06	Pu_antagonis	-	-	-
751	4897274	1	1.49E-06	Pu_antagonis	-	-	-
752	3480514	5	1.59E-06	Pu_antagonism	AT5G11000	hypothetical protein (DUF868);(source: Araport11)	chloroplast；（ GO:0009507 ）； biological_process_unknown；（ GO:0008150
753	3542532	5	1.63E-06	Pu_antagonism	AT5G11130	Exostosin family protein;(source:Araport11)	transferase activity, transferring glycosyl groups；（ GO:0016757
754	3539485	5	1.79E-06	Pu_antagonism	SPS2F	sucrose phosphate synthase 2F	plasma membrane；（ GO:0005886 ）； sucrose-phosphate synthase activity；（ GO:0046524 ）； sucrose biosynthetic process；（ GO:0005986 ）； nectar
755	3540289	5	1.79E-06	Pu_antagonism	SPS2F	sucrose phosphate synthase 2F	plasma membrane；（ GO:0005886 ）； sucrose-phosphate synthase activity；（ GO:0046524 ）； sucrose biosynthetic process；（ GO:0005986 ）； nectar
756	3314724	3	1.90E-06	Pu_antagonis	CAT7	cationic amino	-
757	4652942	1	2.41E-06	Pu_antagonis	-	-	-
758	3521710	5	2.78E-06	Pu_antagonis	-	-	-
759	3481010	5	3.65E-06	Pu_antagonis	-	-	-
760	5205358	4	4.20E-06	Pu_antagonism	AT4G08262	transposable_element_gene;(source: Araport11);non-LTR	-
761	3774046	5	4.30E-06	Pu_antagonism	AT5G11710	EPSIN1 plays an important role in the vacuolar trafficking of soluble proteins at the trans-Golgi network via its interaction with gamma-ADR, VTI11, VSR1, and clathrin. Associated with actin filaments and	clathrin vesicle coat；（ GO:0030125 ）； plasma membrane；（ GO:0005886 ）； thylakoid；（ GO:0009579 ）； endosome；（ GO:0005768 ）； protein targeting to vacuole；（ GO:0006623 ）； protein binding；（ GO:0005515 ）； endocytosis；（
762	1.7E+07	5	4.72E-06	Pu_antagonis	AT5542770	-	-

763	2.3E+07	1	4.86E-06	Pu_antagonism	CuAO1	Copper amine oxidase family protein	primary amine oxidase activity; (GO:0008131); copper ion binding; (GO:0005507); amine metabolic process; (GO:0009308); aliphatic-amine oxidase activity; (GO:0052595); tryptamine:oxygen oxidoreductase (deaminating) activity; (GO:0052593
764	1.7E+07	5	4.91E-06	Pu_antagonism	AT5G41730	Protein kinase family protein;(source:Araport11)	protein kinase activity; (GO:0004672); ATP binding; (GO:0005524); signal transduction; (GO:0007165); protein
765	2E+07	5	5.53E-06	Pu_antagonism	AT5G48650	Negative regulator of defense response to Pseudomonas syringae pv. tomato through	cytosol; (GO:0005829); cytoplasm; (GO:0005737); ribonucleoprotein complex; (GO:1990904);
766	1.8E+07	1	5.89E-06	Pu_antagonism	AT1G48950	C3HC zinc finger-like protein;(source:Ar	nucleus; (GO:0005634); zinc ion binding; (
767	666448	5	6.04E-06	Pu_antagonism	UPL4	ubiquitin-protein ligase 4	nucleus; (GO:0005634); ubiquitin-protein transferase activity; (GO:0004842); protein
768	3528851	5	6.14E-06	Pu_antagonis	-	-	-
769	3537533	5	6.31E-06	Pu_antagonism	SPS2F	sucrose phosphate synthase 2F	plasma membrane; (GO:0005886); sucrose-phosphate synthase activity; (GO:0046524); sucrose biosynthetic process; (GO:0005986); nectar
770	2445725	4	6.49E-06	Pu_antagonis	-	-	-
771	1.3E+07	1	6.88E-06	Pu_antagonism	AT1G35770	transposable_element_gene;(source:Araport11);similar	-

772	1.3E+07	2	7.07E-06	Pu_antagonism	ABCG31	pleiotropic drug resistance 3	transmembrane transport；(GO:0055085)；pollen wall assembly；(GO:0010208)；plasma membrane；(GO:0005886)；plasmodesma；(GO:0009506)；export from cell；(GO:0140352)；efflux transmembrane
773	3717904	5	7.23E-06	Pu_antagonism	AT5G11570	Major facilitator superfamily protein;(source:Ar	transmembrane transporter activity；(GO:0022857)；plasma
774	1204976	1	7.63E-06	Pu_antagonis	CKL13	casein kinase like	-
775	1.9E+07	2	7.93E-06	Pu_antagonism	AT2G47500	P-loop nucleoside triphosphate hydrolases superfamily	microtubule binding；(GO:0008017)；microtubule motor activity；(GO:0003777
776	1.7E+07	5	8.08E-06	Pu_antagonis	-	-	-
777	6149563	1	8.53E-06	Pu_antagonism	AT1G17860	Member of Kunitz trypsin inhibitor (KTI) family involved in plant defense response against spider mites.	cell wall；(GO:0005618)；apoplast；(GO:0048046)；negative regulation of peptidase activity；(GO:0010466)；response to insect；(
778	3789329	5	8.66E-06	Pu_antagonism	AT5G11760	stress response protein;(source:Araport11)	biological_process_unknown；(GO:0008150)；
779	6154171	1	9.27E-06	Pu_antagonis	-	-	-
780	6161730	1	9.46E-06	Pu_antagonis	-	-	-
781	6378690	5	9.48E-06	Pu_antagonis	LUL3	RING/U-box	-
10	7746139	1	3.03E-07	Pu_mutualis	-	-	-
22	7747171	1	6.90E-07	Pu_mutualis	-	-	-
23	7749852	1	6.90E-07	Pu_mutualism	AT1G22010	hypothetical protein;(source:Araport11)	molecular_function_unknown；(GO:0003674)；
497	8900611	2	6.90E-07	Pu_mutualism	AT2G20635	protein kinase and Mad3-BUB1-I domain-containing protein;(source:Araport11)	protein serine kinase activity；(GO:0106310)；protein phosphorylation；(GO:0006468)；protein threonine kinase activity；(GO:0106311)；protein kinase activity；(GO:0004672
498	3943227	1	6.90E-07	Pu_mutualis	AT1G0486	None;(source:Arap	-
499	1783257	4	9.97E-06	Pu_mutualism	AT4G03824	transposable_element_gene;(source:Araport11);Mariner-like transposase	-
504	464186	2	7.83E-06	Pu_mutualis	-	-	-

505	2903965	4	5.12E-08	Pu_mutualism	AT4G05594	transposable_element_gene;(source:Araport11);copia-like	-
506	2.8E+07	1	7.83E-06	Pu_mutualis	-	-	-
511	1140210	5	5.45E-06	Pu_mutualis	-	-	-
513	1.9E+07	1	3.33E-09	Pu_mutualism	VIIIA	P-loop containing nucleoside triphosphate hydrolases superfamily protein	microfilament motor activity; (GO:0000146); vesicle; (GO:0031982); motor activity; (GO:0003774); actin cytoskeleton; (GO:0015629); plasma membrane; (GO:0005886); actin filament-based
514	1.7E+07	4	4.58E-08	Pu_mutualism	MLH3	This gene is a member of the MutL-homolog (MLH) family of DNA mismatch repair (MMR) genes. MLH genes	nucleus; (GO:0005634); mismatch repair complex; (GO:0032300); reciprocal meiotic recombination; (
515	1.7E+07	4	4.58E-08	Pu_mutualism	MLH3	This gene is a member of the MutL-homolog (MLH) family of DNA mismatch repair (MMR) genes. MLH genes	nucleus; (GO:0005634); mismatch repair complex; (GO:0032300); reciprocal meiotic recombination; (
517	1783331	4	3.42E-06	Pu_mutualism	AT4G03824	transposable_element_gene;(source:Araport11);Mariner-like transposase	-
518	2.1E+07	5	3.42E-06	Pu_mutualism	ABCG8	The protein encoded by this	-
519	2.5E+07	5	2.11E-07	Pu_mutualism	ABCA12	The membrane-associated protein	-
520	1640779	4	3.87E-07	Pu_mutualis	AT4G0466	None;(source:Arap	-
521	3386231	3	8.83E-06	Pu_mutualism	AT3G10815	RING/U-box superfamily protein;(source:Ar	ubiquitin protein ligase activity; (GO:0061630); mitochondrion; (
522	1652367	4	3.03E-07	Pu_mutualism	AT4G03728	hypothetical protein;(source:Araport11)	molecular_function_unknown; (GO:0003674);
523	2817901	4	3.03E-07	Pu_mutualism	IBR1	indole-3-butyric acid response 1	peroxisome; (GO:0005777); indolebutyric acid metabolic process; (GO:0080024); chloroplast; (
524	2803877	4	6.98E-08	Pu_mutualism	AT4G05510	transposable_element_gene;(source:Araport11);hAT-like transposase	-

532	1.8E+07	5	2.17E-07	Pu_mutualism	AT5G44010	fanconi anemia group F protein (FANCF);(source: Araport11)	cellular response to DNA damage stimulus; (GO:0006974); Fanconi anaemia nuclear complex; (
536	2.4E+07	1	5.31E-06	Pu_mutualism	AT1G65200	Ubiquitin carboxyl-terminal	nucleus; (GO:0005634)
677	1.7E+07	4	2.19E-06	Pu_mutualism	AT4G36190	Serine carboxypeptidase S28 family protein;(source:Araport11)	proteolysis; (GO:0006508); serine-type peptidase activity; (GO:0008236); vacuole; (
718	2853130	4	4.68E-06	Pu_mutualism	AT4G05582	transposable_element_gene;(source: Araport11);gypsy-like	-
725	1.7E+07	4	2.85E-06	Pu_mutualism	NTRB	NADPH-dependent thioredoxin reductase B	pollen germination; (GO:0009846); positive regulation of cell division; (GO:0051781); cell redox homeostasis; (GO:0045454); mitochondrion; (GO:0005739); removal of superoxide radicals; (GO:0019430);
730	9877773	5	4.91E-06	Pu_mutualism	AT5G27860	hypothetical protein;(source:Ar	molecular_function_unknown; (GO:0003674
736	1.7E+07	4	5.46E-06	Pu_mutualism	AT4G35410	Clathrin adaptor complex small chain family protein;(source:Araport11)	intracellular protein transport; (GO:0006886); mitochondrion; (GO:0005739);
744	1.7E+07	4	9.97E-06	Pu_mutualism	DRL1	KTI12-like, chromatin associated protein	purine nucleotide binding; (GO:0017076); calmodulin binding; (GO:0005516); tRNA modification; (GO:0006400); regulation of transcription by RNA polymerase II; (
782	1.9E+07	1	4.28E-06	Pu_mutualism	AT1G51175	transposable_element_gene;(source: Araport11);gypsy-like	-
783	1.2E+07	5	5.45E-06	Pu_mutualis	AT1G5033	Zinc knuckle	-
784	1.9E+07	1	9.52E-06	Pu_mutualis	AT1G5033	pseudogene of	-

Supplementary Table 5 SNP-based heritability estimates

Number	Chromosome	Positions	additive effect	Phenotype	Var(P)	Heritability
7	4	4865340	0.09369188	Bu_altruism	0.03429377	0.08723791
3	4	5633733	0.115268507	Bu_altruism	0.03429377	0.048464948
4	4	12908721	0.17553581	Bu_altruism	0.03429377	0.094782217
8	5	14259730	0.122685618	Bu_altruism	0.03429377	0.10547675
6	5	24313179	0.076085861	Bu_altruism	0.03429377	0.027480473
1	5	24320640	0.083459303	Bu_altruism	0.03429377	0.033064792
5	5	24332306	-0.00561357	Bu_altruism	0.03429377	0.000157961
9	3	5456536	-0.35424081	Bu_antagonism	0.06820969	0.212215455
14	1	4036563	0.010739803	Bu_mutualism	0.001718784	0.013898619
10	1	7746139	0.018577801	Bu_mutualism	0.001718784	0.025118226
22	1	7747171	-0.013883263	Bu_mutualism	0.001718784	0.011829665
31	1	8152846	0.028294737	Bu_mutualism	0.001718784	0.067162358
26	1	9405110	0.01363703	Bu_mutualism	0.001718784	0.021476789
32	1	13331256	0.004745048	Bu_mutualism	0.001718784	0.003556912
19	1	15979224	0.007810181	Bu_mutualism	0.001718784	0.006100828
21	1	16680413	0.00594834	Bu_mutualism	0.001718784	0.003723853
34	1	22264564	-0.005508963	Bu_mutualism	0.001718784	0.003194043
18	1	22264603	0.017438211	Bu_mutualism	0.001718784	0.025510471
25	2	3688388	0.014901528	Bu_mutualism	0.001718784	0.024515334
13	2	8289541	0.008312033	Bu_mutualism	0.001718784	0.006172374
16	2	8977674	-0.00232582	Bu_mutualism	0.001718784	0.000363042
33	3	7949361	0.003602059	Bu_mutualism	0.001718784	0.001563438
15	3	9696393	0.024608371	Bu_mutualism	0.001718784	0.057355616
20	3	14942387	0.010808832	Bu_mutualism	0.001718784	0.016335066
30	3	20264683	0.014538323	Bu_mutualism	0.001718784	0.021139524
29	4	6221857	0.022904926	Bu_mutualism	0.001718784	0.035209705
28	4	6358289	0.006315022	Bu_mutualism	0.001718784	0.006123325
17	4	7009363	0.021154441	Bu_mutualism	0.001718784	0.058312032
11	4	13613205	0.020565896	Bu_mutualism	0.001718784	0.037786182
24	5	15253322	0.013812904	Bu_mutualism	0.001718784	0.011710066
12	5	17283382	0.019662018	Bu_mutualism	0.001718784	0.03453774
38	3	5872184	-0.0908933	Con_aggression	0.03696607	0.051895217
35	3	5880477	0.243602272	Con_aggression	0.03696607	0.359530514
37	4	6983342	0.100886914	Con_aggression	0.03696607	0.128177769
36	5	14256614	0.151518639	Con_aggression	0.03696607	0.182356012
7	4	4865340	-0.086306875	Con_altruism	0.04186007	0.060646733
3	4	5633733	-0.143295007	Con_altruism	0.04186007	0.061359783
4	4	12908721	-0.224950742	Con_altruism	0.04186007	0.127522128
39	4	17388267	-0.072044155	Con_altruism	0.04186007	0.04303247
6	5	24313179	-0.088391617	Con_altruism	0.04186007	0.030384614
1	5	24320640	-0.054773492	Con_altruism	0.04186007	0.011667355
54	1	5520641	0.008651046	Con_mutualism	0.01217187	0.000886576
45	1	5543599	0.034904417	Con_mutualism	0.01217187	0.012520599
50	1	5545724	0.004350263	Con_mutualism	0.01217187	0.000209435
64	1	6001018	0.053769681	Con_mutualism	0.01217187	0.036473434
56	1	11849661	0.073340602	Con_mutualism	0.01217187	0.063718795
67	1	13389323	0.056127892	Con_mutualism	0.01217187	0.039742868
47	1	18307034	0.022596756	Con_mutualism	0.01217187	0.005247549
44	1	18685098	0.047814678	Con_mutualism	0.01217187	0.025301166
63	1	28201527	0.029076117	Con_mutualism	0.01217187	0.020888451
48	2	358189	-0.012156968	Con_mutualism	0.01217187	0.00151885
51	2	358617	-0.027515981	Con_mutualism	0.01217187	0.00837892
60	2	1602807	0.044427895	Con_mutualism	0.01217187	0.017106644
59	2	7837282	0.014326714	Con_mutualism	0.01217187	0.001778879
55	2	17343383	0.010520028	Con_mutualism	0.01217187	0.001311029
68	3	1475536	0.03301324	Con_mutualism	0.01217187	0.016197251

43	3	20539531	0.029218834	Con_mutualism	0.01217187	0.009448086
69	4	1406662	0.046940498	Con_mutualism	0.01217187	0.024384477
46	4	2866886	0.068851349	Con_mutualism	0.01217187	0.048717978
52	4	6522489	0.005660387	Con_mutualism	0.01217187	0.000354577
62	4	9293684	0.04157938	Con_mutualism	0.01217187	0.035268536
58	4	14976741	-0.003404286	Con_mutualism	0.01217187	0.00010044
65	4	15014918	0.021373804	Con_mutualism	0.01217187	0.005763222
66	5	465269	0.014783821	Con_mutualism	0.01217187	0.002757244
53	5	9472315	0.013751706	Con_mutualism	0.01217187	0.001792181
40	5	9967780	0.074577132	Con_mutualism	0.01217187	0.052708414
41	5	13781553	-0.012686775	Con_mutualism	0.01217187	0.002152666
57	5	13811113	0.014007345	Con_mutualism	0.01217187	0.003058805
49	5	13823261	0.032780055	Con_mutualism	0.01217187	0.017523187
61	5	23957423	0.048995659	Con_mutualism	0.01217187	0.039147944
78	1	4348611	0.121313372	Cu_aggression	0.04453399	0.04451446
74	1	4653504	0.05518206	Cu_aggression	0.04453399	0.01856596
71	1	13238872	0.01460342	Cu_aggression	0.04453399	0.000908689
77	1	27119937	0.062061242	Cu_aggression	0.04453399	0.033028034
75	3	263618	0.088459924	Cu_aggression	0.04453399	0.059885005
76	3	2194772	0.148872192	Cu_aggression	0.04453399	0.076417812
79	3	12023277	0.017656194	Cu_aggression	0.04453399	0.001847402
70	3	12742489	0.134346791	Cu_aggression	0.04453399	0.094108719
80	5	10520445	0.010200594	Cu_aggression	0.04453399	0.000879429
72	5	10528368	0.11183681	Cu_aggression	0.04453399	0.097471278
73	5	13626754	0.086505444	Cu_aggression	0.04453399	0.019383008
83	1	25418493	-0.017058671	Cu_altruism	0.03561312	0.003579461
82	1	25421920	-0.051842682	Cu_altruism	0.03561312	0.032758463
81	1	25423641	-0.071554111	Cu_altruism	0.03561312	0.062404769
510	1	3309721	0.028753287	Cu_mutualism	0.001651776	0.052800069
512	1	3314951	0.002782472	Cu_mutualism	0.001651776	0.001163856
502	1	3941461	-0.051416551	Cu_mutualism	0.001651776	0.200205673
498	1	3943227	0.066137215	Cu_mutualism	0.001651776	0.279351684
507	1	3946688	-0.010266675	Cu_mutualism	0.001651776	0.008595751
501	1	3947434	0.004518247	Cu_mutualism	0.001651776	0.001546003
500	1	4382479	0.008219795	Cu_mutualism	0.001651776	0.00511673
529	1	4394432	0.00373207	Cu_mutualism	0.001651776	0.001215862
508	1	4404315	-0.007775534	Cu_mutualism	0.001651776	0.004930429
10	1	7746139	0.011572864	Cu_mutualism	0.001651776	0.010142668
22	1	7747171	-0.01045746	Cu_mutualism	0.001651776	0.006984124
513	1	18656790	0.02508094	Cu_mutualism	0.001651776	0.047638504
536	1	24218879	0.006103662	Cu_mutualism	0.001651776	0.005600393
506	1	28323308	0.009296061	Cu_mutualism	0.001651776	0.008516836
504	2	464186	0.002929047	Cu_mutualism	0.001651776	0.000845538
497	2	8900611	0.012981115	Cu_mutualism	0.001651776	0.010761764
526	2	10451478	0.001597084	Cu_mutualism	0.001651776	0.000265456
521	3	3386231	0.00125405	Cu_mutualism	0.001651776	0.000286332
520	4	1640779	0.011558713	Cu_mutualism	0.001651776	0.021346502
522	4	1652367	0.005360999	Cu_mutualism	0.001651776	0.002176516
499	4	1783257	0.008929295	Cu_mutualism	0.001651776	0.013106791
517	4	1783331	0.003626823	Cu_mutualism	0.001651776	0.001580712
46	4	2866886	0.005273691	Cu_mutualism	0.001651776	0.002106201
505	4	2903965	0.004172122	Cu_mutualism	0.001651776	0.001215595
527	4	8034073	0.009999073	Cu_mutualism	0.001651776	0.010949408
503	4	9551569	0.010920223	Cu_mutualism	0.001651776	0.009724947
509	4	15170120	0.015373398	Cu_mutualism	0.001651776	0.025882779
528	4	15869814	0.009020814	Cu_mutualism	0.001651776	0.00568285
514	4	16867522	0.011139168	Cu_mutualism	0.001651776	0.020397059
534	5	114805	-0.002585436	Cu_mutualism	0.001651776	0.001245338

531	5	557339	0.00733479	Cu_mutualism	0.001651776	0.004696347
511	5	1140210	0.003489494	Cu_mutualism	0.001651776	0.00077765
537	5	12654062	0.01021614	Cu_mutualism	0.001651776	0.020722232
525	5	17648840	0.005658196	Cu_mutualism	0.001651776	0.003331906
532	5	17708344	0.016450791	Cu_mutualism	0.001651776	0.069031947
518	5	21421485	0.005156592	Cu_mutualism	0.001651776	0.003195402
516	5	21525178	0.017411154	Cu_mutualism	0.001651776	0.039568559
519	5	24797899	0.012635847	Cu_mutualism	0.001651776	0.027682475
535	5	26835954	0.015346847	Cu_mutualism	0.001651776	0.029531635
538	5	6405664	-0.158980353	Eu_aggression	0.05924111	0.208467557
624	1	1284986	-0.035861895	Eu_antagonism	0.01657146	0.012633894
834	1	1568258	0.015793079	Eu_antagonism	0.01657146	0.00285608
539	1	1579057	-0.067228645	Eu_antagonism	0.01657146	0.02877124
835	1	1582248	-0.003559547	Eu_antagonism	0.01657146	0.00017754
836	1	10560976	-0.011252506	Eu_antagonism	0.01657146	0.001774208
837	1	11211696	-0.03645274	Eu_antagonism	0.01657146	0.008458829
838	1	20448033	-0.022406291	Eu_antagonism	0.01657146	0.006531688
572	1	22792647	-0.105313703	Eu_antagonism	0.01657146	0.090153868
542	1	23192292	0.043461713	Eu_antagonism	0.01657146	0.012024404
541	1	23192448	-0.004634247	Eu_antagonism	0.01657146	0.000136712
605	1	25663748	-0.044351436	Eu_antagonism	0.01657146	0.018226935
543	1	27275586	-0.052209908	Eu_antagonism	0.01657146	0.017352247
545	1	27290927	0.066399342	Eu_antagonism	0.01657146	0.028065799
839	1	27292065	-0.003470047	Eu_antagonism	0.01657146	0.000162737
840	1	27295967	0.033956098	Eu_antagonism	0.01657146	0.014410369
841	1	27896616	-0.030407875	Eu_antagonism	0.01657146	0.005886024
546	2	7142207	0.02351493	Eu_antagonism	0.01657146	0.003519956
842	2	7391611	-0.016340986	Eu_antagonism	0.01657146	0.003057689
843	2	9165015	-0.023804917	Eu_antagonism	0.01657146	0.003607308
844	2	11782254	-0.028593243	Eu_antagonism	0.01657146	0.009793031
845	2	12436584	-0.028807543	Eu_antagonism	0.01657146	0.011215743
846	3	241756	0.057598362	Eu_antagonism	0.01657146	0.021118844
604	3	296722	-0.032282203	Eu_antagonism	0.01657146	0.009656608
847	3	296892	-0.031521553	Eu_antagonism	0.01657146	0.013922631
540	3	301029	0.005437013	Eu_antagonism	0.01657146	0.000188179
848	3	335460	-0.006706328	Eu_antagonism	0.01657146	0.000562094
849	3	338522	0.032712452	Eu_antagonism	0.01657146	0.014462447
851	3	3103010	-0.010585095	Eu_antagonism	0.01657146	0.001569985
852	3	4710016	-0.032064547	Eu_antagonism	0.01657146	0.013895232
625	3	5084973	-0.055741707	Eu_antagonism	0.01657146	0.030523285
853	3	5111859	0.101894431	Eu_antagonism	0.01657146	0.066092285
854	3	7951936	-0.046598691	Eu_antagonism	0.01657146	0.013822841
855	3	7957545	-0.038179898	Eu_antagonism	0.01657146	0.009279389
856	3	7964502	-0.008962489	Eu_antagonism	0.01657146	0.000511337
857	3	8594541	-0.014692214	Eu_antagonism	0.01657146	0.001374119
858	3	8837646	-0.008267332	Eu_antagonism	0.01657146	0.000889234
859	3	14861304	0.007825924	Eu_antagonism	0.01657146	0.000765439
860	3	15697200	-0.035276146	Eu_antagonism	0.01657146	0.015552557
544	3	16876866	-0.009048776	Eu_antagonism	0.01657146	0.00052123
861	3	22249812	0.017082223	Eu_antagonism	0.01657146	0.003646937
862	4	1157252	-0.029266995	Eu_antagonism	0.01657146	0.011576356
650	4	1497879	-0.003295828	Eu_antagonism	0.01657146	0.000118574
863	4	5752995	-0.031524353	Eu_antagonism	0.01657146	0.012420295
864	4	8919093	-0.104562085	Eu_antagonism	0.01657146	0.136643196
865	4	8919172	0.035007626	Eu_antagonism	0.01657146	0.015944497
603	4	8921069	0.018284634	Eu_antagonism	0.01657146	0.003097924
866	4	8921350	0.045012958	Eu_antagonism	0.01657146	0.026360922
867	4	9093999	0.024230191	Eu_antagonism	0.01657146	0.003737348

870	4	13756122	-0.037392637	Eu_antagonism	0.01657146	0.008900657
606	4	16692715	-0.037941196	Eu_antagonism	0.01657146	0.013338909
871	4	18176157	-0.040853458	Eu_antagonism	0.01657146	0.010624476
872	5	180671	-0.030473173	Eu_antagonism	0.01657146	0.010633399
873	5	4420488	-0.031385334	Eu_antagonism	0.01657146	0.006270517
874	5	5255835	-0.035961265	Eu_antagonism	0.01657146	0.008232273
875	5	6738187	0.027019718	Eu_antagonism	0.01657146	0.010229814
547	5	15894506	-0.033143564	Eu_antagonism	0.01657146	0.006992755
877	5	17144593	-0.016057633	Eu_antagonism	0.01657146	0.002952567
879	5	17536475	-0.032492517	Eu_antagonism	0.01657146	0.006720732
880	5	18038599	-0.004235698	Eu_antagonism	0.01657146	0.000114209
881	5	18982082	-0.023454476	Eu_antagonism	0.01657146	0.007708282
585	5	19854700	-0.005127297	Eu_antagonism	0.01657146	0.000228745
882	5	22855923	-0.033294721	Eu_antagonism	0.01657146	0.007056683
883	5	24052364	-0.035265872	Eu_antagonism	0.01657146	0.017426708
884	5	24061305	0.00573907	Eu_antagonism	0.01657146	0.000461519
571	5	24098652	-0.02993829	Eu_antagonism	0.01657146	0.007285655
687	1	4548634	-0.028549765	Eu_mutualism	0.02141653	0.005126623
688	1	5533737	0.038391586	Eu_mutualism	0.02141653	0.015413433
45	1	5543599	0.045045459	Eu_mutualism	0.02141653	0.011851534
50	1	5545724	-0.006819624	Eu_mutualism	0.02141653	0.000292514
56	1	11849661	0.073208535	Eu_mutualism	0.02141653	0.036083632
47	1	18307034	0.028023227	Eu_mutualism	0.02141653	0.00458679
513	1	18656790	0.091986076	Eu_mutualism	0.02141653	0.049421592
44	1	18685098	0.040647832	Eu_mutualism	0.02141653	0.010392046
682	1	23602378	0.056680974	Eu_mutualism	0.02141653	0.017304195
63	1	28201527	0.033005957	Eu_mutualism	0.02141653	0.015297705
48	2	358189	-0.02639978	Eu_mutualism	0.02141653	0.004070738
51	2	358617	0.006532527	Eu_mutualism	0.02141653	0.000268404
685	3	2126066	0.09354699	Eu_mutualism	0.02141653	0.070242248
684	3	20047228	0.018021003	Eu_mutualism	0.02141653	0.001749181
43	3	20539531	0.031673656	Eu_mutualism	0.02141653	0.006309903
524	4	2803877	0.067282811	Eu_mutualism	0.02141653	0.032457734
46	4	2866886	0.026123726	Eu_mutualism	0.02141653	0.00398605
505	4	2903965	0.042412832	Eu_mutualism	0.02141653	0.00968883
52	4	6522489	0.03272369	Eu_mutualism	0.02141653	0.006735205
62	4	9293684	0.041772608	Eu_mutualism	0.02141653	0.020231253
514	4	16867522	0.030420234	Eu_mutualism	0.02141653	0.011732463
677	4	17126771	0.038792966	Eu_mutualism	0.02141653	0.00878979
53	5	9472315	0.043505505	Eu_mutualism	0.02141653	0.010194483
40	5	9967780	0.088603006	Eu_mutualism	0.02141653	0.042283771
41	5	13781553	0.040303067	Eu_mutualism	0.02141653	0.012346919
57	5	13811113	0.019105295	Eu_mutualism	0.02141653	0.003234118
49	5	13823261	0.045845022	Eu_mutualism	0.02141653	0.019479882
679	5	13865599	-0.051395514	Eu_mutualism	0.02141653	0.020078573
686	5	14013862	0.097538892	Eu_mutualism	0.02141653	0.064053438
678	5	18885768	0.021019299	Eu_mutualism	0.02141653	0.003914577
683	5	20879073	-0.060854572	Eu_mutualism	0.02141653	0.01994634
693	1	955304	0.017335787	Gu_aggression	0.04233263	0.001023641
74	1	4653504	0.03129933	Gu_aggression	0.04233263	0.006283593
700	1	4654714	0.088957167	Gu_aggression	0.04233263	0.081141916
701	1	5102442	0.012888498	Gu_aggression	0.04233263	0.000974357
697	1	26924468	0.112535919	Gu_aggression	0.04233263	0.054116461
698	2	17597020	0.136739436	Gu_aggression	0.04233263	0.10967333
690	3	4845891	-0.037908949	Gu_aggression	0.04233263	0.00358112
704	3	5775050	-0.013509324	Gu_aggression	0.04233263	0.001104392
385	3	11147236	0.03645154	Gu_aggression	0.04233263	0.00422797
705	4	2386376	0.014635545	Gu_aggression	0.04233263	0.001659421

689	4	2432431	0.134447282	Gu_aggression	0.04233263	0.045044259
109	4	5109002	0.055759601	Gu_aggression	0.04233263	0.014578576
695	4	5214077	0.051367666	Gu_aggression	0.04233263	0.024464756
699	4	5216649	0.014737615	Gu_aggression	0.04233263	0.002305234
706	4	5230364	-0.097129525	Gu_aggression	0.04233263	0.040313415
691	5	578069	0.013964298	Gu_aggression	0.04233263	0.0006642
692	5	579375	0.010722182	Gu_aggression	0.04233263	0.000391586
694	5	623749	0.100798246	Gu_aggression	0.04233263	0.032330027
415	5	667064	0.041155404	Gu_aggression	0.04233263	0.004615347
703	5	668716	-0.109381882	Gu_aggression	0.04233263	0.035353888
696	5	690838	-0.054986003	Gu_aggression	0.04233263	0.010298286
96	5	13533337	0.149727511	Gu_aggression	0.04233263	0.066244467
702	5	13599661	0.088700497	Gu_aggression	0.04233263	0.043156253
97	5	13686275	0.054632269	Gu_aggression	0.04233263	0.008132969
94	5	13707592	-0.106025847	Gu_aggression	0.04233263	0.028012953
4	4	12908721	-0.271313567	Gu_altruism	0.04945644	0.157011264
39	4	17388267	-0.126345074	Gu_altruism	0.04945644	0.112019209
8	5	14259730	-0.150700961	Gu_altruism	0.04945644	0.110355467
670	5	26274990	-0.173317891	Gu_antagonism	0.021301	0.242423473
74	1	4653504	0.109908412	Pu_aggression	0.05348857	0.061321683
71	1	13238872	0.039205089	Pu_aggression	0.05348857	0.005452823
77	1	27119937	0.091066651	Pu_aggression	0.05348857	0.059209467
76	3	2194772	0.156044588	Pu_aggression	0.05348857	0.069902946
70	3	12742489	0.171601096	Pu_aggression	0.05348857	0.127833889
72	5	10528368	0.139126546	Pu_aggression	0.05348857	0.125590776
73	5	13626754	0.079438389	Pu_aggression	0.05348857	0.013608986
498	1	3943227	0.035704891	Pu_mutualism	0.005077452	0.026486236
10	1	7746139	0.047733198	Pu_mutualism	0.005077452	0.056132823
22	1	7747171	-0.026596036	Pu_mutualism	0.005077452	0.014695983
784	1	18644664	0.013425996	Pu_mutualism	0.005077452	0.01016704
513	1	18656790	0.05688634	Pu_mutualism	0.005077452	0.079724501
782	1	18968691	0.014146297	Pu_mutualism	0.005077452	0.012664912
536	1	24218879	0.027285787	Pu_mutualism	0.005077452	0.036409596
506	1	28323308	0.003272548	Pu_mutualism	0.005077452	0.000343366
497	2	8900611	0.031190163	Pu_mutualism	0.005077452	0.020211572
521	3	3386231	0.011844358	Pu_mutualism	0.005077452	0.008309367
520	4	1640779	0.028554818	Pu_mutualism	0.005077452	0.042381066
522	4	1652367	0.019015622	Pu_mutualism	0.005077452	0.008908342
499	4	1783257	0.010934602	Pu_mutualism	0.005077452	0.00639401
517	4	1783331	0.008068951	Pu_mutualism	0.005077452	0.002545302
524	4	2803877	0.006366329	Pu_mutualism	0.005077452	0.00122572
523	4	2817901	0.014753772	Pu_mutualism	0.005077452	0.005362681
718	4	2853130	0.002489774	Pu_mutualism	0.005077452	0.000176039
505	4	2903965	0.025758713	Pu_mutualism	0.005077452	0.015073996
736	4	16833858	-0.032394813	Pu_mutualism	0.005077452	0.054546142
744	4	16834561	-0.018272837	Pu_mutualism	0.005077452	0.0178558
725	4	16843896	0.076091254	Pu_mutualism	0.005077452	0.292115729
514	4	16867522	0.011996586	Pu_mutualism	0.005077452	0.007696313
677	4	17126771	0.010334356	Pu_mutualism	0.005077452	0.002631132
511	5	1140210	0.016379045	Pu_mutualism	0.005077452	0.005573679
730	5	9877773	0.020783503	Pu_mutualism	0.005077452	0.019754159
783	5	12263695	-0.016470238	Pu_mutualism	0.005077452	0.005635917
532	5	17708344	0.019756622	Pu_mutualism	0.005077452	0.032389725
518	5	21421485	0.028676828	Pu_mutualism	0.005077452	0.032148999
519	5	24797899	0.009609922	Pu_mutualism	0.005077452	0.005208843

Supplementary Table 6 The phylogenetic signal measurement of network property parameters

Network type	Classification	Network property parameters	Lambda
Mutualism	Bacteria	Con	0.394572
Mutualism	Bacteria	C(u)	6.46E-05
Mutualism	Bacteria	B(u)	0.190491
Mutualism	Bacteria	E(u)	0.999934
Mutualism	Bacteria	G(u)	0.999934
Mutualism	Bacteria	P(u)	0.482628
Mutualism	Fungi	Con	0.999934
Mutualism	Fungi	C(u)	0.999934
Mutualism	Fungi	B(u)	0.308251
Mutualism	Fungi	E(u)	0.294345
Mutualism	Fungi	G(u)	0.999934
Mutualism	Fungi	P(u)	0.576424
Antagonism	Bacteria	Con	7.90E-05
Antagonism	Bacteria	C(u)	0.051374
Antagonism	Bacteria	B(u)	0.12331
Antagonism	Bacteria	E(u)	5.52E-05
Antagonism	Bacteria	G(u)	6.61E-05
Antagonism	Bacteria	P(u)	0.184003
Antagonism	Fungi	Con	6.64E-05
Antagonism	Fungi	C(u)	6.61E-05
Antagonism	Fungi	B(u)	0.233497
Antagonism	Fungi	E(u)	7.21E-05
Antagonism	Fungi	G(u)	6.61E-05
Antagonism	Fungi	P(u)	5.33E-05
Aggression	Bacteria	Con	0.814775
Aggression	Bacteria	C(u)	6.61E-05
Aggression	Bacteria	B(u)	5.33E-05
Aggression	Bacteria	E(u)	6.61E-05
Aggression	Bacteria	G(u)	0.173614
Aggression	Bacteria	P(u)	7.26E-05
Aggression	Fungi	Con	6.61E-05
Aggression	Fungi	C(u)	0.755548
Aggression	Fungi	B(u)	5.69E-05
Aggression	Fungi	E(u)	6.51E-05
Aggression	Fungi	G(u)	5.85E-05
Aggression	Fungi	P(u)	7.44E-05
Altruism	Bacteria	Con	0.090002
Altruism	Bacteria	C(u)	6.00E-05

Altruism	Bacteria	B(u)	6.61E-05
Altruism	Bacteria	E(u)	0.910777
Altruism	Bacteria	G(u)	7.59E-05
Altruism	Bacteria	P(u)	6.61E-05
Altruism	Fungi	Con	0.163389
Altruism	Fungi	C(u)	0.150665
Altruism	Fungi	B(u)	6.61E-05
Altruism	Fungi	E(u)	0.910777
Altruism	Fungi	G(u)	7.73E-05
Altruism	Fungi	P(u)	6.61E-05

Supplementary Table 7 The gene enrichment analysis of Hub QTLs

gene	**gene description**	**GO**	**KEGG**
AT5G35602	hypothetical protein	-	-
TMK3(AT2G01820)	Leucine-rich repeat protein kinase family protein	plasmodesma；(GO:0009506)；plant-type cell wall；(GO:0009505)；plasma membrane；(GO:0005886)；protein phosphorylation；(GO:0006468)；pollen development；(GO:0009555)；protein binding；(GO:0005515)；protein serine kinase activity；(GO:0106310)；protein threonine kinase activity；(GO:0106311)	-
EMB2296（AT2G18020）	Ribosomal protein L2 family	-	*K02938:large subunit ribosomal protein L8e*
AT2G20635	protein kinase and Mad3-BUB1-I domain-containing protein	protein serine kinase activity；(GO:0106310)；protein phosphorylation；(GO:0006468)；protein threonine kinase activity；(GO:0106311)；protein kinase activity；(GO:0004672)；condensed nuclear chromosome kinetochore；(GO:0000778)；meiotic sister chromatid cohesion, centromeric；(GO:0051754)；mitotic spindle assembly checkpoint；(GO:0007094)；cytoplasm；(GO:0005737)	*K02178 checkpoint serine/threonine-protein kinase*

AT4G05594	transposable_element_gene;(source:Araport11);copia-like retrotransposon family, has a	-	-
AT1G11700	senescence regulator (Protein of unknown function, DUF584)	molecular_function_unknown；(GO:0003674)；biological_process_unknown；(GO:0008150)	-
TNPO3（AT1G12930）	Ran effector.	cytoplasm；(GO:0005737)；protein import into nucleus；(GO:0006606)	K15436 transportin-3
SRF1（AT2G20850）	STRUBBELIG-receptor family 1	-	-
ARPC3（AT1G60430）	This gene encodes one of seven subunits of the human Arp2/3 protein complex	-	*K05756 actin related protein 2/3 complex, subunit 3*
AT4G10201	F-box family protein-related	-	-
AT3G22421	F-box/associated interaction domain protein	biological_process_unknown；(GO:0008150)；molecular_function_unknown；(GO:0003674)	-
RAD23A（AT1G16190）	The protein encoded by this gene is one of two human homologs of Saccharomyces cerevisiae Rad23	nucleotide-excision repair；(GO:0006289)；proteasome-mediated ubiquitin-dependent protein catabolic process；(GO:0043161)；damaged DNA binding；(GO:0003684)；polyubiquitin modification-dependent protein binding；(GO:0031593)；proteasome binding；(GO:0070628)；nucleus；(GO:0005634)；nucleoplasm；(GO:0005654)；ubiquitin binding；	*K10839 UV excision repair protein RAD23*

CYP721A1(AT1G75130)	cytochrome P450, family 721, subfamily A, polypeptide 1	-	-
WSS1/WSS1B（AT5G35690）	WT-like growth phenotype mutants of WSS1B do not display hypersensitivities after treatment with DNA-Protein crosslink inducing agents like camptothecin or	nucleus；(GO:0005634)	-
IBR1（AT4G05530）	indole-3-butyric acid response 1	peroxisome；(GO:0005777)；indolebutyric acid metabolic process；(GO:0080024)；chloroplast；(GO:0009507)；cytosol；(GO:0005829)；response to indolebutyric acid；(GO:0080026)；root hair elongation；(GO:0048767)	K11147 dehydrogenase/reductase SDR family member 4
VIIIA（AT1G50360）	P-loop containing nucleoside triphosphate hydrolases superfamily protein	*microfilament motor activity；(GO:0000146)；vesicle；(GO:0031982)；motor activity；(GO:0003774)；actin cytoskeleton；(GO:0015629)；plasma membrane；(GO:0005886)；actin filament-based movement；(GO:0030048)；actin-dependent ATPase activity；(GO:0030898)；cytoplasm；(GO:0005737)；actin filament organization；(GO:0007015)；plasmodesma；(GO:0009506)；cytosol；(GO:0005829)；actin*	-
AT4G36190	Serine carboxypeptidase S28 family protein;(source:Araport11)	proteolysis；(GO:0006508)；serine-type peptidase activity；(GO:0008236)；vacuole；(GO:0005773)；dipeptidyl-peptidase activity；(GO:0008239)；extracellular region；(GO:0005576)	-

AOC4(AT1G13280)	allene oxide cyclase 4	*mitochondrion；(GO:0005739)；jasmonic acid biosynthetic process；(GO:0009695)；chloroplast；(GO:0009507)；allene-oxide cyclase activity；(GO:0046423)；extracellular region；(GO:0005576)；plasma membrane；(GO:0005886*	*K10525 allene oxide cyclase*
AT5G46850	phosphatidylinositol-glycan biosynthesis class X-like protein;(source:Araport11)	nucleus；(GO:0005634)；molecular_function_unknown；(GO:0003674)；GPI anchor biosynthetic process；(GO:0006506)；endoplasmic reticulum membrane；(GO:0005789)	K07541 GPI mannosyltransferase 1 subunit X
AT2G38260	Probably not a pseudogene based on evidence for transcription (RNA-seq) and translation (Ribo-seq) described in PMID:27791167	-	-
NTRB(AT4G35460)	NADPH-dependent thioredoxin reductase B	pollen germination；(GO:0009846)；positive regulation of cell division；(GO:0051781)；cell redox homeostasis；(GO:0045454)；mitochondrion；(GO:0005739)；removal of superoxide radicals；(GO:0019430)；thioredoxin reduction；(GO:0042964)；chloroplast envelope；(GO:0009941)；cytosol；(GO:0005829)；thioredoxin-disulfide reductase activity；(GO:0004791)；cellular oxidant	*K00384 thioredoxin reductase (NADPH)*
AT1G04867	None	-	-
AT4G03824	transposable_element_gene;(source:Araport11);Mariner-like transposase family, has a 1.5e-62 P-value blast match to GB:AAC28384 mariner transposase (Mariner_TC1-element) (Glycine max);(source:TAIR10)	-	-

AT4G35410	Clathrin adaptor complex small chain family protein;(source:Araport11)	intracellular protein transport；(GO:0006886)；mitochondrion；(GO:0005739)；membrane coat；(GO:0030117)；intracellular membrane-bounded organelle；(GO:0043231)；vesicle-mediated transport；(GO:0016192)	K12394 AP-1 complex subunit sigma 1/2
AT1G50330	Zinc knuckle (CCHC-type) family protein;(source:Araport11)	-	-
AT5G35555	hypothetical protein	-	-
UBP22（*AT5G10790*）	ubiquitin-specific protease 22	-	K11366 ubiquitin carboxyl-terminal hydrolase 22/27/51
AT3G12350	F-box family protein	chloroplast；(GO:0009507)；biological_process_unknown；(GO:0008150)；molecular_function_unknown；(GO:0003674)	-
DELTA-VPE（*AT3G20210*）	delta vacuolar processing enzyme	cysteine-type endopeptidase activity；(GO:0004197)；plant-type cell wall；(GO:0009505)；vacuolar protein processing；(GO:0006624)；protein storage vacuole；(GO:0000326)；programmed cell death；(GO:0012501)；extracellular space；(GO:0005615)；proteolysis involved in cellular protein catabolic process；(GO:0051603)；seed	K01369 legumain
DEL2（*AT5G14960*）	DP-E2F-like 2	-	-
MAIN（*AT1G17930*）	Mobile domain protein involved in silencing of transposable elements. Loss of function affects shoot and root meristem maintenance. Interacts and functions with MAIL1 and PP7L in gene	nucleus；(GO:0005634)；meristem development；(GO:0048507)	-

CAP1(AT4G32285)	Putative clathrin assembly protein, component of TPLATE complex that functions in clathrin-mediated endocytosis	1-phosphatidylinositol binding；(GO:0005545)；clathrin-coated pit；(GO:0005905)；SNARE binding；(GO:0000149)；plasma membrane；(GO:0005886)；clathrin heavy chain binding；(GO:0032050)；cytosol；(GO:0005829)；clathrin-coated vesicle；(GO:0030136)；clathrin coat assembly；(GO:0048268)；nucleus；(GO:0005634)；vesicle budding from membrane；(GO:0006900)；clathrin-dependent endocytosis；(GO:0072583)；cell plate；(GO:0009504)；	-
AT4G31360	selenium binding protein	nucleus；(GO:0005634)；Golgi apparatus；(	-
AT4G25610	C2H2-like zinc finger protein	biological_process_unknown；(GO:0008150)；nucleus；(GO:0005634)；extracellular region；(GO:0005576)	-
NAC058(AT3G18400)	NAC domain containing protein 58	-	-
AT5G20660	Zn-dependent exopeptidases superfamily protein	endoplasmic reticulum；(GO:0005783)；chloroplast；(GO:0009507)；proteolysis；(GO:0006508)	-
AT2G34185	hypothetical protein	molecular_function_unknown；(GO:0003674)；mitochondrion；(GO:0005739)；biological_process_unknown；(GO:0008150)	-
AT5G19480	mediator of RNA polymerase II transcription subunit	transcription factor binding；(GO:0008134)；mediator complex；(GO:0016592)；nucleus；(GO:0005634)；transcription coregulator activity；(GO:0003712)；positive regulation of transcription by RNA polymerase II；(GO:0045944)	-
AT4G00320	F-box/RNI-like superfamily protein	biological_process_unknown；(GO:0008150)；molecular_function_unknown；(GO:0003674)	-

UPL4（AT5G02880）	ubiquitin-protein ligase 4	nucleus；(GO:0005634)；ubiquitin-protein transferase activity；(GO:0004842)；protein ubiquitination；(GO:0016567)；ubiquitin ligase complex；(GO:0000151)；cytoplasm；(GO:0005737)	K10590 E3 ubiquitin-protein ligase TRIP12
AT3G13662	Disease resistance-responsive (dirigent-like protein) family	extracellular region；(GO:0005576)	-
PEPR2(AT1G17750)	PEP1 receptor 2	protein serine kinase activity；(GO:0106310)；peptide binding；(GO:0042277)；kinase activity；(GO:0016301)；protein binding；(GO:0005515)；plasma membrane；(GO:0005886)；defense response to bacterium；(GO:0042742)；protein phosphorylation；(GO:0006468)；response to jasmonic acid；(GO:0009753)；protein threonine kinase activity；(GO:0106311)；response to	-
AT5G11750	Ribosomal protein L19 family protein	chloroplast；(GO:0009507)；structural constituent of ribosome；(GO:0003735)；translation；(GO:0006412)	-
Hrd1A(AT3G16090)	RING/U-box superfamily protein	ubiquitin-dependent protein catabolic process；(GO:0006511)；protein binding；(GO:0005515)；ubiquitin protein ligase activity；(GO:0061630)；ubiquitin-dependent ERAD pathway；(GO:0030433)；protein ubiquitination；(GO:0016567)	K10601 E3 ubiquitin-protein ligase synoviolin
AT3G01850	Aldolase-type TIM barrel family protein;(source:Araport11)	racemase and epimerase activity, acting on carbohydrates and derivatives；(GO:0016857)；carbohydrate metabolic process；(GO:0005975)；cytoplasm；(GO:0005737)；cytosol；(GO:0005829)；chloroplast；(GO:0009507)；metal ion binding；(GO:0046872)；ribulose-phosphate 3-epimerase activity；(GO:0004750)；pentose catabolic process；(GO:0019323)；cellular carbohydrate metabolic process；(GO:0044262)；pentose-phosphate shunt, non-	K01783 ribulose-phosphate 3-epimerase

PIN8(AT5G15100)	Auxin efflux carrier family protein	pollen development；(GO:0009555)；transmembrane transport；(GO:0055085)；auxin homeostasis；(GO:0010252)；auxin efflux；(GO:0010315)；endoplasmic reticulum；(GO:0005783)；plasma membrane；(GO:0005886)；auxin polar transport；(GO:0009926)；auxin efflux transmembrane transporter activity；(GO:0010329)；chloroplast；(GO:0009507)	K13947 auxin efflux carrier family protein
CHL1(AT5G40090)	Disease resistance protein (TIR-NBS class);(source:Araport11)	NAD+ nucleotidase, cyclic ADP-ribose generating；(GO:0061809)；signal transduction；(GO:0007165)；ADP binding；(GO:0043531)；NAD(P)+ nucleosidase activity；(GO:0050135)；chloroplast；(GO:0009507)	-
PXMT1(AT1G66700)	S-adenosyl-L-methionine-dependent methyltransferases superfamily protein	nucleus；(GO:0005634)；gene silencing by miRNA；(GO:0035195)；response to light stimulus；(GO:0009416)；regulation of seedling development；(GO:1900140)；response to herbivore；(GO:0080027)；defense response to bacterium；(GO:0042742)；S-adenosylmethionine-dependent methyltransferase activity；(GO:0008757)；regulation of root development；(GO:2000280)；response to molecule of fungal origin；(GO:0002238)；	-
DFL2(AT4G03400)	Auxin-responsive GH3 family protein	nucleus；(GO:0005634)；chloroplast；(GO:0009507)；cytoplasm；(GO:0005737)；response to light stimulus；(GO:0009416)；acid-amino acid ligase activity；(GO:0016881)	K14487 auxin responsive GH3 gene family
AT4G03410	Peroxisomal membrane 22 kDa (Mpv17/PMP22) family protein;(source:Araport11)	molecular_function_unknown；(GO:0003674)；mitochondrion；(GO:0005739)；cytoplasm；(GO:0005737)；biological_process_unknown；(GO:0008150)；integral component of membrane；(GO:0016021)	K13348 protein Mpv17

SS5(AT5G65685)	SS5 is a chloroplast-localized protein that is related to the canonical starch synthases (most closely to SS4), but is catalytically inactive. Arabidopsis ss5 mutants have near-normal total transitory starch contents, but produce fewer and larger starch granules in their chloroplast.	chloroplast；(GO:0009507)；starch synthase activity；(GO:0009011)；starch biosynthetic process；(GO:0019252)；mitochondrion；(GO:0005739)；starch granule initiation；(GO:0062052)；protein binding；(GO:0005515)；carbohydrate binding；(GO:0030246)	-
AT5G11430	SPOC domain / Transcription elongation factor S-II protein;(source:Araport11)	nucleus；(GO:0005634)；transcription, DNA-templated；(GO:0006351)	-
AT1G72230	Cupredoxin superfamily protein	electron transfer activity；(GO:0009055)；anchored component of membrane；(GO:0031225)；anchored component of plasma membrane；(GO:0046658)	-
CSTF77(AT1G17760)	Tetratricopeptide repeat (TPR)-like superfamily protein	gene silencing by RNA；(GO:0031047)；protein binding；(GO:0005515)；nucleus；(GO:0005634)；mRNA binding；(GO:0003729)；regulation of gene silencing；(GO:0060968)；negative regulation of transcription, DNA-templated；(GO:0045892)；antisense RNA metabolic process；(GO:0042868)；RNA 3'-end processing；(GO:0031123)；embryo sac development；(GO:0009553)	K14408 cleavage stimulation factor subunit 3
GA2OX5（AT3G17203）	-	-	-
AT1G12760	Zinc finger, C3HC4 type (RING finger) family protein	protein ubiquitination；(GO:0016567)；ubiquitin-protein transferase activity；(GO:0004842)；nucleus；(GO:0005634)；chloroplast；(GO:0009507)；vacuolar membrane；(	-
AT3G30405	hypothetical protein	-	-

AT5G19097	transposable_element_gene;(source:Araport11);copia-like retrotransposon family, has a 1.4e-284 P-value blast match to GB:CAA72989 open reading frame 1 (Ty1_Copia-element) (Brassica oleracea);(source:TAIR10)	-	-
AT5G35525	PLAC8 family protein	cytoplasm；(GO:0005737)；biological_process_unknown；(GO:0008150)；molecular_function_unknown；(GO:0003674)	-
AT5G35495	hypothetical protein	-	-
AT3G29175	transposable_element_gene;(source:Araport11);Mutator-like transposase family, has a 3.8e-22 P-value blast match to GB:AAA21566 mudrA of transposon=MuDR (MuDr-element) (Zea mays)	-	-
AT1G14800	Nucleic acid-binding, OB-fold-like protein;(source:Araport11)	molecular_function_unknown；(GO:0003674)；biological_process_unknown；(GO:0008150)；cellular_component_unknown；(GO:0005575)	-
AT4G08280	Thioredoxin superfamily protein;(source:Araport11)	molecular_function_unknown；(GO:0003674)；nucleus；(GO:0005634)；chloroplast；(GO:0009507)	-

PDE320(AT3G06960)	pigment defective 320	nucleus；(GO:0005634)；acylglycerol transport；(GO:0034196)；chloroplast envelope；(GO:0009941)；cytosol；(GO:0005829)；chloroplast outer membrane；(GO:0009707)；chloroplast；(GO:0009507)；ER to chloroplast lipid transport；(GO:1990052)；plastid；(GO:0009536)；phosphatidic acid binding；(GO:0070300)；membrane；(GO:0016020)；protein homodimerization activity；(GO:0042803)；endoplasmic reticulum；(GO:0005783)；mitochondrion；(GO:0005739)；vacuolar	-
EGRET(AT5G60470)	C2H2 and C2HC zinc fingers superfamily protein	nucleus；(GO:0005634)；DNA-binding transcription factor activity；(GO:0003700)；regulation of transcription, DNA-templated；(GO:0006355)	-
RGLG5(AT1G67800)	Copine (Calcium-dependent phospholipid-binding protein) family	nucleus；(GO:0005634)；protein binding；(GO:0005515)；extracellular region；(GO:0005576)；positive regulation of abscisic acid-activated signaling pathway；(GO:0009789)；N-terminal protein myristoylation；(GO:0006499)	K16280 E3 ubiquitin-protein ligase RGLG
PUM11(AT4G08840)	pumilio 11	cytoplasm；(GO:0005737)；mRNA binding；(GO:0003729)；posttranscriptional regulation of	-

Supplementary Table 8 Candidate genes identified within ~10kb windows around associated SNPs

Chromosome	Positions	Gene	Chromosome	Positions	Gene	R^2	Phenotype
1	944214	-	1	940984	*SWI2*	0.846878	Cu_antagonism
1	944214	-	1	945833	*RING1B*	0.846878	Cu_antagonism
1	3941461	*AT1G11684*	1	3943227	*AT1G04867*	0.823471	Cu_mutualism
1	3943227	*AT1G04867*	1	3941461	*AT1G11684*	0.823471	Cu_mutualism
1	4404315	*AT1G12930*	1	4405073	*AT1G12930*	0.922978	Cu_mutualism
1	4405073	*AT1G12930*	1	4404315	*AT1G12930*	0.922978	Cu_mutualism
1	5033389	*NHX8*	1	5029841	*AT1G14650*	0.812369	Cu_antagonism
1	5033389	*NHX8*	1	5031898	*AT1G14650*	0.812369	Cu_antagonism
1	5033389	*NHX8*	1	5032608	*NHX8*	0.858386	Cu_antagonism
1	5033389	*NHX8*	1	5033077	*NHX8*	0.858386	Cu_antagonism
1	5033389	*NHX8*	1	5033635	*NHX8*	0.858386	Cu_antagonism
1	5033389	*NHX8*	1	5033914	*NHX8*	0.858386	Cu_antagonism
1	5033389	*NHX8*	1	5034032	*NHX8*	0.812369	Cu_antagonism
1	5033389	*NHX8*	1	5034852	*NHX8*	0.812369	Cu_antagonism
1	5033389	*NHX8*	1	5034867	*NHX8*	0.812369	Cu_antagonism
1	5102442	*AT1G14800*	1	5094806	*RDR1*	0.824596	Gu_aggression
1	5102442	*AT1G14800*	1	5103585	*AT1G14810*	0.867976	Gu_aggression
1	5543599	*RAD23A*	1	5545724	*RAD23A*	0.91755	Con_mutualism
1	5545724	*RAD23A*	1	5543599	*RAD23A*	0.91755	Con_mutualism
1	6107227	*PEPR2*	1	6107419	*PEPR2*	0.941484	Cu_antagonism
1	6107227	*PEPR2*	1	6107495	*PEPR2*	0.883691	Cu_antagonism
1	6107227	*PEPR2*	1	6108407	*PEPR2*	0.826606	Cu_antagonism
1	6107227	*PEPR2*	1	6108654	*PEPR2*	0.826606	Cu_antagonism
1	6107419	*PEPR2*	1	6107227	*PEPR2*	0.941484	Cu_antagonism
1	6107419	*PEPR2*	1	6107495	*PEPR2*	0.938615	Cu_antagonism
1	6107419	*PEPR2*	1	6108407	*PEPR2*	0.877982	Cu_antagonism
1	6107419	*PEPR2*	1	6108654	*PEPR2*	0.877982	Cu_antagonism
1	6107495	*PEPR2*	1	6107227	*PEPR2*	0.883691	Cu_antagonism
1	6107495	*PEPR2*	1	6107419	*PEPR2*	0.938615	Cu_antagonism
1	6107495	*PEPR2*	1	6107897	*PEPR2*	0.810668	Cu_antagonism
1	6107495	*PEPR2*	1	6108407	*PEPR2*	0.935402	Cu_antagonism
1	6107495	*PEPR2*	1	6108654	*PEPR2*	0.935402	Cu_antagonism
1	6107495	*PEPR2*	1	6108971	*PEPR2*	0.834259	Cu_antagonism
1	6107495	*PEPR2*	1	6109521	*PEPR2*	0.938615	Cu_antagonism
1	6108407	*PEPR2*	1	6107227	*PEPR2*	0.826606	Cu_antagonism
1	6108407	*PEPR2*	1	6107419	*PEPR2*	0.877982	Cu_antagonism
1	6108407	*PEPR2*	1	6107495	*PEPR2*	0.935402	Cu_antagonism
1	6108407	*PEPR2*	1	6107897	*PEPR2*	0.867441	Cu_antagonism
1	6108407	*PEPR2*	1	6109521	*PEPR2*	0.877982	Cu_antagonism
1	6108407	*PEPR2*	1	6109751	*PEPR2*	0.935402	Cu_antagonism
1	6108407	*PEPR2*	1	6110680	*CSTF77*	0.826606	Cu_antagonism
1	6108654	*PEPR2*	1	6107227	*PEPR2*	0.826606	Cu_antagonism
1	6108654	*PEPR2*	1	6107419	*PEPR2*	0.877982	Cu_antagonism
1	6108654	*PEPR2*	1	6107495	*PEPR2*	0.935402	Cu_antagonism
1	6108654	*PEPR2*	1	6107897	*PEPR2*	0.867441	Cu_antagonism
1	6108654	*PEPR2*	1	6109521	*PEPR2*	0.877982	Cu_antagonism
1	6108654	*PEPR2*	1	6109751	*PEPR2*	0.935402	Cu_antagonism
1	6108654	*PEPR2*	1	6110680	*CSTF77*	0.826606	Cu_antagonism
1	6108654	*PEPR2*	1	6111419	*CSTF77*	0.935402	Cu_antagonism
1	6108971	*PEPR2*	1	6107495	*PEPR2*	0.834259	Cu_antagonism

1	6108971	*PEPR2*	1	6111419	*CSTF77*	0.834259	Cu_antagonism
1	6109521	*PEPR2*	1	6107495	*PEPR2*	0.938615	Cu_antagonism
1	6109521	*PEPR2*	1	6108407	*PEPR2*	0.877982	Cu_antagonism
1	6109521	*PEPR2*	1	6108654	*PEPR2*	0.877982	Cu_antagonism
1	6109521	*PEPR2*	1	6109751	*PEPR2*	0.819858	Cu_antagonism
1	6109521	*PEPR2*	1	6110680	*CSTF77*	0.828089	Cu_antagonism
1	6109521	*PEPR2*	1	6111419	*CSTF77*	0.938615	Cu_antagonism
1	6109521	*PEPR2*	1	6111687	*CSTF77*	0.877982	Cu_antagonism
1	6109751	*PEPR2*	1	6107897	*PEPR2*	0.810668	Cu_antagonism
1	6109751	*PEPR2*	1	6108407	*PEPR2*	0.935402	Cu_antagonism
1	6109751	*PEPR2*	1	6108654	*PEPR2*	0.935402	Cu_antagonism
1	6109751	*PEPR2*	1	6109521	*PEPR2*	0.819858	Cu_antagonism
1	6109751	*PEPR2*	1	6111419	*CSTF77*	0.874232	Cu_antagonism
1	6109751	*PEPR2*	1	6111687	*CSTF77*	0.810668	Cu_antagonism
1	6109751	*PEPR2*	1	6112031	*CSTF77*	0.810668	Cu_antagonism
1	6109751	*PEPR2*	1	6113415	*CSTF77*	0.874232	Cu_antagonism
1	6110680	*CSTF77*	1	6108407	*PEPR2*	0.826606	Cu_antagonism
1	6110680	*CSTF77*	1	6108654	*PEPR2*	0.826606	Cu_antagonism
1	6110680	*CSTF77*	1	6109521	*PEPR2*	0.828089	Cu_antagonism
1	6110680	*CSTF77*	1	6111419	*CSTF77*	0.883691	Cu_antagonism
1	6110680	*CSTF77*	1	6111687	*CSTF77*	0.826606	Cu_antagonism
1	6110680	*CSTF77*	1	6113415	*CSTF77*	0.883691	Cu_antagonism
1	6110680	*CSTF77*	1	6114594	*CSTF77*	0.883691	Cu_antagonism
1	6111419	*CSTF77*	1	6108654	*PEPR2*	0.935402	Cu_antagonism
1	6111419	*CSTF77*	1	6108971	*PEPR2*	0.834259	Cu_antagonism
1	6111419	*CSTF77*	1	6109521	*PEPR2*	0.938615	Cu_antagonism
1	6111419	*CSTF77*	1	6109751	*PEPR2*	0.874232	Cu_antagonism
1	6111419	*CSTF77*	1	6110680	*CSTF77*	0.883691	Cu_antagonism
1	6111419	*CSTF77*	1	6111687	*CSTF77*	0.935402	Cu_antagonism
1	6111419	*CSTF77*	1	6112031	*CSTF77*	0.810668	Cu_antagonism
1	6111687	*CSTF77*	1	6109521	*PEPR2*	0.877982	Cu_antagonism
1	6111687	*CSTF77*	1	6109751	*PEPR2*	0.810668	Cu_antagonism
1	6111687	*CSTF77*	1	6110680	*CSTF77*	0.826606	Cu_antagonism
1	6111687	*CSTF77*	1	6111419	*CSTF77*	0.935402	Cu_antagonism
1	6111687	*CSTF77*	1	6112031	*CSTF77*	0.867441	Cu_antagonism
1	6111687	*CSTF77*	1	6113415	*CSTF77*	0.935402	Cu_antagonism
1	6111687	*CSTF77*	1	6114594	*CSTF77*	0.935402	Cu_antagonism
1	6111687	*CSTF77*	1	6116096	*CSTF77*	0.867441	Cu_antagonism
1	6113415	*CSTF77*	1	6109751	*PEPR2*	0.874232	Cu_antagonism
1	6113415	*CSTF77*	1	6110680	*CSTF77*	0.883691	Cu_antagonism
1	6113415	*CSTF77*	1	6111687	*CSTF77*	0.935402	Cu_antagonism
1	6113415	*CSTF77*	1	6112031	*CSTF77*	0.810668	Cu_antagonism
1	6113415	*CSTF77*	1	6116096	*CSTF77*	0.935402	Cu_antagonism
1	6113415	*CSTF77*	1	6116591	*CSTF77*	0.819858	Cu_antagonism
1	6114594	*CSTF77*	1	6110680	*CSTF77*	0.883691	Cu_antagonism
1	6114594	*CSTF77*	1	6111687	*CSTF77*	0.935402	Cu_antagonism
1	6114594	*CSTF77*	1	6112031	*CSTF77*	0.810668	Cu_antagonism
1	6114594	*CSTF77*	1	6116096	*CSTF77*	0.935402	Cu_antagonism
1	6114594	*CSTF77*	1	6116591	*CSTF77*	0.819858	Cu_antagonism
1	6114594	*CSTF77*	1	6118371	-	0.871593	Cu_antagonism
1	6116096	*CSTF77*	1	6111687	*CSTF77*	0.867441	Cu_antagonism
1	6116096	*CSTF77*	1	6112031	*CSTF77*	0.867441	Cu_antagonism
1	6116096	*CSTF77*	1	6113415	*CSTF77*	0.935402	Cu_antagonism

1	6116096	*CSTF77*	1	6114594	*CSTF77*	0.935402	Cu_antagonism
1	6116096	*CSTF77*	1	6118371	-	0.931784	Cu_antagonism
1	6116096	*CSTF77*	1	6118737	-	0.935402	Cu_antagonism
1	6116096	*CSTF77*	1	6120859	*SUVH7*	0.935402	Cu_antagonism
1	6118371	-	1	6114594	*CSTF77*	0.871593	Cu_antagonism
1	6118371	-	1	6116096	*CSTF77*	0.931784	Cu_antagonism
1	6118371	-	1	6116591	*CSTF77*	0.818089	Cu_antagonism
1	6118371	-	1	6118737	-	0.871593	Cu_antagonism
1	6118371	-	1	6120859	*SUVH7*	0.871593	Cu_antagonism
1	6118737	-	1	6116096	*CSTF77*	0.935402	Cu_antagonism
1	6118737	-	1	6116591	*CSTF77*	0.819858	Cu_antagonism
1	6118737	-	1	6118371	-	0.871593	Cu_antagonism
1	6118737	-	1	6121342	*SUVH7*	0.883691	Cu_antagonism
1	6120859	*SUVH7*	1	6116096	*CSTF77*	0.935402	Cu_antagonism
1	6120859	*SUVH7*	1	6116591	*CSTF77*	0.819858	Cu_antagonism
1	6120859	*SUVH7*	1	6118371	-	0.871593	Cu_antagonism
1	6120859	*SUVH7*	1	6121342	*SUVH7*	0.883691	Cu_antagonism
1	6120859	*SUVH7*	1	6122117	*SUVH7*	0.871593	Cu_antagonism
1	6121342	*SUVH7*	1	6116591	*CSTF77*	0.828089	Cu_antagonism
1	6121342	*SUVH7*	1	6118737	-	0.883691	Cu_antagonism
1	6121342	*SUVH7*	1	6120859	*SUVH7*	0.883691	Cu_antagonism
1	6121816	*SUVH7*	1	6122042	*SUVH7*	0.858386	Cu_antagonism
1	6121816	*SUVH7*	1	6123757	*AT1G17780*	0.904989	Cu_antagonism
1	6121816	*SUVH7*	1	6124241	*AT1G17780*	0.812472	Cu_antagonism
1	6122042	*SUVH7*	1	6121816	*SUVH7*	0.858386	Cu_antagonism
1	6122042	*SUVH7*	1	6122693	*SUVH7*	0.818734	Cu_antagonism
1	6122042	*SUVH7*	1	6123757	*AT1G17780*	0.948504	Cu_antagonism
1	6122042	*SUVH7*	1	6124241	*AT1G17780*	0.948504	Cu_antagonism
1	6122117	*SUVH7*	1	6120859	*SUVH7*	0.871593	Cu_antagonism
1	6122693	*SUVH7*	1	6122042	*SUVH7*	0.818734	Cu_antagonism
1	6122693	*SUVH7*	1	6123757	*AT1G17780*	0.863185	Cu_antagonism
1	6123757	*AT1G17780*	1	6121816	*SUVH7*	0.904989	Cu_antagonism
1	6123757	*AT1G17780*	1	6122042	*SUVH7*	0.948504	Cu_antagonism
1	6123757	*AT1G17780*	1	6122693	*SUVH7*	0.863185	Cu_antagonism
1	6123757	*AT1G17780*	1	6124241	*AT1G17780*	0.899038	Cu_antagonism
1	6124241	*AT1G17780*	1	6121816	*SUVH7*	0.812472	Cu_antagonism
1	6124241	*AT1G17780*	1	6122042	*SUVH7*	0.948504	Cu_antagonism
1	6124241	*AT1G17780*	1	6123757	*AT1G17780*	0.899038	Cu_antagonism
1	6128210	-	1	6125607	*AT1G17790*	0.839363	Cu_antagonism
1	6128210	-	1	6126993	*AT1G17790*	0.839678	Cu_antagonism
1	6128210	-	1	6127597	-	0.918784	Cu_antagonism
1	6128210	-	1	6127928	-	0.918784	Cu_antagonism
1	6128210	-	1	6129263	*ENODL22*	0.919394	Cu_antagonism
1	6128210	-	1	6129337	*ENODL22*	0.800139	Cu_antagonism
1	6128210	-	1	6131358	*BETA-TIP*	0.839678	Cu_antagonism
1	6129263	*ENODL22*	1	6127597	-	0.839363	Cu_antagonism
1	6129263	*ENODL22*	1	6127928	-	0.839363	Cu_antagonism
1	6129263	*ENODL22*	1	6128210	-	0.919394	Cu_antagonism
1	6131358	*BETA-TIP*	1	6128210	-	0.839678	Cu_antagonism
1	6131950	*AT1G05333*	1	6133032	*AT1G05333*	0.874232	Cu_antagonism
1	6131950	*AT1G05333*	1	6133261	*AT1G05333*	0.874232	Cu_antagonism
1	6131950	*AT1G05333*	1	6133785	*AT1G05333*	0.874232	Cu_antagonism
1	6131950	*AT1G05333*	1	6134140	*AT1G05333*	0.819858	Cu_antagonism

1	6131950	*4T1G05333*	1	6134713	*AT1G05333*	0.883691	Cu_antagonism
1	6134713	*4T1G05333*	1	6131950	*AT1G05333*	0.883691	Cu_antagonism
1	6134713	*4T1G05333*	1	6133261	*AT1G05333*	0.883691	Cu_antagonism
1	6134713	*4T1G05333*	1	6133785	*AT1G05333*	0.883691	Cu_antagonism
1	6134713	*4T1G05333*	1	6134140	*AT1G05333*	0.828089	Cu_antagonism
1	6168473	*4T1G17930*	1	6172451	*AT1G17940*	0.901486	Cu_antagonism
1	6172451	*4T1G17940*	1	6168473	*AT1G17930*	0.901486	Cu_antagonism
1	7746139	-	1	7747171	-	0.823471	Bu_mutualism
1	7747171	-	1	7746139	-	0.823471	Bu_mutualism
1	13891984	-	1	13891142	-	0.846878	Cu_antagonism
1	15958972	*4T1G42515*	1	15959320	*AT1G42515*	0.800139	Cu_antagonism
1	15979273	*PMI1*	1	15979680	*PMI1*	0.91755	Cu_antagonism
1	15979680	*PMI1*	1	15979273	*PMI1*	0.91755	Cu_antagonism
1	19563312	*4T1G52510*	1	19564595	*AT1G52510*	0.922978	Cu_antagonism
1	19563312	*4T1G52510*	1	19566263	*FRS6*	0.922978	Cu_antagonism
1	19564595	*4T1G52510*	1	19563312	*AT1G52510*	0.922978	Cu_antagonism
1	19566263	*FRS6*	1	19563312	*AT1G52510*	0.922978	Cu_antagonism
1	19671812	*CDT1*	1	19673799	-	0.856225	Cu_antagonism
1	19673799	-	1	19671812	*CDT1*	0.856225	Cu_antagonism
1	19690082	*NAM*	1	19691989	-	0.864394	Cu_antagonism
1	19690082	*NAM*	1	19692692	-	0.864394	Cu_antagonism
1	19691989	-	1	19690082	*NAM*	0.864394	Cu_antagonism
1	19692692	-	1	19690082	*NAM*	0.864394	Cu_antagonism
1	19742479	*4T1G52990*	1	19746941	*CKS*	0.88882	Cu_antagonism
1	19757140	*4T1G53025*	1	19758511	*AT1G53025*	0.927677	Cu_antagonism
1	19757140	*4T1G53025*	1	19758968	*AT1G53025*	0.864394	Cu_antagonism
1	19757140	*4T1G53025*	1	19761305	*AT1G53030*	0.927677	Cu_antagonism
1	19758511	*4T1G53025*	1	19757140	*AT1G53025*	0.927677	Cu_antagonism
1	19758511	*4T1G53025*	1	19758718	*AT1G53025*	0.927677	Cu_antagonism
1	19758511	*4T1G53025*	1	19758968	*AT1G53025*	0.800345	Cu_antagonism
1	19758511	*4T1G53025*	1	19759938	-	0.927677	Cu_antagonism
1	19758511	*4T1G53025*	1	19761305	*AT1G53030*	0.859766	Cu_antagonism
1	19758511	*4T1G53025*	1	19762288	*AT1G53035*	0.927677	Cu_antagonism
1	19758718	*4T1G53025*	1	19758511	*AT1G53025*	0.927677	Cu_antagonism
1	19758718	*4T1G53025*	1	19758968	*AT1G53025*	0.864394	Cu_antagonism
1	19758718	*4T1G53025*	1	19761305	*AT1G53030*	0.927677	Cu_antagonism
1	19758968	*4T1G53025*	1	19757140	*AT1G53025*	0.864394	Cu_antagonism
1	19758968	*4T1G53025*	1	19758511	*AT1G53025*	0.800345	Cu_antagonism
1	19758968	*4T1G53025*	1	19758718	*AT1G53025*	0.864394	Cu_antagonism
1	19758968	*4T1G53025*	1	19759938	-	0.864394	Cu_antagonism
1	19758968	*4T1G53025*	1	19761305	*AT1G53030*	0.800345	Cu_antagonism
1	19758968	*4T1G53025*	1	19762288	*AT1G53035*	0.864394	Cu_antagonism
1	19759938	-	1	19758511	*AT1G53025*	0.927677	Cu_antagonism
1	19759938	-	1	19758968	*AT1G53025*	0.864394	Cu_antagonism
1	19759938	-	1	19761305	*AT1G53030*	0.927677	Cu_antagonism
1	19761305	*4T1G53030*	1	19757140	*AT1G53025*	0.927677	Cu_antagonism
1	19761305	*4T1G53030*	1	19758511	*AT1G53025*	0.859766	Cu_antagonism
1	19761305	*4T1G53030*	1	19758718	*AT1G53025*	0.927677	Cu_antagonism
1	19761305	*4T1G53030*	1	19758968	*AT1G53025*	0.800345	Cu_antagonism
1	19761305	*4T1G53030*	1	19759938	-	0.927677	Cu_antagonism
1	19761305	*4T1G53030*	1	19762288	*AT1G53035*	0.927677	Cu_antagonism
1	19761305	*4T1G53030*	1	19767961	-	0.927677	Cu_antagonism
1	19762288	*4T1G53035*	1	19758511	*AT1G53025*	0.927677	Cu_antagonism

1	19762288	*AT1G53035*	1	19758968	*AT1G53025*	0.864394	Cu_antagonism
1	19762288	*AT1G53035*	1	19761305	*AT1G53030*	0.927677	Cu_antagonism
1	19767961	-	1	19761305	*AT1G53030*	0.927677	Cu_antagonism
1	19785868	*SPA4*	1	19786600	*SPA4*	0.859766	Cu_antagonism
1	19785868	*SPA4*	1	19793358	*AT1G53120*	0.859766	Cu_antagonism
1	19786600	*SPA4*	1	19785868	*SPA4*	0.859766	Cu_antagonism
1	19786600	*SPA4*	1	19793358	*AT1G53120*	0.859766	Cu_antagonism
1	19793358	*AT1G53120*	1	19785868	*SPA4*	0.859766	Cu_antagonism
1	19793358	*AT1G53120*	1	19786600	*SPA4*	0.859766	Cu_antagonism
1	19814114	*MAP4K ALP*	1	19818737	*MAP4K ALPH*	0.808556	Cu_antagonism
1	19814114	*MAP4K ALP*	1	19818926	*MAP4K ALPH*	0.808556	Cu_antagonism
1	19818737	*MAP4K ALP*	1	19814114	*MAP4K ALPH*	0.808556	Cu_antagonism
1	19818737	*MAP4K ALP*	1	19816366	*MAP4K ALPH*	0.883691	Cu_antagonism
1	19818737	*MAP4K ALP*	1	19819633	-	0.883691	Cu_antagonism
1	19818926	*MAP4K ALP*	1	19814114	*MAP4K ALPH*	0.808556	Cu_antagonism
1	19818926	*MAP4K ALP*	1	19816366	*MAP4K ALPH*	0.883691	Cu_antagonism
1	19818926	*MAP4K ALP*	1	19819633	-	0.883691	Cu_antagonism
1	19819633	-	1	19818737	*MAP4K ALPH*	0.883691	Cu_antagonism
1	19819633	-	1	19818926	*MAP4K ALPH*	0.883691	Cu_antagonism
1	20092892	-	1	20093118	-	0.839363	Cu_antagonism
1	20093118	-	1	20092892	-	0.839363	Cu_antagonism
1	20471294	*AT1G54920*	1	20471869	*AT1G54920*	0.944062	Cu_antagonism
1	20471869	*AT1G54920*	1	20471294	*AT1G54920*	0.944062	Cu_antagonism
1	25418493	*AT1G67792*	1	25419492	*AT1G67792*	0.901241	Cu_altruism
1	25418493	*AT1G67792*	1	25419532	*AT1G67792*	0.949818	Cu_altruism
1	25418493	*AT1G67792*	1	25419853	*AT1G67792*	0.974926	Cu_altruism
1	25418493	*AT1G67792*	1	25420666	*AT1G67800*	0.927246	Cu_altruism
1	25418493	*AT1G67792*	1	25420969	*AT1G67800*	0.927246	Cu_altruism
1	25420087	*AT1G67792*	1	25419492	*AT1G67792*	0.901241	Cu_altruism
1	25420087	*AT1G67792*	1	25419532	*AT1G67792*	0.949818	Cu_altruism
1	25420087	*AT1G67792*	1	25419853	*AT1G67792*	0.974926	Cu_altruism
1	25420087	*AT1G67792*	1	25420666	*AT1G67800*	0.927246	Cu_altruism
1	25420087	*AT1G67792*	1	25420969	*AT1G67800*	0.927246	Cu_altruism
1	25420087	*AT1G67792*	1	25421920	*AT1G67800*	0.876142	Cu_altruism
1	25421920	*AT1G67800*	1	25419532	*AT1G67792*	0.827301	Cu_altruism
1	25421920	*AT1G67800*	1	25419853	*AT1G67792*	0.853395	Cu_altruism
1	25421920	*AT1G67800*	1	25420087	*AT1G67792*	0.876142	Cu_altruism
1	25421920	*AT1G67800*	1	25420666	*AT1G67800*	0.903789	Cu_altruism
1	25421920	*AT1G67800*	1	25420969	*AT1G67800*	0.903789	Cu_altruism
1	25421920	*AT1G67800*	1	25423211	*AT1G67800*	0.810139	Cu_altruism
1	25421920	*AT1G67800*	1	25423641	*AT1G67800*	0.949181	Cu_altruism
1	25423641	*AT1G67800*	1	25420666	*AT1G67800*	0.856324	Cu_altruism
1	25423641	*AT1G67800*	1	25420969	*AT1G67800*	0.856324	Cu_altruism
1	25423641	*AT1G67800*	1	25421920	*AT1G67800*	0.949181	Cu_altruism
1	27160352	-	1	27160533	-	0.823471	Cu_antagonism
1	27177803	-	1	27177852	-	0.91121	Cu_antagonism
1	27177803	-	1	27178271	-	0.91121	Cu_antagonism
1	27177803	-	1	27180242	*AT1G72210*	0.836081	Cu_antagonism
1	27177852	-	1	27177803	-	0.91121	Cu_antagonism
1	27177852	-	1	27178271	-	0.829336	Cu_antagonism
1	27177852	-	1	27185583	*AT1G72220*	0.829336	Cu_antagonism
1	27178271	-	1	27177803	-	0.91121	Cu_antagonism
1	27178271	-	1	27177852	-	0.829336	Cu_antagonism

1	27178271	-	1	27185583	AT1G72220	0.829336	Cu_antagonism
1	27180242	AT1G72210	1	27177803	-	0.836081	Cu_antagonism
1	27185583	AT1G72220	1	27177852	-	0.829336	Cu_antagonism
1	27185583	AT1G72220	1	27178271	-	0.829336	Cu_antagonism
1	27290927	AT1G72490	1	27288181	AT1G72480	0.823471	Eu_antagonism
1	27292065	#N/A	1	27295967	AT1G72500	0.805663	Eu_antagonism
1	27295967	#N/A	1	27292065	-	0.805663	Eu_antagonism
2	358189	AT2G01820	2	358617	AT2G01820	0.91755	Con_mutualism
2	358617	AT2G01820	2	358189	AT2G01820	0.91755	Con_mutualism
2	8977674	SRF1	2	8975776	SRF1	0.903712	Bu_mutualism
2	8977674	SRF1	2	8976007	SRF1	0.903712	Bu_mutualism
2	8977674	SRF1	2	8976392	SRF1	0.903712	Bu_mutualism
2	8977674	SRF1	2	8977571	SRF1	0.903712	Bu_mutualism
2	8977674	SRF1	2	8978576	SRF1	0.903712	Bu_mutualism
2	16033300	FAC1	2	16037544	FAC1	0.829336	Gu_mutualism
2	16033300	FAC1	2	16039348	-	0.91121	Gu_mutualism
2	16037544	FAC1	2	16033300	FAC1	0.829336	Gu_mutualism
2	16037544	FAC1	2	16039348	-	0.91121	Gu_mutualism
2	16039348	-	2	16033300	FAC1	0.91121	Gu_mutualism
2	16039348	-	2	16037544	FAC1	0.91121	Gu_mutualism
2	16777709	TBL30	2	16780243	GEA6	0.890715	Gu_mutualism
2	17597020	AT2G42240	2	17596120	AT2G42230	0.955295	Gu_aggression
2	17597020	AT2G42240	2	17596174	AT2G42230	0.955295	Gu_aggression
2	17597020	AT2G42240	2	17596306	AT2G42230	0.867976	Gu_aggression
2	17597020	AT2G42240	2	17596379	AT2G42230	0.834772	Gu_aggression
2	17597020	AT2G42240	2	17597427	AT2G42240	0.800139	Gu_aggression
2	17597020	AT2G42240	2	17598046	AT2G42240	0.955295	Gu_aggression
2	17597020	AT2G42240	2	17599494	AT2G42240	0.91203	Gu_aggression
2	19481571	UNE5	2	19481988	UNE5	0.815659	Cu_antagonism
2	19481988	UNE5	2	19481571	UNE5	0.815659	Cu_antagonism
3	263618	AT3G01720	3	263124	AT3G01720	0.967449	Cu_aggression
3	263618	AT3G01720	3	263322	AT3G01720	0.935507	Cu_aggression
3	263618	AT3G01720	3	263960	AT3G01720	0.903441	Cu_aggression
3	263618	AT3G01720	3	264195	AT3G01720	0.876298	Cu_aggression
3	1238302	EIN4	3	1246911	MUG1	0.823471	Gu_mutualism
3	2064891	AT3G06620	3	2061444	AT3G06610	0.948504	Cu_antagonism
3	3103010	#N/A	3	3103312	AT3G10060	0.871685	Eu_antagonism
3	3603933	MYB65	3	3605408	AT3G11450	0.903712	Cu_antagonism
3	3604005	MYB65	3	3605408	AT3G11450	0.903712	Cu_antagonism
3	3605408	AT3G11450	3	3603933	MYB65	0.903712	Cu_antagonism
3	3605408	AT3G11450	3	3604005	MYB65	0.903712	Cu_antagonism
3	3890072	Nek7	3	3890092	Nek7	0.91121	Cu_antagonism
3	3890092	Nek7	3	3890072	Nek7	0.91121	Cu_antagonism
3	4452230	PUT4	3	4453610	-	0.823471	Cu_antagonism
3	4453610	-	3	4452230	PUT4	0.823471	Cu_antagonism
3	5111859	#N/A	3	5111049	CUC1	0.903712	Eu_antagonism
3	5775050	AAE7	3	5774540	AAE7	0.805475	Gu_aggression
3	5775050	AAE7	3	5777263	CTL2	0.805475	Gu_aggression
3	5872184	GA20X5	3	5871518	GA20X5	0.95219	Con_aggression
3	5880477	UPL6	3	5876702	UPL6	0.81873	Con_aggression
3	7949361	AT3G22421	3	7949149	WNK2	0.858386	Bu_mutualism
3	7957545	AT3G22430	3	7955257	AT3G22430	0.903712	Eu_antagonism
3	7964502	OASA2	3	7961919	AT3G22440	0.903712	Eu_antagonism

3	15697200	*AT3G43835*	3	15696449	*AT3G43830*	0.848308	Eu_antagonism
3	15756656	*DCL3*	3	15757847	*DCL3*	0.91755	Cu_antagonism
3	15757847	*DCL3*	3	15756656	*DCL3*	0.91755	Cu_antagonism
4	122676	*AT4G00280*	4	124943	*AT4G00290*	0.885704	Cu_antagonism
4	1406662	*GRH1*	4	1405973	*GRH1*	0.91755	Con_mutualism
4	1509997	-	4	1507633	-	0.917119	Gu_mutualism
4	4865340	-	4	4863366	*AT4G08032*	0.903712	Bu_altruism
4	4865340	-	4	4863663	*AT4G08032*	0.967449	Bu_altruism
4	8919093	*AT4G15640*	4	8919172	*AT4G15640*	0.948504	Eu_antagonism
4	8919172	*AT4G15640*	4	8919093	*AT4G15640*	0.948504	Eu_antagonism
4	8919172	*AT4G15640*	4	8921350	*AT4G15640*	0.899038	Eu_antagonism
4	8921350	*AT4G15640*	4	8919172	*AT4G15640*	0.899038	Eu_antagonism
4	12343539	*CHX17*	4	12343347	*CHX17*	0.903712	Eu_antagonism
4	12343752	*CHX17*	4	12343347	*CHX17*	0.903712	Eu_antagonism
4	12908721	-	4	12910654	*AT4G25170*	0.823471	Bu_altruism
4	13010771	*ABCB28*	4	13010466	*ABCB28*	0.846878	Cu_antagonism
4	13010771	*ABCB28*	4	13011004	*ABCB28*	0.91755	Cu_antagonism
4	13011912	*ABCB28*	4	13010466	*ABCB28*	0.846878	Cu_antagonism
4	13011912	*ABCB28*	4	13011004	*ABCB28*	0.91755	Cu_antagonism
4	13030241	*SLK3*	4	13032861	*SLK1*	0.836081	Cu_antagonism
4	13054736	*ACYB-2*	4	13056621	*AT4G25580*	0.903712	Cu_antagonism
4	13056621	*AT4G25580*	4	13054736	*ACYB-2*	0.903712	Cu_antagonism
4	15222944	*AT4G31360*	4	15223348	*AT4G31360*	0.877982	Cu_antagonism
4	15223348	*AT4G31360*	4	15222944	*AT4G31360*	0.877982	Cu_antagonism
4	15419654	*AT4G31880*	4	15419728	*AT4G31180*	0.877982	Cu_antagonism
4	15419654	*AT4G31880*	4	15421099	*AT4G31180*	0.819858	Cu_antagonism
4	15419728	*AT4G31880*	4	15419654	*AT4G31180*	0.877982	Cu_antagonism
4	15419728	*AT4G31880*	4	15421099	*AT4G31180*	0.935402	Cu_antagonism
4	15419728	*AT4G31880*	4	15425107	-	0.935402	Cu_antagonism
4	15421099	*AT4G31880*	4	15419654	*AT4G31180*	0.819858	Cu_antagonism
4	15421099	*AT4G31880*	4	15419728	*AT4G31180*	0.935402	Cu_antagonism
4	15421099	*AT4G31880*	4	15425107	-	0.874232	Cu_antagonism
4	15425107	-	4	15419728	*AT4G31180*	0.935402	Cu_antagonism
4	15425107	-	4	15421099	*AT4G31180*	0.874232	Cu_antagonism
4	16833858	*AT4G35410*	4	16834561	*DRL1*	0.879111	Gu_mutualism
4	16834561	*DRL1*	4	16833858	*AT4G35401*	0.879111	Gu_mutualism
5	667064	*UPL4*	5	668716	*UPL4*	0.91121	Cu_antagonism
5	668716	*UPL4*	5	667064	*UPL4*	0.91121	Gu_aggression
5	3408362	*AT5G10780*	5	3408858	*AT5G10780*	0.903712	Cu_antagonism
5	3408362	*AT5G10780*	5	3410142	*UBP22*	0.903712	Cu_antagonism
5	3408858	*AT5G10780*	5	3408362	*AT5G10780*	0.903712	Cu_antagonism
5	3408858	*AT5G10780*	5	3409353	*AT5G10780*	0.903712	Cu_antagonism
5	3408858	*AT5G10780*	5	3410142	*UBP22*	0.815659	Cu_antagonism
5	3408858	*AT5G10780*	5	3410949	*UBP22*	0.903712	Cu_antagonism
5	3409353	*AT5G10780*	5	3408858	*AT5G10780*	0.903712	Cu_antagonism
5	3409353	*AT5G10780*	5	3410142	*UBP22*	0.903712	Cu_antagonism
5	3410142	*UBP22*	5	3408362	*AT5G10780*	0.903712	Cu_antagonism
5	3410142	*UBP22*	5	3408858	*AT5G10780*	0.815659	Cu_antagonism
5	3410142	*UBP22*	5	3409353	*AT5G10780*	0.903712	Cu_antagonism
5	3410142	*UBP22*	5	3410949	*UBP22*	0.903712	Cu_antagonism
5	3410949	*UBP22*	5	3408858	*AT5G10780*	0.903712	Cu_antagonism
5	3410949	*UBP22*	5	3410142	*UBP22*	0.903712	Cu_antagonism
5	3410949	*UBP22*	5	3415727	*AT5G10800*	0.823471	Cu_antagonism

5	3415727	*AT5G10800*	5	3410949	*UBP22*	0.823471	Cu_antagonism
5	3415727	*AT5G10800*	5	3416182	*AT5G10800*	0.823471	Cu_antagonism
5	3416182	*AT5G10800*	5	3415727	*AT5G10800*	0.823471	Cu_antagonism
5	3416182	*AT5G10800*	5	3417902	*AT5G10800*	0.823471	Cu_antagonism
5	3417902	*AT5G10800*	5	3416182	*AT5G10800*	0.823471	Cu_antagonism
5	3417902	*AT5G10800*	5	3419070	*ER*	0.823471	Cu_antagonism
5	3417902	*AT5G10800*	5	3421461	*AT5G10820*	0.823471	Cu_antagonism
5	3419070	*ER*	5	3417902	*AT5G10800*	0.823471	Cu_antagonism
5	3419070	*ER*	5	3422619	*AT5G10820*	0.903712	Cu_antagonism
5	3421461	*AT5G10820*	5	3417902	*AT5G10800*	0.823471	Cu_antagonism
5	3421461	*AT5G10820*	5	3422619	*AT5G10820*	0.903712	Cu_antagonism
5	3422619	*AT5G10820*	5	3419070	*ER*	0.903712	Cu_antagonism
5	3422619	*AT5G10820*	5	3421461	*AT5G10820*	0.903712	Cu_antagonism
5	3480514	*AT5G11000*	5	3480589	*AT5G11000*	0.953806	Pu_antagonism
5	3480514	*AT5G11000*	5	3480966	-	0.953806	Pu_antagonism
5	3480514	*AT5G11000*	5	3481010	-	0.909175	Pu_antagonism
5	3480514	*AT5G11000*	5	3483194	-	0.863185	Pu_antagonism
5	3480589	*AT5G11000*	5	3480514	*AT5G11000*	0.953806	Pu_antagonism
5	3480589	*AT5G11000*	5	3481010	-	0.953806	Pu_antagonism
5	3480589	*AT5G11000*	5	3483194	-	0.904989	Pu_antagonism
5	3480966	-	5	3480514	*AT5G11000*	0.953806	Pu_antagonism
5	3480966	-	5	3481010	-	0.953806	Pu_antagonism
5	3480966	-	5	3483194	-	0.904989	Pu_antagonism
5	3481010	-	5	3480514	*AT5G11000*	0.909175	Pu_antagonism
5	3481010	-	5	3480589	*AT5G11000*	0.953806	Pu_antagonism
5	3481010	-	5	3480966	-	0.953806	Pu_antagonism
5	3481010	-	5	3483194	-	0.863185	Pu_antagonism
5	3483194	-	5	3480514	*AT5G11000*	0.863185	Cu_antagonism
5	3483194	-	5	3480589	*AT5G11000*	0.904989	Cu_antagonism
5	3483194	-	5	3480966	-	0.904989	Cu_antagonism
5	3483194	-	5	3481010	-	0.863185	Cu_antagonism
5	3520545	-	5	3521242	-	0.858971	Pu_antagonism
5	3521242	-	5	3520545	-	0.858971	Cu_antagonism
5	3521242	-	5	3521710	-	0.908205	Cu_antagonism
5	3521710	-	5	3521242	-	0.908205	Pu_antagonism
5	3537533	*SPS2F*	5	3539485	*SPS2F*	0.926699	Pu_antagonism
5	3537533	*SPS2F*	5	3540289	*SPS2F*	0.926699	Pu_antagonism
5	3537533	*SPS2F*	5	3542532	*AT5G11130*	0.890787	Pu_antagonism
5	3539485	*SPS2F*	5	3537533	*SPS2F*	0.926699	Pu_antagonism
5	3539485	*SPS2F*	5	3542532	*AT5G11130*	0.961247	Pu_antagonism
5	3540289	*SPS2F*	5	3537533	*SPS2F*	0.926699	Pu_antagonism
5	3540289	*SPS2F*	5	3542532	*AT5G11130*	0.961247	Pu_antagonism
5	3542532	*AT5G11130*	5	3537533	*SPS2F*	0.890787	Pu_antagonism
5	3542532	*AT5G11130*	5	3539485	*SPS2F*	0.961247	Pu_antagonism
5	3542532	*AT5G11130*	5	3540289	*SPS2F*	0.961247	Pu_antagonism
5	3547479	*VAMP713*	5	3549811	-	0.89059	Cu_antagonism
5	3549811	-	5	3547479	*VAMP713*	0.89059	Cu_antagonism
5	3614061	-	5	3613523	*YUC4*	0.875464	Cu_antagonism
5	3648231	*AT5G11430*	5	3649416	*AT5G11430*	0.874232	Cu_antagonism
5	3649416	*AT5G11430*	5	3648231	*AT5G11430*	0.874232	Cu_antagonism
5	3666881	*AT5G11470*	5	3663584	*AT5G11470*	0.858386	Cu_antagonism
5	3666881	*AT5G11470*	5	3665774	*AT5G11470*	0.818734	Cu_antagonism
5	3686139	*ASP3*	5	3689078	-	0.950429	Cu_antagonism

5	3686741	*ASP3*	5	3689078	-	0.950429	Cu_antagonism
5	3687178	*ASP3*	5	3689078	-	0.950429	Cu_antagonism
5	3688241	-	5	3689078	-	0.950429	Cu_antagonism
5	3689078	-	5	3686139	*ASP3*	0.950429	Cu_antagonism
5	3689078	-	5	3686741	*ASP3*	0.950429	Cu_antagonism
5	3689078	-	5	3687178	*ASP3*	0.950429	Cu_antagonism
5	3689078	-	5	3688241	-	0.950429	Cu_antagonism
5	3704279	*GuILO3*	5	3704802	*GuIL03*	0.871685	Cu_antagonism
5	3704802	*GuILO3*	5	3701691	-	0.863657	Cu_antagonism
5	3704802	*GuILO3*	5	3704279	*GuIL03*	0.871685	Cu_antagonism
5	3728453	*TINY2*	5	3729221	-	0.955295	Cu_antagonism
5	3728453	*TINY2*	5	3731946	-	0.875464	Cu_antagonism
5	3729221	-	5	3728453	*TINY2*	0.955295	Cu_antagonism
5	3729221	-	5	3731946	-	0.834772	Cu_antagonism
5	3737307	*AT5G11610*	5	3734555	*AT5G11600*	0.810401	Cu_antagonism
5	3741193	*NOXY2*	5	3741348	*NOXY2*	0.867604	Cu_antagonism
5	3741193	*NOXY2*	5	3744717	*AT5G11650*	0.867604	Cu_antagonism
5	3741348	*NOXY2*	5	3741193	*NOXY2*	0.867604	Cu_antagonism
5	3741348	*NOXY2*	5	3741637	*NOXY2*	0.830009	Cu_antagonism
5	3744717	*AT5G11650*	5	3741193	*NOXY2*	0.867604	Cu_antagonism
5	3744717	*AT5G11650*	5	3741637	*NOXY2*	0.830009	Cu_antagonism
5	3764090	*AT5G11700*	5	3764778	*AT5G11700*	0.810401	Cu_antagonism
5	3764090	*AT5G11700*	5	3769610	*AT5G11700*	0.853873	Cu_antagonism
5	3769610	*AT5G11700*	5	3764090	*AT5G11700*	0.853873	Cu_antagonism
5	3769610	*AT5G11700*	5	3764778	*AT5G11700*	0.950429	Cu_antagonism
5	3774046	*AT5G11710*	5	3774465	*AT5G11710*	0.960222	Pu_antagonism
5	3774465	*AT5G11710*	5	3774046	*AT5G11710*	0.960222	Cu_antagonism
5	6367261	-	5	6370884	*AT5G19050*	0.836081	Cu_antagonism
5	6370884	*AT5G19050*	5	6367261	-	0.836081	Cu_antagonism
5	6381285	-	5	6380360	*LUL3*	0.808556	Cu_antagonism
5	6571559	*AT5G19480*	5	6572794	*AT5G19480*	0.931784	Cu_antagonism
5	6571559	*AT5G19480*	5	6572992	*AT5G19480*	0.818089	Cu_antagonism
5	6572245	*AT5G19480*	5	6572794	*AT5G19480*	0.931784	Cu_antagonism
5	6572245	*AT5G19480*	5	6572992	*AT5G19480*	0.818089	Cu_antagonism
5	6572576	*AT5G19480*	5	6572794	*AT5G19480*	0.931784	Cu_antagonism
5	6572576	*AT5G19480*	5	6572992	*AT5G19480*	0.818089	Cu_antagonism
5	6572726	*AT5G19480*	5	6572794	*AT5G19480*	0.931784	Cu_antagonism
5	6572726	*AT5G19480*	5	6572992	*AT5G19480*	0.818089	Cu_antagonism
5	6572794	*AT5G19480*	5	6571559	*AT5G19480*	0.931784	Cu_antagonism
5	6572794	*AT5G19480*	5	6572245	*AT5G19480*	0.931784	Cu_antagonism
5	6572794	*AT5G19480*	5	6572576	*AT5G19480*	0.931784	Cu_antagonism
5	6572794	*AT5G19480*	5	6572726	*AT5G19480*	0.931784	Cu_antagonism
5	6572794	*AT5G19480*	5	6573441	*AT5G19480*	0.931784	Cu_antagonism
5	6573441	*AT5G19480*	5	6572794	*AT5G19480*	0.931784	Cu_antagonism
5	6573441	*AT5G19480*	5	6572992	*AT5G19480*	0.818089	Cu_antagonism
5	6779063	*AT5G20060*	5	6779815	*NUDX19*	0.840986	Cu_antagonism
5	6779815	*NUDX19*	5	6779063	*AT5G20060*	0.840986	Cu_antagonism
5	6825667	*AT5G20220*	5	6830174	*PI*	0.922978	Cu_antagonism
5	6830174	*P1*	5	6825667	*AT5G20220*	0.922978	Cu_antagonism
5	6955406	*AT5G20560*	5	6955554	*AT5G20560*	0.91755	Cu_antagonism
5	6955406	*AT5G20560*	5	6955967	*AT5G20560*	0.846878	Cu_antagonism
5	6955554	*AT5G20560*	5	6955406	*AT5G20560*	0.91755	Cu_antagonism
5	6965654	*TBL5*	5	6962574	*AT5G20580*	0.846878	Cu_antagonism

5	6965654	*TBL5*	5	6966349	*AT5G20600*	0.91755	Cu_antagonism
5	6966349	-	5	6965654	*TBL5*	0.91755	Cu_antagonism
5	6971527	*AT5G20610*	5	6972635	*AT5G20610*	0.840986	Cu_antagonism
5	6985904	*COPT5*	5	6987427	*AT5G20660*	0.818734	Cu_antagonism
5	6986306	*AT5G20660*	5	6987427	*AT5G20660*	0.818734	Cu_antagonism
5	6986306	*AT5G20660*	5	6990737	*AT5G20660*	0.948504	Cu_antagonism
5	6987427	*AT5G20660*	5	6985904	*COPT5*	0.818734	Cu_antagonism
5	6987427	*AT5G20660*	5	6986306	*AT5G20660*	0.818734	Cu_antagonism
5	6987427	*AT5G20660*	5	6990330	*AT5G20660*	0.818734	Cu_antagonism
5	6987427	*AT5G20660*	5	6991854	-	0.818734	Cu_antagonism
5	6990330	*AT5G20660*	5	6987427	*AT5G20660*	0.818734	Cu_antagonism
5	6990330	*AT5G20660*	5	6990737	*AT5G20660*	0.948504	Cu_antagonism
5	6990330	*AT5G20660*	5	6992629	-	0.948504	Cu_antagonism
5	6990737	*AT5G20660*	5	6986306	*AT5G20660*	0.948504	Cu_antagonism
5	6990737	*AT5G20660*	5	6990330	*AT5G20660*	0.948504	Cu_antagonism
5	6990737	*AT5G20660*	5	6991854	-	0.948504	Cu_antagonism
5	6990737	*AT5G20660*	5	6992629	-	0.899038	Cu_antagonism
5	6991854	-	5	6987427	*AT5G20660*	0.818734	Cu_antagonism
5	6991854	-	5	6990737	*AT5G20660*	0.948504	Cu_antagonism
5	6991854	-	5	6992629	-	0.948504	Cu_antagonism
5	6991854	-	5	6995739	-	0.858386	Cu_antagonism
5	6992629	-	5	6990330	*AT5G20660*	0.948504	Cu_antagonism
5	6992629	-	5	6990737	*AT5G20660*	0.899038	Cu_antagonism
5	6992629	-	5	6991854	-	0.948504	Cu_antagonism
5	6992629	-	5	6995739	-	0.812472	Cu_antagonism
5	6992629	-	5	6995926	-	0.948504	Cu_antagonism
5	6995739	-	5	6991854	-	0.858386	Cu_antagonism
5	6995739	-	5	6992629	-	0.812472	Cu_antagonism
5	6995739	-	5	6995926	-	0.858386	Cu_antagonism
5	6995926	-	5	6992629	-	0.948504	Cu_antagonism
5	6995926	-	5	6995739	-	0.858386	Cu_antagonism
5	9726633	*MIRO1*	5	9726494	*MIRO1*	0.927677	Cu_antagonism
5	9852873	-	5	9851209	-	0.927677	Cu_antagonism
5	9880110	*AT5G27870*	5	9879652	*AT5G27870*	0.867441	Cu_antagonism
5	10520445	*AT5G28526*	5	10514237	*AT5G28527*	0.80454	Cu_aggression
5	10528368	-	5	10529312	-	0.88293	Cu_aggression
5	10528368	-	5	10529541	-	0.856914	Cu_aggression
5	10528368	-	5	10534780	-	0.808205	Cu_aggression
5	13369544	-	5	13366818	*AT5G35111*	0.819858	Cu_antagonism
5	13490178	*AT5G35230*	5	13490609	*AT5G35230*	0.815659	Cu_antagonism
5	13490609	*AT5G35230*	5	13490178	*AT5G35230*	0.815659	Cu_antagonism
5	13865599	*AT5G35690*	5	13867993	*AT5G35690*	0.877982	Eu_mutualism
5	13865966	*AT5G03545*	5	13867993	*AT5G35690*	0.877982	Eu_mutualism
5	13866162	*AT5G35690*	5	13867993	*AT5G35690*	0.877982	Eu_mutualism
5	19011159	*AT5G46850*	5	19011480	*AT5G46850*	0.906079	Gu_mutualism
5	19011159	*AT5G46850*	5	19012284	*VAM3*	0.863657	Gu_mutualism
5	19011159	*AT5G46850*	5	19012633	*VAM3*	0.906079	Gu_mutualism
5	19011159	*AT5G46850*	5	19012820	*VAM3*	0.863657	Gu_mutualism
5	19011480	*AT5G46850*	5	19011159	*AT5G46850*	0.906079	Gu_mutualism
5	19011480	*AT5G46850*	5	19012038	*AT5G46850*	0.871685	Gu_mutualism
5	19011480	*AT5G46850*	5	19012284	*VAM3*	0.953806	Gu_mutualism
5	19011480	*AT5G46850*	5	19012820	*VAM3*	0.953806	Gu_mutualism
5	19012038	*VAM3*	5	19011480	*AT5G46850*	0.871685	Gu_mutualism

5	19012038	*VAM3*	5	19012284	*VAM3*	0.82983	Gu_mutualism
5	19012038	*VAM3*	5	19012633	*VAM3*	0.871685	Gu_mutualism
5	19012038	*VAM3*	5	19012820	*VAM3*	0.82983	Gu_mutualism
5	19012038	*VAM3*	5	19013193	*VAM3*	0.871685	Gu_mutualism
5	19012284	*VAM3*	5	19011159	*AT5G46850*	0.863657	Gu_mutualism
5	19012284	*VAM3*	5	19011480	*AT5G46850*	0.953806	Gu_mutualism
5	19012284	*VAM3*	5	19012038	*AT5G46850*	0.82983	Gu_mutualism
5	19012284	*VAM3*	5	19012633	*VAM3*	0.953806	Gu_mutualism
5	19012284	*VAM3*	5	19012820	*VAM3*	0.909175	Gu_mutualism
5	19012284	*VAM3*	5	19013193	*VAM3*	0.953806	Gu_mutualism
5	19012633	*VAM3*	5	19011159	*AT5G46850*	0.906079	Gu_mutualism
5	19012633	*VAM3*	5	19012038	*AT5G46850*	0.871685	Gu_mutualism
5	19012633	*VAM3*	5	19012284	*VAM3*	0.953806	Gu_mutualism
5	19012633	*VAM3*	5	19012820	*VAM3*	0.953806	Gu_mutualism
5	19012820	*VAM3*	5	19011159	*AT5G46850*	0.863657	Gu_mutualism
5	19012820	*VAM3*	5	19011480	*AT5G46850*	0.953806	Gu_mutualism
5	19012820	*VAM3*	5	19012038	*AT5G46850*	0.82983	Gu_mutualism
5	19012820	*VAM3*	5	19012284	*VAM3*	0.909175	Gu_mutualism
5	19012820	*VAM3*	5	19012633	*VAM3*	0.953806	Gu_mutualism
5	19012820	*VAM3*	5	19013193	*VAM3*	0.953806	Gu_mutualism
5	19013193	*VAM3*	5	19012038	*AT5G46850*	0.871685	Gu_mutualism
5	19013193	*VAM3*	5	19012284	*VAM3*	0.953806	Gu_mutualism
5	19013193	*VAM3*	5	19012820	*VAM3*	0.953806	Gu_mutualism
5	19324172	*AT5G47690*	5	19327938	*AT5G47750*	0.91121	Cu_antagonism
5	19327938	*AT5G47700*	5	19324172	*AT5G47690*	0.91121	Cu_antagonism
5	20472163	-	5	20476125	*AT5G50310*	0.948504	Cu_antagonism
5	20476125	*AT5G50310*	5	20472163	-	0.948504	Cu_antagonism
5	20476125	*AT5G50310*	5	20477554	*AT5G50310*	0.948504	Cu_antagonism
5	20477554	*AT5G50310*	5	20476125	*AT5G50310*	0.948504	Cu_antagonism
5	20501387	*AT5G50350*	5	20501258	*AT5G50350*	0.832293	Cu_antagonism
5	21525178	*AT5G53090*	5	21525637	*AT5G53090*	0.847443	Cu_mutualism
5	21525178	*AT5G53090*	5	21525953	-	0.847443	Cu_mutualism
5	25443680	*AT5G60470*	5	25441459	*RMI1*	0.897656	Cu_antagonism
5	25443680	*AT5G60470*	5	25441526	*RMI1*	0.848308	Cu_antagonism
5	25443680	*AT5G60470*	5	25442858	*RMI1*	0.897656	Cu_antagonism
5	25443680	*AT5G60470*	5	25443333	*RMI1*	0.848308	Cu_antagonism
5	25443680	*AT5G60470*	5	25443981	*RMI1*	0.848308	Cu_antagonism
5	25443680	*AT5G60470*	5	25445916	*AT5G63550*	0.803447	Cu_antagonism
5	25443680	*AT5G60470*	5	25447660	*AT5G63550*	0.803447	Cu_antagonism
5	25443680	*AT5G60470*	5	25447730	*AT5G63550*	0.897656	Cu_antagonism
5	25480351	*AT5G63640*	5	25484569	-	0.851026	Cu_antagonism
5	25484569	-	5	25480351	*AT5G63640*	0.851026	Cu_antagonism
5	25576787	*TOP3A*	5	25570047	*AT5G63900*	0.91755	Cu_antagonism
5	25576787	*TOP3A*	5	25574863	*TOP3A*	0.840986	Cu_antagonism
5	25576787	*TOP3A*	5	25575629	*TOP3A*	0.91755	Cu_antagonism
5	25576787	*TOP3A*	5	25575883	*TOP3A*	0.91755	Cu_antagonism
5	25576787	*TOP3A*	5	25575946	*TOP3A*	0.840986	Cu_antagonism
5	25576787	*TOP3A*	5	25576197	*TOP3A*	0.91755	Cu_antagonism
5	25576787	*TOP3A*	5	25577648	*TOP3A*	0.840986	Cu_antagonism
5	25576787	*TOP3A*	5	25577785	*TOP3A*	0.91755	Cu_antagonism
5	25576787	*TOP3A*	5	25578214	*TOP3A*	0.91755	Cu_antagonism
5	25576787	*TOP3A*	5	25578698	*TOP3A*	0.91755	Cu_antagonism
5	25576787	*TOP3A*	5	25579172	*TOP3A*	0.91755	Cu_antagonism

5	25576787	*TOP3A*	5	25579459	*TOP3A*	0.91755	Cu_antagonism
5	25690076	*AT5G64220*	5	25683638	*SC35*	0.91755	Cu_antagonism
5	26835954	-	5	26836247	*CYCD3;2*	0.848308	Cu_mutualism

Supplementary Table 9 The list of accessions

ID	Name	Latitude	Longitude	Country
5837	Bor-1	49.4013	16.2326	Czech Republic
6008	Duk	49.1	16.2	Czech Republic
6009	Eden-1	62.877	18.177	Sweden
6016	Eds-1	62.9	18.4	Sweden
6024	Fly2-2	*55.7509*	*13.3712*	Sweden
6039	Hovdala-2	56.1	13.74	Sweden
6040	Kni-1	55.66	13.4	Sweden
6042	Lom1-1	56.09	13.9	Sweden
6043	Lov-1	62.801	18.079	Sweden
6046	Lov-5	62.801	18.079	Sweden
6064	Nyl-2	*62.9513*	*18.2763*	Sweden
6074	Or-1	*56.4573*	*16.1408*	Sweden
6243	Tottarp-2	*55.95*	*13.85*	Sweden
6009	Eden-1	*62.877*	*18.177*	Sweden
8222	Lis-2	56.0328	14.775	Sweden
8230	Algutsrum	56.68	16.5	Sweden
8231	Bro1-6	56.3	16	Sweden
8233	Dem-4	41.1876	-87.1923	United States of America
8235	Hod	48.8	17.1	Czech Republic
8236	HSm	49.33	15.76	Czech Republic
8237	Kavlinge-1	55.8	13.1	Sweden
8238	Kent	51.15	0.4	United Kingdom
8239	Koln	51	7	Germany
8240	Kulturen-1	55.705	13.196	Sweden
8241	Liarum	55.9473	13.821	Sweden
8242	Lillo-1	56.1494	15.7884	Sweden
8243	PHW-2	*43.7703*	*11.2547*	Italy
8245	Seattle-0	*47*	*-122.2*	United States of America
8247	San-2	*56.07*	*13.74*	Sweden
8248	Shahdara	*38.35*	*68.48*	Tajikistan
8249	Vimmerby	*57.7*	*15.8*	Sweden
8251	Ag-0	45	1.3	France
8253	An-1	51.2167	4.4	Belgium
8254	Ang-0	50.3	5.3	Belgium
8256	Ba1-2	56.4	12.9	Sweden
8258	Ba4-1	56.4	12.9	Sweden
8259	Ba5-1	56.4	12.9	Sweden
8261	Bg-2	47.6479	-122.305	United States of America
8262	Bil-5	63.324	18.484	Sweden
8263	Bil-7	63.324	18.484	Sweden
8264	Bla-1	41.6833	2.8	Spain
8265	Blh-1	48	19	Czech Republic
8266	Boo2-1	55.86	13.51	Sweden
8268	Bor-4	49.4013	16.2326	Czech Republic
8269	Br-0	49.2	16.6166	Czech Republic
8270	Bs-1	47.5	7.5	Switzerland
8271	Bu-0	50.5	9.5	Germany
8272	Bur-0	54.1	-6.2	Ireland

8274	Can-0	29.2144	-13.4811	Spain
8275	Cen-0	49	0.5	France
8276	CIBC-17	51.4083	-0.6383	United Kingdom
8277	CIBC-5	51.4083	-0.6383	United Kingdom
8278	Co	40.2077	-8.42639	Portugal
8280	Ct-1	37.3	15	Italy
8283	Dra3-1	55.76	14.12	Sweden
8284	DraII-1	49.4112	16.2815	Czech Republic
8285	DraIII-1	49.4112	16.2815	Czech Republic
8287	Eden-2	62.877	18.177	Sweden
8288	Edi-0	55.9494	-3.16028	United Kingdom
8289	Ei-2	50.3	6.3	Germany
8290	En-1	50	8.5	Germany
8291	Est-1	58.3	25.3	Russia
8292	Fab-2	63.0165	18.3174	Sweden
8293	Fab-4	63.0165	18.3174	Sweden
8294	Fei-0	40.5	-8.32	Portugal
8295	Ga-0	50.3	8	Germany
8296	Gd-1	53.5	10.5	Germany
8297	Ge-0	46.5	6.08	Switzerland
8298	Got-22	51.5338	9.9355	Germany
8299	Got-7	51.5338	9.9355	Germany
8300	Gr-1	47	15.5	Austria
8301	Gu-0	50.3	8	Germany
8303	H55	49	15	Czech Republic
8304	Hi-0	52	5	Netherlands
8306	Hov4-1	56.1	13.74	Sweden
8308	HR-10	51.4083	-0.6383	United Kingdom
8309	HR-5	51.4083	-0.6383	United Kingdom
8310	Hs-0	52.24	9.44	Germany
8311	In-0	47.5	11.5	Austria
8312	Is-0	50.5	7.5	Germany
8313	Jm-0	49	15	Czech Republic
8314	Ka-0	47	14	Austria
8317	Kno-10	41.2816	-86.621	United States of America
8318	Kno-18	41.2816	-86.621	United States of America
8319	Kondara	38.48	68.49	Tajikistan
8320	Kz-1	49.5	73.1	Kazakhstan
8322	Kz-9	49.5	73.1	Kazakhstan
8323	Lc-0	57	-4	United Kingdom
8324	Ler-1	47.984	10.8719	Germany
8325	Lip-0	50	19.3	Poland
8326	Lis-1	56.0328	14.775	Sweden
8328	LL-0	41.59	2.49	Spain
8329	Lm-2	48	0.5	France
8332	Lp2-2	49.38	16.81	Czech Republic
8333	Lp2-6	49.38	16.81	Czech Republic
8334	Lu-1	55.71	13.2	Sweden
8335	Lund	55.71	13.2	Sweden
8336	Lz-0	46	3.3	France
8337	Mir-0	*44*	12.37	Italy

8338	Mr-0	44.15	9.65	Italy
8339	Mrk-0	49	9.3	Germany
8340	Ms-0	55.7522	37.6322	Russia
8341	Mt-0	32.34	22.46	Libya
8342	Mz-0	50.3	8.3	Germany
8343	Na-1	47.5	1.5	France
8344	Nd-1	50	10	Switzerland
8345	NFA-10	51.4083	-0.6383	United Kingdom
8346	NFA-8	51.4083	-0.6383	United Kingdom
8347	Nok-3	52.24	4.45	Netherlands
8348	Nw-0	50.5	8.5	Germany
8349	Omo2-1	56.1481	15.8199	Sweden
8350	Omo2-3	56.1481	15.8155	Sweden
8351	Ost-0	60.25	18.37	Sweden
8353	Pa-1	38.07	13.22	Italy
8354	Per-1	58	56.3167	Russia
8355	Petergof	59	29	Russia
8358	Pna-10	42.0945	-86.3253	United States of America
8359	Pna-17	42.0945	-86.3253	United States of America
8360	Pro-0	43.25	-6	Spain
8361	Pu2-23	49.42	16.36	Czech Republic
8362	Pu2-7	49.42	16.36	Czech Republic
8363	Pu2-8	49.42	16.36	Czech Republic
8364	Ra-0	46	3.3	France
8365	Rak-2	49	16	Czech Republic
8366	Rd-0	50.5	8.5	Germany
8367	Ren-1	48.5	-1.41	France
8368	Ren-11	48.5	-1.41	France
8369	Rev-1	55.6942	13.4504	Sweden
8370	Rmx-A02	42.036	-86.511	United States of America
8371	Rmx-A180	42.036	-86.511	United States of America
8372	RRS-10	41.5609	-86.4251	United States of America
8373	RRS-7	41.5609	-86.4251	United States of America
8374	Rsch-4	56.3	34	Russia
8375	Rubezhnoe-1	49	38.28	Ukraine
8376	Sanna-2	62.69	18	Sweden
8378	Sap-0	49.49	14.24	Czech Republic
8379	Se-0	38.3333	-3.53333	Spain
8381	Sorbo	38.35	68.48	Tajikistan
8382	Spr1-2	58.4168	14.1612	Sweden
8383	Spr1-6	58.4168	14.1612	Sweden
8384	Sq-1	51.4083	-0.6383	United Kingdom
8385	Sq-8	51.4083	-0.6383	United Kingdom
8386	Sr:5	58.9	11.2	Sweden
8387	St-0	59	18	Sweden
8388	Stw-0	52	36	Russia
8389	Ta-0	49.5	14.5	Czech Republic
8390	Tamm-2	60	23.5	Finland
8391	Tamm-27	60	23.5	Finland
8392	Ts-1	41.7194	2.93056	Spain
8393	Ts-5	41.7194	2.93056	Spain

8394	Tsu-1	*34.43*	*136.31*	Japan
8395	Tu-0	*45*	*7.5*	Italy
8396	Ull2-3	*56.0648*	*13.9707*	Sweden
8397	Ull2-5	*56.0648*	*13.9707*	Sweden
8398	Uod-1	*48.3*	*14.45*	Austria
8399	Uod-7	*48.3*	*14.45*	Austria
8400	Van-0	*49.3*	*-123*	Canada
8401	Var2-1	*55.58*	*14.334*	Sweden
8402	Var2-6	*55.58*	*14.334*	Sweden
8403	Wa-1	*52.3*	*21*	Poland
8404	Wei-0	*47.25*	*8.26*	Switzerland
8405	Ws-0	*52.3*	*30*	Russia
8406	Ws-2	*52.3*	*30*	Russia
8407	Wt-5	*52.3*	*9.3*	Germany
8408	Yo-0	*37.45*	*-119.35*	United States of America
8409	Zdr-1	*49.3853*	*16.2544*	Czech Republic
8410	Zdr-6	*49.3853*	*16.2544*	Czech Republic
8412	Sav-0	*49.1833*	*15.8833*	Czech Republic
8420	Kelsterbach-4	50.0667	8.5333	Germany
8421	Buckhorn Pass	41.3599	-122.755	United States of America
8422	Fja1-1	56.06	14.29	Sweden
8423	Hov2-1	56.1	13.74	Sweden
8424	Kas-2	35	77	India
8426	Ull1-1	*56.06*	*13.97*	Sweden
8428	Uod-2	*48.3*	*14.45*	Austria
8429	N13	*61.36*	*34.15*	Russia
8430	Lisse	52.25	4.5667	Netherlands
9057	Vinslov	*56.1*	*13.9167*	Sweden
9058	Vastervik	*57.75*	*16.6333*	Sweden

Supplementary Table 10 The relative abundance of top 100 OTUs in bacteria and fungi

OTU ID								
	5837	6009	6016	6024	6039	6040	6042	6043
1	365.67	661.67	526.75	528	184	402.33	631.33	480.5
2	88	708	724.25	85.5	60.33	36.33	633	413.25
3	23	101	132	60	141.33	62.67	73.67	81.25
4	0.33	4.67	3	111	49	55	0	0
5	305.67	0	0	0	371.67	164.33	0	0
6	22	146.67	110.75	38	47.33	35.67	73.67	96.5
7	0	0	22.25	2.5	32	12.67	0	0
8	224.33	0	43	0	0	149.67	0	0
9	4.33	31.67	94.75	55	40.67	0	19.33	174
10	0	0	5.75	0	0	361	0	0
11	53.33	24.33	7.5	171	0	0	1.33	0.25
12	152	17	20.5	0	0	0	96.67	100
13	0	0	0.5	11	37.33	23	0	0
14	101	0	0	0	99	162.67	0	0
15	21.33	17	18.5	72.5	6.67	0	10	51
16	15	20.33	19	20	4.67	4.33	17.67	18.5
17	0	85	0	19.5	0	0	0	0
18	0.67	38.67	27.25	0	0	55	66	0
19	0	1	33.5	0	1	0	7	67.75
20	12.67	18.67	17.75	11.5	7	8.67	5	8.25
21	45	0	0	0	51.33	7	0	0
22	0	18	10.75	0	0	1	5.33	26.25
23	0	9.33	17.5	0	31.67	84	19	0.5
24	0	0	0	0	0	5	0	0
25	0	52	30.75	0	0	27.33	60.67	23.5
26	0	0	0	0	0	0	0	0
27	0	32	24	0	23.33	0	0	8.75
28	7.33	0	0	0	0	0	0	0
29	0	0	17.25	0	0	4.33	0	0
30	0	69	31	0	0	1.33	48.33	85
31	3.67	6.67	8.5	3	5.67	1.33	4.33	12.5
32	15.33	10	10	13.5	2	0	1.33	16.25
33	0	0.33	0.5	1.5	0	38	0	0
34	0	63.33	9.25	69.5	0	0	0	34.5
35	24.33	3.67	4	0	0	0	5.67	17.25
36	0	0	10.5	0	20	0.33	0	0
37	0	0	0	0	12.67	0	0	0
38	0	6	10.75	3.5	6	7.33	9.33	3
39	41.67	0	5.75	0	0	0	0	0
40	0.33	1.67	0	0	0	0	0	0.25
41	6.67	4	2	4.5	2.67	0.67	7.33	1
42	0	6.33	19.5	6	5.67	0	1	7
43	50.67	0	0	0	50	35	0	0
44	0	0	0.25	0.5	1.67	9.33	0	0
45	23	2	4.25	0	0	0	0	5.25
46	8.33	4	0	0	54.67	0	0	0.25
47	25	0	0	0	0	0.33	0	0
48	0	0	0	0	19	0.33	0	0
49	1.33	0	0	0	0	0	0	0
50	0	20	5	0	0.33	18.33	24.33	16.75

51	0	0.33	0	0	0	50.33	46	0
52	0	0	0	0	0	3.33	0	0
53	1	20.67	6	0	0.33	10.67	25	8.75
54	5.33	6	3.75	10.5	4	2.67	8	5.5
55	3.67	0.67	15.75	0	3.67	2	0	3.25
56	0	0	0	65	0	0	0	0
57	3.67	0.67	0.5	0.5	0.67	2.33	2	2.25
58	0.67	3	2.75	0.5	1	2.67	0.67	0.25
59	0	0	4	0	0	3.67	0	0
60	0.33	0	33.5	0	3.67	0	0	10
61	1.33	0	0	0	0.67	0.67	0	0
62	0	0.67	0.25	4	0	0	2	2
63	3	4	9.75	0	0	0	4.33	7
64	0	0	0	0	0	0	0	0
65	0.33	0	0	0	0	0	0	0
66	5.67	0	0.25	0	0	0.33	0.33	0.5
67	0	0	0	0	0	0	0.33	0.5
68	0	7	1.25	0	0.67	2	1.33	0.75
69	3	9.33	2.75	5.5	0.33	5.33	8	7.75
70	6.67	4.33	2.5	6.5	3	2.33	7.67	2
71	1.67	3.33	0	0.5	0	0.67	0	0
72	0.67	0.33	4	0	3.33	0	0.33	2.25
73	9	4.67	0.75	0.5	0	0	0.33	2.75
74	11.33	0	0	0	13	2.33	0	0
75	3.33	8	3.5	4.5	4	0	0.33	2.5
76	0.67	2.67	2.75	3.5	0.67	0	4	3
77	3.67	1.33	2	3.5	0.33	1	3.67	5.25
78	1.67	6.67	7.75	0	2	3.33	2	3
79	0	0	0.25	0.5	0	6	0	0.25
80	5.33	0	0	0	5	0	3	0
81	0	0	2.25	0	0	1.67	0	0
82	0	0	0	0	0	0	0	0
83	10.67	0	0	0	0	0	0	0
84	0	0	0	0	0	0	0	0
85	2.67	0.67	3.75	0.5	0	0	0.33	0.5
86	0.33	1.33	0	0	3	6	2	2
87	0	0	0	0	0	0	0	0.25
88	0	0	0	0	0	1	2.33	0
89	0	1	0.5	0	1	1.67	2.67	1.5
90	0	10	4.75	0.5	0	3	2	3.25
91	0	9.33	1.5	0	0	8.67	70	7.25
92	1	0	0	0	4	0.67	0	0
93	28.67	0.33	0	0.5	0	0.67	1.67	0.5
94	0	0.33	4	0	5	1	0	2.25
95	0	12	6	2	13.33	2.33	10.67	1.5
96	0	7.33	3.5	0	0.33	1.33	2	2
97	0	0	0	1	0	0	0.33	4.25
98	0	2	2	1	1.67	0.67	3.33	0.75
99	0	0	0.75	0	6.67	3	0	0
100	0.33	5.67	1.5	8.5	0.67	1	0.67	1.25
101	59	47.33	0.25	0.5	0	59.67	9.33	68.33
102	0	0	0	0	60.67	0	0	0
103	31	78	0	24	0	20	0	2.67
104	0	0	0	0	0	0	0	0

105	6	0.33	44.25	27	0	10.67	14.33	30.33
106	48.67	0	41.25	18.5	0	0.33	0	0
107	0	0	0	0	0	0	0	0
108	25.67	0	13.75	9.5	0	0	0	0
109	3.67	8.33	3.75	0	11.33	0	0	0
110	0	0	0	0	0	0	0	0.33
111	0	0	0	0	23.33	0	0	0.33
112	28.67	0	24.25	5	0	0	0	0
113	2.67	2.67	0	0	0	7.33	0	0
114	0	0.33	22.25	0	0	44	26.67	41
115	0	62.33	0	0	4	31.33	0	0.67
116	2	51.67	0.25	0.5	0	5	0	7.67
117	5.33	0.33	0	0	1	3.67	0	1
118	0	1	0	0	0	0	0	0
119	0.33	1	0	0	20.33	0.33	0	0
120	0	0.33	0	0	0	0	0	0
121	0.67	0	0.25	0	0	0	0	0
122	0	0	0	0	0	0	0	0
123	18.33	6	0	0	16.67	0	0	0
124	1.67	0.33	0	0	0	0	0.33	0
125	0	7.67	0	0.5	0	0.67	16	11
126	1	0	22	0.5	0	23.67	50.67	55.33
127	2.67	10.67	0	2	0	4	0	2.33
128	1	0	1	0.5	0	0.67	0	0
129	0.67	19.67	0	0	0	0	8	5.67
130	0	0	0	0	0	4	1.33	1.67
131	0	0.33	0	0	0	0	0	0
132	4.33	0	12	15	0	0.33	0	0
133	0	0	0.25	0	9	0.33	8.67	1.33
134	3.67	0	0	0	0.67	0	35	3.67
135	0.33	0	0	0	28.33	0	12.67	6.33
136	0	0	0	0	0	0	0	0
137	0	2.33	3.75	0.5	29	0.67	3	0.33
138	0	0	0.5	0	0	16	0	0
139	0	0	0.25	0	62	0	0.33	7.67
140	0	0	0.75	2	0	0.33	0	0
141	0	0	0.25	0	0	0	0	0
142	0	4	1.75	1.5	0	3.33	2	12.67
143	0	0	0	0	32	0.33	0	0
144	0	0	0	0	0	2.33	0	14.33
145	0	0	0	0	0	0	0	0
146	0.67	0.67	0.25	0	1.67	0.67	2	18.33
147	4.67	0	0	8.5	0	0	0	0
148	8.33	9.67	0	0	0	0	0	1
149	0	0	0	0	0.33	0	0	0
150	0	0	0.25	0.5	0	0	0	0
151	0	1.67	2.75	0.5	0	1	1	0
152	0	5.67	16.25	0.5	0	3.67	10	1
153	0	0	0	2	0	0	0	0
154	0	0	0	0	0	0	0	1.33
155	0	0.33	0	2	0	0	1.33	1.33
156	0	1	0	0.5	0	1.33	0.33	0.33
157	2.67	0	1.5	3	0	0	0	0
158	2.33	0	0	0	0	2	0	0.33

159	1.67	0.67	0	0	0	5.33	3	2.67
160	1	0	0	0	0	0	0	0
161	0	0	0	0	0	0	0	0
162	9	0.33	0	0	13.33	0	0	0
163	0	0	0	0	1.67	0	0	0
164	0	8.33	0	15	0	0	0	0
165	0.33	0	1.5	4	0	0	0	0
166	1	0	0	1	0	0	0	0
167	5.67	0	0	0	17	0	0.33	0
168	0	0	0	0	0	0	0	0
169	0	0	0	0	0	1.33	0	0
170	0	0	0	0	0	0	0	0
171	0	0	0	0	0	0	0	0
172	0	0	0	0	0	0	0	0
173	0	0	0	0	0	0	0	0
174	0	6.67	1.5	0.5	0	2	5	1.33
175	0	0	1.25	0.5	0	0.33	0	22
176	0	1.33	0	0	0.67	9.33	0	0
177	3.33	0	0	0	0	16	0	4.33
178	0.67	0	0	0	0	1.33	0	0
179	11.33	1.33	0.25	0	0.67	0	0.33	0
180	0	0	0.5	0	0	0.67	8	0.67
181	0	1.33	0.25	0	0	0	0.33	0
182	6.33	3.67	0	0	2.67	0	0	0.67
183	0	0	0	0	0	14	0.33	1
184	0.67	0	0	0	5	0	0	0
185	0	0	0	0	0	1	0	0
186	0	0	0	0	0	0	0	0
187	0	0.67	0	0	0.33	0.33	0	0
188	0	0	1.5	0	0	2.33	3	2
189	0	0	0	2	0	0	0	0
190	1.33	0	0	0	9.67	0	0	0
191	1	0.67	0	0	1	0	0	0
192	0	0.33	0	1	0	0	0	0
193	0	1	0	0	0	0	0.33	0.33
194	0	2	0	3	0	0	0.33	1.67
195	0	0	4.75	0	0	1.67	5.67	1.67
196	0	1	0	0	0	1.33	17.67	1.67
197	0.67	0	0.25	0	0	0	0	0
198	0	0	0	0	0	0	0	0
199	0	1	0	1	0	0	0	0
200	3.67	0	0	0	0	0	0	0

6046	6064	6074	6088	6243	6909	8222	8230	8231
937.67	467.25	370.25	405.5	508	634.33	649.25	538.5	194
313	174	131.5	102	44	114	425.5	338.25	98.5
672.67	37.5	40.5	101.25	126.33	33.33	187.75	126.25	62
321.33	75.75	0.25	159.25	97.67	26	107.75	6.25	2
0	183	168	198.5	0	0	148.5	0	239
331.33	48.75	50.5	46	37	47.33	87.25	85.75	30
179.33	9.25	14.5	29.5	20	368.67	30	5.75	4.5
0	0	53.25	21.75	0	0	36.5	46.5	307
124.33	16.5	33.75	25	8.67	24	41.75	40.25	0
0	0.5	0	0.5	0	91.33	371.5	2.5	0
18.67	1.75	4.25	20.5	31	6.67	1	0.75	0
0	41.75	118.5	0	121.33	0	46.25	0	0
131.33	4	9.5	15	20.33	0.33	41.75	0	0
0	105.5	86.75	28.75	0	0	16	0	0
0	2.25	18.5	22.25	0	3.67	27.5	35.25	0
50.33	13.75	24.25	13.75	9	2.33	27.5	11.75	16
0	0	0	0	0	106.67	0	0	0
0	26.5	11	0	37.33	0	97.5	19.25	10
0	0.75	0	30.25	0	0	0	78.5	0
1	5.5	16.75	7.5	1.33	12.33	39	21.5	13
0	19.75	22	17.75	0	0	17.25	0	52.5
0	60.25	0	22	0	0	4.75	15.5	13.5
0	0.75	0	1	0	0	0	26.25	0
0	0	0	0	0	132	69.75	0.25	0
0	88.5	0	0	0	0	0	0	0
0	3	0	0	0	0	0	0	88.5
0	0	0	22.75	0	0.67	0	25.75	0
48.33	0.25	1.5	1.5	0	4.67	0	0	6.5
0	0	0	1	0	68	7	6.25	2
0	133.5	0	0	0	0	0	0	0
7.33	2	11.5	2.25	2.67	9.67	20.5	3.75	0.5
0.33	7.25	20.75	5.25	0	4.33	4.75	6.5	0
0	0	0	1	0	0	32	0	0
0	0.5	0	0	0	53	0	0	0
0	4.75	58.75	0	8	0	10.75	0	0
32	9.25	0	3.5	6	0	0	6	15
22.33	3.75	0	1.25	21	0	8.75	0	0
12.33	5.75	5.5	4.5	5.67	2.67	4.75	3.75	3.5
0	0	36.5	20	0	0	0	15.25	0
0	0	0	0	0	0	0	0	0
20	4.5	5.5	4.75	14.33	3.33	8	0	0
44.33	0.75	9.25	1.75	0	0.67	10.25	9.5	0
0	0.25	0	7	0	0	5	0	1
42.33	1.75	0	4.5	10.33	0	7	0	0
0	2.75	11.75	0	2	0	3	0	0
0	10	0	0.5	0	4.67	5	76.5	0
0	0	27.25	56.75	0	0	0	0	0
24.33	2	0	3.75	7	0	1.5	2	0.5
0	0	0.75	0.75	0	0	0	0	25
0	45.25	0	0	0	0	0	0.25	0

0	45.75	0	0	0	0	0	0	0
0	0	13.75	0	0	0	0	0	0
0	11.75	0.25	5	0	4.33	0	0.5	2.5
0	24.5	6.25	5	0	0	18.5	4.75	0
0	0.75	1	5.25	3	0.33	9.5	3.75	4
0	0	25.75	0	57	0	0	0	0
65	0.75	2.25	3.5	2.67	3	3	1.25	0
0	1	0	3	0.67	0.33	14	6.5	1
0	0	0	5	0	0	8.75	4	1
1.67	1	11.25	7	10.33	4	1	10	0
13.67	0.5	1	2.75	3.67	5	2.5	0	0.5
23.33	0.75	1.75	0	0	16	6.75	0.5	0
9.67	2	3.5	2.25	0.33	1	0.5	1.25	0.5
0	0	0	0	0	0	0	0	15.5
0	0	0	0	0	0	0	0	1
9.67	3	6.5	2.25	3.67	0	13.75	0.75	1.5
15	0	0	11.75	8	0	0.25	0.25	14
0	1.5	0	5.75	0	0	0	63.25	0
3	8.25	5	6.75	8	2	0.25	6.75	0
0	6.5	3	2.75	3.33	9.33	6.75	6.25	0
16.33	1.75	2	4.25	0.67	1.67	4	0	1
16.33	2.25	3.25	2.5	2	2	1.5	3	3
8.33	5	4.5	0.25	4.67	0.33	0	1.5	0
0	4.25	3.5	5.5	0	0	5	0	5
7.67	0	1.75	1.25	2.33	0	3.75	2.25	0
10.67	1.25	2.75	4	1.33	0	0.75	2.25	0
9	4.5	6.25	2.75	4.33	2.67	1.25	0	1.5
1.33	26	2	3.75	0.33	1	0.75	6.25	0
1.67	0	0	0	0.33	18.33	15.75	0	0
0	0	1.25	1.5	2.67	0	26	0	1.5
16.33	0.5	0.5	2.5	5.67	1.33	3	1.5	1
0	0	0	0.25	0	2.33	0	0	0.5
0	0	5.75	11.5	0	0	0	0	10.5
0	0	0	2	0	205	0	0	0
15.33	0.25	0.75	0.75	4.67	0	0.25	1.25	0
19	4	0.25	1.75	3.67	3.67	1.75	0.5	0
39.67	0	0.5	0.5	0	0	0	1.25	0
0	2	0	0.25	0	0	0	0.25	0
15.33	1.5	0	0.5	1	1.67	1	1.25	1.5
0.33	2.25	0	0	0	2.33	0	0	0
0	9.25	0	0	0	0	0	0	0
0	2	0	0	0	0	2	0	0
0	0.25	3	4.5	1	0	1.75	2.5	3.5
4	2.5	1.25	3	2.33	1	2.75	0.25	2.5
0	3.25	0	1	0	0.33	0	2.25	0
8.67	0.5	2.25	2.25	0.33	0	0	3.25	1
38	0.25	0.25	1	0	8.67	18.25	0	0
6.67	0.75	0	1.25	0.67	1.67	0.75	0.25	0
15.67	0.75	0	1.25	0.33	7.67	2.75	3.75	2.5
0.33	2.5	1.5	1	0.33	6.33	1.5	1.5	1
0	40.33	8	63	8	12.33	201.5	4.5	19
100.33	0	72	79.25	0	0	0	16.5	47.67
0	36	16	8.5	0	22.33	54	0	0
11.33	0	0	0	0	0	0	0	7.33

0	10	1.75	4.75	0	67	42	0	0.33
0	0.67	0	22	0	34.67	0	0	19.67
210	0	0	0	0	0	0	0	0
0	0	0	12	0	16.33	0	0	7
0	0	38.25	21.75	43	0	0	36.75	20.67
62.67	0	0	0.25	0	0	0	0	0
0	0	23.25	0.25	0	0	0	51.75	0
107	0	0	2	0	20	0	0	4.67
0	12.33	0.25	10.75	0	1.67	21.25	0	0
0.33	81.67	0.25	0.25	0	0.33	0	0	0
1	1.33	75	2.5	1	0.67	4.5	24.25	27
0	12.33	0	2.25	0	17	12.75	0	0
74.67	8	0.5	4.75	0	0	31.75	0	0
3.33	0	0	0	0	0.33	0	0	0.33
0.67	0	12.75	5	2.5	0.33	0	2	0.67
0	0	0	0	0	0	0	0	0
1.67	0	0	0.25	0	30	0	0	2
0	0	0	0	0	0	0	0.25	15.33
0	0	13.75	3.25	44.5	0	0	6.25	6.67
19.67	0.33	1.5	2.5	0	0	1.5	0	0
0	27.67	0	0	0	1	2.5	0	0
0	36	10.25	0	0	0	0.5	0.25	0.67
0	7.67	0.75	13	0	3.33	16.5	0	0
0	2.67	49.75	8.5	0	60.67	1.5	0	0
0	0	0.5	3.5	8	0	0.25	6	0
6.33	6.67	0	0	0	0.33	0	0	0
4.67	0	0	0	0	0	0	0	0
0	1	1	4.5	0	0.67	1.25	0	3.33
0.67	0	1.75	1.75	0	2	3.25	0	0
3.33	0	0	6.75	7.5	0.33	0.5	1.25	3.67
0	0	6.25	14.25	0	0	0	0	0
23	0	0	0	0	0	0	0	0.67
4.33	5	0	0	0	3.67	0	0	0
0	5.33	4	0	0	0.33	6	0	0
0	0	9.75	1.75	0	0	0	0.75	0
2	0	0	1.25	0	0.67	0	0	1.33
0	0.67	0	0.25	0	13.67	0.75	0.25	0
0	6.67	0.25	1.5	0	0.67	4.75	0	0
0	0	0.25	3.25	0.5	0	0	0.25	0.67
0.33	11.67	0	0	0	0	6	0	0
21.33	0	0	0	0	0	0	0	0
2	0.33	9.25	0.75	0	1	0.75	0.75	0.67
3.67	0	0.75	0.25	0	0	0	0	0.33
0	0	0.25	1.25	0.5	0	0	2.5	11.67
0	0	0.25	0	0.5	0	0	5.5	2.33
0	0	0	0	0	0	0	0	0
35.33	0.67	0	0.25	0	0.33	1.5	0	0
4.33	15.67	0	0	0	1.67	0	0	0
0	0	0	66	0	0.33	0	0	0
0.33	0	0	0	0	0	0	0	0
13.67	1.33	0	0.75	0	2.33	0.75	0	0.33
0	1.33	0	0.5	0	3.67	4.25	0	0
0	0	0	0.25	0	8.33	0	0	1
75.67	2.33	0.5	0.5	0	0	3.75	0	0

0	11	0.25	0.5	0	2	0.25	0	0
0	0	7	3.25	0	0	1.25	2.25	3.67
0	0	0.25	0.25	0	0	0	0	0
0	0	1.75	1.75	4	0	0	0.5	0.33
0	0	2.25	0.25	0	0	0	1.75	0
0	0	0	0	0	27.33	0	0	0
0	0	0.25	2.5	0	5	0	0	3
2	0	0	0.5	0	1	0	0	1
0	0	0	0	0	0	0	0	0.67
1.67	0	0	0	0	0	0	0	0
48	1.33	2	0	0	0	0	0	0
0	0	0	0	0	0	0	0	1.33
2.33	0	0	0	0	0	0	0	1.67
0	3.67	0	0	0	0.33	1	0	0
1.33	0	0	0	0	0.67	0	0	5.67
0	3	0	0	0	3	0.5	0	0
0	0	0	1.75	0	0.33	1.25	0	0
0.67	0.33	0	5	0.5	0	1.75	0.25	0.33
0	3.33	0	0.25	0	0	3.25	0	0
0	1	0.25	0.5	0	1.33	22	0	0
0	0	1.5	0	16	0	0	1.5	7
0	5	0	0	0	0	0	0	0
0	0	0.25	0.25	9.5	0	0	0	7
0	0	12	2.5	1.5	0	0	0.25	5.33
0	3	0	0	0	0.33	5.75	0	0
0	0	0.25	1.25	0.5	0	0	1.5	0.33
2.67	0	0	0.25	0	0	0	0	0
1.33	0	0	0	0	0	0	0	0.33
0	0.67	0	0	0.5	3.33	1.5	0	0
10.67	1	0	0	0	0	0	0	0
1.33	0	0	0	0	1.67	0	0	0
0	0	3	1.25	13.5	0	0	1.5	3.67
0	0	7.5	0.5	1	0	0.25	4.5	0.33
3.33	0	0	0	0	0.33	0	0	0.33
0	0	0.25	0.25	0	0	0	0	0.67
1.33	0	0	0	0	0.33	0	0	0
0	10	0	0	0.5	0	0.25	0	0
1	0.33	0.25	0	0	0	0.25	0	0.33
0	0.33	0	6.75	0	0	22.5	0	0
8.33	0	0	0	0	0	0	0	0
1.67	0	0	0	0	16.33	0	0	0.33
0	1.67	0	0	0	0	9	0	0

8233	8235	8236	8237	8238	8239	8240	8241	8242
341.25	572	839.75	908.33	324.67	0	498.67	637	524
205.75	175.5	166.75	165	139.33	0	273.33	321.5	181.5
80	95.5	100.25	94.33	67	69	81.67	119.5	97
9.75	1.25	28.25	0	49.67	89.67	0	0	0
180.75	93	118.25	0	279.67	703.33	0	0	0
88.5	60.5	151.5	146	66	0	123	63	113
20.25	0	0	0	23	23.33	0	0	0
111.25	28.5	0	0	0.33	13	0	0	0
0.25	28	112	35	0	0	53	20.5	64
0	0	0.25	0	0	0	0	0	0
13.75	16	14.75	5.67	34.33	0	25.33	0.5	0.5
0	37.5	23.75	26.33	89	0	110.67	91	121
0	0	0	0	33.67	31	0	0	0
66.5	40.25	32.75	0	49.67	232	0.33	0	0
0.25	17.5	78	44.33	8.67	0	57.67	60.5	43.5
24.25	19.25	25.5	32.67	21.33	0	25	9	19
42	0	0	0	0	0	0	0	0
18.5	4.75	0	0.33	1.33	0	0	0	0
0	0.5	25.5	88.33	0	0	102.67	0	0
25	14.25	42.75	34.33	5.33	5.67	19.33	15.5	35.5
26.5	26.25	10.25	0	31.67	90.33	0	0	0
11.5	42.25	0	12.67	13.67	0	0	0	0
0	5.25	576.5	1	0	0	66.33	0	0
0.75	0	0	0	0	0	0	0	0
0	44.25	0	94.33	0.33	0	0	0	0
0.5	0	0	0	0	56.67	0	0	0
0	0.25	156.5	72.67	0	0	82.67	0	0
7.75	3.5	0	0	0.33	35.67	0	0	0
9.5	1.75	0	0	0	0	0	0	0
0	40.75	7	36.67	33.33	0	0	0	0
6.75	1	6.5	35.33	2	0.67	21.33	38.5	29
17.75	8.5	32.5	63.33	18.33	0	30.67	15	36.5
0	0	1.25	0.33	0	0	0	0	0
28.75	0	0	29	2.67	0	0	0	0
0	36	9.25	32.33	24	0	20.67	19	59
3.25	0	0	0	15.67	4	0	0	0
0	0.25	0	0.33	4	8	0	0	0
3	9.5	14.5	12	12	0	9	0.5	0.5
39.25	20.25	0	0	0	0	0	0	0
0	0	117	0	0	0	0	0	0
0.5	4	4.75	3.33	2.33	0	5.67	7	10.5
2.25	1	16.25	4	0	0	3.33	0	26.5
0.25	27.75	0	0	0.67	26.33	0	0	0
0	0	0	0	12	15	0	0	0
0	1.75	1	72	12.33	0	15.67	8	43.5
1	1.5	0.75	47.33	3.33	63.67	0	0	0
21.25	43	0	0	0	0.33	0	0	0
1.5	0	0	0	3.67	7.67	0	0	0
0.5	0.25	0	0	0	27.67	0	0	0
0	23.75	0	52	22.67	0	0	0	0

0	21.75	0	22.33	0	0	0	0	0
2.25	0	17	0	0	5.67	0	0	0
0.25	7.5	0	12.67	6.67	0	0.33	0	0.5
4	20.5	1.5	8.67	3.33	0	7.67	24.5	9
1	7	8.5	2	0	10	2	0	0
0	0	0	0	0	0	0	0	0
2.25	1.5	4.75	3.67	3.33	0	3	0.5	1
9.5	2.5	3	3	2	0	1.33	2	0
1.75	0	0	0	0	0	0	0	0
0	6.75	19.25	9.67	4	0	19.67	10.5	16.5
0.75	0.5	0	0	0.67	0.67	0	0	0
1.5	2.75	0.25	3.67	0.33	0	5	0.5	7
0	9.25	8.75	9	2.33	1	4	1.5	8
0	0	0	0	0	23	0	0	0
0	0	0	0	0	11	0	0	0
4.75	3.75	3.75	1	1	0	0.67	7.5	2
2	0	0	0	0	0.33	0.33	0	0
0	0.5	20	0.67	0.33	0	26.67	0	0
0.25	1.75	12	7	3	0	9.33	5	12.5
2.5	6	12.25	8.67	1	0	9	2.5	1.5
0	2.25	0	0	1.67	4	0	0	0
1.75	0.5	6	4	1.33	5.67	2.33	0	0
1.75	5.5	0	1	0	0	4	3	9.5
3.25	2.75	7.5	1.33	16.33	17.67	0	1	0
0.5	5.5	6.75	3	0.33	0	3	2.5	1.5
0.5	9	2.5	3.67	1	0	1	4.5	5
2.25	5.25	4.5	4.67	2.33	0	3	9	6
0	1.75	5.5	6.33	2.33	0	9.33	0	0
0.25	0.25	0	0.67	0.67	0	0	0	0
2.5	8.5	0.25	0	4.33	9.67	0	0	0
0	0	0	0	0	1	0	0	0
0	0	0	0	0	0	0	0	0
9	2.75	0	0	0	6.67	0	0	0
0	0	0	0	0	0	0	0	0
1.75	1.25	3	3	0.67	0	5	0.5	2
0.25	1.75	4.75	5.67	2.33	0	1.33	0	0
0.25	0	0.25	0	0	0	8	0	1.5
0	1.25	25.25	0.33	2.33	0	0	0	0
0.25	1	1.75	1.67	1.33	0.33	1.33	2	0
0.75	1.5	0	12.67	14	0	0.33	0	0
0	2	0	26.33	35.67	0	0	0	0
0	0.75	0	0	0.67	4.33	0	0	0
1.75	1.5	0.25	0	0	3.33	0	5	3.5
0.25	1.25	5.25	1.67	1.33	4.33	1.67	1	1.5
0.5	2.5	8.5	7.67	0	0	0	0	0
2.5	0	2.75	6	1.33	0.33	0	0	0.5
0	0.25	0	1	0	0	0	2.5	8
0.25	3	6.75	0	0.33	0.67	0	0	0
0.25	0	0	0	0	3	0	0	0
3	5.75	1	3.67	0.67	0	4	0.5	1
30.25	114.33	7	122	8	2.67	37	17.5	30.5
0	0.33	5	0	33	17.67	13	0	0
19.5	54.67	22	30	3.67	12	61	0	3.5
0	0	0	0	0	6.33	0	0	0

33.5	0	24	1.5	43	2.33	109.67	69.5	23
0	0	0.67	0.5	0.33	0.33	1	0	0
0.25	0	0.33	0	0	0	0	0	0.5
0	0	0	0	0	0	0	0	0
14.25	3	1.33	0	2.67	2.33	12	0	0
0.25	0	0.33	0	0	0	0	0	0
0	0	38.67	0	12.67	57.33	1.67	0	0
0	0	0	0	0	0	0	0	0
0	2.67	10	4.5	10	53	0	0	0
3.25	0	0	0	35	0	0	0	0
0.5	3	0.33	13.5	0	0	0	0	3.5
0.25	0	9	4.5	5	14.33	4.33	0	2.5
0	0	4.33	1	0.67	5	2	6.5	10
0	0	0	0	0	17.33	0	0	0
1	1	41.33	0	0.33	16.33	40	0	0
0	0	0	0	0	0	0	0	0
0	0	0	0	0	0.33	0	0	0
0	0	0	0	0	0	0	0	2
8.25	17.33	5.33	0	7.67	7.67	6.33	0	0
0	0	0	3	1	0.67	1.67	1.5	0
0	4	0	3.5	0	0	0	26.5	11
0	0	0.33	0	13.33	0.33	0	0	0
0.5	0	9.33	1.5	1.67	7	14.67	6.5	3
0	0	0	0	0.33	2.67	1.67	1.5	0.5
0.25	54	0	0.5	0	0	0	8	7
0.5	0	2.33	0	0.33	0	0	0	0
0.25	0	0.33	0	0	0	0	0	0
0	0	1	0	0	0	1	0.5	0.5
0	0	4.67	0	9	15	4.67	4	4
2.25	1.67	0	0	0	2.33	1	0	6
0	0.67	3.33	0	6.33	5.67	7	0.5	3
0	0	0	0	0	0.33	0	0	0
1	0	7.33	0	3	1	6.67	0	0
0	0	4.33	139.5	42.67	0	0.67	3	87
0	2.33	14	0	10.67	1.33	15.33	1	0.5
0	0	0.67	0	0.67	5.33	0	0	0
0.25	0	0	0	0.67	0.33	0.33	4.5	0
1.25	0.67	11.33	1.5	1	3.67	0.33	0.5	0.5
0.25	0	0	0	19.67	0	0	0	0
0	0	4	8	0.33	8	54	10.5	2
0	0	0	0	0	1.33	0	0	0
1.5	0.33	0	2	0	2	3.67	0	5.5
0	0	0	0	0	0	0	0	0
16.75	7.67	0	0	0	0.33	0	0	13.5
0	2	0	0	0.33	2.67	0	0	0
0	0	0	0	0	0	0	0	0
0	1.67	0.67	1.5	2	0	0	0.5	1
1.5	0	0.33	0	5	0	0	0	0
0	0	0	0	0	0	0	0	0
0	0	0.67	0	0	0	0	0	0
8	0	2	0	2	0	0	0	0
0.5	1.67	2	0.5	0	0	0	1.5	0
0	0	0	0	0	0	0	0	0
0	0	1	0	0.67	0.33	0.67	0	0

0	2.33	15.33	1	7	2.67	0	0.5	9.5
1	2.33	3.67	0.5	0	7.33	7	0	2.5
0.25	0	0	0	0	0	0	0	0
5.75	10.33	0.33	0	0	0.33	2	0	0
0	0	0	0	0	0	0.33	1.5	0
0	0	0	0	0.33	0	0	0	0
0	0	0	0	0	0.33	0	0	0
0	0	0	0	0	0	0	0	0
0	6.33	5.67	0.5	5.67	0	0	0	0
0	0	0	0	0	0	0	0	0
0	0	0	0	0	0	0	0	0
0	0	0	0	0	0.67	0	0.5	0
0	0.67	0	0	0	0.67	0	0	0
0	4.67	0	1.5	0	0	0	0	0
0	0	0	0	0	0.67	0	0	0
0.25	0	0	0	4.67	0	0	0	0
0	0	0	3.5	1.67	2	0.33	0	1
3	4.67	0	0	0	0	0	0.5	0
0	0	5	0.5	0.67	7.67	3.67	0	0.5
0	0	1	1	0	16	0	0	9
3	8.67	0.33	0	0	0.33	0.33	0	0
0	0.33	0	0	1.67	0	0	0	0.5
11.75	0	0	0	0	0	0	0	0
0.5	0.33	0.33	0	0	2.33	0	0	0
0	18.67	1	0	0	0	0	0	0
0.75	1	3	0	0	3.67	4.33	0	0
0	0	0	0	0	0	0	0	0
0	0	0	0	0	0.33	0	0	0
0.25	0.33	0	0.5	0	0.33	0	0	0
0	0	0	1.5	0.67	0	0.33	0	0
2	0	0	0	0	0	0	0	0
0.75	0.33	0	0	1	0	1.33	0	0
0.25	1.67	0	0	0	0.67	0	0	0
0.25	0	0	0	0	0	0	0	0
0	1	0.33	0.5	0	0.33	0.33	0	0
1	0	0	0	0	0	0	0	0
0	0	0	0	2.67	0	0.33	0	0
0	1.67	0	0	0.67	1.33	0	2.5	4
0	0	7	0.5	0.67	2.33	0.33	0	1
0	0	0	0	0	0.33	0	0	0
1.75	0	0	0	0	0	0	0	0
0	0	8	0	0.67	2.33	0.33	0.5	0

8243	8245	8247	8248	8249	8251	8253	8254	8256
631	619.5	822.5	605	587	600.33	500.33	630.5	225.5
1	642.5	0	0	115	211.67	232.33	34	143.25
99	131	146.5	280	153.67	125	86.33	133.5	44.25
32	1	8	217	166.33	14.67	175	57	23.75
0	0	0	0	0	0	0	0	122.25
0	106.5	0	0	94.67	74.67	51.67	29	30.5
218	31.5	62	43	81	13.67	46.67	33.25	6
0	56.5	95	0	0	105	3.33	140.25	63.5
0	33	0	0	0.33	25.33	120.67	5	25.25
159	1	200.5	148	0	0.67	75	53.5	1.5
0	10.5	0	0	0.67	1	0	0.25	49
0	0	0	0	0	0	19.67	10.75	11.5
0	0	0	0	68.33	0	0	0	0
0	0	0	0	0	0	0	0	178.5
0	5.5	0	0	17.67	34.33	17.33	11	23.5
0	24.5	0	0	21.67	24.67	11	8	12.25
0	0	0	0	0	49.67	43.33	0	23
0	72.5	1	0	14	6.67	23	41.5	15.75
0	44	0	0	133.33	0	0	0	0
0	32	40.5	0	23.67	22	20.33	24.5	8.25
0	0	0	0	0	0	0	0	21
0	1.5	141	0	61.33	4	0.67	3.5	0
0	5.5	0	0	0	0	0	0	0
137	26	89.5	103	1	0.33	32.33	39	0
0	0	0	0	0	0	0	0	0
0	0	0	0	0	0	0	0	0.25
0	24	0	0	0	11.33	0.33	0	7
4	0	4	0	3.67	0	0.33	0	8.75
28	15.5	17.5	17	24.67	11.33	11.33	16	0.5
0	0	0	0	0	0	0	0	0
1	3	3	1	5.67	10.33	4.33	2	7.5
0	3	0	0	2	11	4.67	2	10
0	0	177	0	1	10.67	41	33.75	0.25
0	4	0	0	7.67	26.33	17	0	7.5
0	0	0	0	0	0	0	12	11.75
0	15.5	0	0	11	0	0	0.5	2
0	0	0	0	0.67	0	0	0	0
0	4	0	0	1.33	16.67	8.33	1.75	15.5
0	15.5	0	0	0	8	0	5.25	45
0	0	0	0	0	0	0	0	0.25
0	0	0	0	0	0.33	3.33	1.25	2.5
2	9	0	1	5.67	6.67	0.67	2	2.5
0	0	0	0	0	0	0	0	10
0	0	0	0	0	0	0	0	0
0	0	0	0	0	0	5.33	1.25	8.25
0	0	0	0	1.33	4.33	13.33	0	0.25
0	0	0.5	0	0	0	0	0	29.75
0	18.5	0	0	4	2	0	1.75	0.5
0	0	0	0	0	0	0	0	1
0	0.5	0	0	0	0	0	0	0

0	0	0	0	0	0	0	0	0
0	0	0	0	0	0	0	0	0.5
39	0	0.5	55	10.67	1	0	0.25	0.25
0	15	0	0	2.67	5	11.33	3	0
0	13.5	5	0	13	1.33	1	4.25	3.5
0	0	0	0	0	0	0	0	0
0	2.5	2	12	3	1	4.67	3.25	0.5
0	2	7	0	6.33	5.67	2.33	9	4
0	4.5	15	15	14	5.33	2.67	7	0.5
1	0.5	0	4	3.67	0	0.33	0.25	0
1	0	2	34	0.33	1.67	1.33	3	1
8	0	0	6	0	1.33	1	0.75	0
0	1.5	0	3	6.33	3.67	0	0	0.5
0	0	0	0	0	0	0	0	0
0	0	0	0	0	0	0	0	0
15	6	1.5	5	2.67	4.67	0.33	0.75	2.5
0	6.5	0	0	2.33	5.67	3	5.25	0.5
0	0	0	0	0.33	0	0	0	0.25
2	7	0	2	0.33	1.33	0	1.75	4.25
8	1.5	0.5	9	0.33	1.33	7.67	6.25	0
0	0	9.5	0	5.67	0	4.33	3.5	0
6	5.5	0.5	2	5.33	1	0.33	2	0.5
0	2	0.5	0	10	6.33	0.67	1.25	0.5
0	0	0	1	0	0	0	0	2.5
0	0	0	0	2.33	0.33	1.67	2.5	0.75
0	4	0	0	0.67	0.67	1.67	0.75	0.75
0	0.5	0	0	0	0	0	0	0.5
0	1	0	0	7.33	1.67	1.67	0	0.75
30	0.5	26	10	1	2	9.67	15.25	0.25
0	0	11.5	0	0	0	3.33	5	2.5
7	0	1.5	9	2	1.33	1.67	4.75	0.25
123	0	0	10	9.67	0.33	0	0.25	0
0	0	0	0	0	0	0	0	4
64	0	0	5	2	0	0	0	0
0	3.5	0	0	1.67	0	0.67	1.75	0.25
0	3.5	1	0	3.33	0	0	0.25	0.25
1	1.5	0	0	5	0	0	0.75	0
0	0.5	0	0	0	0	0	0	0
1	2	0.5	1	4.67	0.33	0	0	0
0	0	0.5	0	0.33	2	4.33	0.75	0
0	0	0	0	0	0	0	0	0
0	0	0	0	0	0	0	0	0.25
0	1.5	2.5	9	0	0	2.67	0.25	2.25
0	1.5	5	0	0.33	0	0.33	0.5	1.25
0	0	0	0	7.67	0.67	0.33	0	0.75
0	0.5	0	0	3	0.33	0	0.25	0.5
0	0	0	2	2	0.33	0	0.25	0
0	0	0.5	4	0.33	0.67	1	1.5	0
8	0.5	0.5	4	1.67	0	0	1.75	0
0	2	2.5	8	2.33	1.67	1.33	2	2.5
254	95	89.67	53	18	56.33	5	35.75	100.75
0	63	0	0	4.67	39	0	16	0
0	0	29.67	0	0	0	24	9.5	50.75
0	0	0	0	0	52	0	13.25	0

0	0	0	0	0	0	7	0	0
0	0	29.33	0	21	0	0	0	0
0	0	0	0	0	0	0	0	0
0	0	39	0	17.67	0	0	0	0
0	20.5	0	0	3	21.67	0	12.5	13
0	0	0	0	0	0	0	0	0
0	67	0	0	12	0	0	0	0
0	0	1.33	0	1.33	0	0	0	0
0	0	2	0	0	0	2	4.5	7.25
1	0	0	1	0.33	0.33	0	0	0
4	2	3	0	0.67	3.33	0	2.25	7.75
0	0	0	0	0	0	1	0	0
0	0.5	0	0	1	0	0	0.25	0
0	0	0	0	0	3.67	0	5	0
1	3	0	5	0	2.67	0	3.75	1
0	0	0	0	0	0.33	0	14	0
0	0	1.67	0	0	0	0	3.25	0
21	0	0	17	3.67	77	0	18.25	0
0	11	0.33	0	0	34	0	36.5	8.25
0	0	0	0	0	0	0	0	0
63	0	2.67	0	0.33	0	0	1.25	5
0	0	0.33	0	0	0	0	0	0
0	0	0	0	0	0	4	0	0
0	0	1.67	0	5.67	0	1	0	0
0	12	0	0	0.33	5	0	13.75	0.75
0	0	0	1	0	0	0	0	0
0	0	0	0	0	0	0	0	0
0	0	3	0	4.67	0	0	0	0
0	1	0	0	0.33	0.33	0	1.25	0.5
45	1	0.67	0	0	0.33	0	0.25	0.75
3	0.5	0	0	0.33	0	0	0.25	0
0	0	0	0	0	0	0	1	0
0	0.5	0	0	1.33	0.33	0	0	0
0	0	0	0	0	0	0	0	0
0	5	0	0	0.67	0	0	0	0
0	0	2.67	0	0.33	1	0	2	0
1	0	28	1	31.33	0	0	0.25	0.75
0	0	1	1	0.33	0	0	1.75	0.25
3	2	0.33	2	1	1.67	0	0.25	1.25
0	0	0	0	0	0	0	0	0
0	0	0	0	0	0	0	0.25	0
0	16	1.67	0	1.33	0.67	0	0.5	6.75
0	0	1	0	1	0	2	0	0
1	0	0	0	0	0.33	0	1	11.25
0	0	0	0	73	0.33	0	0	6.5
0	0	1	0	0	0	0	10.25	0
8	0	0	1	0	0	0	1	0.25
0	0	0	0	0	0	0	0	0
0	0	0	0	0	0	0	0	0
45	0	0	40	0.33	0	0	0	0
0	0	0.33	0	0	0	0	0	0
0	0	1	3	0	0	0	0.5	3.25
0	0	1.33	0	1.33	0	0	0	0
0	0	0	0	0	0	0	0	0

4	0	2.33	0	0.67	0	0	1	1
0	3.5	0	0	0	2.33	0	0.25	4
0	0	0	0	0	0	0	0	0
0	12.5	0	0	0	1.33	0	2	0.25
0	0.5	0	0	0.33	0	0	0	0
0	0	0	0	0	0	0	0	0
0	0	2	0	2.67	0	0	0	0
0	0	0.33	0	0	0.33	0	1	0
0	0	0	0	0	0	0	0.25	0
0	0	0	0	0	0	0	0	0
0	0	0	0	0	0	0	0	0.75
0	0	0	0	0	0	0	0	0
0	0	0	0	0	0.33	0	0	0
0	0	0.67	0	0	0	0	0.75	4
0	0	0	0	0	1.67	0	0.5	0
0	0	0	0	0	0	0	4.25	0
0	0	1	0	5.33	0	0	0	0
0	0	5.33	0	0	0	0	0.5	5
0	0	0	0	0	0	0	0	0
0	0	0	0	0	0	0	0	0
0	4.5	0	0	0.33	3	0	2.75	5
0	0	0	0	0	0	0	0	0
0	1.5	0	0	0	1	0	3.5	4.5
0	0.5	0	0	0	1	0	1.25	1.25
0	0	0	0	0	0	0	1.75	4.5
0	2.5	0	0	0	1	0	1.5	2.5
0	0	6.33	0	4.33	0	0	0	0
0	0	0.33	0	0	0	0	3.5	0
0	0.5	6	0	0	0	0	0	0.25
0	0	0	0	0	0	0	0	0
0	0	0	0	0	0	0	0	0
0	0.5	0	0	0	1	0	0.75	0.75
0	3.5	0	3	0.33	2	0	2.25	1.75
0	0	0	0	0.33	0	0	0	0
3	0	0	0	0	1	0	0	0.5
0	0	0	0	1	0	0	0	0
0	0	0	0	0	0	0	0	0.25
1	0	1	0	0	0.33	0	0.25	0
0	0	0.33	0	0	0	0	0	0
0	0	0	0	0	0.67	0	0	0
0	0	0	0	0	0	0	0	0
0	0	0	0	0	0	0	0	0

8258	8259	8261	8262	8263	8264	8265	8266	8268
521.33	607.5	245.33	1101	702	483.5	421	579	442.67
213.33	122.25	250.67	496	368.5	360	161.5	65.5	245.33
204.67	163.5	51	309	586.5	2.5	123	127.75	157
49	0.25	42.67	126.5	264.5	14	1	0.25	4
0	0	0	0	0	0	0	0	0
40	79.25	38.33	162	263	33.5	34.5	78.75	82.33
23	19	0	0	294.5	11	27	31.5	0
64	6.75	0	0	0	53	43	34	0
50.33	56.5	21	246	95.5	0	101.5	44.75	9.67
741	0	0	0	0	3	109.5	71.5	0.67
0.33	3.75	83	4	201	0	7.5	0	89.67
3	3.25	70.33	0	0	0	19.5	34.75	2
0	0	0	0	84	0	0	0	0
0	0	0	0	0	0	0	0	0
46.67	26.5	9.33	176.5	0	0	28	54.5	38.33
8.33	19.5	14.33	21.5	106.5	9	16.5	21	21.67
0	69.25	8	143.5	0	0	0	0	26
0	2.5	8	2	0	144	57	61.25	0
11.33	2.75	0	0	0	0.5	0	19	0
25.67	24.75	8.33	12.5	0	10	11.5	37.25	28.67
0	0	0	0	0	0	0	0	0
170.33	0.25	0	40	0	1	5	2	0.33
22.33	14.5	0	44.5	0	18.5	0	1.75	43.33
20	0.25	0	0	0	0	16	26.75	0
0	0	0	222.5	0	76	0	0	0
0	0	0	0	0	0	0	0	0
15.33	0	0.33	0	0	0	0	17.75	24
0	0	0	0	0	0	0.5	0.5	0
0.33	15	0	0	0	7.5	10.5	4.75	0
0	0	0	49.5	0	115.5	0	0	0
6.33	4.25	7.33	9	97	0.5	4	21.5	6
24.67	10.5	7	41	0	0	9	17.5	13.33
10.67	0	1	0.5	1.5	0	25	55	0.33
0	15.25	18.33	44.5	0	15	0	0.25	7.33
1.33	3.25	3.33	0	0	0	31	43	7
1.67	0.25	0.67	0	173	0	7	3.25	3.67
0	0	0	0	77	0	0.5	0	0.33
21	0.5	2.33	18.5	10	7.5	0	9.5	10.33
0	6.25	0	0	0	6	0	0	0
0	0	0	0	0	0	0	0	0
2.33	2.75	3	22.5	65.5	0	5.5	3.5	3.33
4	4.75	4.33	7	10	6	0	6.5	18.33
0	0	0	0	0	0	0	0	0
0	0	0	0	55	0	0	0	0
12.67	2.5	3.33	0	0	0	8	6	22.67
8	9.5	0.33	4.5	0	0	0.5	28.75	42
0	0	0	0	0	0	0	0	0
0	0.75	0	0	28	14	0	0.5	0
0	0	0	0	0	0	0	0	0
0	0	0	64	0	10	0	0	0

0	0	0	97.5	0	12.5	0	0	0
0	0	0	0	0	0	0	0	0
0.67	0.5	0	28	0	22.5	0	0	0
1.67	6.5	2	5	0	6	0.5	4.25	9.67
2	7.75	0.67	0	0	1.5	10	14.25	13
0	0	23	0	0	0	0	0	0
7.67	4.75	3.33	3	31.5	0.5	1.5	7	1.67
4	4.75	0.67	7	0	4	8.5	6	3.33
4.33	1	0	0	0	0	0	7	0
3.33	8.25	0	0	0	2	7	8.75	0.33
5	0.5	0	0	33	0	0.5	1.75	0
0.67	0	0	0	79.5	0	0	0	2
1.67	2.75	0	5.5	6	0.5	0	2.25	3.67
0	0	0	0	0	0	0	0	0
0	0	0	0	0	0	0	0	0
0.33	1.75	0.67	0.5	38.5	2.5	3.5	3.25	0.67
0	0	0	0	76	0.5	0	0	0
6.67	2.25	13.33	3.5	0	2	0	19.5	0.67
2.33	4.5	1.67	31.5	0	0.5	2.5	5.75	4.33
2.33	4.25	0	10.5	0	5	0	5.75	18.67
0	0	0	0	1.5	0	6.5	2.5	1.67
1.67	4	0.33	0	4.5	0.5	0.5	3.75	0
0	2.75	0.67	1	43.5	3	6.5	2.25	2
0	0.25	0	0	0	0	0	0	0
5	3	1.67	6.5	15.5	0	0	1.5	10
1.33	2.75	1.33	5	23	0	0.5	3.5	10
0	0	0.33	1.5	35.5	1.5	0	0	0
5.33	3.25	2.33	9	0	1	3.5	7.75	8
2.33	0.25	0.33	0	1.5	6	7.5	12.75	0
4.67	0	0	0	0	0	0	7.75	0
2.33	6	0	0	26	0.5	0	2.25	0.33
0	0.5	0	0	0	0	0	0	0
0	0.25	0	0	0	0	0	0	0
0	0	0	0	0	0	0	0	0
0.33	0	2.67	0.5	12	6	3.5	0.25	3
0	0.75	0	6.5	10.5	3	1.5	1	1.67
0	0.25	0	0	7	0	0	0	0
0	0	1	4	0	0.5	0	0	0
2	1.25	0	8.5	9	7.5	0.5	0.75	0.67
0	0.75	0.67	12.5	0	4.5	0.5	1.25	6.67
0	0.25	0	11.5	0	2	0.5	0	0
0	0	0	0	0	0	0	0	0
0	0	0	0	1.5	0.5	0.5	0.25	0
1.33	2.5	0	10	4	1	6.5	4.5	1.67
2.67	6	2.33	3.5	0	8	0	3.25	0.33
0	3	3	5	0.5	4	0.5	3	1.67
0	0	0	0	16.5	0	0	0	0
0	1.5	0	0	14	7.5	0.5	0.75	0.67
0	0.75	0	0	11	0	0	0.25	0
1.33	2.75	3.33	2.5	0	0.5	0	2.25	3.67
77.75	47.75	16.25	0	61.67	0	66	122.5	52.75
7	19	2.5	0	20.33	60.33	0	0	0
0	24.5	6.75	3.33	53.33	0	32	35.5	32.5
31.75	0	33.75	0	20.33	87	0	0	0

0	0	93.25	24	0	0	0	0	28.25
0	11.75	0.25	14	0	0	0	13	32.25
0.25	0	0	0	0	73.67	0	0	0
0	5.25	0	3.33	0	0	0	22	9
7.5	14.75	7.5	0	0	0	0	0	0
0	0	0	80	0	138.33	0	0	0
0	84.25	0	0	0	0	0	0	0
0	2.75	0	7.33	0	59	0	11	17.75
0	4.75	0	0	5.33	0	19	0.5	3
0	0	0.25	1	0.33	2.67	0	0	0
15.75	8.5	6.25	0	5	0	0	58	1
0	0	0	39	0	0	0	0	0
0.25	0.25	0.75	0	0	8	0	0	0
6.75	0	1.75	0	15.33	27.33	0	1	0
3.25	0.75	15.5	0	1.67	126.33	0	0	0.25
0	0	0.25	2.33	6	89.33	0	0	0
2.75	0.25	0.25	0.67	0	4	0	1	1.25
4.25	0.25	0	0	61.33	0	0	0	0.25
16.5	8.75	19.75	0	0	0.33	0	0	0
0	0	0	0	0	7.33	0	0	0
0	6.5	0	0	0.67	0	10.5	0	0.25
0	0	0.25	0	0	0	0	0	0.25
0	0	0	0	0	0	0	0	1
0	0.75	0.75	1.67	0	0	0	2.5	4.5
0.5	2.25	6	0	0.33	1	0	0	0
0.25	0	0	3	0	3	2	0	0
0	0	0	5.33	0	87.67	0	0	0
0	2	0.25	15.67	0	0	0	20.5	10.25
0.75	0.75	0	0	2	11.67	0	0	0.25
9.75	1.25	1.75	0	2.67	16	0	0	0.25
0.25	1.75	0	0	0	0	0	0	0
1.5	0	0.25	0	7.67	9.33	0	0	0
0	0.5	0	9	0	2.33	0	0	0
0	0.25	0	0	0	0	0	0	0
1.25	1	0.25	0	0	0	0	0	0
2.25	0.25	0.75	0.67	3.33	25.33	0	0.5	0.75
0	0.5	0.25	0	0.33	0	0	0.5	0
0	2	0	2.33	2	0	2	1.5	1
0.25	0.25	1.25	0	0	0.67	0	0	0
0	0	0	0	0	0	0	0	0
0	0	0	0	0.33	50.33	0	0	0
0.75	0.25	1	0	1.67	3	1	0.5	0.5
0	1.25	0.75	0	0	0	0	0	6.25
0.75	0	18.5	0	0	0	0	0	0.25
20.75	0.25	0	0	0	0	0	0	0
0	0	0	0	7	0	0	0	22.25
0.25	0	0	0	2	2.67	4	2.5	0.5
0	0	0	0.33	0	0	0	0	0
0	0	0	0	0	0	0	0	0.25
0	0	0	2	1.33	0.33	0	0	0
0	0	0	1	0	5.33	1.5	0.5	0.25
0	2	0.5	0.33	0	0	5	0.5	0.25
0	2	0	0	0	0	0	1.5	4.5
0	0	0	0	0	2	0	0	0

0.5	0	0	0	2	1	3	0.5	0.5
0.5	2.25	8.75	0	0	0	0	0	0.25
0.25	0	0	0	0.33	0	0	0	0
1.75	0.25	1.75	0	0	0	0	0	0
0	0.25	0	0	0	0	0	0	0
0	0	0	44	0	0.67	0	0	0
0	1.25	0	1.67	0	0	0	1.5	3.25
16.25	1	0	2	19.33	3.67	0	0	1
1	0.5	1.75	0	0	0	0	0	0
0	0	0	0	0	12.33	0	0	0
0	0.25	0	0	1	2.67	1.5	0.5	0
0	0	0	0	1.67	55.67	0	0	0
5	0	3	0	2.33	1	0	0	0
0	0	0	0	4	0	1.5	1.5	0.75
4.5	0	0.25	0.33	7	8	0	0	0
0	0	0	0.33	0	0	0	2	0
0	0.5	0	1.67	0	0	0	0.5	2.25
3.75	14.25	0	0	0.67	0	15	0	3.25
0	0	0	0	0	0	0	0	0
0	0	0	0.67	0	0	0	0	0.25
3.5	0.25	2.5	0	0	0	0	0	0
0	0	0	0	0	0.67	59.5	0	0
0	0	0.25	0	0	0	0	0	0
2.25	0.5	0.5	0	0	0	0	0	0
1.25	0	0	0	0.67	0	2	0	0
2	1.5	1	0	0.67	2	0	0	0
0	0	0	0	0	0	1	0	0.25
0.25	0	0	0	0	0	0	0	0.25
0	0	0.25	0	0	0	0	0	0.25
0	0.5	0	0	0	1.67	1	0	0
0	0	0	0	0	0.33	0	0	0
0.5	1	1	0	0	0	0	0	0
0.5	0.25	1	0	0	0	0.5	0	0
0	0	0	0.33	0	4	0	0	0
0	0.25	0	0	0.33	0	0.5	0	0
0.25	0	0	3.33	0	1	0	0	0
0	0.25	0	0.67	0	0.67	9	0	1
0.5	0	0	0	1	0.33	2	0.5	0.25
0	0	0	0.33	0	0	0	0	0.25
0.25	0	0	0	0	1.33	0	0	0
0	0	0	2	0	0	0	0	0
0	0	0.5	0	0	0	0	0	0.25

8269	8270	8271	8272	8274	8275	8276	8277	8278
547	383.25	338	313.25	359.33	517	523.75	940.33	728.5
120.75	118.75	154	244.75	79.33	495.5	58	267.67	207.5
39.75	70.25	50.5	53.25	145.33	140.5	68	264	58.5
28.5	33.75	1.5	7.5	20.33	0	29.75	308	0
0	142.25	0	0	252.67	0	0	0	0
62.5	31.25	20	63.5	15.67	76.5	30.5	256.33	94
15.75	38.5	10	14.25	53.33	18.5	47.5	141.67	0
37	188.75	58.5	144.5	70	46.5	87.75	87	0
20	17	0	55.75	5	0	0	21.67	44
0.75	58.75	67	34.75	218.33	0	65.5	0	0
3.25	0.75	0	2	28.67	18.5	0	114	1
0	15	0	7	13.33	0	0	0.67	0
0	0	0	0	0	0	0	159.67	0
0	102.75	0	0	0	0	0	0	0
11.75	1	0	50.75	7.33	13	0	0	12.5
14.75	4.5	6	13.75	6.33	20.5	17.5	31.33	15
63	0	0	0	0	0	0	0	0
9.75	45	42	2.25	62.33	24	34.75	14	1
0.5	0	0	60.25	0	171	0	0	29.5
7	9.5	6	14.25	7.33	34.5	15.75	12.33	18
0	16.75	0	0	57.33	0	0	0	0
29.25	2.25	23	13	4.67	10.5	21.75	0	29.5
130.75	0	0	0.75	0	0	0	0	21
0	32.25	24	30	48	0	32.5	0	0
64	0	0	0.25	0	0	0	0	78.5
0.25	0	0	0	211.67	0	0	0	0
0	0	0	1.75	0	57.5	0	0	95.5
0	2.5	0	0	9.33	0	0.25	0	0
17	5.5	22	7.5	5.67	7	39.75	6	0
2.25	0	0	0.5	0	0	0	0	47.5
5.75	3.25	0	8	1.33	3.5	8.25	7.67	6.5
2.25	3.5	0	12	8.67	3.5	0	0	4.5
0.25	29	6	27	15.33	0	37.5	0	0
13.25	0	0	5.5	0	0	0	0	0
0	2.5	0	5.25	10	0	0	0	0
1.25	4.25	2.5	5.25	0	0	9.5	5.33	0
0.25	0	0	0	0	0	0	25.67	0
0	4	0	3.75	0	6.5	1	38	0
15.75	36	27	13.75	0	25.5	47.75	32.33	0
0.5	0	0	0	0	0	0	0	0
1	0	0	1.75	0	0	0	25.67	0
3.25	3	0	4.25	0	6	6	10.67	6.5
0	0.75	0	0	0	0	0	0	0
0	0	0	0.25	0	0	0	45.33	0
0	6.25	0.5	0.25	1.33	0	0	0.33	0
0	0.25	0	0.5	0.33	0.5	0	0	0.5
0	31.5	0	0	0	0	0	0	0
4.5	1.5	1	0.5	1.33	4.5	1.75	49.67	0
0	0.25	0	0	0	0	0	0	0
25	0	0	0	0	0	0	0	17.5

37.25	0	0	0	0	0	0	0	105
0	19.5	0	0	114.67	0	0	0	0
13.75	2	0.5	0.75	0.67	0.5	0	1	16
1.75	0	0	2	4	3.5	0	0.67	10.5
0	1.75	3.5	6.75	4.67	16.5	7	0	0.5
0	0	0	0	0	0	0	0	0
2.75	1.25	0	1.25	0	1.5	0.5	7.33	1
2.5	5.75	3	3.25	14.33	4	13.25	0.67	0.5
6.25	5	14	1.25	11.67	0.5	11.5	0	0
0	2.25	0.5	5.25	0	2	0	0.67	1.5
0	2.25	2	0.75	1	0	6	7.33	0
0	0	4	0.5	0.33	0	2.25	29.33	0
5.25	0.25	0	4.75	1.67	3	0.5	17.33	2.5
0	0	0	0	30.33	0	0	0	0
0	0	0	0	0.67	0	0	0	0
1	1.5	0	1.25	5	1.5	2.75	11.67	0
6.5	0	0	0.5	0	0.5	1.5	10.33	0
0	0	0	27.75	0	2	0	0	15
3	1	0	0	1.33	0	1	0	7.5
0.5	1.25	1	3.25	9.33	5	0.75	0	7
0	1.25	0	3.75	3.33	0	0	15	0
0.25	2.75	1	1	2.67	2.5	2	13.33	0
0.75	0	0	2.25	0.33	0	3	15	0.5
0.5	4.25	0	0	5	0	0	0	0
0.5	0.5	0	2	2	2.5	0	7.67	2.5
0.5	0.75	0	3.25	1.67	2.5	0	8	1
1.75	0	0.5	0	0.67	0.5	1	11	0.5
5.5	0	0	0	0.33	2	0.25	0	1.5
1	7.25	3	3.25	11	1	11.75	0.33	0
0	0.75	0	0.25	7.33	0	8.5	0	0
0.75	1.25	2.5	0.75	3	0	3	11	0
0.25	0	0	0	0	0	1.25	1.33	0
0	5.75	0	0	12.67	0	0	0	0
0	0	0	0	0	0	0	0	0
0.75	0.5	2	0	0.33	1.5	1.75	10.67	0
1	0.25	1	0.25	0	3.5	0	3.67	3.5
1	0	1	0	0	0	1.75	7.67	0
0.25	0	0	0	0	0.5	0	0	1
3.25	0.25	0	1.25	0	2	0.25	2.33	6
1.75	0.25	0	0	0.33	0	10	0.33	6
4	0	0	0	0	0	0	0	35
0	0	0	0	0	0	0	0	0
0	2.5	0	0.25	6	0	0.25	0.33	0
0	0.75	1	0.75	1	0.5	0.75	1	5.5
1.25	0	0	2	0	3	0	0	7
2.25	0.5	0	0	1.33	0	1.5	8.33	3.5
0	0	0	0	0	0	0	10	0
2.5	0.5	3.5	0.25	0	0.5	15	5.67	1
0.5	0.75	2	1	0.33	0	3.75	9.33	0
5.75	0.75	1.5	0.75	3.67	2	0.25	0.33	1.5
8.25	13	64	65.33	42.25	97	3	88.75	35
39.25	0	0	0	0	4.33	28.67	0	332
11.25	11.25	36	19.67	68.25	2.33	0	27.5	0
0	0	0	0	0	29.33	0	0	78.25

96.25	32.5	0	0	4.75	0	0	0	0
4.75	25.25	10.5	0.33	40.25	36	65.33	33	0
0.5	0.25	0	0	0	0	0	0	0
16	6	3.5	0	12	45.33	32.33	12.5	0
16	14	11	11.33	0	0	17	0	0
0	0	0	0	0	0	0	0	0
29	0	0	0	0	0	35	0	0
5.25	8.5	7	0	25.5	1.33	18.33	4.75	14.5
0	0	7	2.67	36	1	0	7	0
0	30	0	0.67	0	0.33	0	0.5	0
9	0.75	4.75	3	1	2	2.67	1.5	4.25
0.5	3	0	0	15	0	0	0	0
1.5	0.25	2.25	0	22.5	0	0	0	0
0	0	0.5	0	0	4.67	0	0	17.25
1	0.25	1.75	0.33	0	1.33	0	0	0.75
0	0	0	0	0	2.33	0	0	0.25
0.75	0	0.75	0	0.25	10.67	1.33	0	232
0	0	0	1.33	0	11.67	4.67	0	6.5
9	10.5	4.5	1.33	0	0	4.33	0	0
0.25	0	0	0	4	0	0	0	0
0	0	4	2.67	2	0	32.67	1.5	1.5
0	14.75	0.25	0	0	0	0	0.5	0.5
1.75	2.75	3	0	21.5	0	0	0	0
0	0	3.75	0	1.5	1	24.33	0.25	0
28.75	0.5	9	0.67	0	0	0.33	0.25	23.75
0	2.5	0	0	0	0	0	0.25	0
0	1.5	0	0	0	0	0	0	27
2.75	5.25	1.75	0	5.5	9.33	18.67	1.25	0
0.5	0	2.5	0.67	3.75	2	0.67	1	1.25
0.75	1.5	0	0.33	0	0.33	0	1.25	11
0.25	0.25	0	1.33	0	0.33	2.33	0	0
0	0	0	0	0	0	0	0	4.25
1.75	0.5	0	0.67	0	0	0	0.25	0.5
0	0.75	6	0	0	0	0	0	0
2.5	0	0.25	1.33	0	0	25	0	0
0.75	0	0.25	0	0.25	8.67	2	0	12
0.5	0	0.5	0	0	0	0.33	17	0
1.25	1.5	0.75	0.67	1.25	0	0	1.25	0
1.5	0.5	0	1.33	0	0	0	0.5	0
0	0	0	0	0.25	0	0	0	0
0	0	0	0	0.5	0	0.33	0	4.5
0	0.5	0.5	1	0.75	0.67	0.67	2.5	8.25
1.75	0	0	0	0	0	0	0.75	0
10	18.75	0	3.33	0	0	0	0	0
34	0.25	1.25	0	0	0	0	0	0
0	0	0	0	0	5.67	13.33	0.5	0.25
0	0.25	0.75	2.67	1	0	0	1.75	0
0.5	2	0	0	0	0	0	0	0
0	0.25	0.25	0	0.5	0	2.67	0.25	0
0	0.5	0	5.33	0	0	0.33	4.25	0
1	1.25	1	0	0	0.33	0.33	0.75	1.25
5.75	0	0.25	0.67	2.5	0.33	0.33	4.25	1.75
1.75	3.5	0.25	0	1.5	1	9.33	0	0
0	1	0	0	11.25	0	0	0	0

0.25	0.25	1.5	0.67	1	0	0	2	0
2.5	0	4.25	0	0	0.33	2.33	0	0
0	0	0	0	0	0	0.67	0	0
4.25	0	0.75	0	0.25	0	1.33	0	0
0	0	0	0	0	0	0.67	0	0
0	0.25	0	0	0	0	0	0	0
5.25	1.75	0.25	0	3	8	2.67	0.75	0
10.25	1	0	0	0.75	4	0.33	0	3
3	0	0	0.67	0	0	0.33	0	0
0	0	0	0	0	0	0	0	15.5
0	0	0	0	0.25	0	0	0	0
0.25	0	0	0	0	0	0	0.25	8.5
0	0	0	0	0	0	0	0	2.25
0	0	0.5	1	0.5	0	0	0.75	0
0	0	0	0	0	0.33	0	0	4.25
3	18	0	0	0.25	0	0	0.5	0
0	0.5	0.25	0	0.5	0.33	1	0.5	0
0	0	1.25	0.33	0.25	0.33	0	1.75	0
0	0	0.25	0	0	0	0	0	0
0	0.25	0	0	4.25	0.33	0	0	0
2.75	1.5	3	2.33	0	0	0.67	0	0
0	1	0.25	0	0.25	0	0	0	0
0	10	0.5	0	0	0	0	0	0
1.25	0	0.25	1.33	0	0	0.33	0	0
0	0	0.25	17.67	0.5	1.33	0	1	0
0.25	0.75	1	0	0	0	0.33	0	0.25
0	0	0	0	0	0	0	0.25	0
0	0	0	0	0	0	0	0	22.25
0	0	1	0	0.5	0	0.33	7.5	0.25
0	9.75	0.25	0	0	0	0	0	0.25
1.5	2	0	0	0	0	0	0	0
1.5	0.25	0.25	0	0	0	1	0	0
2	0.75	0.75	1	0	0	0	0.75	0.5
1.5	0.25	0	0	0	0	0	0	0
0	0.25	0	0.67	0.25	0.33	0	0.25	0.5
0	0.25	0	0.33	0	0	0	0	0
0.25	6	0	0	0.5	0	0	0	0
0	0	1.75	0.33	0.5	0.67	0	0.25	0.25
0	0.25	0.25	0	0.25	0	0	0	0.25
0	0	0	0	0	1	0	0	0.5
0.75	3.75	0	0	0	0.33	0	0	0.25
0	0	3.5	0	0	0	0	0	0

8280	8283	8284	8285	8287	8288	8289	8290	8291
802	399.25	484	372	562	566.67	529.75	276.67	751.5
287	157.75	330	159.5	246	418	222.5	70.67	122.5
256	56.5	80	86.75	129.33	35	62	59.33	82.25
0	2.5	54.75	0	10.33	0	1.5	29	73.5
0	120.25	0.25	104.25	0	0	0	101	0
137	42	76.25	59.25	32	63.33	58.5	40.33	38.75
0	9.25	34.5	11.75	31	0	0	24.67	19.5
0	29.5	10.5	53.5	73.33	0	0	289.33	175.5
323	0	24.25	15.25	0	3	27	0	43
0	0	63.75	2.5	56	0	0	81	48.5
2	20.75	0	6	14	12	10.5	0	6.75
0	102.25	0	68.25	0	35	57.5	0	11.75
0	0	0	0	0	0	0	0	9.25
0	73	0	0	0	0	0	0	0
146	7	11.5	12.25	23	23.33	39.75	0	31.25
19	9.75	15	17.75	22.67	14.67	18	11	21.5
0	0	0.25	0	78.33	0	83.25	0	56.5
0	23.75	15.25	26.25	28.67	33	0.25	116	18
273	0.5	39.5	0.25	0	0	9.25	0	0
35	3.25	7.5	10	20	0	16.25	16	15.75
0	14	0	17	0	0	0	32	0
0	7.75	1.5	1	31	5.67	0	50	7.5
186	6.5	40.5	1	0	11	14.75	0	0
0	0	10.25	0	29	0	0	18.33	25.5
0	30.5	47.25	0	0	122.67	0	0	0
0	0	0	31.25	0	0	0	65	0
392	0	16.25	14	0	0	0.25	0	0
0	37.25	0.25	0.5	0.33	0	0	79.33	0.5
0	1.25	24.5	6.75	16	0	0	9.67	3.75
1	82.75	45	0	0	134.33	0	0	0
10	12	4.25	0.5	4.33	0.67	4.25	1	3.75
13	2.25	1.75	6.25	5.67	13.67	18	0	20
5	0	0	1.75	19.67	0	0.5	21.33	42.25
2	0	0.25	0	21.33	0	17	0	20.25
0	3.5	0	6.5	0	13	17.5	0	24.75
0	13.75	1.5	2.5	0	0.33	2	0.67	0.25
0	0	0	0	0	0	0	0	0
9	0	7.25	6.5	12	11	4	0	0.75
0	10.75	8.25	4.5	34.67	0	0	18	0
0	0	0	0	0	0	0	0	0
0	0	0	1.75	1	4.67	15.5	0	3.5
20	0.5	5.5	5.5	3.67	0	2.5	3	0
0	13.75	0	1	0	0	0	21.33	0
0	0	0	0	0.33	0	0	0.33	0
0	2	0	2.5	0	22.67	6.25	0	2.25
2	0	1.25	0.75	1.33	0.33	40.25	0	4.5
0	0.5	0	0	0	0	0	0	0
0	11.25	0	0.25	1.33	0	0	3.33	0
0	0.25	0	26.25	0	0	0	1.33	0
0	4.75	16.75	0	0	33	0.5	0	0

0	4.5	13.25	0	0	55.33	0	0	0
0	0	0	23	0	0	0	1	0
0	7.25	12.5	0.25	1	28	1.25	0.33	0
10	1.5	6	2.5	2.33	5.67	6.75	0	8.5
6	4.25	15.75	3.5	7	0.33	6.25	18.67	2.75
0	0	0	0	0	0	0	0	106.5
4	0.75	7.5	1.75	5.33	2	0.25	1	1
2	1.25	2.5	3.75	4	0.33	3.75	3.67	5.25
0	1.75	2.25	0	3	0	0	8.67	2.75
5	0.5	2.25	2.75	1.67	0	0	0.67	0
0	1.25	0	0.25	6	0	0	2	2.75
0	0	3	0	0.33	0	1.75	0	4.25
10	0.5	1.75	3	0	7.67	2.75	2	0.75
0	0	0	9.25	0	0	0	1.67	0
0	0	0	0.75	0	0	0	32.33	0
0	2.25	1.25	5	0.33	0	5.5	6.33	3
0	0.25	3.25	1	6	0	0	0	0
2	0	0.25	1.25	0	0.67	0.25	0	0
17	3.25	6.75	4.75	2.67	8.33	8.75	0.67	5
27	1.25	2.5	5.25	1	3.33	6.75	4.33	4.25
0	0.25	0	1.5	0.33	0	2.75	5.67	4
12	0.75	3.75	0.75	5.33	4.33	5.25	2.33	0
0	1	0	4.25	1	2.67	2.5	2.67	5
0	4	0	7	0	0	0	13	0
24	3	1.25	4	0.33	1.67	1	0	2.5
42	0.25	0.75	1.5	0	0.33	0.75	0	0.75
0	2.25	0.75	0.75	0.33	2.67	0.75	0.33	0
34	0.75	3.5	0.5	2.33	1	6.5	0	1.75
2	0	8.25	0	3.67	0	0.25	4.33	7.5
0	1.5	0	1	2	0	0	9.33	0
0	0.5	1.5	0.5	4.67	0.67	0	1.67	1.5
0	0	0.25	0	0	0	0	1	0
0	6	0	6.25	0	0	0	3.33	0
0	0	0.75	0	0	0	0	0	0
0	2.5	0.25	1.25	0	1	0.25	0.33	0.5
1	0.5	0.5	1.5	0.33	3.67	1.25	0.67	0.5
0	0	0.25	0	0	0	0	0.67	0
0	0.75	0.25	0	0	0.67	0	0	0
0	2	1.5	0.25	0	4	1	0.67	0
0	0.25	2.75	0	2	1.67	0	0	1
0	4	4.75	0	0	7.33	0	0	0
0	0.25	0	0	0	0	0	0.67	0
0	45.75	0	1	1.67	0	0	3	0.25
5	1.25	3	3.5	0.33	1.33	1.75	2	2.25
12	0.25	2.75	0.5	1	1	2.5	0	3.25
10	0.75	0.75	1.75	0.67	11.67	1.25	0.33	1.25
0	0	0.25	0	0	0	0	0	0
0	1.5	2.5	0	0.67	6	0	1.33	0
0	0.25	3.75	0.25	0	0	0	4.67	0
0	1.75	2.25	2.25	7	2.33	1	1.67	5.25
82.5	47.75	44.67	0	62	0	3	40.33	111.75
42	0	0	57.25	7.33	0	67.75	11.33	0
55.5	5.5	19.33	0	42.33	0	0	25	36.25
0	0	0	8	15	0	38.25	7.67	0

0	5.25	0	0	0.33	34.67	0	0	3.25
0	0.5	16.67	24.75	38.33	14.33	24.75	5.67	0
0	0	0	0	0	0	0	0	0
0	0	7.33	5	20	13.33	6.75	4.33	0
75	0	0	5.25	0	0.33	48.25	0	13.25
0	0.5	0	0	0	0	0	0	0
143	0	0	25.25	0	0	10	0	0
0	0	18.33	8.5	19	10.67	12.5	14.67	0
3	0.25	10.33	3	8.33	0	0	3	8
0	24.25	0	0.25	0	10.33	0	0.33	0.75
41	0.25	1.67	4.5	1.33	0.33	1.25	6	1
0	0	0.33	0	0	0.33	0	0	2
0	0	0	0	0	0.67	0	0	0.5
0	0	0	7.75	1	0	3.75	0	0
0	0	0	0	0	0	7.75	0.33	0.25
0	0	0	1.5	0.33	0	0	159	0
0	0	1.67	0.75	1	0.67	5.5	1.33	0
0	10.5	0	15.25	7.33	0	33.25	3	0
0	0	0	0	0	0	5.5	0	4
0.5	0	0	0	0	0	0	0	0
0.5	2.25	4	0	3.33	0	0	0.67	12.25
0	6.75	0	0	0	25.33	0	0	0.25
0	0.75	0	13.5	0	0	5	0.33	4.25
0	1	39.33	6.5	0	25	0.5	0	0
0	0	0	0.25	0.67	0	3.25	0	0.25
0	0	0	0.25	1.33	3	0	0	0.25
0	0	0	0	0	0	0	0	5.25
0	0.25	14	7.5	5	14.33	3.25	22.67	0
1.5	0	3.67	0	3	0	14.75	2.67	1
0	4.25	0	0	0	0	0.25	0	17
1	0.5	0	0	0	0	1.25	0	0
0	0	0	10.25	1.67	0	0	1.33	0
0.5	1.25	0	0.5	1.67	3.33	2.75	0.33	0.75
0	0	0	0.25	0	0	0.25	0.33	0
10.5	0	0	0	0	0.33	8.5	0	0.25
0	0	0.67	4	1.67	0	2.5	0.67	0
0	18.5	0	0.25	0.67	0.33	0.25	0	0
0.5	2.75	0.67	0.25	0	2	0	3.67	24.75
3	0.25	0	0.25	0	0	1.5	0	1
0	0	0	0	0	0	0	0	0
0	0	0	0.25	0.33	0	0	1.67	0
2.5	0	2	1	1	0	0.75	1	0.25
0	0.25	0	0.25	0.33	0	1.5	0	0
0	0.25	0	0	0	0	1.25	0	12.5
14.5	0	0	2	0	0	0.25	0	0
0	0	49.33	0.25	0.33	0	0	82.67	0
0	1.5	1	0	1.33	0	0	1.33	3.75
0	3.75	0	0	0	4.67	0	0	0
0	0	0.67	0.75	0	0	0	0	0
0	1.5	0	0	1.33	0.67	0	0.33	7.25
0	0.5	0	0.5	0.67	1.67	0	0	1
0	0.25	1	0	2.67	0.33	0	1	13.75
0	0	4.33	4	3.33	2	1.5	3	0
0	0.25	0	0	0	0.67	0	0	0.75

0	0.25	1.67	0	6	0	0.25	0.33	0
0	0	0	0.75	0	0	1.25	0	1
0	0	0	0	0	0	0	90.33	0
19.5	0	0	0	0	0	0.5	0	0.25
0	0	0	0.25	0	0	0.25	0	0
0	0	0	0	0	0	0	0	0
0	0	2	1.5	1.33	0.67	0.75	2.67	0
0	0	0.33	2.5	1.33	0.67	1.5	2.33	0
0	0	0	0	0	0	0.75	0	0.75
0	0	0	0	0.33	0	0	0	0
0	0	0	0	0	0	0	0	0
0	0	0	0	0	0	0.25	0	0.5
0	0	0	0	6.67	0	1.25	0.67	0
0.5	0	1.67	0	4.67	0	0	3.33	0.75
0	0	0	2.75	16.33	0	3.5	0	0
0	0.75	0	0	0.33	2	0	0	0
0	0	0.67	1.25	0.33	3.67	0	0.33	0
1.5	0	0	0	1	0	0.25	1	0
0	0.25	0	0	0	0	0	0	0
0	0.75	0	0.25	0	0	0	0.33	0.25
0	0	0	0	0.33	0	1.5	0	5.5
0	6.5	0	0	0	36	0	0	4.25
0	0.25	0.33	0.25	0	0	0.25	0	0
0.5	0	0	0.75	0	0	1.75	0	3
2.5	0	1	0	2	0	0	1.33	1
0	0	0	0.25	0.33	0	1	0.33	0.25
2	0.5	0	0	0.33	1	0	0	0.5
0	0	0	0.25	0	0.67	0.5	0	0.25
0.5	0.25	0.33	0.25	0	0	0	0.33	1.5
0.5	0	0	0	0	11.67	0	0	1.5
0	0	0	0	0	0	0	0	0
1	0	0	0.25	0	0	2.75	0	2.25
0.5	0.75	0	0	0	0	0.75	0.33	0.5
0	1.25	0	0	0.33	0	0	0	0
0	0.25	0	3.75	1	0	0.25	1.67	1.5
0	0.75	0	0	0.33	0	0	0	1
0	10.25	0	0	0	9.67	0	0	2.25
3.5	0	0.33	0.25	2.33	0	0.25	1.67	0.5
0	3.75	0	0	0	0	0	0	0
0	0	0	0.25	0	0	0	0	0
0	0	0	0	0	0	0	0	0
0	7.5	0	0	0	0	0	0	0

8292	8293	8294	8295	8296	8297	8298	8299	8300
412	652.5	583.75	489	607.33	338.33	647	600.75	423.67
312.25	89	408.25	79.25	237.33	54.67	478	80	69.67
103.25	126.5	69.5	90.25	168.33	35	0	132.25	73.33
2.25	2	56.25	55.5	15.67	0	0	4.5	69.67
0	0	0	402.5	0	184	0	0	0.33
86	41	38.75	39	90.33	60.67	89	66.25	63
59.5	57	7	34.25	0	0	0	33.75	6.33
103.25	82.5	102.5	155.5	0	11.33	0	56.75	48
1	75	47.5	14	56.33	27	0	19.5	17
0.75	66	34.25	30.5	0	0	0	1	1
5.75	0	0	14.5	6.33	0	0	8	0
0	22.5	29	0	15	14	0	27.75	73.67
0	0	2.75	0	0	0	0	1.25	0
0	0	0	0	0	32.33	0	0	0
9.25	38.5	20.25	11	42	89.33	0	25	21.67
17.5	27.5	17	10.5	22.67	17.67	25	10	9
0	0	0	47.5	23.67	0	0	67	0
66.5	0	30.75	130.75	0	0	61	6.75	26
42.5	0	0	0	0	58.67	0	0	0
23.5	43.5	16	15	29.33	14	0	16.25	15.33
0	0	0	76	0	25	0	0	0.33
1	293.5	0.5	1.25	0	0	27	0	0.67
10	0	18.5	0.5	0	34.33	4	0	0
0	134	18.75	19.25	0	0	0	44.25	0
0	0	64.75	0	0.33	0	147	0	0
0	0	0	143.25	0	0	0	0	0
68.5	0	0	0.5	0	14.33	0	1	0
0	0	0	140.75	0	98.33	0	0	90.67
5.5	12	5	14.25	0	0	0	6.25	3.67
0	0	9.75	0	0	0	141	0	0
0.25	5	6.75	5.75	9.33	5	0	1	1
2	30	8.75	2.5	14.67	41.67	0	10.75	9
0	92.5	27.75	0	4.67	0.33	0	0.5	0
0	0	0	18.5	24.33	0	0	56	0
0	27.5	0	0	34.67	23	0	5.5	18
23.25	11.5	1.5	0.25	9.33	0	0	4.75	12.67
0	0	0.25	0	0	0	1	0	0
1	0	4.75	0	0.33	0	24	4.75	1.33
51.5	0	0	17.75	0	46.33	0	10.25	52
0	0	0	0	0	0	0	0	0
0	5	2.25	3.5	13	3.33	0	4	3.33
4	0	3.75	7.5	8.33	1.67	0	0.5	7
0	0	0	6.25	0	18	0	0	0
0	0	0.25	0	0	0	0	0.25	0
0	5.5	8	0	14.33	1.33	0	0	2.33
17.25	61	0.5	4.5	7.33	22.33	0	0.5	36
0	0	0	0	0	50	0	0	0
1.5	0	0	4.75	0	0	0	0.25	1.33
0	0	0	89.75	0	0	0	0	0
0	0	4	0	0	0	41	0	0

0	0	14.25	0	0	0	69	0	0
0	0	0	39.5	0	0	0	0	0
0	2.5	9.5	5.25	0	2.67	26	0	0.67
0.25	0	8.75	3	8	0	4	5.75	4.33
1.75	12.5	2	5	5.67	6	0	8.5	0
61.5	0	0	0	0	0	0	0	0
1	7	3.5	1.5	1.33	1	7	1.25	1.67
4.5	24	6.25	2	2.33	1.67	10	1.5	0
8.25	13	3.75	3.75	0	0	0	2.5	1.67
9	0.5	4	0.25	0.33	21	0	4	0
0	0.5	0.5	2.75	0	1.33	0	11.75	0.33
1	0	0.5	1.5	0	0.33	1	7.75	0
1.5	13	5.75	3.5	1.67	10.67	2	2	0
0	0	0	47.75	0	0	0	0	0
0	0	0	218	0	0	0	0	0
6.75	13.5	2	2.25	10.67	3	0	0.5	1
1.75	0	0	3.5	0	0	0	4.75	0.33
2	0	1.25	0	0	0	0	0.5	0
0.75	1	2.25	2.25	1.67	8.33	4	2.75	7.33
4	2.5	4.25	5.75	1.33	0	5	4.75	0.67
0	2.5	3	6.5	0.33	2.33	0	0.25	0.33
5.5	1.5	1	8.5	1	3.33	0	4.5	2
2.75	0	2	0.75	8.67	2	0	2.25	4.33
0	0	0	19.75	0	3	0	0	4
2.25	1.5	0.75	0.75	2.67	4.67	0	0.75	0.33
1.25	10.5	2.75	1.25	12.67	4.33	0	2.25	1
0	0	1.25	6.25	0	0.33	8	1	0
0.75	6.5	1	1.5	16	10.33	4	0	0.33
2.25	16.5	5	14	0.33	0	0	4.75	0.33
0	7	0	1	0	11	0	0.75	4.33
0.25	2	1.25	5	0	0.67	0	3.5	2
0	0	0	24	0	0	0	1.5	0
0	0	0	23.5	0	9	0	0	0
0	0	0	0.5	0	0	0	0	0
2.75	0	3	0	0.33	6.33	0	1.5	3.33
1.5	1	1.75	0.25	0	0.33	4	0.5	0
2	0	0	2.5	0	0	0	0	2.33
8.25	0	1.25	0	0	0	1	0	0
0	1	2.5	1.75	0	1	6	0	0.33
0.25	2	0.75	1.75	1.33	1	2	14	0
0	0	1.5	0	0	0	14	0	0
0	0	0	0	0	0	0	0.25	0.33
1.5	0	1	17	0	3	0	1.75	0.33
1	5.5	0.25	13.25	0.33	2	3	2	0
6.75	0	2	3	1	0	4	5.25	0
4	0	0.5	0.5	3	4.33	1	1	0
0	0	1.25	0.75	0	0	0	0	0
0	0	2.25	0.25	0	0	0	1.25	0
2	0	1.25	6.5	0	0	0	3	0
1.75	0.5	7.5	8.25	1.33	1	0	0.5	0.33
42	70.33	70.75	0	5.33	189	0	77.5	69.33
56.25	0	0	0	11.33	0	0	9	0
0	0	12	27.25	0	70.33	0	0	0
7.5	0	0	0	12	0	0	52.25	0

18.5	0	3.25	148.75	0	15.67	90	0	0
0.25	0	34.25	18	24.33	0.67	0	0	9.33
0	0	0	0	0	0	0	0	0
0	0	24.5	13.25	1.33	0	0	0	7
38	13.67	0	0	4.67	7.67	0	8	2.67
47.25	0	0	0	0	0	1	0	0
7.75	0	0	0	0	0.33	0	0	0
0	0	14.75	19.25	6	0	0	0	12.33
0	0.33	2.75	0	0	0	0	0.25	0
1.5	0	37.5	19	0	0	59	0.5	0
5.25	17.33	0.75	0	0.33	9	0	9.75	1.33
0	0	0	1.75	0.33	9.33	0	0	0
1.75	0	0.25	0.5	0.33	21	0	0	0
0	0	0.25	0	3	0	0	10	0
0.75	14.67	0	0	2.33	0.67	0	6.5	0.67
1.75	0	0	0	1	0	0	27.5	0
0.5	0	1.75	0	9.67	0	0	0.5	0
0	0	0	0	44	0	0	0	0
12.5	17.33	0	0	1	14	0	11.25	7
0	0.33	0.25	0.5	0	0.33	0	0	0
0	1	0.5	0	0	0	0	0.5	1
0.5	0	15.5	0	0	0	56	0	0
0	0	0	1.5	0	11.33	0	6.25	0
0	0.33	0.75	1.5	1	4.33	0	0	2.67
17.25	2	0	0	0	0	0	15.75	2.33
0	0	0	2.5	0	0	6	0	0
0	0	0	0.25	0	0	0	0	0
0.25	0.33	11.5	57	1	7	0	0	11.67
1.75	4.67	0	0	0	0	0	2.25	2.33
0	5	0	0	1	0	0	4.5	3.67
0	0.67	0	0.5	0	0	0	0	0
0	0	0	0	0	0	0	3.25	0
2.75	0	7.5	1.75	0	0	3	0	0.33
0	0	0	0.25	0	0	0	0	0
2.5	1	0	0	0	0	0	1.75	0.33
0.25	0	3	1	0.67	0	0	0.75	0
0	0	0.25	0	0	0	0	0	0
0.25	4.67	3	1	0	1.67	18	0.75	0
0.25	1.33	0	0	0.33	0.33	0	1	0.33
0	0	0	0	0	7.67	0	0	0
0	0	0.5	0.5	0	0	0	0.25	0.33
0.5	0	0	0	0	0.33	1	1.25	0
0	0	0	0	32.33	0	0	0	0
2	21.67	0	0	0	0.33	0	16.75	10.33
7.5	0.33	0	0	0	0	0	1	0
0	0	0.25	0.25	0	0	0	0	0
0.25	0.33	1.25	5	0	0.33	1	1.25	0
1	0	3.25	0	0	0	5	0	0
0	0	0	0.5	0.33	0	0	0	0
0.25	0	0.5	0	0	0	9	0	0
0.25	0	0.75	2.5	0	1	6	0.25	0.33
0	5	0	1	0	2	0	3	2
0	0	6.5	1.75	1	0	0	0	3
0.25	0	0	0	0	5	0	0	0

0	1	0.25	0.5	0	0.67	26	0	0.67
1.5	1	0	0	0.33	0	0	2.25	0.33
0	0	0	0	103.33	0	0	0	0
1	5.67	0	0	1	2	0	0.5	1.67
0.25	0	0	2	0	0.33	0	0	0
4.5	0	0	0	0	0	65	0	0
0	0	4.25	11.25	0	0	0	0	3
0.25	0	0.25	1.25	0	0	0	0.25	1.33
0	4.67	0	0	23.67	3	0	10.75	0.67
0	0	0	0	0	0	0	0	0
0	0	0	0	0	0	0	0	0
0.5	0	0	0.25	0	0	0	0	0
0	0	0	0	19	0	0	0	0
0	0	0.5	0	0	0	0	0.75	0.33
1	0	0.75	0	0.33	0	0	0	0
0	0	0.75	3	0	9.67	4	0	0
0	0	1.25	0.25	1.33	4.33	0	0	1
0	0	1.5	0	5	0	0	0	0.33
0	0	0	0	0	3.33	0	0	0
0	0	0.25	0.25	0	0	0	0	0
1	9	0	0	0.67	0.33	0	6.5	2.67
5.25	0	8.75	0	0	0	6	0	0
1.25	0	0.25	0	1.67	0.33	0	5.75	0.33
1.5	0	0	0.25	1.33	9.33	0	2.25	0
0	0	0	0	0	0.33	0	0	0
0.25	4.33	0	0	0.33	0.33	0	3	0.67
0	0.33	0	0	0	0	3	0.25	0
0	0	0	0	5	0	0	0	0
0	7	0.5	0	0	0	0	5.75	0
0.5	0	4.75	0.25	0	0	4	0	0
0	0	0	0.5	0	0	0	0	0
0.5	1.33	0	0.25	0	2	0	0	1
0.5	2	0	0	0.33	1.33	0	2	0.67
0	0	0	0	0	0	0	0	0
0	0	2.25	0	0	0	0	0.75	0
0.25	0	0.25	0.5	0	0	1	0	0
2.25	0	3.5	0	0	0	7	0	0
0	0	1.25	0	0	0	0	0.5	1.67
0	0	0	0	0	0.33	0	0	0
0	0	0	0	0	0	0	0	0
0	0	0	0	0.33	0	0	0	0
0	0	0	0	0	1.33	0	0	0

8301	8303	8304	8306	8308	8309	8310	8311	8312
441.75	790.75	873.5	114	282	965.25	523	472	451.25
160.75	186.75	65.5	28.75	0.33	331.75	72	160.67	326.25
97.5	105.75	115.5	81.25	72.67	610.5	151.67	165.33	52
1.25	0	16	116.5	51.67	304.25	21.33	0	0
108.25	0	0	315	190	0	190.33	0	300.5
45.25	117	51.5	35	0	162	25.33	92.33	51.25
4.25	0	31	29.5	20	196.5	24.33	0	0
63	0	177.5	31	33	0	31	0	194.5
32.5	31.75	0	31.5	0	0.75	0	31	59.75
3	0.25	98	0	244	0	67	0	0
1.5	14.25	0	0.5	0	512	111.33	74.67	10.75
2.75	39.5	0	0	0	0	11.33	15.67	76.25
0	0	0	47.75	11.33	67.75	2	0	0
12.25	0	0	37.5	100.33	0	22.67	0	0
15.75	59.5	0	18.5	0	0	49	77.67	18.25
8.25	17.5	8.5	6.25	0	48.25	9.33	24.33	21
51	53.5	0	6	0	0	0	0	0
4	0	45.5	0	28.67	0.25	34.67	0	4
0	0	0	0	0	0	0	7.33	0
10.5	19	31.5	6.75	9.67	0	11	18.67	21.5
22.25	0	0	65.25	49.33	0	16	0	80.5
2	10.25	9	0	2.67	0	0	0.33	2.25
0	87.75	0	0	0	0	0	4.67	8
0	0	47	0	26	0	47.67	0	0
0	51.75	0	0	0	0	0.33	0	53.75
0	0	0	43.75	6.33	0	0	0	146.5
0.25	0.25	0	0	0	0	0	22.67	0
97.5	0	0	56	392.67	0	0.33	0	0
3.75	0	7.5	0.5	5.67	0	3	0	0
0	59.75	0	0	0	0	0	0	45.75
0.25	7.75	13.5	2	0.67	96.75	17	3.33	3.5
8.25	22.25	0	4.75	0	0	6.67	49.33	21
0.75	12.25	14.5	0	8.33	0	33.67	0.67	0
25.5	17.5	0	26.5	0	0	0	2.67	0
0.75	35.25	0	0	0	0	5	12.33	19.5
5	1	13	7.75	0	29	0	0	0
0	0	0	77.5	7.33	41.75	0.33	0	0.25
11	10.5	15.5	0	0	19.75	2.33	13.33	0
9.75	0	8.5	0	0	0	0	0	0.25
0	0	0	0	0.67	0	0	0	0
2	6	0	7.75	5.67	22	6.67	0.33	3.75
3	0.25	14	2.5	0	28.25	4	10	0.25
31	0	0	3	48	0	54.67	0	49.25
0	0	0	4.25	5	57.75	0.67	0.67	0
2.75	25	0	0	0	0	20.33	118	17
0.75	53.5	0	3.25	0	0	0.33	0.33	0
13.75	0	0	0	0	0	0	0	0
5.75	0	0.5	5	8.33	65.25	0.67	0	0.5
5.25	0	0	8.25	10.67	0	0	0	91.75
0	20.5	0	0	0	0	0	0	37

0	54.75	0	0.25	0	0	0	0	9.75
0.5	0	0	9.5	81.33	0	0	0	2
0.25	10.5	1.5	0.25	0	0.5	7.33	0.33	7.5
6	6.25	0	4.75	0	0	1.33	16.67	11
3.75	1	6.5	4.5	1	0	5.67	3	13.5
0	0	0	0	0	0	0	0	0
2.25	2	0.5	2.25	0.33	19.75	1.67	3.67	2
3.75	1.5	6	2.25	2.33	0.5	2.33	0	1.25
2.75	0	6.5	0	1.33	0	5.67	0	0.5
1.5	5.75	0.5	0.75	0	0.5	2.67	4.67	2.5
0.25	0	3.5	0	2	36.75	2.67	0	3.25
1	0.75	0	0	0	21	4	7.67	2
1.5	10.5	0	1	0	9	2.67	0.33	1.5
0	0	0	10.5	9	0	0	0	80.5
0	0	0	24	2.67	0	0	0	0.5
3.25	0.5	1.5	0	0.33	21	5.33	0	2.25
2.5	0	4	0.5	1	34.5	0	0	0
0	2	0	0	0	0	0	2.67	0.5
0.25	9.25	0.5	0.75	0	0	0.33	2.33	2.75
2	2	1.5	0	1.33	0	1	8.67	6.25
3.75	0.75	1.5	5.75	1	11	0.33	0	10.5
3.75	2	0	1	3.67	7	1.67	3	8
1.75	2.75	3	0	0	13.5	0	2.67	4
3	0	0	13.25	7	0	7.33	0	10.75
5.25	3	0	7	0	11	8.67	5.67	2.75
2.5	0.75	0	1	0	14.5	0.67	4.33	0.75
0.25	1.25	0	1.5	0	15.25	3	1.33	9.75
2.25	9.75	0	0.75	0	1	0.33	2.67	0.5
0.75	0	8	0	5.67	0.5	6.33	1	2.25
1.25	0	3	3.25	7.67	0	6	0.33	4.25
2.75	0	3.5	2.25	2.33	8	0.67	0	2.25
0	0	0	0	0	0	1	0	0
6.75	0	0	4	1	0	0	0	13.5
0	0	0	0	0	0	0	0	0
0.75	2	4.5	0	0	8.5	3	1.33	1.75
0.25	2.25	0.5	0	0	25.25	0	0	1
4.75	0	0	0	0	10	0	0	0
0	0.5	0	0	0	0	0	0	1
0	1.25	0	0	0	12.75	0	1	1
1.25	6.5	1	1.5	0	0	0.33	0	0.25
0	7	0	0	0	0	0	0	8
0	0	0	5.5	59.33	0	0	0	0.5
1.25	0.25	0	2.25	1.67	0.5	2.67	1.33	6.25
2	5.75	6	0.75	0.67	3	1.33	0.33	7.75
4	4.25	0	4.5	0	0	0	1.67	0.75
2	1.5	0	1	0	2.5	0	5	0.25
0	0.25	0	0	0	18.25	0	1	0.75
0	1	2	0	3.67	6	6	0	2.25
1.25	0.25	0	0.5	1.67	10.25	2	0	0
0.25	3.25	1	1.75	0	0	1.33	0.67	2
69.25	31	45.5	0	75	0	53.67	41.33	6.25
0	0	0	40.75	12	403	18.67	27.67	60.75
23	31	2	1.5	43.25	0	26.67	0	5
0	0	0	8.25	0.75	288.75	0	0	20

26.75	8	0	0.25	0	0	26.33	1	7
0.25	23.5	0	0	0.25	0	0	1	0
0.25	0	0	0	0	0	0	0	0.25
0	0	0	0	0	0	0	0	0
7	5.25	4	10.75	4.25	0	6.67	11	0
0	0	0	0	0	0	0	0	0
0	0	0	3.5	83	0	7.33	3	6
0	5.5	0	0	0	68	0	0	0
0	2	0	10.25	4.75	0	5	16	4
0	0.25	0	0	0	0	0	0	31.5
0.5	18.75	1	1.25	1.5	5	2.33	3.33	0.5
0.75	0.5	0	0	0	0.25	0	7.67	1.5
0	0	0	0	0.25	0	0	6	3
0	0	0	2	0	130	0	0	14.25
1	1.5	0.5	14	22.25	8	4.33	35.33	19
0	0	0	0.5	0.75	4	0	0	0
0	0.5	0	0	3	22	0	0	0.25
0	0	0	19.25	3	0	0	0	21.25
7.25	2	17.5	12	6.75	0	6.33	11.67	7.5
2.25	0.25	0	0	0	0.25	0.67	1.33	0.5
0.25	2	1.5	0	0.75	0.25	1.33	0	0.25
0	0	0	0	0	0.25	2	0	13.25
1	0.5	0	7.25	0	0	0	4.67	1.75
0.25	0	0	0	0	0	1	0.33	0.25
1.5	1.25	0.5	0	0.25	0.5	1	0	0
0.5	32.25	0	0.5	0	1.25	0	0	0.5
0	0.25	0	0.25	0	46.25	0	0	0
0.25	12.75	0	0	0	0	0.33	0.33	0.25
0	0.5	0.5	7.5	8.75	15.75	3.67	2.67	8
4.25	0.25	0	0	1.5	19.75	21.67	0	11.75
0	0	0	7	7.75	0	5.67	6	8.25
0	0	0	21	0.5	21.25	0	0	0.5
0.75	0.5	0	5.25	0.25	0.5	2.33	3.33	11.25
0.25	0	0	0	0	0	6.33	10.67	2.75
0	0	2.5	5	3.25	0	4.33	9.67	18.75
0	0.25	0	0	0	28.75	0	0	5
0	0	0	0	0.5	0.25	1	3.67	0
0.25	3.75	0	0.25	0.75	0.5	0.67	0.33	1.75
0.25	0	0.5	1	0	0	0.33	17	0.25
0	0	0	0	0	0	19.33	7.67	0
0	0	0	0.75	0	14.5	0	0	0.25
0.25	1.5	1.5	2.5	5	3.75	1.33	1.33	1
0.75	0	0	0	0	13	0.33	0	0
11	0.75	3.5	0.25	0	0	0.33	0.33	0
3.75	0.25	0	0	0	0	0	0	0
0	0.25	0	0	0	3.75	0	0	1
0.75	2.5	0	0	0.25	0.25	0.33	1	3.5
0.5	0	0	0	0	0	0	0	10
0	0	0	0	0	0	0	0	0
0	0	0	0.5	0	0	0	0	3.5
0	1.25	0	0	0	0.5	0.33	0	0.25
0.5	3.25	0	0	0.75	0	3.33	0	0
0	3	0	0	0	0	0	0	0
0	0	0	0	0	0	1.33	0.67	0.75

1	0.25	0	0	1	0	0.67	0.33	2
3	0.5	1.5	23.25	1.25	0	1.33	5.33	20.5
0	0	0	0	6.5	0	0	0	0
0.25	0.5	2	0	0	0	1	2.67	1
0	0	0	0.75	0.25	0	1	0	0.75
0	0	0	0	0	0	0	0	0
0	2.5	0	0	0	0	0	0	0
0	0	0	0	0	3.5	0	0	0
4.75	0.25	0	0.25	2.75	0	1	23	0
0	0	0	0	0	33	0	0	0
0	0.5	3.5	0	0.25	0	0	0	0
0	0	0	0.25	0	7.75	0	0	0.25
0	0	0	0.25	0	46.25	0	0	0
0	1.75	1	0	0.5	0	0.33	0	0
0	0.25	0	0	0.25	7	0	0	1.5
2.75	0.25	0	0	0	0	0.33	0	2
0	0	0	0	0	0	1.67	4.33	0
0	0.25	0	0	0.25	0.5	0	0	0
0	0	0	0	0	0	13	0	0
0.75	0	0	0	0	0	7	0	1
2.5	1.25	1.5	0	2.25	0	0	0	0
0	0.25	0	0	0	0	0	0	0.75
2.75	0	6.5	0	0.25	0	0	0	0.25
0	0	0.5	0.25	4	0	0	2.33	0.75
0	0.5	0	0	0.5	0	0	0	0
2.25	0.5	2	1.5	0.25	3	0.33	0.33	0.75
0	0.25	0	0	0	0	0	0	0
0	0	0	0.25	0	5.5	0	0	0.25
0	0	0	3.5	0	0	19.67	0.33	0
0	0.25	0	0	0	0	0.67	0	0
5	0	0	0	0	0	0	0	0
1	0.75	0.5	2.25	1.5	0	3	0	0.25
2.5	0	3	0	1.25	1	1.33	0	0
0	0	0	0	0	0	0	0	0
0.25	0	3	0	0	32.5	0	2.33	0
1.25	0	0	0.5	0.25	0	0	0	0
0.25	0	0	0	0	0.25	0.33	0	1.5
0	0.75	0	1.75	3	1.5	0	0.33	0.25
0	0	0	0	0	0	0	0.33	0.25
0	0	0	0	0	1.5	0	0	0
4.25	0.75	0	0	0	0	0	0	0
2.5	0	0	0	0	0	1.67	0.67	0.5

8313	8314	8317	8318	8319	8320	8322	8323	8324
323.67	518.75	800	324.33	578	282	667.5	431.67	387.33
99.33	149	318.5	40.33	42	162.33	514	331.33	63.67
32	118	598.75	137.67	137	54	545.25	122.33	0
3	110	289.5	34.33	8.67	0.67	302.75	0	0.67
358	793.75	0	69.67	0	0	0	0	211
25.67	31.5	213.5	30.67	38.67	49.67	257.25	118	0
0	20.25	135.5	25.33	32.67	0	260.25	0	0
21.33	4.25	0	21.33	70.33	0	0	0	2.33
47	15.5	100.5	25.67	6.67	64	247.5	42	27.67
0	39.5	0	52.33	56.33	0	0.25	0	0
16.67	1.5	7.75	0	22	17.67	29.25	5.67	7
136	13.25	0	0	23.67	6.67	0.25	64	54.33
0	29.75	77.75	19.33	0	0	130.5	0	0
0	340.75	0	55	0	0	0	0	55.33
26.33	28	0	36.67	8.33	13	0	21	0
8.67	13.5	54.5	9.33	21.33	9.67	74.5	20.33	5.67
24	83.25	0	0	0	65.67	0	0	0
0	0	0	9.33	25.67	9.67	0	0	0
0	0	0	32.33	0	0	0	17	0
6.33	24	0.75	14	20.67	12.33	7.25	26.33	0
28.33	60.5	0	16.67	0	0	0	0	30.67
0	36.5	3.25	0.33	3.67	0	0	0	0
0	0	0	22.67	0	0.33	0	0.67	0
0	32.5	0	26.33	47.33	0	0	0	0
0.67	0	0	0	0	0	0	0	0
94	0	0	0	0	0	0	0	0.33
0	6	0	6	0	0.33	0	23.67	0
73.33	0	0	0	0	0	0	0	143
1.67	11	0	1.67	3	0	0	0	0
0	0	0	0	0	0	0	0	0
1	3.5	9	5.33	13.67	8.33	18.5	11	0
9.33	5.75	0	2.67	2	10.33	0	7.33	0
0.67	21.75	0.25	22.33	18.67	0.33	0	0	0
23.67	21	0	0	0	12.67	0	0	0
19	0.25	5.75	0	4.67	53.33	0	22.33	36
1.33	7.5	16	5.67	0	0	64.25	0	0
0	19.25	29	2.33	0	0	35	0	0
0.67	3.25	17.75	3.67	4.67	0.33	20	4.67	0
0	0	0	0.33	0	0	0	0	8.33
0.33	0	0	0	0	186	0	0	0
1.67	19.5	17.5	1.33	7	12	23.25	2	0
0.33	0.75	15	8	0.33	3.33	30.5	7.67	0
0	0.75	0	0	0	0	0	0	30.67
0	3.5	50.25	1.67	0	0.33	45	0	0
3.33	11.25	1.25	0	5	40.33	0	9.67	6.33
0.67	7.5	0	20.33	3	0.33	0	0	0
0	0	0	0	0	0	0	0	27
10.67	7.25	7.25	0	0	0	24.75	0	1.33
91.33	0	0	0	0	0	0	0	0
0	0	0	0	0	0	0	0	0

0	0	0	0	0	0	0	0	0
8	13	0	0	0	0	0	0	1.67
3.67	0.25	0	0	0	0.67	0.25	2.33	0.33
0.67	3.25	0	0.33	1.67	10	0.25	8	0
0.33	1.25	0	3.33	6	1	0	3.33	4.67
16.33	0	0	0	0	0	0	0	12
5.67	1	8.75	1	0.33	1	14.5	0.67	0.33
1	6.25	0.75	3	1.67	1.33	0	1.67	0
0	4.75	0	0	10	0	0	0	0
0	0.75	0.5	4	6.33	9.33	0.5	4.67	0
1.67	4	9.75	1.67	4	0	23.75	0	1.33
1.67	0.25	20.25	1	3.33	4.33	27.25	3.67	10
4.67	2	4.25	3.33	0.67	3.67	19	1	1.33
11.67	0	0	0	0	0	0	0	0
26	0	0	0	0	0	0	0	0
0	0.25	18	0	11.33	7	15.5	7.33	0
4.33	0	20.25	0	0	0.67	12.75	0	3
0	0	0	0	0	4.33	0	35	0
2	0.25	0.5	0	2	1	0	1	0
0	2.5	0	5.67	3.33	1	0	3	1.67
0	0.75	18.5	1.67	0	0	23	0	1.67
0.67	0.25	10.25	0.33	0	0.33	13	1.33	7.67
0	2	24.5	0	1	3.67	17	0	0
8.33	31	0	6.67	0	0	0	0	9.33
4.67	1.5	9.75	4.33	0.33	2	16.25	2.33	0
0.67	1.5	8.5	0.67	2	3	15.75	2.67	0
1	1	11.25	0	4.67	1	9	3.33	2.33
1	0.25	0	1	0	0.33	0	1	0
0	5.25	0.75	4.67	11.67	0	0.5	0	0
11	0.25	0	2.67	3.33	0	0	0.33	2
2.33	1	8.25	1	6.67	0	16.75	0	1.67
0	0	0	0	0	0	0	0	0
8	0	0	0	0	0	0	0	10.67
0	0	0	0	0	0	0	0	0
0	0.25	5	0	1.33	5	7	0.33	0
0	0.5	15.25	0.33	0	0.67	25	0.33	0
0	1.75	10.5	0	1.67	1	34	0.67	0
0	0	0	0	0	56.33	0	0	0
0	0.75	14.25	0	0	0	21.25	0	0
0	8	7.25	0	0.33	4.67	0.25	0	1.33
0	0	0	0	0	0	0	0	0
0	134.75	0	1.33	0	0	0	0	2
3.33	0.25	0.5	0.33	0.67	0	1	0	1.67
0.33	1	2	1	0	0	3.5	3	0
0	1	0	1.67	0	1.33	0	1.67	0
0	1.5	3.25	1.33	2	0.67	1	6	5
0	0	17.25	0	4	0	25.75	0.67	4.33
0	1	1.5	2.67	0	0	2.5	0	0.67
0	1.75	11	0.33	0	0	14.5	0	0
5	1.5	1.5	1	2.67	3.33	0	1.67	0.67
4.67	27.5	0	80.67	84	0	0	71.33	105.67
20.67	11.75	78.5	31	19.33	32.67	302.5	21	0
0	29	0	186	40	2.33	0	20	18
60.67	20	129.5	0	0	10.67	124.5	0	0

0	15	0	0	15.33	11.33	0	15.33	29
0	0.25	0	0	0.67	0	0	12	0.33
0	0	203.25	0	0	25.67	56	0	0
0	0	0	0	0	0	0	0	0
0	0	0	6	5.67	2	0	0	0
0	0	232.25	0	0	4.67	16.25	0	0
0.33	16.25	0	19.33	9	2.67	0	18.67	0
0	0	5.75	0	0	0	23.25	0	0
0.33	39.75	0	22.67	30	0	0.25	18.67	27.33
0.33	0	0.25	0.33	0	0	0.5	0	0
19.67	1	1	3.33	17.33	0	17.5	0	0
0	3.25	0	0	9	0.67	0.5	5.33	14.33
0	2	127.75	0.33	11.33	1.33	8	9.67	15.67
4.33	20	23.25	0	0	0	23.75	0	0.33
16.33	4	0.5	9.67	10	19	4	0	0
0	12.25	0	0	0	2	0	0	0
23.33	2	17.5	0	0	0	12.25	0	0
21	4.75	0	0	0	0	0	0	0
0	0	0	7.33	5.33	3	0.25	0	0
0	0.25	16.5	0	0	0.67	6.25	0.67	0.33
2.67	5.5	0	4.67	2.33	0	0	0	5.67
0	13.5	0	0.33	0.67	0	0	1.33	1
1	0.5	0	3.33	5.33	0.33	0	8	45.33
0	1.25	0	2.67	0.33	0	0	2	2.67
53.33	0	0.5	0	0	0	27.75	0	5.33
0	0	15.75	0	0	0.67	1.25	0	0
0	0	4.25	0	0	41.67	24.25	0	0
0	0.25	0	0.33	0	0	0	1	1.67
4	3.75	8.25	8.67	8.67	3.33	24.5	0	6.67
7.67	0.25	6.75	0.33	0.33	0	9.25	0	1
4.33	0	0	6.33	0.67	4.67	0	0	5
1.33	0.25	6	0	0	7.33	14	0	0
0	0	8.75	0	4.67	0	3.75	1	0
0	5	0	0	0	0	0	0	13
0.33	0	0	3	2.67	7.33	0	0.33	0
5.33	0	7	0	0	0	10.5	0	0
0.33	0.75	0	0.67	0.67	0	0	0	0.67
0	0.25	0.5	10.33	9.67	2.67	0	2.67	2.33
0	0.75	0	0.33	0.33	0	0	0	0
0	8	0	0	0	0	0	10.67	17.67
0	0.25	1.25	0	0	1.67	21	0	0
2.33	0.75	4	1	0.33	6.67	16.5	0	3.33
0	0	0	1.33	0	0	0	0	3
5.67	0.5	0	0	0	0	0.25	0	0
0.33	0	0	0.33	0.33	0	0	0	0
0.33	0	0	0	0	0	0	0	0
1	0.5	12	0.67	0.67	0	0.5	0	0.33
0	0	33.5	0	0	0.33	4.75	0	0
0	0	0	0	0	0	0	0	0
0.33	0	0.5	0	0	0	0.25	0	0
1	0.25	37.25	0	0.33	0	1.5	0	0
0	0	0	1.33	14.33	0.33	0	0	0
0	0	0	0	0	0	0.25	0	0
0	0.5	3	0.33	0.67	0	2.5	4.67	2.67

0	1	0	1.67	3.67	0.67	0	2.67	3.67
0	0	0	2	0.33	0	0	0	0
0	0.25	0.5	0	0	0	0.5	0	0
0	0	0	0.67	0	2	0	0	0
0	0	0.25	0	1.33	0	0	2.33	0
0	0	0	0	0	0.33	0	0	0
0	0	0	0	0	0	0	0	0
3.67	1	12.25	0	0	0	10.75	0	0
0	0	0	4.33	8	0	0	0	0
0	0	2	0	0	0.67	6.25	0	0
0	3.25	13.25	1.67	1.67	0	3.25	0	0.33
0	0	6.5	0	0	0	4.5	0	0
1.33	2.25	11.75	0	0	0	8.75	0	0
0	2.5	0	0.33	2.67	0	0	0	0
13	0	9.25	0	0	0	9.75	0	0
0	0	0	0	0	0	0	0	4.33
0	1.5	0	0	0	0	0	11.33	2
0	7	0	0.33	0	0	0	0	0
0	1.5	0	0	3	0	0	12.67	9.67
0	0.75	0	0.33	1	0	0	2	11.33
0	0	0	0	0	0	0	0	0
0	0	0	0	0	0	0.75	0	0
0	0.25	0	0	0	0	0	0	0
0	0	0	1	3.67	1	0	0	0
0.33	1.25	0	1.67	2	0	0	0.33	0
0.33	2	0.25	2	1.67	0	1.25	0.67	0
0	0.25	59.75	0	0	0	0	0	0
0	0	2.75	0	0	0.33	13.25	0	0
0.33	0	0	0	0	0	0.5	0	0
0	0.75	10	0	0	0	2.5	0	0.33
0	0	3.25	0	0	5.33	4.75	0	0
0	0	0	0	1	0	0	0	0
0	7.75	0	0.67	0.33	0	0	0.33	0
0.33	0	2.25	0	0	0.33	0	0	0
5.33	0.25	2.25	0	0.33	0	0.75	0	0
0	0	0.25	0	0	0	11.5	0	0
0	0.25	0	0	0	0	0	0	0
1.67	2.5	0.5	0	0.67	0	4.5	0	2.67
0	0.75	0.75	0	8	0	0	1	1.33
0	0	1	0	0	0	3	0	0
0	0	6.5	0	0	0	0.25	0	0
0	0	0	1.33	0	0	0	4	5.33

Accessions

8325	8326	8328	8329	8332	8333	8334	8335	8336
84.75	272.67	333	463	436.33	345	117.75	107.67	406.67
102	123.33	58	122	32.33	100.5	16	8	51.33
62.75	92.67	126	71.75	79	30.25	65.75	36.67	67
60.25	29.33	110.5	48	354.67	1	51.25	68.33	254.33
344.25	153.33	138	83	139.67	0	394	311.67	162.67
20.75	48.67	34	34.75	12.33	43	28.75	13.33	16.67
16	36.67	36	14	48.33	0	13.75	16.33	33.67
59.5	0	0	25.75	146.33	0	2.75	9.67	12
17.75	26.67	43.5	0	11.33	56.5	7.5	20.67	16
0	0	0	57	174.33	0	0	0	59
18	38.67	68.5	12.5	1	8.75	0.25	0	0
0	25.67	0	2	155.33	112.75	0	58.33	79.33
6.75	17	32.5	27.25	15.67	0	7.25	36.67	8.67
114.25	29	86.5	15.5	24	0	87	20.33	68
0.75	15.67	15.5	2.75	11.33	22.5	13	17	20
2.5	12.67	5.5	17.25	3.33	8.25	4.25	3	5.33
6.5	73.67	0	0	0	16.5	0	0	0
0	0	0	11.5	23.33	0	0	0	23.67
0	0	36.5	0.25	0	0	11.25	0	0
6.25	4.67	9.5	7.75	19.67	8.25	8	5	15.33
61.5	17.33	43.5	19	25	0	113.75	61.33	28
0	0	0	42.5	2	0	0	0	53.67
0	0	0	0	0	0.25	5	0	0
0	0	0	28.5	43	0	0	0	49
0	0	0	52.75	0	0	0	0	0
20	0	0	0	0	0	61.75	44.67	0
2.25	0	73.5	0	0	14.25	8	0	0
4.25	0	0	0	0	0	43.75	68	0
0	0	0	1	5.33	0	1.75	0	7.33
0	0	0	96	0	0	0	0	0
2	7	2	1.5	4	4	6.5	0.33	11
10.75	21	3	5.25	9.33	3.25	2.25	4	11
0	0.33	0	14.5	46.33	0	0	0	14.33
3.75	19	0	0.5	0	0.5	0	0	0
0	15	0	21.75	4.67	29.25	0	6	9.33
7.5	11.67	3	0.5	15.67	0	5.25	3.33	2.67
1.5	11.33	6.5	8.5	8.33	0	4	15.67	14
1.25	9	0	2.5	0	1.75	4	2.33	0
0	0	0	0	0	0	0	0	8.67
0	0	0	0.25	0	142.75	0	0	0
3.5	6.33	0	5	7.67	0	9.25	4.67	5.33
2.5	2.67	10	2	1	2.25	2.25	1	1.67
37.25	0.33	0	0	23.33	0	0	0	0
3.5	1.33	4	7.5	4.33	0.25	4	6.67	0
0	2	0	97	3.67	9	0	3.33	3.67
4.25	13	64	1	0	0.25	2.5	20.33	2.67
0	0.33	0	0	0	0	0	0	0
5.25	0.67	2.5	1	12.33	0.5	10.5	2	7.67
14	0	0	0	0	0	29	0.67	0
0	0	0	26.75	0	0	0.25	0	0

0	0	0	0.25	0	0	0	0	0
19.75	0	0	0.25	0	0	1.75	10.67	0.67
0.5	0	2	14.25	0	0	1	2	0.33
1.25	5.33	6	18.75	1.67	6	0.5	0	0
10	0	5.5	6	1	1	5.25	0.33	0.33
0	0	0	0	0	2.5	0	0	0
0.5	0.67	0	0.5	0.33	0	4.25	0	0.33
0.25	0	1.5	1.5	5.33	2.75	1	0	10
0	0	0	2.25	7.33	0	0	0.33	4.67
0	9.67	6	3	1.67	12.75	3.25	0	12.67
1	0	0	5	2	0	5	0.67	0.33
0	0	0	2.25	4.33	5.75	0	0.33	0
8	1.33	2	2.25	0.33	10.75	4	0	0.33
35.25	0	0	0	0	0	20	35	0
16.75	0	0	0	0	0	77	42	0
0.25	0.67	0	3.25	1.67	3	0	0	3.67
0.25	0	0	0	0	1.25	1.75	0	0
0	0.67	48	0	0	2.25	0.75	0	0
2.5	4.67	5.5	6.25	0	2	2.75	0.67	1
1.25	4.33	3	0.75	12	6	0.75	0	1.67
0.5	1	2	5	6.67	0.25	4.5	6	5.33
1	1.67	2.5	0.5	0	7	2	2	1.33
0	1	0	0.5	0	0	0	0	3
17	11.33	7	10.75	5.67	0	24	7.67	5.33
0.25	0.33	1	0.25	0	2.25	2.25	0.33	0.33
0.5	1	1	0.25	0	3.5	0.75	1.33	0
1.25	1	1.5	3	0.67	1.25	4	3.33	0.33
0	3.67	2	0.25	0	1	0.25	0	0.33
0	0	0	2	6.33	0	0	0	4.67
4.5	5.67	0	0	8.67	3.5	8.5	15	5.67
1.25	0	0	3.25	0	2	1	0.67	2.67
0	0	0	0	0	0	0	0	0
4.75	0	0	0	0	0	14.25	5	0
0	0	0	0	0	0	0	0	0
0	0	0	0	0	1.75	0	1.67	3
0.25	0	0.5	0.25	0	0.5	0.25	0.33	0.33
0	0	0	0.5	0	0.5	0	0	2.67
0	0	0	0.75	0	44	0	0	0
0	0	0.5	2.25	0.33	1	0.25	0	2
0.5	0	0	1.5	0.33	1.75	0	0	0.67
0	0	0	4.75	0	0	0	0	0
0.5	0.33	72	11	0	0	50.75	0.67	25.33
0.25	0	0	0.75	0	0	3	3	0
1.25	0.33	3	1.75	0.33	0	1.25	4.33	2.33
1.75	0	2	0	0	0.75	4.75	0	0
0.75	1	4	1	0.33	5.25	1.25	0.67	3.33
0	0	0	0.25	0.33	0	0	0.67	0
0	0	0	3.75	0.33	0	2.5	0	0
2	2.67	0	1	0.67	0	1.25	1	4.33
0.25	5.33	1.5	2	6	0	1.5	0.67	1
0	54	0	107.75	41.33	23	12.25	32	173
83	0	42	70.75	0	0	86.25	10.67	0
5.33	40.33	0	31.75	23.33	13.25	7.5	24.67	44
14.67	0	0	0	0	21.5	7.5	48.67	0

10	28	0	0	30.33	84.75	1.5	20	22.67
0	11.33	0	0	0.67	0	0.5	0.33	1
0	0	0	0	0	11.75	0	0	0
0	0	0	0	0	0	0	0	0
0.67	0	19.5	0	0	0	25.75	0	0
0	0	0	0	0	143.25	0	0	0
28.67	0	54	5.25	0	0	9	0.33	0
0	0	0	0.25	0	0	0	0	0
5.67	7	0	14.75	7.33	17.75	20.75	41	112.67
0	0	0	0	0	0.25	0	0.33	0.33
0.33	2	71	3.25	2	0	0.5	7.33	1
10	143.67	0	0	10.67	5	2.5	27.67	14.67
0	0.67	1.5	0	4.33	5.25	1.75	8.33	7.67
6.67	0	0	0	0	0	4	52	0
0.33	0	4.5	4.5	0	0	3.5	1.67	0
3.67	0	0	0	0	0.5	3.75	0	0
9	0	0	0	0	0	4	17	0
0	0	0	0	0	0	2	0.67	0
5.67	0	4	8.5	0	0.25	5	0.33	0.33
0	7	0	0	1.33	7.25	0.25	2	1
0	10	0	7	44.67	0.25	0	0	12
0.33	2.33	0	0	1	0.25	0.25	1.33	2
5.67	6.33	0	0	15	1.75	0	36	23.33
0	6	0	0	0.67	0.75	0.25	0.33	2
0	18.33	0.5	1	0.33	0	0.25	0	0.67
23.67	0.33	0	0	0	4	0.25	0	0
2	0	0	0	0	0	0	0	0
0	1.67	0	0	6.33	0.75	0.5	0.67	0.33
39.67	13	15	2.5	0.33	0	1.25	0.33	0
3	2	0	0	0	0	1.25	0	0.33
2	17.67	5.5	8.75	0	0	7.75	0	0
0.33	0	0	0	0	1	2.5	7.67	0
3.33	0	2.5	4.25	0	0	3.25	2.33	0
0	0	0	0	1.33	0	0	3.33	54
3.33	0.33	14.5	2	0	0	6.25	0	0
1.67	0	0	0	0	0.5	1.5	1.33	0.33
0	2	0	0	0	0.25	0.25	0.33	1
2	4	0	4.75	1.67	1.25	2	1	3
24.67	0	21.5	3	0	0	16.25	0	0.33
0	0	0	0	0.33	1	0	1.33	0
0	0	0	0	0	0.5	0.25	0	0
1.67	1.33	18.5	1.5	0	0	7.25	0	0.33
0	0.33	0	0	0	0	0	0	0
0	14	0	0.25	0	0	0	0	0
0	0	0	0.25	0	0	0	0	0
0	0	0	0	0	0	0	0.33	0
0.33	1.33	0	1.25	2	0.25	0.25	0.33	1.33
0	3	0	0	0	0	0	0	0
0	0	0	0	0	0	0	0	0
0.33	0	0	0	0	0	0	0	0
2	0.67	0	0	0	0.75	0.5	0.67	0.33
0	4.33	0	1.5	2	1	0	2	2.67
0	0	0	0	0	0	0	0	0
0	3	0	0.25	1.33	0.75	1.25	2	1.33

0	4	0	3.5	1.33	0	0	2	0.67
3.67	0.33	1	0.5	17.33	3	1.75	0.33	2.33
0	0	0	0	0	0	0.25	0	0
1.33	0	2	0	0	0	1.5	0	0
15	0	1	0.5	0	0	0	0.33	0.33
0	0	0	0	0	0	0	0	0
0	0	0	0	0	0	0	0.33	0
2.33	0	0	0	0	0.25	0.25	2	0
1.33	0	0	0	0	0	2.75	0	0
0	0	0	0	0	0	0.25	0	0
0	0	0	5.75	3.33	0	0	0	0.67
0.33	0	0	0	0	0	0	0	0
0	0	0	0	0	0	0.25	4.67	0
0	0	0	1.5	0.33	0	0	0	7
2	0	0	0	0	0	5.25	1.67	0
0	0.33	0	0	0.67	0.25	0	0	6.67
0	5.67	0	0	1	11	3	7.33	3.33
0	0	0	2.75	0.33	0	0.25	0	1
0	6.67	0	0	2	12.25	0.75	1.33	12
0.33	1.33	0	0	0.33	21.75	2.5	19.33	4.67
0	0	0.5	0.25	0	0	0	0	0
0	0	0	0	0	0	0	0	0
0	0	0	0	0	0.25	0	0	0
2.67	0	4	2	0	0	0	0	0
0	1.33	0	2.5	0	0	0	0	0
5	0	2	0.75	0	0	0.25	0	0
0	0	0	0	0.67	0.5	0	0.33	0.33
3.33	0	0	0	0	0	0	0.33	0
0	0.33	0.5	0	0	0	0	0	0
0	0.33	0	0	0	0	0	0	0
8	0	0	0	0	32.5	0	0	0
0.67	0	2.5	0.75	0	0	0.25	0	0
0.33	0	2.5	0.25	0	0	0.5	0	0.67
0.33	0	0	0	0	0.25	0	0	0
0	0.33	3.5	0.75	0	0	0	1	0.33
2	0	0	0	0	0.25	0	0	0
0	0	0	0	0	0	0	0	0
0.67	0	0.5	2.25	1	0	0.25	0	0.33
0	0.33	0	0	0.33	6.25	0	1.33	2
0.33	0	0	0	0	0	0	0	0
0	0.33	0	0	0	0.25	0	0	0
0	0.67	0	0	9	2	0.25	0	4.33

8337	8338	8339	8340	8341	8342	8343	8344	8345
406	135.33	533.33	395	433.75	827	244.5	851.5	460.5
122	40.67	502	25.67	65.25	210.25	54.5	194.75	233
111	37	313	119.33	96.5	639.5	35.5	515.5	108
0	0	518	69.33	0	423	1	222.75	97
0	23	0	157	792	0	245.5	0	0
70	23.33	145.33	22.67	64	239.5	44.5	160	31
0	0	163.67	40.67	0	238.5	0	157.25	0
0	23	0	67	72.75	0	93.25	0	0
22	7.33	120	14.33	16.5	10	46.75	170	36
0	0	0	75.67	0	0.25	0	0.25	0
26	8.67	205.67	0	29.75	239.5	20	40	18.5
105	35	0	19.67	56.25	0	60.5	1.5	45
0	0	57.67	12.33	0	147.5	0	56.25	0
0	390.67	0	17.67	78.75	0	14	0	0
45	15	0.67	49	29.5	0	37	0	21.5
21	7	35.33	5.67	10.75	51.5	7.5	46.5	18.5
0	0	0	0	0	0	0	0	38.5
0	0	0	7.67	0	0.5	0	0	0
0	0	0	0	17.75	0	0	0	0
8	2.67	4.33	22.33	15	4.5	7.75	8.25	8
0	12.67	0	28.67	87	0	58.25	0	0
0	0	0	0	0	0	0	0	0
0	0	0	0	36.25	0	0	0	0
0	0	0	30.67	0	0	0	0	0
0	0	0	0	0	0	0	0	0
0	1.67	0	0	0.5	0	71.75	0	0
0	0	0	0	37	0	0	0	0
0	0.33	0	0	72.25	0	2.25	0	0
0	0	0	9	0	0	0.25	0	0
0	0	0	0	0	0	0	0	0
9	3	57	4.67	6.25	14.25	12.25	14.25	8.5
29	9.67	0	3.33	16	0	20.25	0	8
0	0	0	36.33	0.5	0	0	0	0.5
0	0	0	0	0.25	0	0	0	27.5
95	31.67	0.33	31.33	22.25	0.25	35.25	0	9
0	0	24	3.33	0	10.75	0.5	37	0
0	0	78.67	0.67	0	36.5	0	26.5	0
0	0	16.67	1	8	15.5	5	22.5	2
0	2.67	0	0	35.25	0	11.5	0	0
0	0	0	0	0	0	0	0	0
13	4.33	15.33	5.67	3.25	29.75	2.75	18.75	3
2	0.67	23.67	1	15	15.75	6.5	22.75	1.5
0	2.33	0	11.67	98.25	0	30.25	0	0
0	0	43	0.33	0	55.5	0	34.5	0
14	4.67	0	8.33	7	0	3.75	0.5	0
0	0	0	8.67	19.25	0.25	0	0	2.5
0	29.33	0	0	176.5	0	11.5	0	0
0	0	20.67	2	0	40	0	13	0
0	0	0	0	0.5	0	8	0	0.5
0	0	0	0	0	0	0	0	0

0	0	0	0	0	0	0	0	0
0	0	0	0.33	3	0	1	0	0
2	1	0	0	0.75	0	2	0	0
0	0	0	4.67	7	0.5	1.75	0	9
0	0.33	0	0.67	20	0	17.5	0	0
0	0	0	0	0	0	0	0	21.5
0	2.33	16	0	3.5	44	0.5	21.5	0.5
0	0	0.33	11.67	0.75	0	1	0	1
0	0	0	7.33	0	0	0	0	0
0	0	2.33	14.67	8.75	1	7.25	3.5	0
0	1	4.33	0.33	3.5	14	1	11.25	0
4	1.33	16	0	1.5	39	3.75	28.25	3
5	1.67	19.67	0.33	5.75	4.5	13.5	21.5	3
0	0	0	0	0	0	32.75	0	0
0	0	0	0	0	0	0.25	0	0
0	0	10	4.33	1.5	9.25	4.5	10	0
0	0	7.67	0.33	0	11	0.25	24	4
0	0	0	0	4.75	0	0	0	0
1	0.33	2.33	0	5.5	0	1.75	0.75	3
3	1	0	3.33	1.25	0.5	0.75	0	0
0	0	12.67	2	0	32.25	1.25	28.25	0.5
0	0	3.33	1	9.75	7.25	3.25	8.25	0
0	0	7.33	1	0.5	21	0.25	18.75	0
0	4	0	4	32.25	0	20.5	0	0
3	1	12	1.33	8	17.5	2.5	11.25	1
1	0.33	25.33	0	2.5	8.25	1	16	0
4	2.67	5.33	2	11	9.75	3.75	16.25	0
3	1	0	0.67	0.5	0.25	0	0	0
1	0.33	0.33	6	0	0.25	0	0	0
0	0	0	4.33	4.75	0	5.5	0	4
0	0	9.67	5	0	9.75	0.25	11	3
0	0	0	0.33	0	0	0	0	0
0	4.67	0	0	33.75	0	7.25	0	0
0	0	0	0	0	0	0	0	0
7	2.33	6.67	0.33	0.5	18	2.5	3	0
0	0	8.67	0	2	17.75	1	15.25	0
0	0	38.33	0	0	18	2.75	28.25	0
0	0	0	0	0	0	0	0	0
0	0	10	0	1.25	16.5	0	11.25	0
0	0	0	0	1	0	0	5.75	1
0	0	0	0	1.75	0	0	0	0
0	0.33	0	0	2.25	0	0	0	0
0	2.33	1.67	0	14.25	0.75	25	0	0
2	0.67	4.33	0	2.5	4.75	3	5.25	0
0	0	0	0	1.75	0	0	0	1
0	0.67	2.67	5.33	3.75	4.75	1.75	2.75	0.5
0	0	22	0	1.75	11.75	3.75	6.25	8
0	0	8.33	2.67	0.25	3.25	1.5	4.75	0
0	0	7	2	0	13.75	0	8	0
0	0.67	0	1	0.5	0	0	0	0
0	132	0	60	22	0	71.5	0	0
25	0	48.25	0	10.5	2	9.25	64.25	14
0	14	0	41	11.75	0	0	0	0
0	0	657.25	0	0	76.5	26.75	126	54

0	21.5	0.75	5.33	8.5	0	0	8.75	0
0	0.5	0	0.67	0	0	0	0	0
0	0	1135	0	0	253	0	48.5	0
0	0	0	0	0	0	0	0	0
38	0	0	0	1.75	0	0	0	0
0	0	108.5	0	0	216	0	9.75	0
75	0	0	0	45.25	0	2.5	0	0
0	0	23.5	0	0	0.25	0	32.25	0
0	0	0	3	3.75	0.25	4.5	0	0
0	76.5	105.5	0	0	0.25	0	51.25	0
0	0	3.5	5.67	3	0	2.25	5.5	0
0	0.5	0.25	3.67	2	0	0	0	0
1	0	138.5	1	6	73.75	2.25	4.25	0
0	0	6	0.33	0	0	3.75	11.25	47
1	0	0.5	0	2.25	0	4	5.75	23
0	0	80.5	0	0	23.75	3	24.5	33
0	0	1	0	0	0	0.25	4	0
0	0	0	0	0	0	3.25	0	6
2	0	0	0	5.25	0	0	0	0
0	0	225.75	0.33	1	67.5	0.5	3	0
0	0	0	1	0.75	0	3	0	0
0	0.5	0.5	0.67	0.25	0.25	1	0.25	0
0	2	0	11.33	4.25	0	0	0	0
0	9.5	0	26.67	2.25	0	0	0.25	0
1	0	0.5	0	5.75	0	8.25	0.75	0
0	3.5	14.25	0	0	13	0	0	0
0	2.5	4.5	0	0	29.25	0	17.25	0
0	0.5	0	1.33	0	0	0	0	0
0	0	1.25	0	10.75	0.25	0.25	8.75	0
0	0	4	0	0.25	0	7.75	2.75	1
3	68.5	0	0	3.75	0	0.75	0	0
0	0	60	0	0	11.25	0	10.5	4
3	0.5	3	0	1.5	0	3	9.75	0
0	0	0	6.33	0	0	0	0	0
4	2	0	0	10.75	0	0.25	0	0
0	0	60.25	0	0	5.5	1.5	6.25	0
0	0	0	0	0.25	0	0.25	0.25	0
0	0	0	0.67	0	0	0	0	0
22	0	0	0	27.5	0	5.5	0	0
0	2.5	0	14.33	0	0	0	0	0
0	0	22.75	0	0	9	0.25	28.25	0
0	0	0.5	2	0.5	0	1.5	4.25	0
0	3	1.25	0	0	0	1.5	0.25	0
0	0	0	0	0	0	5	0.25	1
0	0	0	0	0	0	0	0	0
0	0	0	0	0	0	0	0	0
0	0	2.25	0.33	0.75	6	5.25	1	0
0	0	1.25	0	0	0.25	0	0	0
0	0	0	0	0	0	0	0	0
0	3	70.5	0	0	0.25	0	3.5	0
0	0	1	0	0	1	0.75	2.5	0
0	0	0	2.33	0	0	0.5	0	0
0	0	0	0	0	0	0	0	0
0	0.5	14.5	5.33	0	2.75	0	0.5	0

0	0	0	1.33	0	0	1.75	0	0
0	0.5	0	0	4	0	0	0	0
0	0	0.25	0	0	0	0	0.5	0
0	6	0	0	0.75	0	0.5	0	0
1	18.5	0	0	0.25	0	0.25	0	0
0	0	0	0	0	0	0	0	0
0	0	0	0	0	0	0	0	0
0	0	13	0	0	2	2.25	5	1
12	0	0	0	2	0	0	0	0
0	0	60.75	0	0	14.5	0	3.75	0
0	0	1.5	0	2.75	2.75	2.5	0.75	0
0	0	64	0	0	1.25	1.5	6.5	0
0	0	3.25	0	0	0.25	0	1	0
0	0	0	3	0	0	0	0	0
0	0	7.25	0	0	0	4.25	5	1
0	0.5	0	0	0	0	0	0	0
0	0	0	3.67	0	0	0	0	0
0	0	0	1	0	0	1	0.25	0
0	0	0	2.33	1	0	0	0	0
0	1	0	1.67	0	0	0	0	0
0	0	0	0	0.25	0	0	0	0
0	0	0	0	0	0	0	0	0
0	0	0	0	0	0	0	0	0
0	0	0	0	1.5	0	0.25	0	0
0	0	0	1.33	0	0	0	0	0
3	0	0.75	0	3.25	0	0.25	13.75	0
0	0	0	37	0	0	0	0	0
0	0	1	0	0	0.25	0	1.5	0
1	0	0	0	0	0	0.25	0	0
0	0	2.75	0	0.25	12.25	0.25	1	0
0	0	9.25	0	0	1.5	0	2.5	0
1	2.5	0	0.33	0.75	0	0	0	0
1	0	0.25	0.33	7	0	2	0.75	0
0	0.5	17	0	0	31	0	0	0
2	0	0.25	0.33	0.25	0.25	0.75	0	0
0	3	36.75	0	0	1.75	0	16.25	1
0	0	0	0	0	0	0	0	0
1	0	0	0.33	0	1.25	0	3	0
0	0	0	0.67	0	0	0	0.25	1
0	0	79.5	0	0	0.5	0.25	1	0
0	1	7.75	0	0	15.75	0	0.25	0
0	0	0.25	2.67	2	0	0	0	0

8346	8347	8348	8349	8350	8351	8353	8354	8355
635	279.75	168.67	801.5	321	0	0	525.75	435
257	58.25	0	627	145.33	0	0	247.25	187.5
98	82.5	75	759	38	30.33	0	94.25	86
6	139	73.67	451.5	44.67	73.33	2	43	67.75
0	116.5	452.33	0	244.33	414	446	0	0
72.5	58.75	0.33	302	38.67	0	0	124.25	59
0	13.75	36	173.5	17.67	80.33	0	0	0
0	0	67	0	12	182.67	94	0	0
47.5	34.5	0	147	0	0	0	78.25	30.25
0	0	53	0	0	0	0	0	0
90	1.75	0	0.5	31.33	0	0	262.25	31.25
48	208	0	0	50.33	0	0	72.5	73.25
0	12.25	50.67	118	9	8.33	0	0	0
0	36	55	0	40	61.67	0	0	0
50.5	61.25	0	0	28.33	0	0	73	21.5
27	19.75	0.67	57.5	5	0	0	29.25	11
294.5	0	0	0	0	0	0	160.25	50
0	12.25	22.67	0	0	0	0	0	0
0	18	0	0	0	0	0	0	0
7.5	17.25	14	2	29	2	0	20	12.75
0	44.25	53	0	30.33	154	89	0	0
0	0	1.33	0	0	0	0	0	0
0	0.5	0	0	0	0	0	0	0
0	0	26.67	0	0	0	0	0	0
0	0	0	0	0	0	0	5.75	0
0	0	46.33	0	0	0.33	270	0	0
0	19.75	0	0	0	0	0	0	0
0	0	14.33	0	15.33	5.67	157	0	0
0	0	6.33	0	0	0	0	0	0
0	0	0	0	0	0	0	0	0
11.5	17.25	0	3.5	39.33	0	1	18.75	17.25
6	23.25	0	0	61.33	0	0	49.25	20.75
0.5	0	57	0	0	0	0	2.25	0
19.5	0	0	0	0	0	0	92.25	19.5
20.5	23.25	0	0	49	0	0	35.5	11
0	1.25	0.67	25	4	0	0	0	0
0.5	2.25	52.67	45.5	7.67	4	0	0	0
9.5	5	0	2	22.67	0	0	6.5	8.75
0	0	0	0	31.67	90	0	0	0
0	0	0	0	0	0	0	0.25	0
10.5	4.75	15.67	23.5	6.67	4	0	5.75	9.5
5	6.75	0	17	0	0	0	9	6.5
0	0	27.67	0	12.33	60.67	0	0	0
0	0	4.67	75.5	1.67	9	0	0	0
2	51.25	0	0	1.67	0	0	13	51.25
1	3.5	1	0	0	0.33	0	0.75	0.75
0	0	0.33	0	34.33	65.67	0	0	0
0	1.75	1.33	39.5	0	7	2	0	1
0.5	0	85.67	0	0	0.67	113	0	0
0	0	0	0	0	0	0	0	0

0	0	0	0	0	0	0	0	0
0	0.75	46.33	0	0	1.67	263	0	0
1	1	0	0	2.33	0.67	0	1	0.25
4	4.25	0	0	7.67	0	0	15	11.25
0	0	4.33	0	3.67	6.67	6	0	0
0	47	0	0	0	39.67	0	0	2
1.5	1.25	0.67	19.5	11.33	0	0	1.75	1
1.5	0.75	1.33	0	0.33	0	2	3	3.75
0	0	1.67	0	0	0	0	0	0
13	18	1	0.5	1.33	0	0	10.25	0.25
0	0.5	7.33	20.5	7	0.33	0	0	0
0	7.75	0	29	3	0.67	0	4	3.75
9.5	1.75	0	1	0.33	0	4	6.25	0.5
0	0	10.67	0	0	0	51	0	0
0	0	16.67	0	0	0	70	0	0
4.5	5.75	1.33	11	5.33	0	0	3.5	1.25
0	0	0	7	0	0	0	0	0.25
0	1.75	0	0	0	77.33	0	0	0
4	4.5	0	0	5	0	0	9.5	2.75
2	6.75	2.33	0	0	0	0	3	3.5
0	1	8.67	11	0.33	17	2	0	0
0	1	3	6	0.33	2	1	0.25	0.25
3	2.5	0	28	8.67	0	0	2	3
0	4.75	11.67	0	7	13.67	8	0	0
1.5	4.25	0	11	0.67	0	0	6	4.5
2	3.75	0	12.5	3.33	0	0	7.75	3.25
5	5.75	0	5	6.33	1	1	6	1.5
2.5	4.25	0	0	0	0	0	15.75	14.75
0	0	5	0	0	0	0	0.25	0
3	2.75	3.33	0	2.33	8	0	0.25	1.5
0	1.5	3.67	9.5	2.33	0	3	0	0
0	0	0	0	0	0	0	0	0
0	0	22.33	0	16.67	6	15	0	0
0	0	0	0	0	0	0	0	0
1.5	14.5	0	8.5	3	0	0	2.5	0
0	1.25	2.33	10	3	0	0	0	1.25
0	0	0	8.5	0	0	0	3.5	0
0	6	0	0	0	0	0	0	0
0	2.25	0	12.5	0	0	0	0	0.5
3	0.25	0	2.5	0	0	0	2.25	4.5
0	0	0	0	0	0	0	0	0
0	0	1.67	0	1	44.33	0	0	0
0.5	0.5	2.67	0	3.33	5	2	0	0
0	6	2	8.5	0.33	0.33	1	0	0.25
0	0	0	0	0	2.33	0	12.25	1
0	3.25	2.67	0	3.33	1	1	2.25	5.75
3.5	0	0	19.5	0.67	0	0	3.25	0
0	1.25	1	10.5	2.33	1	1	1	0
0	0	5.33	19	2.33	13.67	2	0	0
3.5	1.25	1.67	0	0.33	0	0	5	0.25
12	27	96	0	68.33	0	0	0	0
0	28.25	25.33	129.67	4.67	0	0	19	48
25.5	1.25	18	0	27.67	0	0	38.25	27
0	0	37.33	137.67	0	17	117	0	2.25

21	15.5	0.67	304.33	17.33	0	0	9	2
0.5	0	0	0	0.33	0	0	0	0
0	0	0	1	0	107	0	0	0.25
0	0	0	0	0	0	0	0	0
0	22.5	0	0	15	0	0	56.75	28.5
0	0	0	0	0	50	1	0	0
0	33.25	0	0	19.33	0	0	10.25	13
0	0	0	70	0	0	0	0	0
17.5	16.75	3.33	0	16	0	1	0	0
0	0	0.33	0.67	0	1	8	0	0.5
0	1	8	1	0	0	0	1.25	1.25
25.5	1.75	1.33	0	30.67	0	0	11	0.75
3.5	4.25	2.67	0	25	1	0	0.25	1
0	0	24	44.67	0	0	18	0	0
0	21	14.33	21.33	0	0	0	0.75	49.25
0	0	5.67	82.67	0	1	5	0	0
0	0	0.33	21.67	0	0	53	0	0
0	0	21.33	0	0	0	1	0	0.25
0	7.75	0	0	1	0	0	5.25	14
1	0	6	1.33	1	30	0	0	0
0	0	3	0.33	1	0	0	0	0.25
1	0	1.33	0	0	0	0	0	0.75
3	2.5	1.33	0	14.67	0	1	47.25	0.5
0.5	0	0	0	1.67	0	0	0	0
0	0	0.33	0.67	0.33	0	0	0	0
0	0	0	13.33	0	2	1	3	1
0	0	0	5	0	0	2	0.5	0.25
0	0.5	0.33	0	0.67	0	0	0	0
0	8	2.33	18.33	1.33	0	0	4	6
0	0	0.33	0	0	0	6	0	0
0	8	0.67	0.33	0	0	0	7.5	13.25
0	0	2.33	52	0	5	4	0	0
0.5	10.25	9.33	4.33	0	0	0	5.5	5.25
1	0	0.33	0	0	0	0	0	0
0	5	0	0	3.67	0	0	4.25	3.75
0	0	1.33	20	0	1	9	0	0.25
0	0	0	0.33	0.67	0	0	0.25	0
1.5	0	2.67	0	0.67	0	0	2.25	0
0	24.25	0	0	7.33	0	0	11.25	3.5
20	0	32.33	0	0.33	0	0	0	0
0	0	0.33	27.67	0	8	3	0	0
0	4.25	0.67	2	0.33	0	0	2.25	8.5
0.5	0.25	0	0	0.33	0	100	0	0.5
0	0	0.67	0	0	0	0	0	0
0	0	0	0	0	0	0	0	0.25
0	0	0	0	0	0	0	0	0
1	0	1	1.67	0.67	0	0	1.25	0.5
0.5	0	0	0	0	0	0	2.5	1.25
0	0	0	0	0	0	0	0	0
0	0	0	0.33	0	6	0	0	0
0.5	0	0	20.33	0	0	0	2.5	2.75
0	0.25	3	0	0.33	0	0	1	2.5
0	0	0	0	0	0	0	0	0
0	0.25	0.33	0	7.67	0	0	0.25	0

4.5	0	1	0.33	0.33	0	0	0.5	0
3	0.25	0	0	1	0	0	2	3
0	0	0	1.67	0.33	0	0	0.25	0
0	1	0	0	0	0	0	1.75	1
0	0.5	0	0	0	0	1	0.75	2.25
0	0	0	2.33	0	0	1	7.75	0
0	0	0.33	0	0	0	0	0	0
0	0	1	10.67	0	2	2	0	0
0	0	0	0	0.33	0	0	0.5	1
0	0	0	1.67	0	0	0	0	0
0	0	0	0	0	0	0	0	0
0	0	0.33	1	0	3	2	0	0
0	0	1.33	1.67	0	0	0	0	0
0	0	3.33	0	0	0	0	0	0
0	0	3.33	3	0	0	15	0.25	0
0.5	0	0	0	0	0	0	0.5	0.25
0.5	1.25	17	0	1	0	0	0	0
0	0	1	1.33	0.33	0	0	0	0
1.5	0.75	2	0	1.33	0	0	0	0
10	0	0	0	0	0	0	0	0
0	0.25	0	0	0.67	0	0	0.25	0
0	0	0.33	0.33	0.33	0	0	0.25	0
0	0	0	0	0	0	0	0	0
0	1.75	0	0	2.67	0	0	0.25	4.5
0	0	3.33	0	0	0	0	0	0
0	0.5	2	1.33	1	0	1	1	5
0	0	0	0	0	0	0	0.5	0
0	0	0	21	0	0	0	0	0
0	0	0	0.33	0.33	0	0	0	0.25
0	0	0.33	0	0	0	0	0	0
8	0	0	0	0	3	0	0	0.5
0	1.75	0	0	0.67	0	0	0.75	3
0	2	0.67	0.33	0	0	10	0.25	0.5
0	0	0	0	0	0	0	32.25	0.5
0	0	1	0	0	0	0	0.5	0.75
0.5	0	0	6.67	0	3	0	2.25	0.25
0	0	0	0	0.33	0	0	0	0
0	0	0.67	0.33	0	0	1	0.25	0
0.5	0	38.33	0.33	0	0	0	0	0
0	0	0	9	0	2	0	0	0
0.5	0	0	0.33	0	1	0	8	0.5
5.5	0	0.67	0	0.33	0	0	0	0

8358	8359	8360	8361	8362	8363	8364	8365	8366
416.25	581.67	1046.75	738.33	205.33	566.33	552.5	903	879
144	172	247.75	65	66	136.67	253.5	407.75	222.25
16	59	263.75	212.33	77.67	136	163	167.5	245.5
15.75	172.67	186.5	333.33	69.67	73.33	63	191.25	164
74	0	0	0	186	0	0	0	0
28.25	105.33	68.5	74.33	44	89.67	62	161.75	83.25
3.25	44	83	162	30	45	39.5	58.75	68.5
5.25	0	19.25	29.33	72.67	0	0	5.75	0
49.5	24.67	33	11.67	0	0	0	33.75	24.5
0	0.33	267.75	108.33	0	1	216	0	0
6.5	0.33	1.75	2.67	0	17.33	68.5	10.75	2
69.75	0	0.25	1	0	0.33	0	0	0
6.5	23.67	15	115.33	6.33	98	0	38	24.5
65.5	0	0	0	0	0	0	0	0
6	28.67	10.75	0	0	8.67	56.5	9.75	16.5
17.5	31	16.75	14	5.67	14	14.5	33.25	31.5
0	97	20.75	0	0	25	42.5	0	50
7.5	0.33	22.75	24.33	32	0	0	55.25	0.75
129.5	0	0	0	0	0	0	134.75	13.25
8	18	65.5	19.67	13.33	32.33	28	21.75	23
8.75	0	0	0	32	0	0	0	0
0	26.67	189.75	0.33	0.33	22.67	0	0.25	37.25
17.25	144	0	0	0	0	0	4.25	5.5
0	0	124.25	26	0	0	74	0	2.5
0.25	164.67	0	0	0	0	0	30.75	0
0	0	0	0	45.33	0	0	0	0
41.5	2.33	0.25	0	0	12.33	0	27.5	0.25
1	0	0	0	17.67	0	0	0	0
2.5	4.33	19.5	10.67	4.33	37.33	1.5	9.5	41.5
0	140.33	0	0	0	0	0	32.25	0.5
2	1.67	4.5	59	0.67	5.33	7	2.75	5.5
1.5	9.67	11	0	0	0.33	10	1.5	6.25
0	0	32.25	30	0	0	1	0	0.5
0	11.33	28	0	0	0	34	3.25	34.75
51.75	0	0	0	0	0	0	0	0
1	4.67	4.25	7.67	0	1	0	9	3.75
0	0.33	0	15	0	6.33	0	4	0
0	18.33	0.75	3.67	6	3.67	7.5	3.25	5
0	0	0	0	0	0	0	0	0.25
0	0.33	0.25	0	0	0	0	0	0
0	5.67	2.25	7	0	6	4.5	4.5	2.75
3.75	1.33	12.25	3.33	2.33	0.67	16.5	5	8.25
31	0	0	0	0	0	0	0	0
0	0	0	16.33	0	1	0	9.75	0
73.5	0	0	0.33	0	0	0	0	0
0	3.33	1	0	0	8.33	1.5	0.25	8.75
39.25	0	0	0	0	0	0	0	0
0.75	1	3	4.67	10	3	0	2	15.75
0.25	0	0	0	43.67	0	0	0	0
0	70	0	0	0	0	0	5	0

0	0.33	0	0	0	0	0	1.25	0
0	0	0	0	45.67	0	0	0	0
0.25	22	9	15.33	4	4	16.5	15.25	9.25
7.75	4.67	5	0	0	5.33	40	1.75	2.5
7.75	0	3	12.33	1.67	1	0	7	2.5
21.5	0	0	0	0	0	0	0.25	0
2.75	11	3.75	5	0.67	1.67	5.5	4.25	1.5
2.5	8	19.5	14.33	3	7	3	6.75	7.75
1	6.33	25.25	22.33	11.33	7.67	4.5	11	33.25
7	0	0.25	2.33	0	0	0.5	8.75	1.25
0.5	0	3.5	19.33	1.33	2	2	4.25	7.5
0.25	0.33	1	0.33	0	0	3	0.25	0.25
8	1.33	2.5	1.33	2	2	8	9	1.5
0	0	0	0	4.67	0	0	0	0
0	0	0	0	0.33	0	0	0	0
0.25	3.33	5.5	9	1	4.33	2.5	4.25	4
9	0	2.5	3.33	0.33	1	0	5	16
1.75	1.67	0	0	0	0	0	10	23
0.25	19.33	1.75	0.33	0	2	2.5	7	2.5
3.25	6.33	1	2	0	0	6	7	1.75
2	3.33	9.5	8.67	1.33	6.67	8	2.5	2
5.25	2.67	6	3	2	17.33	0	4.5	2
0	0.33	1	3	0	5.67	0.5	1	1.75
3.75	0	0	0	3.67	0	2	0	0
4	1	0.5	0	0	3	1	5	1.75
1.5	2.67	0	1.67	0	1.67	3	0.75	1
0	2.33	0	1	3.67	4.67	0	4.25	0
2.75	4.33	1	0.67	0	1.67	3	6	0.25
0	1	24.75	12	1.33	0	5.5	0.5	0.75
3.75	0	1.75	6.67	2.33	0	0	0	0
1	1	6.75	7.67	1	1	1.5	3.5	5.25
0.25	7	30.75	0.33	0	2.33	32	0	0.75
4	0	0	0	7.33	0	0	0	0
0	0.33	0.5	0.33	0.67	1.33	0.5	3.25	3
0.25	2.67	1	0.67	1.33	1	0	0.25	6.75
0.25	3.33	1.5	0.67	0	5	0.5	7	2.5
0.25	1.33	0	4.67	1.67	0.67	3	1.25	2
0	4.33	0	0	0	0	0	0	0
0.5	4.67	1	1	0	2.33	1.5	6.5	2.75
0	3.33	0.5	0	0	3.67	0	1	0
0	28.33	1	0	0	0	0	4	0.75
0	0	0	0	0	0	0	0	0
1.75	0.33	0	0	1.67	2	1.5	0.25	0
0.75	0.67	4	2.33	0.67	2	2	1	0
4.25	0.67	0.75	0	0	0.67	5.5	4.25	12
4	2.33	1.5	0	0	1.67	0	6.5	1.5
0	0	0.5	0.33	2.33	0	3.5	2.25	3.75
0.5	4	4.25	12.67	3.33	2	1.5	1.25	3.5
0	0.33	3.25	2	0	2.33	4.5	0	4
0.25	1.33	3.5	0	2.67	5.67	2.5	2	5.5
30	55.33	58.75	52	0	0	71.5	98.75	0
13.25	27.33	0.25	0	0	33.33	0	0	44.5
39.25	13.67	20	50.67	0.33	0.33	8	32.5	0
0	23.33	0	0	0	8.67	0	0	14.25

31	1.33	21.75	0	11.33	0	183	108.5	0
28.5	68.33	33.75	13.67	29.67	83	0	21.25	89.75
0	0	0	0	0	0	0	0	0
12.25	24.33	33	16	36.67	27.33	0	11.75	60.25
0.5	0	0	11.67	0.33	0	0	7.25	15.25
0	0	0	0	0	0	0	0	0
5.5	0	0	0	0	0	0	0	5
3	19	4.25	0	17.33	3.67	0	1.25	6
0.5	21.67	6.25	17	0	2	0	8.75	0
0	0.33	0	0	6.33	0.67	0.5	0	0.25
0	2	9.5	2.33	0	1	1.5	1.5	1.25
4.75	0	7	0	0	0	5	0.75	0
3	0	0	0	0	0.67	0	1.75	0
0	1	0	0	0	17.67	0	0	25.75
3.75	0	0	4	0	0	0.5	0.5	2.5
0	13.67	0	0	0	0	0	0	29.75
0.75	5.33	0	0.33	2.33	19.33	0	14.5	1
0	0	5.5	0	0	44.33	32.5	0	13.5
1.5	0	0	3.33	0.33	0	0	0.25	0
0	0	0	0	0	0	0	30.25	0
0.5	3.67	2.25	23.33	0	0	0.5	0.25	0.25
0.75	0.33	0	0	0	0	0	0	0.25
7	0.33	2.25	0	0	0	2.5	5.25	6
12	0.33	0	8	0	3	0	1	1.25
0	0.67	0.25	0	0	0.33	1	0	0.25
0	0	0.25	0	0.33	0	2.5	0	0
0	0	0	0	0	0	0	0	0
3.25	4.33	4.75	2.33	12	11.67	0	0.25	3.5
2	0.33	0	4	0	0	0	0	0.75
0	0.33	15.25	4.67	0	0	1.5	0.5	0
1.25	0	0	0	20	0	0	0	0.25
0	0.67	0	0	0	0	0	0	0.25
0.25	0	0.5	0	0.33	0	0.5	0	0.5
0	0	0	0	0	0	0	0	0
1.75	0	0	0	6.67	0	0	0	4.75
6.5	6	0.25	2	0.33	1	0	1	0.75
0.5	0	9.25	0.33	0.33	0.33	28	0	0
0.75	7	2.25	15	0	1.33	0	2	0
16	0	0	0	0	0	0	0	1.5
6.25	0	0	0	0	0	0	0	0
0	0	0.25	0	0.33	0.33	0	0	0
6.5	0.67	2	3	0	0	0	0	0
4.75	0	0	2.33	9	14	0	2.5	1
0.25	0.33	0	0.33	0	0	0	0	0.25
0.25	0	0	2.67	0	0	0	0.5	0
0	0	0	0	1	4	0	0	0.25
0	1.33	0.75	3	0	0	3.5	0.75	0
0	0	1.5	0	0	0	4	0	0
0	0	0	1	17.67	1.33	0	0	6.5
0	0.67	0.5	0	3.67	0	1	0	0
1.5	0.33	2.5	0	0	0.33	0.5	0.75	0.25
0.25	4.33	2	1.33	0	0	1.5	1	0
1	4	0.25	0	6.33	24.33	0	1	6.25
0.75	0	0	0	0	0	0	0	0

0.25	1.33	0.75	0.33	0	0	0.5	1.25	0
0	0	0	0.67	1	0	0	0.75	0.25
0	0	0.25	0	0	0.33	0	0	10.75
0	0	0	1	0.67	0	0	1.25	0.5
0	0	0	0	19	0	0	1	0
0	0	0	0	0	0	0	0	0
0.25	1	0.25	3.67	4	1.33	0	0	6.5
1.25	1.67	0	0.67	0	5.67	0	0.5	1
0	0	0	0	0	0	0	0	0
0	0	0	0	0	0	0	0	0.25
0	0.33	0.25	0.67	0	0.33	0	0	0
0	0	0	0	0	0	0	0	0.5
0	10.33	0	0	0	4	0.5	0	0.75
0	2.33	1.25	4	0	0	0	1.75	0
0	0.33	0	0	0	0.33	0	0	1
0	0	0	0	0.33	0	30	0	0
0.75	1.33	0	2	0	1	0	0.25	0.25
0	0.67	0.25	1	0	0	0	11.25	0
1.25	0	0	0	0	0	0	0.25	0
0	0	0	0	0	0.33	0	0.5	0.25
0	0	0	7	0	0	0	0	0
0	0	0	0	0	0	0	0	0
0	0	0	2.33	0	0	0	1	0
0	0	0	4.33	1.33	0	0	0	0.25
0.25	1.67	0	3	0	0	0	0.5	0
1.5	0	0	0	0	0	0	0	0.5
0	0.33	0	0	0	0	0	0	0
0.25	0.33	0	0	0	0.33	0	0	1.25
0	0	0	0	0	0	0	0.25	0
0	0.33	0.25	0	0	0	0	0.5	0
0	0	8.75	0	0	0	0	0	0
1	0	0	4.67	0.33	0	0	0	0
0.5	0	0	0.67	0	0	1	1.75	0.25
0	0	0	0	0	0	0.5	0	0
0	3	0.25	0	0	0	11.5	1.25	0
0	0	0.5	0	0	0	0	0	0.25
0	0	0	0.67	0	0	0	0	0
1.75	1.67	1	1.33	0	0	0	2	0.5
0.5	0	0	0	0	0	0	0	0
0	0	0	0	0	0	0	0	0.25
0	0	0	0	0	0	3	0	0
1.75	0.67	0	0	0	0	0	0.25	0

8367	8368	8369	8370	8371	8372	8373	8374	8375
430.25	673	693	617.33	422	955.75	533.25	461	446.25
191.75	122.67	102.67	100	136.25	302	166.25	125.5	144.25
28	81.33	160.67	231.67	106.5	574.75	147.75	138.25	105.5
135.75	232	144.67	237.33	108.75	370	65.25	83.5	137.5
138	0	0	0	0	0	0	127.25	0
58	87	182	77.67	56.25	252.75	71.75	87.25	53.5
17.5	120.67	118	65.67	29	260	49	29.75	66.25
45.5	0	0	12.33	0	0	21	64.25	0
8.75	32.67	34.33	22.33	55	60.75	0	9	48.75
0	1	0	180.33	0.5	0.5	443.25	0.5	36.75
13	1.33	119.33	0.33	50.75	204	0	0	1.25
0	0	0	0	0	0	0	0	0
25.25	15.67	63	6.33	25	158	10.75	8.75	18.75
0	0	0	0	0	0	0	66	0
0	11	10.33	0	27.5	0	0	10.25	45.5
16.5	28.33	22	10.33	14.25	68.5	5.5	14	15.75
0	23	0	0	108.5	0	0	0	0
0	1	14.33	31	0	0.5	125.5	45	22.5
0	0	12.33	0	0	0	0.25	15.75	24.25
0.75	12	27.33	7	12.75	8	13.75	17	2.75
31.25	0	0	0	0	0	0	14	0
47.25	0	0.33	0.67	0	3.25	3.5	0.75	0.75
9.25	0	60.67	0	0	0	0.75	1	60
0	0	0	40	0	0	23.75	0.5	33
66.5	0	0	0	0	0	38.75	0	23.5
36.5	0	0	0	0	0	0	0	0
0	0	14.33	0	4	0	0	3.25	1.75
24.75	0.33	0	1	0	0	0	2.75	0
0.25	103	0	5	0	0	9.25	7.25	17.25
126.5	0	0	0	0	0	18.75	0	20.25
3.25	5.67	6	3	11	55.75	0.75	1.25	5.5
0	8.67	4.33	0	5.5	0	0	1.75	3.5
0	0	0	4.33	0	0	9	0.75	0.75
0	58	0	0	19.5	0	0	0	0.75
0	0	0	0	0	0	0	0	0
0.75	5.67	25.33	1.33	7.5	27.75	9.75	4.25	5.5
0.75	1	29	39	8.75	45.75	0.75	0	7
0.5	0.67	3.33	20.67	8.25	29	7	0	2
0	0	0	0	0	0	0.25	0.25	0
0.25	0	0	0	0	0.25	0	0	0
6	21.67	28.33	23	8.75	37	0	0	3.75
0	0	11	0	5	31.5	5.75	8	10.25
0.25	0	0	0	0	0	0	10.5	0
9.5	6	21.67	32	5.75	64.5	0	0	4
0.25	0	0.33	0	0.5	0	0	0	0
0	1.67	0.67	0	0.75	0	0	0.25	0.5
0	0	0	0	0	0	0	41.75	0
1.75	53.33	5	8.33	3	23.5	6	1.25	2.25
24.25	0	0	0	0	0	0	0	0.25
31	0	0	0	0	0	25.5	0	35.75

24.25	0	0	0	0	0	0.75	0	0
7.5	0	0	0	0	0	0	0.25	0
12.75	7	0.33	0	0	0	14	4.75	14.75
8	6	7.67	0	1.5	0	1.5	5.25	3
2.5	2.33	22	9.67	1.25	0.75	0.75	11	5.25
0	0	0	0	0	0	0	0	0
4	4.67	8.67	4.67	1.25	28	8.25	3.75	4.25
2.25	3	2	5.67	2.75	0	9	7.25	2.25
0	20	0	2.67	0	0	11.75	9	9.5
0.25	0	7.33	0.33	0	0.5	0	2.25	34.5
1.25	2	4.67	6.67	1.25	15.25	4.5	2.25	11.5
0.25	0.67	0	0	2.5	28.5	0.25	0	3
4.25	2	7	6.67	2.5	5.25	0.5	0.75	4.25
13	0	0	0	0	0	0	0	0
4.75	0	0	0	0	0	0	0	0
0.75	7	4	6.67	1.25	25.5	3	2.25	4.5
11.25	2.67	1.67	7.33	5	17	4	3.5	4
6	0	34	0	0	0	0	0.25	1.75
2.75	1.33	11	0.67	9.75	0.5	2.25	1.5	9.25
1.75	0.67	2	0.67	0.75	0.25	0.5	1.75	3
5.5	3.67	5	2	1.25	20	1.5	3.25	2.75
3	0.33	9.33	2	2	17.75	2.25	7	3.5
1	1	6	2.67	2	21.5	1.25	1.25	0.75
4.25	0	0	0	0	0.5	0	3	0
0.75	1.67	5.67	1.67	4	6.75	0	0.75	7.25
1.25	0.33	5	2.33	5	15	0	2.5	3.25
5.75	9.33	6.67	10.33	2.5	22	1.25	1.25	3.5
1.25	11.67	12	1	4	0	1	0	8.5
0.25	0.67	0.33	6	0	0.5	4.5	0.5	6
7	0	0.33	0.67	0	0	6.5	0.75	0
2.5	3	3.67	3.33	1.75	15.5	3.25	1.5	1.25
0	0	0	0	0	0	0	0	107
8.75	0	0	0	0	0	0	4	0
0	4.33	0	0	0	0	1.75	28.25	32.75
0.75	5.33	10.33	2.33	0.75	12	2	0.5	1.5
1.5	7	4.33	2.33	1	22.75	4	1.75	2
0	0	0	0	0	14	3.5	2.5	3.25
0.75	0	0.33	0	0	0	0.25	0	0.75
2.5	4	2	0.67	0	18.25	4.75	2	1.75
8	2.33	0	0	3.5	4.75	2.25	0	1.25
5.25	0	0	0	0	0	4.5	0	5
0	0	0	0	0	0	0	0.25	0
1.5	2	0.67	0.33	0.5	2.75	0.25	2.75	0
2.25	0.67	9	3	1.5	1.75	1.5	2.5	0.25
3.25	0	8.33	0	6.75	0	0.25	5	1.5
0	0.33	4.33	0	1	4	2.5	1.75	0.5
0	3.33	0	0	0.5	10.25	1.25	4.5	1
1.25	0.33	1.33	4.33	0.75	6	0.25	1.25	13.25
0.25	1.67	1	0	0	13.5	1.5	2	4
0	22.67	2	0.67	0	0.5	2	0.25	2.25
46.5	9.67	0.67	79	0	0	34	0.75	31.5
28.25	0	0	0	4.25	245.75	0	20.25	21
0	7	119.33	0	30.5	0	14.5	0	0
35.25	0	0	0	13.25	307.5	0	0	0

0	43.67	36.67	0	63.25	5.5	0	2	56.25
28.75	76	63.33	0	42	0	146	63.5	14
0	0.33	0	0	0.25	24	0	0	0
15.75	0	54	0	48.25	0	38.5	18.5	14
25.25	62	0	75.33	0	0	0	11.25	5
0	0	0	0	0.25	13.25	0	0	0
0	0	0	0	0	0	0	3.5	24
0.75	0	4.67	0	2.5	7.5	1.75	3.75	2.25
2.5	0	0.33	2	0	0	0.75	0	0
57.5	2	0	0	0.5	0	0	0	43.5
10.25	7.33	0	2	0.5	14	37.5	0.5	2
0	1	1	0	3.75	0.25	0	0	0.25
0.25	0	0.33	0	0	71	0	0	0.25
0.75	0	0	0	0	13.5	0	0	0
0.25	2.33	0	1	0.25	5.5	0.25	2	0.25
0	0	0	0	2.75	3.25	0	0	0
6.75	1	7.67	0	1	13.75	0.5	2.25	0
27.25	0.33	0	0	2.25	0	0	0	0.5
6.5	0.33	0	5.33	0	0	0	0.75	0
0	6.33	0	0	0	137.75	0	0	0
0	0	0	12	0	0.25	3	0	5.25
6.5	0	1	0	0.25	1	0.25	0.5	34.5
0.25	3.67	5	0	2.25	0	0	0	0
4.75	0	3.33	0	15.25	0	28.5	2.75	2.5
5	0.33	0	25.67	0	0	0	0.25	0
0.25	2	0	0	1.5	42.5	0	0	3.25
0	0.33	0	0	16	18.25	0	0	0
1.75	0	9.33	0	5.25	0	6.5	4.25	2
0	0	0	0.67	0.25	1.5	0	1.25	0
7	0.67	0	4.67	0	7.5	0	0	0.25
0	0	0	0	0	0	0	6.25	1.75
3	0.33	0	0	1	13	0	0	0
0.25	0	0.33	0	31.25	29.5	0	0	13.5
0	0	0	0	0	0	0	0	0
0.5	0.33	0	1	0	0	0	0	0.75
0.75	0.33	1.67	0	3.25	11.75	3.75	0.75	0.75
0	0	0.33	0.67	0.25	0	0	0	6.25
0	0.33	0	1	1	0.25	2.5	0.25	1.5
0.5	0	0	0.33	0	0	0	0	4.25
0	0	0	0	0	0	0	0	0
0	0	0	0	0.75	3.5	0.25	0	0
0.25	0	0	1.67	0	8.75	0.25	0.5	1
0.5	0	3	0	6	2	1.5	3.75	1.5
6.25	0	0	9.67	0.25	0.25	0	0	1
0	0	0	0.33	0	0	0	71.5	1.75
0.5	0	7.33	0	0	0.75	0	0	0
0.25	0	0	0	0	1.75	0	0	0
0.5	3.67	0	0	0.5	0.25	0	0	14.25
35.25	0	1.67	0	0.5	0	0	0	9.25
0.75	0	0	0	0	0.25	0	0	7
0	1	0	2.33	1.25	5.5	2	2.25	1.75
0	0.33	0.67	0	28.5	0	0	0	0
0.5	0	2.67	0	5	0	0.5	2	2
0	0	0.33	0	0.25	22.75	0	0.5	0

1.25	0	0.67	0	0	0	2.75	0	1.25
0.5	1.33	0	2	0	0	0	0.25	0
0	0.33	0	0	0	0.25	0	0	0
0.75	0.67	0	0.33	0	0	0	0.5	4.25
0	0	0	0	0	0	0	0	0
0.25	0	0	0	7.75	0	0	0	0
0.5	0	4	0	2.25	0	1.25	1	1.25
0.5	0	1.33	0	0.25	2.5	0.5	1.5	0
0	0.67	0	0.33	0	0	0	1.25	0
0	0	0	0	0	4.75	0	0	0
0	0	0	0	0	41	0.25	0	0
1.25	0	0.33	0	0	1.75	0	0	0
0.5	0	0	0	1.75	3.75	0	0	0
0	0	0	0	0	0	0	0	0
1.75	0	0	0	3.25	11.25	0	0	0
0	4.67	0	0	0.25	0	0	0	4.25
0.25	0	1.33	0	0.5	0	3.75	1	0.5
0	1.33	0	3.67	0	0.5	0.25	0	0
0	0	0	0	0	0	0	0	0
0	0.33	0	0	0	0	0	0	0
1.5	0	0	2.67	0	0	0	0.25	0
0	0	0	0	0	0	0	0	8.75
0	2.67	0	30.67	0	0	0	0	0.25
1.5	1	0	4	0	0	0	0.5	0
0	0	0	0	0	0	0.5	0	0
0.75	0.67	0	1.33	0.5	0.5	0	1.25	0.5
0	0	0	1.33	0	2.5	9.25	0	0
1	0	0	0	0.25	42.75	0.25	0	0
0	0	0	12	0	0	0	0.25	0.25
0	0	0	0	0	7.75	0.25	0	0.25
0	0	0	0	0.25	0.25	0	0.25	0
0.75	0.67	0	1	0	0	0	0.5	0
0	0.33	0	2	0	0.25	0	0	0
0	0	0	0	1.25	0	0	0	0
0	1	0	1.33	0	0.75	0	0.25	0
0	0	0	0	0.75	4	0	0	0.5
1	0	0	0	0.75	0	0	0	7.75
0	0	0	0	0	2.25	0.25	0	0
0	0	0	0	0	0	0	0	0
0.25	0	0	0	0	3.5	0	0	0
0	0.33	0	0	5.25	1.75	0	0	0
0	0	0	0	0	0	0	0	0

8376	8378	8379	8381	8382	8383	8384	8385	8386
1621	531.67	829.33	466.67	669	426	303.25	167	518.25
493.75	124	574	45.67	91.33	3	109.75	33	104.75
519.5	62	235.33	53.67	324.67	110.5	268.75	2.5	228
115	215.33	7	85	155.67	12	78.75	13.5	81.75
0	223.33	0	1	0	0	233.25	0	0
338.25	107.33	223.33	26.33	55.67	0	56.25	42.5	129
68	184.67	0	79.33	111.33	57	49.75	135.5	37.75
0	167.67	0	56.67	33.33	159.5	80	0	0
1.75	0	83	0	0	0	0.25	0	83.75
0	0	0	43	63.67	404.5	0	0	0
231.5	73.33	305.33	0.33	0	0	18.25	0	1
0	0	0	0	0	0	0.25	92.5	0.25
17.75	44.33	0	21.33	7.33	0	26.25	56	10.75
0	0	0	0	0	0	72	0	0
61.5	0	91.33	0	0	0	18.75	0	29.25
51.25	20.33	46.67	7.33	7	0.5	15.75	11	21.25
0	0	198.33	0	0	0	40.5	0	0
77.25	52.67	0	14.67	34	1	2.5	20.5	43.5
248.5	0	9.33	0	0	0	0	0	21.5
42.25	23.67	79.33	22.33	23.67	25	12.25	5.5	13.25
0	46	0	0	0	0	50.5	0	0
0.5	0.67	0	43.67	17.67	60	50.25	0	0
199.25	0	155.67	0	0	0	0	0	53
0.5	0	0	66	63.67	123	0	0	2
1	0	0	0	0	0	0	0	0
0	42.33	0	0	0	0	0	0	0
134	0	11.67	0	0	0	0	0	39.25
0.25	0.33	0	9.33	1	0.5	10.75	0	0
51	4.67	0	80	36	22	22.5	125	2.75
18.25	0	0	0	0	0	0	0	0
1	39	32	21	3	15	4.75	9	3.25
22.25	0	35.33	0	0	0	5.25	0	6.75
1.25	0	6.33	121	25.67	81	0	0	0.75
0.25	0	73.33	0	0	0	3	0	0
0	0	0	0	0	0	0	0	0
9	29.33	0	4.67	6.67	0	1.25	0	1.75
1.25	10	0	0	0.67	0	1.25	0	1.5
14	15.33	2	0	1	0	0.75	0	9.25
0	0	0	0	0	0.5	32.25	0	0
0	0	0	0	0	0.5	0	0	0.25
0	14	10.33	0	0	0	3.5	0	0
23.25	12.33	25.33	3.33	2	1.5	3.5	0.5	10.25
0	0	0	0	0	0	29.5	0	0
0	8.67	0	0	0	0	0	0	5.5
0	0.33	0	0	0	0	0	41	0
6.25	0	20.67	0	0	0	1.5	0	50.75
0	0	0	0	0	0	0.25	0	0
3.25	25.67	0	3	2.67	0	3.25	3	9.75
0	0	0	0	0	0	0.5	0	0
44.5	0	0	0	0	0	0	0	0

24	0	0	0	0	0	0	0	0
0	55.67	0	0	0	0	0	0	0
8.25	24.33	1	2	8.67	0	5	0.5	4
18.25	0	38.33	0	0	0	2.75	0	8.5
12.5	0	7	14.67	3.67	13	0.75	0	5.5
0	0	0	56.33	0	0	0	84	0
3	6.67	17.67	3.33	10	2	4.5	1	5
9	2.33	5.67	11	18.67	4.5	9.5	3	3
10.75	24	0	13	17.67	21	8.25	6	6.75
42.75	0	11.33	0.33	3.33	1.5	0	5.5	16.5
1	10.67	0	4.33	10.67	23	6.5	0.5	3.5
0	0	15.67	0	5	0	0	1.5	0
2.75	1	11.67	0	1.33	0	0.5	0	0.75
0	38	0	0	0	0	0	0	0
0	61.67	0	0	0	0	0	0	0
1.75	4.67	0.33	5.33	8	5	0.75	0	2.25
2.25	25.67	0	0	6.67	0	7.75	8.5	8.25
8.25	0	1.33	0	0	0	0	0	6.25
8.25	3	14	0	0	0	4	0	3.5
20.5	0.67	7.33	0.67	12.67	2.5	0.5	6	8.75
0.5	7.33	0	1	7.67	3	4	4	2.5
14.25	2	1	2.67	6.67	1	2.25	7	5.75
3.5	4.67	0	2.67	1	0	0.25	0	0.5
0	12	0	5.33	0	0	7.75	0	0
13.25	1.33	20.67	0	0	0	1.5	0	5
9	1.67	3.33	0	0	0	0.25	0	4.5
0.75	2	0.33	1.33	0	0	0	0	0.5
24.75	0	10.67	0.33	0	0	1.5	0	7.75
0	0.33	0	9	16.33	16.5	0.5	0	2
0.75	6	0	8.67	0.33	5	6.25	4	0
6.5	4.67	0.33	2.67	5	1.5	1.75	1.5	0.25
0.25	2	0	1	42.33	0.5	0.25	0	0.5
0	0.33	0	0	0	0	15.25	0	0
4.25	1	0	34	3	0	0.5	0	0.25
1.75	5.33	0	0.33	1.33	0	0.75	1.5	0.25
9.25	2	0	2	2.67	0	7	3	3.75
15	0	0	0	2	0	0.75	0	1.5
0.75	0	0	0	0	0	0	0	0.25
4.5	3.33	0	1.67	2.33	0	0.75	1.5	2.5
3.5	0	7.33	1	0	0	1.25	1	0
34	0	0	0	0	0	0.25	0	0
0	0	0	0	0	0	0.25	0	0
0.25	1.33	0	1.33	2.33	0.5	36.25	0	0
3	1.67	0.67	0.67	2.33	3.5	1.5	1	3.5
3.25	0	40	0	0	0	0	0	4.5
10	4	5	0	1.33	0	1.25	9	0.25
1.25	0	0	0.67	5	0	1	0	0
0.25	17.33	0.33	0.67	5.67	9	1.25	0.5	2.25
3.25	2	0	0.67	3.67	0.5	1.25	1	0.75
3.75	1.33	5.33	1.67	2.67	1	2.75	6.5	0.5
0	33	8.67	226.33	123.33	173	20.75	0	1
8.5	45	0	0	0	0	1.75	0	2.5
0	0	29	0	39	229.5	2	0	0
0	30.67	0	0	0	0	3.75	0	0

16.25	4.33	68.33	0.33	0	0	14.5	0	0
42.5	8	19.33	0	31.67	0	104	52.5	63.75
0	0	0	0	0	0	0	0	0
25.75	10.67	39	0	66.33	0	40.75	14	62.25
2	10.33	0	0	0	0	0	0	1.25
0	0	0	0.33	0	0	0	0	0
2	0	0	1	0	0	0	49.5	2
0.75	27.33	18.67	0	1.67	0	8.75	0.5	2.25
0	0	5.33	1.33	1	50	0	0	0
10	0	0	0	0.33	0	0.25	0	0
0	2.67	0	0.67	0	4	2	0	0.25
0.25	0	4.67	0	0	0	0	0	0
0	0	0	0	0	0	0	2.5	0
0	2.67	0.67	0	0	0	3.75	0	1
4.75	3.67	0	0.33	0.67	0	0.75	1	1
0	0.33	0	0	0	0	0	0	0
0.5	8	1.33	0	0	0	1	16	1.75
0	5.67	0	1	0	0	24.75	0	0
2	2.33	0	0	0	0	0	0	11
0	0	0	0	0	0	0	3	0
0	0	0	2	7	13.5	0	23	0.25
5.75	0	0.33	0	0.33	0	0.25	40.5	0
0	2	5	0	0	0	1	0	0.25
4	1	2	0	1.33	0	5.5	42	1.5
0	12.67	0	6.33	0	0	0	10.5	0
0.25	0	2	0.33	0	0	0	0	0
0	0	0	0	0	0	0	0	0
0.75	0	10.67	0	4.33	0	3.25	2.5	9.75
2	3.33	0	0	0	4.5	0	0	0.75
0	0.33	0	1	0.33	0	0.5	0	0
0.75	0	0	2.33	0.33	0	0	0	0.25
0	0	0	0	0	0	0	0	0
0	0	1	0	0.33	0	0.25	0	0.75
0	0	0	0	0.33	0	0	0	0
0.75	0	0	0.33	0	0	0	0	13.25
2.25	2	0.67	0	0.67	0	7.25	0	1.75
0	0	0	66.67	6.67	2	0	0	0.5
0.25	0	3.67	3.33	1.33	6	0	0	0
0.25	0.33	0	0.33	0	0.5	0	12	0.5
0	0	0	0	0	0	0.25	0	0
0	1.33	0	0	0	0	0	0	0
0	0	0	0.67	0	3	0	0.5	0
0	6.67	0.33	0	0.33	0	1.5	5.5	0
0	0	0	0.33	0	0	0	0	0
0	0.33	0	0	0	0	0	0	0
0.25	3	1	0	0.33	0	2.25	0	0.5
0	0	1	1	1.33	0	0.5	1.5	0
1	0	1.33	0	0	0	0	0	0
0.25	19.33	0	0	0	0	0.25	24.5	0
0	0	0	0	2	0	0	0	0
0.5	0	0.67	0.33	1	0.5	0	0	1.25
0.25	0	3	2	3	5.5	0	0	0
0.75	7.33	8.67	0	0.67	0	1.5	2	3.25
0.25	0	0.33	0	0	0	0.25	0	0.25

0.75	0	1.33	2.33	1.67	2.5	0	0	0.5
0.25	0.33	0.33	0	0	0	0.25	0	0.5
0	0.33	0	0	0	0	2	0	0
1	3.67	0.33	0	0	0	0	0	0
0.5	0	0	0	0	0	0	0	0
0	0	0	0	0	0	0	0	0
1.25	0.67	3	0	1.67	0	6.25	0.5	4.75
1.25	2	0	0	0.67	0	1.5	0.5	3.5
0	0	0	0	0	0	0	0	0
0	0	0	0	0	0	0	0	0
0	0	0	0	0	1.5	0	4.5	0
0	0	0	0	0	0	0	0	0
0	0.33	0	0.33	0	0	0.25	0	0
0	0	0	0.33	0.33	96	0	0	0
0.75	1.33	0	0	0	0	0.25	0	0.25
0	0	9.33	1.33	0.67	0	0.25	0	0
1	0	5.33	0	0.67	0	0.75	0.5	1.25
0.25	1.33	0	0.33	5	1	0	0	0
0	0	0.67	0	0	0	0	0	0
0.25	0	3	0	0	0	0.5	0	0
0	0.67	0	0	0	0	0.25	0	0.25
0	0	0	0	0	0	0	0	0
0.25	1	0	0	0	0	0	1	0
0.5	0.33	0	0	0	0	0	0	0
0.5	0	0.33	0	0.67	4	0.25	0	0
0	7.67	0	0	0	0	0	0.5	0
0	0	0	0	0	0	0	0	0
0	0.33	0.33	0	0	0	0.25	0.5	0
0	0	0	6	0	0.5	0	0	0
0.25	0	0	0	0	0	0	2	0
0	0	1	0	0	0	0	0	0
0	0	0	0	0	0	0	0	0
0	0.67	0	0.33	0.33	0	0	10	0
0	0	0.33	0.33	0.33	0	0	0	0
0	0	0	0	0	2.5	0.25	0	0
0	0	4.33	0	0	0	0.25	0	0
2	0	0	0	0	0	0.25	0	0
0.25	0.67	0	0	0.67	2.5	0	0	0
0.5	0	0	0	0	0	0	0	0
0	1	0	0	0	0	0	0	0
0	0	0	0	0	0	0	0	0
0	0	2.67	0	0	0	0	0	0

8387	8388	8389	8390	8391	8392	8393	8394	8395
635.25	199.33	571	641.5	946.67	1438.33	351.33	1220.67	1542.75
193	17	243.5	174.25	258	667.67	158	165.33	541.5
136.5	24.67	153.5	169.75	377	216.67	150.33	1	125.5
106	23.33	196	145.5	260.33	33.67	111.33	645	516.5
158.5	150	0	0	0	81	262	335.67	0
129.5	3.67	73.5	141	172.33	119	56	165	330
62	26.33	48.5	97	122.67	129.33	44.33	312.67	275
67	82.33	0	0	0	22.67	29.67	34.67	14.5
61.75	0	0	25.75	111.33	308.67	115	0	131
0.5	4	0.5	0	0.67	0	0	2.67	32.25
0.5	0	128	15.75	3.33	22.33	13.67	0	243
0	0.33	0	0.75	0	0	0.33	0	0
57.75	27.33	66	33.25	134	25.33	18.67	165.33	240.25
30.5	115	0	0	0	143	0	23.33	0
23.25	0	0	20.25	14.33	150.33	0	0	0
18	6	23	24.25	31.67	63.33	12.67	40	70
0	0	0	40	0	0	0	0	0
92.5	0	0	14.75	1	1	0	0.33	14.5
91.5	0	0	0.75	58	500	0	0	0
21.25	3.67	7	26.5	13.67	92.33	4	20	12.75
22.25	46.67	0	0	0	10.33	31.33	103	0
7	27.33	168	35.25	0	0.33	0	368.67	14.75
101.25	0	0	5.5	0.33	3.33	0	0	2.5
0	3.67	0	0.25	0	0	0	0	20.5
0.25	0	0	0.25	0.33	0.33	0	0	44.75
0	0	0	0	0	0	83.67	103.33	0
69.25	0	0	31.5	1.33	142.67	0	0	0
16.25	19.67	0	0	0	9	34	30.67	0.5
2.5	36.33	19	16.5	9.67	232.33	0.33	231	29.75
0	0	0	0	0	0	0	0	14
2.5	2.67	5	3.5	3.33	12.67	3	9	32
0.25	0	0	6.5	10	225	0	0	0
0.5	0	0	0.25	0.33	4	0	0	14
0	0	0	39.5	0	0.33	0	0	0
0	0	0.5	0	0	0	0	0	0
6.75	1	36.5	16	33.33	0	16.33	12	0
0	0.33	14.5	0	6.67	0.33	16	0.33	59.5
3.5	1.33	8	3.5	3	65.67	3	5.67	3.5
28.75	17	0	0	0	0.67	0	20.33	0
0	0	0	0	0	0	0	0	0
0	0	14.5	0	14.67	0	16.33	0	73.5
11.75	0.67	6.5	6.5	4	39.67	0	10.33	39
35	47	0	0	0	12.67	12.67	87.33	0
0.25	0	9.5	0	8.67	0	14.33	0	59.75
0	0	0.5	0	0	0	0	0	0
0	0	0	1	66.67	2.33	0	0	0
49	40.67	0	0	0	33.33	0	14.67	0
3.25	1.33	9.5	6.25	37	4	20.67	6	9.75
0	0	0	0	0	0	0.33	30.33	0
0	0	0	0	0	0	0	0	21

0	0	0	0	0	0	0	0	43.75
0	0	0	0	0	0	1	0	0
17	2.67	9.5	2	20.33	1.67	0.33	0.67	3.25
8	0	0	3.25	6.33	4.33	0	0	4.75
6.5	3.33	1	3	20.33	10.33	5.33	7.67	5.75
0	0	0	0	0	0	0	0	0
5	1.67	3.5	6	9.33	13	4.33	0.67	2.75
8.5	0.33	10	5.25	14.67	9.67	4	33	6.25
9.75	4.33	14.5	16.75	12.67	0	0	46.33	2.75
6.75	1	0	3	5.67	32.33	0	4	15
4.75	2.67	17	4.75	9	4	0	9.33	18.5
0	1.33	1	3	0	0	1	0.33	17.75
10	0	9.5	3.25	3.33	26.67	4.67	11.67	8.75
0	0	0	0	0	0	53.33	30.67	0
0	0.33	0	0	0	0	35.67	18.33	0
2.5	1.33	5.5	4.25	6.67	0.67	3.67	7	19.75
12.75	0.33	23.5	9.25	6	1.33	4	0	5.25
2.5	0	0	6	18.67	0	0	0	0.75
4.75	0	2.5	2.75	8	30.67	3.33	0	5.25
8.5	0.33	0	3.75	0.33	21.33	0	0	3.25
5.25	4.33	1	0	4	3.33	4.33	5.33	22
4.25	4	4.5	6.75	11	4	2.33	4.33	6.75
1	0	3	5.5	5.33	7.67	2	4.67	22
6.25	14.33	0	0	0	2.33	4	10.67	0
11	0	1.5	2.5	6	21.67	2.67	0	13
4	0	1.5	0.5	2.67	28	3.67	0	23.25
1.75	0.67	5.5	0.25	6	0	4.67	3	19.75
0	0	1.5	1	12	19.33	3	0	15.75
0.5	1.33	0.5	0.5	0.33	2.67	0	0	4.5
15.75	16.67	0	0	0	1	2.33	1	44.75
1	1	1.5	1.5	2.33	1.33	2.67	1.67	10.25
0.5	2	22.5	0.5	46.33	0	0	13	0.25
1.5	14.67	0	0	0	4	6.67	16.33	0
0.75	2.33	16	3.5	2.33	2	0	6	0
2.5	1	3	2	8.33	1.33	1.33	35.67	23.25
1	1	2.5	2.75	18.33	7.67	2	0.67	3
4.25	0.67	3.5	13.5	11.67	0	0	1	0
0	0	0	0	0	0.33	0	0	4.25
1.75	0.67	5	3	15.67	21.67	3.67	2.67	3.5
0	0	3	19	1.33	0	0	0	5
0	0	0	0	0	0	0	0	2.75
0	0.33	0	0	0	0	0	0	0
9	22.67	0.5	1.25	0	1	3	5.33	6.5
2.5	0.33	1.5	0.75	12.33	16.67	0.67	6.33	4.25
2.25	0	0	7.25	5	10.67	0	0	2.75
2.75	1	0.5	1.25	2	6.33	0	11.33	15.25
2	1	0.5	3.25	2.67	0	0	0	9.5
2	0	6	3	2.67	0	5	6.33	7.25
2.5	1.67	1	3.5	7.33	0.67	0.33	2.67	4.75
2	0.67	1.5	3.5	8	11	0	1.67	0.75
25	69	0	73.25	69	4.67	0	1.33	80
0	0	0	0	0	59	7	0	0
58.25	0	0	24.25	15	8	0	2	14
0	0	0	0	0	0	6	0	0

44.25	0	0	5	4.33	1	0	2	5
60.25	36.67	126.5	0	45	11.33	56.33	26.33	0
0	0	0	0	0	0	0	0	0
24	26	21.5	0	27.33	6.67	37	28.67	0
0	0	0	0	8	5.67	0	0	12.75
0	0.33	0	0	0	0	0	0	13.75
0	0	0	0	0	1.33	0	0	0
2.25	0.67	24	0	2.67	2.67	6.67	5.33	0
0.5	0	0	0	0	0	0.33	0	6
0	0	0.5	0.25	0	0	0.33	10	7.5
0	3.33	0	0.5	0.33	0	6.67	0	2.25
6.75	0	0	0.25	18.67	0	0	78.33	0
14.75	0	0	0.5	1	0.67	0	27	0
0	0	0	0	0.33	0	0	0	0
0	0	0	0.25	0	0	0.67	0	0
0	0	0	0	0	0	0.67	0	0
1.5	1.67	4	0	2	0	0.33	1.33	0
0	1	0	9	0	0	0.67	0	0
0	0	0	0	0.33	9.67	0	0	9.75
1.25	0	0	0	0.33	0.33	0	0	0.5
0.25	1	0	0	0	0	0	0	17.25
0.25	0	0	0.25	0	0	0	0	11.75
16.75	0	0	1.75	2	0.67	0.67	1	0
5.5	0	3	1	3.67	38.33	1.33	1.33	0.25
0	7.67	0	0	0.33	0	0	0	4
0	0	0	0.5	0	0	0	37.67	0.25
0	0	0	1.75	0	0.33	0	0.33	0
4.25	1	9	0	3	0	4.67	0.33	0
0	0	0	0	0	1.67	0	0	0.5
0	0	0	0	0	0	0	0	5.25
0	0.67	0	0.25	0	18	0	6.33	0
0	0	0	0	0	0	0	0	0
0	0.67	0	0.25	0	2.33	0.67	0.33	2
0	0	0	0	0	0	0.33	0	0
0	0	0	0	0	9.67	0	0.33	0
0.75	3.67	8	0	1.67	0	4.33	1.33	0
0.5	9	0	17	0.33	0	0	0.67	0.25
0	0	0	0.5	0	0	0	1.67	3
0	0	0	0	0	1.67	0	0	0.25
6.25	0	0	0	0	0	0	21.33	0
0	0	0	0	0	0	0	0	0
0	0	0	0	0	0	0	0	0
19.5	1	0.5	0	0.67	0.67	0.67	0.33	0
0	0	0	0.5	0	0	0	0	1.5
0	0	0	0	0	7.33	0	0	0.75
37	0	0	0	0.33	0	0.33	0	0
0	0.67	0	0	0	0	0	0	0.5
0	0	0	0	0	0	0	0	0.75
1.5	0	0	0	0	0	1.33	46.67	0
0	0.33	0.5	0	0	0	0	0	0
0	0	0	1	0.67	0.33	1	0	3.25
1.75	0	0.5	1.5	1	0	0	1	3.75
1.75	0.67	12	0	3.67	0	9.33	0.33	0
0.25	0	0	0	0.33	0	0	1.67	0.5

4.75	4.33	0	0.25	0	0.33	0	0	2
0	0	0	0	1.67	4.67	0	0.33	0.5
0.25	0	0	0	0	0	0	0.67	0
0	0.33	0	0	0	15.33	0	1.33	0.5
0	0	0	0.25	0.33	0	0	13	0
0	0	0	0	0	0	0	0.67	0.75
1.75	0.33	2.5	0	0.33	0	4	0	0.25
1	0	0	0.25	0.67	0	0.67	0.33	0
0	0	0	0	0	0	0	0	0
0	0	0	0	0	0	0	0	0
0	0	0	0	0	0	0	0	0.25
0	0	0.5	0	0	0	0.33	0	0
0	0	0	0	0	0	0	0	0
0	0	0	0	0	0	0	0	2.5
0	0	0	0	0	0	0.33	0	0
2	0	0	0.25	0	0	0	0	0
3	0.33	1	0	0	0.33	0	0.67	0
0	0.33	0	0.25	0	0	0	0	0.5
10	0	0	0	0.67	0	0	7	0
2.5	0	0	0	1	0	0	0	0
0	0	0	0	0.67	0	0	0	0.75
0	0	0.5	0	0	0	0	0	0.75
0	0	0	0	0	0	0	0	0.75
0	0	0	0	0	2.33	0	0.33	1.75
0.25	1.33	0	0	0	0	0	0	3.5
0	0	0	0	0	0	0	0	0
0	0.33	0	0	0	0.67	0	0	0.25
0	0	0.5	0	0	0	0.33	0	0.75
0	0	0	0.25	0	0	0	0	0.5
0	0	0	0.25	0	0	0	0	1.5
0	0	0	1.5	0	0	0	0	0
0.25	0	0	0	0	9.67	0	0.33	3.25
0	0	0	1	0	0.33	0	0	0.25
0	0	0	0	0	0	0	0.67	0
0	0	0	0	0	0	0	0	0.5
0	0	0	0.75	0	0	0	0	0
0	0	0	0	0	0	0.33	0	1
0	0.33	0	0	0	0.33	0	0	0
0.25	0	0	0	0	0	0	0	0
0	0	0	0	0	0	0	0	0
0	0	0	0	0	0	0	0	0
1.75	0	0	0.5	0.67	0.67	0	0.33	0

8396	8397	8398	8399	8400	8401	8402	8403	8404
688.33	947.5	503.25	300	774.67	622.75	149.33	601	634.75
239.67	246	169	86.75	37.67	175.25	30	91	45.25
112.67	1141	127.75	58.25	197	145	47.33	48	98.25
140	122	118.75	119.25	183.33	172.75	80.33	101.5	49.25
0	0	0	76	0	0	311	0	152.5
114.67	320.5	89	39.25	28.67	119.5	23.67	51	68
48.67	163	44.25	55.25	78.33	105.75	48.33	105.5	74.25
0	0	0	15	47.67	0	97.33	0	57
23	63.5	57.75	31.5	0.67	70	22	81.5	0
0.33	0.5	0	0	210.67	0.25	0	0	0
2.33	107.5	21.25	38.75	12.67	0.25	0	0	11
0	0	0.25	0	0	0	0	1.5	0
49	92	6.75	20.25	1.33	50.75	17.67	99.5	19.5
0	0	0	0	0	0	58.33	0	63.5
14.67	0	35	0	0	40.5	1	0	0
23.67	36.5	21	14.25	2.33	26.75	10.33	14.5	15.25
66.67	0	14.25	0	0	112	2.67	0	0
30	2	0	0	0.33	25.75	0	0	23
0	0	7.75	0	0	85.5	0	0	0
22.33	1.5	10.75	2	10.67	21.25	3.67	11.5	4.25
0	0	0	12.75	0	0	54.33	0	50.5
2.67	0	0	0	13.67	0.5	0	0	6.25
0	0	0.25	0.25	0	18.25	0	0.5	9.25
0	0	0	0	73	0	0	0	0
0	0	0	0	0	0	0	0	37.75
0	0	0	50.75	0	0	0	0	0
0.33	0	12.25	0	0	33	0	0	0.5
0	0	0	8.5	0.33	0.25	0.67	0	1.25
18	0	0	0	14.33	10	0	0	3
0	0	0	0	0	0	0	0	69
16.67	19	2.5	4.75	6.67	101.25	27.67	49.5	7.25
2.67	0	23.5	0	0	13.75	0	0	0.25
0.33	0	0.25	0	43.33	0	0	0	0
32.67	0	12.5	0	0	46.75	0.67	0	0
0	0	0	0	0	0	0	0	0
19	25.5	9.75	10	0	15	9	34.5	11
0	22	34.75	64.5	1.67	13.25	6.33	18.5	15.5
3.33	79.5	12.5	6.5	1.67	0.5	0.33	2	0.75
0	0	0	0	0	0.25	75.33	0	14.75
0	0	0	95	0	0	0	388	0
3.33	4.5	4	14.25	5.33	10.5	8.33	22.5	8.25
13.33	23	1.25	0	8	3.5	0	0	1
0	0	0	31.75	0	0	0.33	0	52
0	82	15	18	6.33	9.5	7.67	3	7.75
0	0	0.25	0	0	0	0	0	0
13.33	0	6.75	0	0	6.5	0	0	0
0	0	0	0	0	0	95.67	0	33.75
5.67	21.5	4.5	1.5	0	13.5	3	8.5	6.25
0	0	0	0	0	0	0	0	0.5
0	0	0	0	0	0	0	0	4.75

0	0	0	0	0	0	0	0	17.75
0	0	0	34.75	0	0	13.33	0	0.75
6.67	0	1.5	1	19.33	5	0.67	0	18
8	0	5	0	0	2.5	0.33	0	7
1	0	6.5	2.25	14	2.5	8.33	1.5	2.25
0	0	0.25	0	0	0	0	0	0
2.67	5.5	1.75	4.75	4	5.5	1	0	9.5
13.33	0	3.25	3.5	3	4.5	9	9	6.75
19	0	0	0	19	1.25	0	0	3.25
0.67	0.5	3.75	0	19	3.25	0	0	3
8.33	33.5	5.5	1.75	16.67	5	16	0.5	7
0	13.5	0	0.5	6	1.25	0	0	0
1	14.5	7	5.25	10	4	2.33	19.5	4.75
0	0	0	2.75	0	0	0	0	0
0	0	0	24.25	0	0	0	0	0
5	13	3.75	3.5	7.33	1.25	1.33	7	2
4	41	17	4.25	14.33	1.25	15.33	0.5	2
0	0	0	0.75	0	0.5	0	61	0.25
1.33	0	8.75	1.5	0	10.25	0.33	0	4.75
0	0	1.5	0	17	0.75	0	0	1
3.67	11.5	1.25	1.5	4.33	6.25	8	4	5.25
2	31.5	0.25	1.25	4.67	3.25	6	0.5	2.75
4	9.5	1.5	5.5	0.33	3.75	4.33	1.5	5.25
0	0	0	8.5	0	0	2.67	0	6.75
2	14.5	12	2.75	1.67	1	1.33	5.5	2
0	5.5	2	3.5	0.33	2.5	2	6	1.5
0	3	5.75	7	1	1.5	4	6	9
10.33	0	7	3.75	0	2.5	0.67	8.5	0.5
0	0	0.25	0.25	10.67	0.25	0	0.5	0
0	0	0	9.5	1	0	7.67	0	7.25
4.67	11.5	3.75	1.5	5.67	3.25	0.67	5	1.75
0.67	0	0.25	0	3	0	0	0	0
0	0	0	7	0	0	18.33	0	14.25
29	0	0	0	2	0	0	0	0.75
2	16.5	0.75	1.75	1	2	0.33	11.5	2.75
0.67	13.5	4.25	3.25	1.67	4.25	3.33	0	2.25
14.67	35.5	0	0	3	3.75	0	0	0.25
0.33	0	0	74.25	0	0	0	34	0.25
1	20	1.5	4.5	1.33	6.5	1	2	3.25
4	0	1.75	0.25	0.67	1.75	0.67	4	1.25
0	0	0	0	0	0	0	0	3.25
0	0	0	0	0	0	0.33	0	0.5
0.67	0	1	8.5	0.67	0	5	0	3.25
2	1.5	3.75	0.5	6.67	2.5	1	6	0.75
3.33	0	1.25	1.75	0	1.25	1.33	3	1
2	11	3.75	0.75	1.67	5	5.67	1.5	2
4.33	34.5	0	0.25	8.33	0.25	0	0	0.25
0.67	10	2	1	9.33	2	2.67	0	1.25
2.67	20.5	0	3	1.67	0.25	0.67	4	0.75
0.67	4.5	4.25	0	0.33	3.75	2.67	0	0.25
0	0	21.25	41.5	93.67	9.25	29.67	0	64.5
41	90.5	0	14	0	14.5	0	0	0
0	0	9.75	6.75	36	18.75	22	0	15.5
19	67	0	14.25	0	0	0	4.5	0

0	0.25	2.75	18.5	0	17.25	2.67	1	23
113.33	0	0	0	0	1.25	0	54	0.25
0	49	0	0	0	0	0.33	42	0
83.67	0	0	0	0	0.25	0	27	0
0	0	25.25	25.25	0	64	8.67	0	20
0	58.25	0	0	57	0	0	7.5	0
0	0	0	0	0.33	15	0	0	0
3	15	0	0	0	0.75	0	5.5	0
2.67	0.25	3.5	9	5.67	0	0.33	0	0
0	120	0	0	1.33	3.25	0	1	19.25
1.33	1.75	2.5	65.75	0.67	3.5	1	0	0.75
0	0	1	1.75	0	5.75	4.33	0	0.75
0	45.25	4	0	0	0.25	0.33	0	1
21.33	109	0	3	0	0	0	0	0
12.33	0	0.75	1.5	0	2	5	0	0.5
3.67	0.25	0	0.25	0	0	0	58	0
10.33	5.75	0	0.25	0	0	0	0	0
14.33	0	0	1	0.67	0	0	0	7.5
0	0	3	2.5	0	3.25	9.67	0	6.5
0	22	5.75	0	0	0	0	0	0.75
0	0	0.75	0	8.33	0	1.33	0	0.25
0.33	0.5	0	0	0	0.25	0	0	8.75
0	0	2.25	12.5	0	0.25	6.33	0	0
4.33	0	0.5	0	0	0.25	0	0.5	0.75
0	0	24.25	0.75	0	17.25	20	0	2.5
0	17.5	0	0.75	0.67	0.75	0.67	0	2.25
0	42.5	0	8.5	0	0	9	14.5	0
0.33	0	0	0.25	0	0	0	16	0.25
0.33	3.25	0	0.25	1.67	0.5	3	0	0.25
0.33	17.25	2	1	2	0.25	1.33	0	0
0	0	0	0.25	0.33	4.25	0.67	0	1
26.33	39	0	0.5	0	0	0	7	0
0.33	5.25	0	6.5	0.33	0	43	0.5	6.75
0	0	0	0	0	0	0	0	0
0	0	0	0	0.33	6.5	0.67	0	0
0.67	35.25	0	0.75	0	0	0	3	0
0	0	0	0	9.33	0.25	2.67	0	1
0	0	3.75	1	0.33	0	0.67	0	3
0	0	0.5	0	0	0.75	0.33	0	0.5
0	0	0.25	0	0	0	0	0	1.75
0	23.75	0	0	0	0	0	1	0
0	14.5	0	0.25	2	1.25	0.33	0	0.25
3.67	0	0.25	0	0	0	0	0	0
0.33	0.25	6.75	0.5	0	0.25	0.33	0	0.5
0	0.25	0	0.75	0	1.5	0	0	0
1.67	0	0	0	0	0	0	0	0
0	25.75	0	0	0.67	0	1	0	3.25
0	28.75	1.25	0	0	0	0	0.5	4
0.33	0	0	0	0	1	0	0	0
0	0.25	0	0	1.33	0	0.33	3	0.25
0	31	5.75	1	0	0.5	3.33	0	0.75
0	0	0.5	0	0.67	0.5	1	1	0
3.67	0	0	0	0	1.25	0	5.5	0
0.33	16.5	0	0	0	0	0	0	0.25

0.33	0	3.25	0	0.33	0	0	0	1
0	0	4.75	5.25	0	0.25	0	0	1
0	0	0	0	0	0	0	0	0
0	0	1.75	2	0	3.75	2.67	0	2.5
0	0	0	0	0	0.25	0	0	0
0	0	0	0	2	0	3.33	0	0
1.67	0	0	0	0	0.5	0	1	0
1.33	0.75	0	0.5	0	0	0	0.5	0
0	0	0	0.25	0	0.25	0	0	0.75
0	13.75	0	0	0	0	0	0.5	0
0	35.5	0	0	0	0	0	0	0
0	1.25	0	0	0	0	0	0.5	0
1.33	2.5	0	0	0	0	0	0	0
0	0	0	0	8	0	0	0	0
1.67	3.25	0	0	0	0	0	0.5	0
0	0	0.25	0.25	0	1.75	0.33	0	1
1.33	0	0.25	0	0	0.25	0	1.5	3
0	0.25	2.25	2	2	1.5	0.67	0	0.25
0	0	0.25	0	0	0	0	0	0
0	0.25	0.25	0	0	0	0	0	0.25
0	0	0.5	2.25	0	7	7	0	2.5
0	0.25	0.25	0	0.33	0	0	0	4.25
0	0	0.5	17.75	0	0.5	0	0	0
0	0	0.5	11	0	0.25	2	0	0.75
0.33	0	0	0	1	0.25	0	0	0
2	1	0.25	1	0	0.5	0	0	0
0	2.5	0	0	0	0	0	0	0.5
0	1.5	0	0.5	0	0	0	0	0
0	0	0	0.25	0.33	0	0	0	0
0	11.5	0	0	0.67	0	0	0	2.25
0	4.5	0	0.25	0	10.5	0	0	0
0	0	4.25	1.5	0	2	0	0	0.5
0	0	1.75	1.5	0.67	0.25	0.33	0	1
0	21	0	0	0	0	0	0	0.25
5.67	3.75	1	0.25	0	0	0	0	1.25
0	3.75	0	0.25	0	0	1.33	0	0.25
0	0.25	0.25	0	2.33	0.25	0.33	0	4.75
1.33	0.5	0	0.5	0	0	1.33	0	0
0	0.75	0	0	0	0	0	0	0
2.33	1	0	0	0	0	0	0	0
0	18.25	0	5.25	0	4.25	0.33	0	0
0	0.5	14	0	0	0	0	0	2.25

8405	8406	8407	8408	8409	8410	8412	8420	8421
674	751	253	155.5	422.5	761	581	661.75	0
115	227.33	0.5	43	0	158	238	124.5	0
272	177.67	90.5	94	102.5	1	90.33	138.5	0.5
230.5	230.67	107	14	15	0	132	16.25	0
0	0	0	303	0	0	0	0	489
86.5	104	0	40.5	0	47	60.33	117.75	0
107	53.67	39.5	0	27.5	0	17.67	42.5	0
0	0	11.5	102	256	0	0	50.25	43.5
39	1.67	0	27.5	0	0	22	68	0
0	0	50	0	269	0	0	0.25	0
2.5	226.67	0	18	0	0	0	1.75	0
0	0	0	0	0	0	0	10	0
78.5	38	0	0	0	0	14.33	0	0
0	0	0	0	0	0	0	0	59.5
0	57	0	52	0	0	15.67	31.5	0
20.5	23.33	0	6.5	0	10	14.33	29.75	0
0	144	0	35	0	0	85.33	30	0
31.5	0	14.5	0.5	63.5	20	17.67	72.25	0
0	0	0	0	0	2	10.33	0.25	0
0	32.33	22	9	13.5	0	7.33	26.5	0
0	0	0	43	0	0	0	0	89.5
0	0	17	0	2.5	4	6.33	0	0
0	0	0	0	0	1	1	34.25	0
0	0	8	0	69	0	0	0	0
0	0	0	0	0	196	57.67	0	0
0	0	0	54.5	0	0	0	0	141.5
0	1	0	0.5	0	0	0	5.25	0
0	0	0	1.5	1	0	0	0	1
11.5	0	21	4.5	6	0	3.67	5	1
0	0	0	0	0	38	40.67	0	0
4	15.67	4.5	11.5	4.5	1	2	2.5	0
0	16.33	0	18	0	2	2.33	9.75	0
1.5	0	94	1.5	78.5	0	0.67	0.75	0
0	57.67	0	21	0	0	11.67	19.5	0
0	0	0	0	0	0	0	17	0
38.5	15.67	0	0.5	0	0	6	5.25	0
16	4.33	0	0	0	1	0	0	0
18	0	0	0.5	0	17	12	0.5	0
0	0	0	0	0.5	0	0	5.75	40.5
0	0	0	0	0	0	0.33	0	0
15	24	0	3.5	0	0	2	3	0
11.5	10	0	0	0	0	9.67	15.75	0
0	0	0	8.5	0	0	0	0	18
14	11	0	0	0	0	0	0	0
0.5	0	0	0	0	0	0	0.5	0
0	2	0	2.5	0	0	0.67	7.25	0
0	0	0	0	0	0	0	0	1.5
9	6	0	0	0	0	4.67	1.75	0.5
0	0	0	44.5	0	0	0	0	136.5
0	0	0	0	0	60	5	0	0

0	0	0	0	0	110	6.67	0	0
0	0	0	0	0	0	0	0	5
8.5	0	0	0.5	0	23	17.67	1.75	0.5
0	8.67	0	12	0	12	6.67	3.75	0
2.5	0.33	11	3	7	0	1.67	7.25	14.5
0	0	0	0	0	0	0	0	0
2	4.67	0	5.5	2.5	2	6.33	2.25	0
9	5.67	8.5	1	1	1	5.33	10.5	0
7.5	0	7	0	9.5	0	5.67	2.25	0
2.5	0	0.5	0.5	0.5	0	0	1.25	0
7.5	3.67	3.5	1	5.5	0	1	1.75	1
0	2.67	0	4	0	0	0.33	0.25	0
10	18.33	0	1.5	0	0	2.67	2.25	3
0	0	0	72.5	0	0	0	0	17.5
0	0	0	1.5	0	0	0	0	3
6.5	4.33	2	0	4.5	0	1.33	1.25	0
7.5	0.33	0	0	0	0	1	3.5	0
0	0	0	0.5	0	1	0.33	12.5	0
0.5	11	0	2	0	6	3.33	5.5	0
0	12.33	0.5	1	2	6	2	1.25	0
6	1	0	4.5	7	0	0.67	0	1.5
8.5	2.67	2.5	2.5	0	0	1.33	0.25	1.5
7	4	0.5	0	0	0	0	3.25	0
0	0	0.5	9	0	0	0	0	20
5	5.67	0	8.5	0	0	1	5.25	0
0	14.67	0	3	0	0	0	4	0
7.5	0	0	1.5	0	2	1	0.25	3
0.5	8.33	0	1.5	0	2	0.67	4.5	0
1	0.33	9.5	0.5	12.5	0	0.33	0	0
0	0	0.5	5	0	1	0	1.75	6
3.5	1.67	1.5	0	2	0	2	2.5	0
0.5	0	0	0	0	0	13.33	0	0
0	0	0	9.5	0	0	0	0	30.5
1	0	0	0	0	0	0.33	0	0
7.5	3.67	0	0	0	0	1	1.5	0
2.5	0.67	0	0	0	2	1.67	0.5	0
12	0	0	0	0	0	1	0.5	0
0	0.33	0	0	0	1	0	0	0
1.5	1.67	0.5	0.5	0	1	1.67	0.25	0.5
2	17.67	5.5	9	0	9	3.67	1.5	0
0	0	0	0	0	7	4.33	0.25	0
0	0	0	0.5	0	0	0	0	3
2	0.33	0	1	2	0	0	0.25	3.5
0	3	0.5	1.5	2.5	5	1.33	3.25	3
0	1.67	0	0	0	9	2	2.5	0
1.5	3.67	0	2.5	0	5	1	2	6
5	0	0	0	0	0	0	0	0
0	1	0	0	1	3	4.67	1.5	0
2	0	0.5	0	0.5	0	1	1	0
1.5	0.67	0.5	0	0.5	0	4.33	1	1
10.5	34.33	52.5	0	168	68	66	0	0
0	0	0	50.5	0	0	0	19.25	0
0	19.33	0	66	76	134	40	17.25	0
0	0	0	28	0	0	0	0	0

0	132.67	29	12	0	0	20	3.5	65
41.5	0	0	0	0.5	0	0	19.75	0
0	1.67	0	0	0	0	0	0	0
12.5	0	0	0	0	0	0	6.75	0
7	12	0	0	0	0	0	16.25	0
0	0.33	0	0	0	0	0	0	0
0	0	0	1	0	0	0	41.25	0
0	0	0	0	0	0	0	19	0
0	0	9	1.5	16.5	39	20.33	0.25	0
0	0	23	0	0	0	0.33	0.25	31
1	0	0.5	0.5	3.5	9	1	9	0
0	2.33	0	7	0	0	52	104.25	0
0	0	0	0	0	0	0.33	0	0
0.5	0	0	25.5	0	0	0.33	0	0
0	0.33	0	0	0.5	1	0.33	23	0
0	0	0	92.5	0	0	0	0	0
0	0.33	0	0.5	0	0	0	0	0
0	0	0	19.5	0	0	0.67	0	0
2.5	1.33	0	0	0	0	0	1	0
0	0	0	0	0	0	0	0	0
0	0	14	0	35.5	12	13.67	0	0
0	0	0	0	0.5	0	0	0	0
0	2.67	0	10.5	0	0	11.67	5	0
0	0	0	0	0	0	0	0.25	0
10	1.33	0	0.5	0	0	0.33	0.25	0
0	100.33	1	157.5	0	0	22.67	0	7
0	0	0	0	0	0	0	1	0
8.5	0	0	0	0	0	0	18.5	0
0	0	0	0	8.5	2	0.67	1.5	0
0	0	0	3	0.5	1	1	0.25	0
0	0	4	0.5	0	0	0	1	28
0	0	0	7.5	0	0	0	0	0
0	1.67	1.5	1	0	0	0	2.25	62
0	0	0	0	0	0	0	2.5	0
0.5	0.67	2.5	0	0	0	0	2.5	10
0.5	0	0	4.5	0	0	0	0	0
0	0	50	0	0	1	0	0.5	0
0	1.67	0	2.5	5	14	12.33	6	0
0	0	0	0	0	0	0.67	0	0
0	0	0	0	0	0	0	0	0
0	0	0	0	0	0	0	0.5	0
0	0	0.5	0	7.5	2	1	0.5	0
0	0	0	0	0	0	0	0	0
0	0.33	0	0	0	0	0	0	0
0	0	0	0	0	0	0	0	0
3	0	0	2	0	0	0	0	0
0	1.67	3.5	0	6	3	6.33	2.5	0
0	0.67	0	2.5	0	0	15	0	9
0	0	0	0	0	0	0.33	0.25	0
0	0	1	0	0	0	1	0	2
2	2	2.5	1.5	0	0	0	2.75	0
0	1.33	1	4	7	4	4	0.75	0
0.5	0	0	0	0	0	0	9.75	0
0	0	0	0	1	0	2	0	0

0	0	1.5	0.5	3	1	2	0.25	0
0	0.67	0	0	0	0	0	0.25	1
0	0	0	1	0	0	0	0	0
0	6	1	0	0	0	0	0.25	6
0	0	21	0	0	0	0	0.25	90
0	0.33	0	0	0	0	0	0	2
0.5	0	0	0	0	0	0	0.25	0
0.5	0	0	4	0	0	0	0.5	0
0	0	0	0	0	0	0	0	0
0	0	0	0	0	0	0	0	0
0	0	0	0	1.5	0	1.33	0	0
0	0	0	2.5	0	0	0	0	0
0	0	0	0	0	0	0.33	0	0
0	0	1	0	3	6	0	0	0
0	0	0	0.5	0	0	0	0	0
0	20.67	0.5	0.5	6	0	2.33	4.75	0
0	0	0	0	0	0	0	0.75	0
0	0	0	0	1	5	9.33	0	0
0	0	0	0	0	0	0	0	0
0	0	0	0	0	0	0	0	0
1.5	5.33	0	0	0	0	0	0	0
0	0	0	0	0	0	0.67	0	0
15.5	0	0	0	0	0	0	0	0
0.5	0	0	0	0	0	0	0.25	2
0	0	0	0	6.5	9	0	0	0
0.5	0	0	0.5	0	0	0	0	0
0	0	0	0	0	1	0.33	0	0
0	0	0	1.5	0	0	0	0	0
0	0	11.5	0	0	0	0.33	0.25	0
0	0	0	0	0	0	0	0	0
0	10.33	0	0	0	0	0.33	1.25	1
2	8.33	0	0	0	0	0	1.5	0
0.5	0.67	0	0	0.5	0	1	0	0
0	0	0	0	0	0	0	0	6
0	0	0	0	0	2	0.67	0	0
0	1	1	0	0	0	0.67	0.75	0
0	0	0	0	0.5	0	0.33	0	0
0	0	0	0	4.5	1	0.67	0.25	0
0	0	0	0	0	0	0	0	0
0	0	0	0	0	0	0	0	0
0	1	0	0	0	0	0.33	0	0
0	0	0	0	0	0	0	0	0

8422	8423	8424	8426	8428	8429	8430	9057	9058
243	621.75	342.67	310.5	265.25	619	352	485.75	365.5
45.75	262.5	410.33	0	90.75	177.33	124	80.5	37.25
55.75	71.75	94	0	62	121.33	114	126.25	84.75
0.25	0	2.67	0	126.25	28	0	78.75	129.75
97.25	0	0	0	0	0	0	0	221
31.5	98.75	57.67	0	40.5	87.33	49	74.25	43.75
0	3.5	0	87	99.25	30	0	51.5	59.75
35.5	0	0	0	0	73.67	0	0	23.5
6.5	48.5	60.67	0	9.25	0	50	2.75	10.25
0	0	0	0	0	4.67	1	1	0.25
9.5	14.5	70.33	0	22.75	0	0	84.75	20.5
19.75	54.75	31.33	134	56.25	0	46	0	0.25
0	0	0	0	23	0.33	0	34.75	40.25
48.5	0	0	0	0	0	0	0	42.25
13	49.5	18.67	0	0	0	26	5	0
14.75	13.25	14.67	0	16	26	13	17.75	12.25
0	0	38.33	0	0	0	0	0	0
2.25	0	0.67	0	5	29.67	0	0.5	0
0	74.25	52	0	0	0	0	35.5	0
6.5	12	24.33	0	3.75	14.67	24	10.5	1.75
21.75	0	0	0	0.25	0	0	0	39.5
0	19.5	0	0	0	31	0	1.75	0
0.25	17.25	1	0	0	0.33	0	13.5	0
0	0.25	0	83	0	0.33	0	0.25	0
0	52	0	0	0	99.33	0	0	0
0.5	0	0	0	0	0	0	0	36.5
0	24.25	16	0	0	0	0	8	0
3	0	0	0	0	0	0	0	46.5
0	0	0	0	0	19.33	0	0.5	1
0	28.5	0	0	0	47.67	0	0	0
6.25	19	2.33	0.5	16.5	1.33	5	11.25	33.25
22.75	18.5	11.33	0	0	0.33	20	2.25	0
0.25	0.25	0.67	0	0	0	0	0	0
0	0	9	0	0	86.33	0	0	0
31.75	59.75	1.33	10	11.25	0	31	0	0
1.75	0	0	0	2	20	0	17	7.75
0	0	0	0	45.5	0	0	7	16
0	8.75	2	0	0.25	12.33	3	3.5	0
5.25	0	0	0	0	26	0	0	27
116	0	0	0	0	0	0	137.5	0
8.25	2.5	2	0	15.25	0	5	8	9.5
0	4.75	4	0	7	10	4	12.5	0
21.25	0	0	0	0	0	0	0	28.75
0	0	0	0	11	0	0	0	8.5
64.75	9.75	0	27	4.75	0	2	0.75	0
21.5	0.5	1.33	0	0	0	0	0.5	0
22.5	0	0	0	0	0.33	0	0	46.75
0	0	0	0	0.5	6	0	12.75	4.25
0	0	0	0	0	0	0	0	40.5
0	3.75	0	0	0	46.33	0	0	0

0	13	0	0	0	0	0	0	0
0	0	0	0	0	0	0	0	1.5
1.25	4.5	1.33	0	0.5	22	0	3.25	0.25
3.75	5.75	1.67	0.5	0	3.67	18	0.25	0
4	1.75	8.67	6	2.25	0	0	6.75	0.75
0	0	0	43	42.25	0	0	0	0
0.75	1.25	0.33	1.5	1	7.33	0	2.5	1.75
4	1.75	4.33	0	1	12.67	0	4.5	6.25
0	0	0	0	0	0	0	2.75	0
0	5.5	3.67	1.5	4.25	0	8	2	0.25
0.25	0	0	3	4.75	1.67	0	2.25	5.25
0	1	1.33	12.5	8	0.33	14	1.25	0
0.25	2	4	3.5	3.75	1.33	0	1.25	3.5
0	0	0	0	0	0	0	0	23.25
0	0	0	0	0	0	0	0	0.25
1.25	2.75	3.67	0	1.5	5	4	2.25	2.25
0	0	0	0	4.75	2	0	3.75	1.75
2.25	1.5	23	0	0	1.33	0	2	0
2.75	4.5	5.33	0	1	2.67	1	5.75	4
0.25	3.75	4.67	5.5	1.25	0.33	3	1	0
5.5	0	0	0	4.25	0	0	4.5	4.5
1	3.75	2.33	3.5	1.25	8	2	3.75	5.5
6	0.75	1.67	0	6.5	9.67	4	2.25	1
8	0	0	0	0	0	0	0	9.5
1.5	3.5	6	0	6.75	0	0	3.25	3
0.25	1.75	0.33	0	4.75	0	4	1	3.5
2	3.25	2.67	0	5	4.33	5	3.25	5.25
3.25	4	2	2	0.25	1	0	0	1.5
0	0.25	0	19.5	0.25	0.33	0	0	0.5
4.25	1.75	4.33	2	4.5	0	0	0	8.5
0	0	0	8.5	5.75	4.67	0	2	4.5
0	0	0	0	0	0.33	0	0.25	0
2.75	0	0	0	0	0	0	0	9.75
0	0	0	0	0	0	0	14.25	0
0.5	2	0	0	2	0.33	3	2.75	0.75
0.5	1.5	0	0.5	3	1.67	0	1.75	1.25
0	0	0	0	0	1	0	5.5	0
10	0.25	1	0	0	27.33	0	63	0
0	1.75	0	0	2.25	2.33	0	1.5	1.25
0	1.5	0	42	0.5	4.67	0	2.25	0.5
0	4	0	0	0	7	0	0	0
0.75	0	0	0	0	0	0	0	0.5
4	0.25	0.33	0	0	0.67	0	0	7.75
0.5	1.5	2	3.5	0	9	1	1	3.25
0	1.75	4.67	0	0	0.67	0	5	0
0.25	2.75	0	1	2.5	0.33	2	1.25	3.75
0	0.75	0	0	0	0	7	0.25	0.5
0	0	0.33	0	4.25	2.67	0	1.5	2
0.25	0	0	1	0.75	9.33	0	2	0.25
1.75	0.75	1.67	0.5	1.75	7	0	2	0.75
63.5	0	15	161	0	66	76	0	58.5
0	58.75	35	0	5	0	0	4	28.25
55.75	0	5	0	0	63.67	30	0	5
0.25	0	0	0	0	0	0	7	32.75

32	20.75	9	0	0	0	10	0	24.25
45.25	0.5	0	0	13.67	20	0	35.25	21.75
0	0	0	0	0	0	0	27	0
5	0	0	0	16.33	6.33	0	35	5.5
6	7.25	28.67	0	51	7.33	0	1.75	22.75
0	0	0	0	41.33	0	0	94.25	0
0	57.25	5.67	0	11	0	0	23.75	0.25
4.25	0	0	0	9.33	21.67	0	7.75	0.75
0	0	0.33	57	0	9.33	97	0	8.75
0	11.5	0	0	0	0	0	0	0
0.25	27.75	0	0	4	4	0	2.25	2.5
3.25	0	3	0	0	0	41	0	0
1	2	4.67	0	0	0.33	20	0.25	2.75
0	0	0	0	0	0	0	0	3.5
1	3	0.67	1	4.67	1	0	0	11.5
0	0	0	0	0	0	0	0.75	0
1.75	0	0	0	0	1.33	0	2.75	16
0	0	0	0	0	0	0	0	0
6.25	10	0.33	0	0.67	3	0	0	7.25
12	0	0.67	0	0	0	0	1	0
0	0	0	35	0.33	0.67	0	0	0
0.5	7.25	0.33	0	0.67	0	0	0.25	0
2.5	0	8	0	0	0	14	0	6.25
1.75	0	0	0	3.67	17.67	4	32.75	0.5
3	0.25	0	0	0	17.33	0	0	20.5
0	0	0.33	0	1.33	0	0	1.5	0.25
0	0	0	0	0	0	0	0	0
3	0	0	0	6	15.33	1	3.25	1
0.25	11.75	0	23	1.67	1.67	0	0.25	0
7.5	0	0	42	0	0	0	0	0.5
0	5.5	0.33	0	1.33	0	0	0	0.25
0	0	0	0	0	0	0	1.25	0.75
1.25	1	1	0	0.67	0	0	0	0
0	0	0	0	0	0	1	0.25	0
0.25	4	2	0	10.33	1	0	2.25	0.25
0	0	0	0	2	0	0	2.75	0
0	0.25	0	0	0.33	0.67	0	0	0
0.25	3	0.33	17	0	2	0	0	0
0.5	7	0	0	0	0.33	0	0.5	0
0	0	4.33	0	0	0	0	0	7.75
0	0	0	0	0	0	0	0.5	0.25
0	3.75	0	3	0	2	0	0	0.75
0.5	0	0	0	1.67	0	0	0	6.5
0.5	0	0	0	0	1	0	0	0
0	0	0	0	0	0	0	0	0.75
0	0	0	0	0	0	0	0	0
21	0	0	4	0.33	1.67	0	0	0
1.5	8	0	0	0	0	0	0	0
1	0	0	0	0	1	0	3.5	0
0	0	0	0	0	0	0	4	0
1	0	0	8	0	0	0	0.5	0
2.75	0	0.33	1	0	0.67	5	0	0
1	0	0	0	2	2.67	0	1.75	0
0	0	0.33	0	0.33	0	4	0	0

0.25	0	0.67	1	0.33	2.33	1	0.25	1.5
2.5	0.75	2	0	0	0.33	2	0	1
0	0	0	0	0	0	0	0	0
0.75	1.5	1.67	0	1	0	0	0	4.25
0	1.5	0.67	0	0	0	0	0.25	0
0	0	1.33	0	0	0	0	0.75	0.25
1	0	0	0	1	0.67	1	0.25	0
0.5	0	0	0	0.33	0	0	0.75	2.25
0.75	9.25	0	0	0	2	0	0	0
0	0	0	0	0	0	0	0.5	0
0	0	0	0	0	0	0	0	0
0	0	0	0	0.33	0	1	0.5	0
0	0	0	0	0	0	0	0	0.25
0	0	0	0	0	1.67	0	0	0
0	0	0	0	0	0	0	0	4.25
0	2.5	0	1	0	0	0	0	0
0.75	0	0.33	0	1.33	1.67	1	0	0.25
0.25	0	0	11	0	1.33	0	0	0.25
0.75	0	0.33	0	0	0	11	0	0.5
0.25	0.25	4.67	0	0	0	5	0	0.75
1.5	0.25	0	0	0	0.67	0	0	4.5
0	1.75	0	0	1	0	0	0	0
0.25	0	0	0	0.33	0	0	0	20
0.5	0.75	0	0	0	1	0	0.25	0
0.25	0	0	0	0	0.33	4	0	0.25
1	1.75	0	0	0.67	1	0	0	0.75
0	0	0	0	0	0	0	0	0
0	0	0	0	0.33	0	0	0	0.25
0	0	0	3	0	0	0	0	0.5
0	2.25	0	0	0	0	1	0	0
0.25	0	0	0	0	0	0	0	0
0.25	0.5	1.67	0	0	1.33	0	0	0.5
1.25	0	0.67	0	0	0.67	0	0.75	0.75
0	0	0	0	0	0	0	0.25	0
0	0	0	0	0	0.33	0	0	0
0.25	0	0	0	0	0	0	0.25	0
0.25	16.5	0	0	2	0	0	0	0
0	1.25	0.33	0	0	0.33	0	0	0
0	0	5.67	0	0	0	0	0	0
0	0	0	0	0	0	0	0	0.5
0.25	0	0	0	0	0	0	1	0
3.5	0	4	0	0	0	1	0	1.25

Phylum	Class	Order	Family	Genus
roteobacter	aproteobact	rkholderia	alobacterac	Massilia
roteobacter	aproteobact	aproteobact	rkholderia	alobacteraceae
ctinobacter	ctinobacteria			
acteroidete	lavobacteri	vobacteria	vobacteriac	avobacterium
roteobacter	aproteobact	rkholderia	alobacterac	Massilia
roteobacter	aproteobact	aproteobact	rkholderia	namonadaceae
ctinobacter	ctinobacter	romonospo	omonospor	ctinoplanes
acteroidete	lavobacteri	vobacteria	vobacteriac	avobacterium
roteobacter	naproteoba	udomonad	domonada	seudomonas
roteobacter	aproteobact	aproteobact	rkholderia	alobacteraceae
roteobacter	naproteoba	udomonad	domonada	seudomonas
acteroidete	lavobacteri	vobacteria	vobacteriac	avobacterium
acteroidete	lavobacteri	vobacteria	vobacteriac	avobacterium
roteobacter	aproteobact	aproteobact	rkholderia	alobacteraceae
ctinobacter	ctinobacter	romonospo	omonospor	ctinoplanes
roteobacter	aproteobact	aproteobact	rkholderia	namonadaceae
acteroidete	lavobacteri	vobacteria	vobacteriac	avobacterium
roteobacter	naproteoba	udomonad	domonada	seudomonas
acteroidete	lavobacteri	vobacteria	vobacteriac	avobacterium
ctinobacter	ctinobacter	ctinobacter	icrococcal	robacteriaceae
roteobacter	aproteobact	aproteobact	rkholderia	namonadaceae
roteobacter	naproteoba	udomonad	domonada	seudomonas
acteroidete	lavobacteri	vobacteria	vobacteriac	avobacterium
roteobacter	aproteobact	aproteobact	rkholderia	namonadaceae
ctinobacter	ctinobacter	ctinobacter	ineosporial	neosporiaceae
roteobacter	aproteobact	aproteobact	rkholderia	alobacteraceae
acteroidete	lavobacteri	vobacteria	vobacteriac	avobacterium
acteroidete	lavobacteri	vobacteria	vobacteriac	avobacterium
ctinobacter	ctinobacteria			
acteroidete	lavobacteri	vobacteria	vobacteriac	avobacterium
ctinobacter	ctinobacteria			
ctinobacter	ctinobacteria			
acteroidete	lavobacteri	vobacteria	vobacteriac	avobacterium
acteroidete	lavobacteri	vobacteria	vobacteriac	avobacterium
acteroidete	lavobacteri	vobacteria	vobacteriac	avobacterium
roteobacter	aproteobac	Rhizobiales	hizobiacea	Rhizobium
ctinobacter	ctinobacteria			
roteobacter	aproteobact	aproteobact	rkholderia	namonadaceae
acteroidete	lavobacteri	vobacteria	vobacteriac	avobacterium
roteobacter	aproteobact	rkholderia	alobacterac	Massilia
ctinobacter	ctinobacter	ionibacter	cardioidac	ocardioides
roteobacter	aproteobac	ingomonad	ngomonad	phingobium
roteobacter	naproteoba	udomonad	domonada	seudomonas
ctinobacter	ctinobacter	ctinobacter	icrococcal	robacteriaceae
acteroidete	lavobacteri	vobacteria	vobacteriac	avobacterium
ctinobacter	ctinobacteria			
ctinobacter	ctinobacteria			
roteobacter	aproteobac	aproteobac	Rhizobiales	hizobiaceae
ctinobacter	ctinobacteria			
ctinobacter	ctinobacter	omonospor	omonospor	ctinoplanes

Bacteroidete lavobacteri avobacteria vobacteria avobacterium
roteobacter naproteoba udomonad domonada seudomonas
ctinobacter ctinobacter ctinobacter Iicrococcal robacteriaceae
roteobacter aproteobact aproteobact rkholderia namonadaceae
roteobacter naproteoba naproteoba thomonad thomonadaceae
ctinobacter ctinobacter ctinobacter ineosporial neosporiaceae
Chloroflexi Chloroflexi hloroflexal FFCH7168
roteobacter naproteoba thomonad thomonada Arenimonas
ctinobacter ctinobacter pionibacteri cardioidac Nocardioides
roteobacter aproteobac Rhizobiales Rhizobiacea Rhizobium
ococcus-Th occi;Deino einococcace Deinococcus
roteobacter naproteoba thomonad thomonada Arenimonas
roteobacter aproteobact aproteobact ethylophila thylophilaceae
Bacteroidete lavobacteri avobacteria vobacteria avobacterium
ctinobacter ctinobacter ctinobacter ineosporial neosporiaceae
roteobacter aproteobac ingomonad ngomonada phingomonas
roteobacter aproteobact aproteobact rkholderia namonadaceae
Bacteroidete lavobacteri avobacteria vobacteria avobacterium
roteobacter naproteoba thomonad thomonada Arenimonas
roteobacter aproteobac Rhizobiales homicrobia Devosia
roteobacter aproteobac Rhizobiacea Rhizobiacea Shinella
Bacteroidete hingobacte ingobacter env.OPS 17
roteobacter aproteobac ingomonad ngomonada phingomonas
roteobacter aproteobact aproteobact rkholderia namonadaceae
roteobacter aproteobac ulobacteral ulobacterac Asticcacaulis
roteobacter aproteobac ulobacteral ulobacterac evundimonas
ctinobacter ctinobacter ctinobacter Iicrococcal asporangiaceae
roteobacter naproteoba naproteoba thomonad thomonadaceae
roteobacter aproteobact aproteobact rkholderia namonadaceae
roteobacter aproteobac aproteobac ingomonad ngomonadaceae
roteobacter aproteobac ingomonad ngomonada phingomonas
roteobacter naproteoba udomonad domonada seudomonas
ctinobacter ctinobacter ctinobacter Iicrococcal robacteriaceae
ctinobacter ctinobacteria
roteobacter aproteobac Rhizobiales homicrobia Devosia
Chloroflexi Chloroflexi hloroflexal oseiflexace Roseiflexus
roteobacter aproteobac aproteobac ingomonad ngomonadaceae
Bacteroidete lavobacteri avobacteria vobacteria avobacterium
Chloroflexi Chloroflexi hloroflexal oseiflexace Roseiflexus
roteobacter naproteoba naproteoba thomonad thomonadaceae
ctinobacter ctinobacteria
roteobacter naproteoba udomonad domonada seudomonas
ctinobacter ctinobacteria
Bacteroidete hingobacte hingobacte ingobacteri env.OPS 17
Bacteroidete lavobacteri avobacteria vobacteria avobacterium
roteobacter aproteobac aproteobac Iyxococcal ndaracinaceae
roteobacter naproteoba naproteoba thomonad thomonadaceae
ctinobacter ctinobacteria
roteobacter aproteobac Rhizobiales dyrhizobia adyrhizobium
ctinobacter ctinobacter ctinobacter Iicrococcal icrococcaceae
etes;Heloti Ascomycot eotiomycet Helotiales Helotiaceae Tetracladium
etes;Heloti Ascomycot eotiomycet Helotiales Helotiales Helotiaceae
riomycetes Ascomycot rdariomyce Hypocreale rdariomyce Hypocreales
riomycetes Ascomycot rdariomyce Hypocreale rdariomyce Hypocreales

;Helotiace	Ascomycot	eotiomycet	Helotiales	Helotiacea	etracladium
etes;Heloti	Ascomycot	eotiomycet	Helotiales	Helotiacea	etracladium
etes;Heloti	Ascomycot	eotiomycet	Helotiales	Helotiacea	etracladium
riomycetes	Ascomycot	rdariomyce	Iypocreale	rdariomyce	Iypocreales
riomycetes	Ascomycot	rdariomyce	Iypocreale	rdariomyce	Iypocreales
;Helotiace	Ascomycot	eotiomycet	Helotiales	Helotiacea	etracladium
;Helotiace	Ascomycot	eotiomycet	Helotiales	Helotiacea	etracladium
etes;Pleos	Ascomycot	thideomyce	leosporale	leosporace	Alternaria
gi;Ascomy	Ascomycota				
etes;Heloti	Ascomycot	eotiomycet	Helotiales	Helotiacea	etracladium
ectosphaere	Ascomycot	rdariomyce	lomerellal	osphaerell	ectosphaerella
etes;Pleos	Ascomycot	thideomyce	leosporale	leosporace	Alternaria
omycetes;I	Ascomycot	thideomyce	leosporale	thideomyce	leosporales
omycetes;I	Ascomycot	thideomyce	leosporale	thideomyce	leosporales
gi;Ascomy	Ascomycota				
etes;Pleos	Ascomycot	thideomyce	leosporale	leosporale	leosporaceae
;Phaeospha	Ascomycot	thideomyce	leosporale	eosphaeria	Paraphoma
;Helotiace	Ascomycot	eotiomycet	Helotiales	Helotiacea	etracladium
etes;Pleos	Ascomycot	thideomyce	leosporale	leosporace	Alternaria
;Phaeospha	Ascomycot	thideomyce	leosporale	eosphaeria	Paraphoma
etes;Pleos	Ascomycot	thideomyce	leosporale	leosporale	leosporaceae
riomycetes	Ascomycot	rdariomyce	Iypocreale	rdariomyce	Iypocreales
es;Capnodi	Ascomycot	thideomyce	Capnodiale	Capnodiale	adosporiaceae
ectosphaere	Ascomycot	rdariomyce	lomerellal	osphaerell	ectosphaerella
;Helotiace	Ascomycot	eotiomycet	Helotiales	Helotiacea	etracladium
ectosphaere	Ascomycot	rdariomyce	lomerellal	osphaerell	ectosphaerella
gi;Ascomy	Ascomycota				
gi;Ascomy	Ascomycota				
es;Helotiac	Ascomycot	eotiomycet	Helotiales	Helotiacea	rticulospora
etes;Heloti	Ascomycot	eotiomycet	Helotiales	Helotiacea	rticulospora
omycetes;P	Ascomycot	thideomyce	leosporale	idymellaceae	
es;Capnodi	Ascomycot	thideomyce	Capnodiale	Capnodiale	adosporiaceae
etes;Heloti	Ascomycot	eotiomycet	Helotiales	Helotiacea	rticulospora
nycetes;Ca	Ascomycot	thideomyce	Capnodiale	thideomyce	Capnodiales
es;Capnodi	Ascomycot	thideomyce	Capnodiale	Capnodiale	adosporiaceae
Dothideom	Ascomycot	thideomyce	leosporale	Ascomycot	thideomycetes
riomycetes	Ascomycot	rdariomyce	Iypocreale	rdariomyce	Iypocreales
nycota;Dotl	Ascomycot	thideomycetes			Ascomycota
gi;Ascomy	Ascomycota				
gi;Ascomy	Ascomycota				
porales;Did	Ascomycot	thideomyce	leosporale	idymellace	Epicoccum
es;Helotiac	Ascomycot	eotiomycet	Helotiales	Helotiacea	rticulospora
ortierellales	rtierellomy	tierellomyc	Iortierellal	ortierellace	Mortierella
gi;Ascomy	Ascomycota				
nycetes;Ple	Ascomycot	thideomyce	leosporale	thideomyce	leosporales
Dothideom	Ascomycot	thideomyce	leosporale	Ascomycot	thideomycetes
;Pleosporal	Ascomycot	thideomyce	leosporale	eosphaeria	haeosphaeria
gi;Ascomy	Ascomycota				
Dothideom	Ascomycot	thideomyce	leosporale	Ascomycot	thideomycetes
gi;Ascomy	Ascomycota				
les;Plectos	Ascomycot	rdariomyce	lomerellal	osphaerella	Lectera
Dothideom	Ascomycot	thideomyce	leosporale	Ascomycot	thideomycetes
es;Capnodi	Ascomycot	thideomyce	Capnodiale	Capnodiale	adosporiaceae
porales;Did	Ascomycot	thideomyce	leosporale	idymellace	Epicoccum

ycetes;Hyp	Ascomycot	rdariomyce	Hypocreale	Hypocreale	Nectriaceae
ycetes;Hyp	Ascomycot	rdariomyce	Hypocreale	Nectriaceae	Fusarium
les;Melanc	Ascomycot	thideomyce	Pleosporale	anommata	Herpotrichia
;Phaeospha	Ascomycot	thideomyce	Pleosporale	eosphaeria	Paraphoma
riomycetes	Ascomycot	rdariomyce	Hypocreale	rdariomyce	Hypocreales
comycetes;	asidiomyco	aricomyce	uriculariale	Exidiaceae	
etes;Heloti	Ascomycot	eotiomycet	Helotiales	Helotiacea	rticulospora
ycetes;Hyp	Ascomycot	rdariomyce	Hypocreale	Hypocreale	Nectriaceae
ycetes;Cap	Ascomycot	thideomyce	Capnodiale	osphaerellaceae	
Dothideom	Ascomycot	thideomyce	Pleosporale	Ascomycot	thideomycetes
es;Capnodi	Ascomycot	thideomyce	Capnodiale	Capnodiale	adosporiaceae
;Pleosporal	Ascomycot	thideomyce	Pleosporale	eosphaeria	haeosphaeria
ortierellale	rtierellomy	tierellomyc	Mortierellal	ortierellace	Mortierella
etes;Heloti	Ascomycot	eotiomycet	Helotiales	Helotiacea	rticulospora
les;Plectos	Ascomycot	rdariomyce	lomerellale	osphaerella	Lectera
;Phaeospha	Ascomycot	thideomyce	Pleosporale	eosphaeria	Paraphoma
es;Helotiac	Ascomycot	eotiomycet	Helotiales	Helotiacea	rticulospora
ortierellale	rtierellomy	tierellomyc	Mortierellal	ortierellace	Mortierella
etes;Heloti	Ascomycot	eotiomycet	Helotiales	Helotiacea	rticulospora
;Pleosporal	Ascomycot	thideomyce	Pleosporale	eosphaeria	haeosphaeria
es;Capnodi	Ascomycot	thideomyce	Capnodiale	Capnodiale	adosporiaceae
etes;Pleosp	Ascomycot	thideomyce	Pleosporale	leosporacea	Alternaria
;Helotiacea	Ascomycot	eotiomycet	Helotiales	Helotiaceae	Tetracladium
ycetes;Ple	Ascomycot	thideomyce	Pleosporale	eosphaeriaceae	
;Phaeospha	Ascomycot	thideomyce	Pleosporale	eosphaeria	Paraphoma
s;Bulleriba	asidiomyco	emellomyce	Tremellales	leribasidia	ishniacozyma
tes;Xylaria	Ascomycot	rdariomyce	Xylariales	Diatrypacea	onosporascus
;Phaeospha	Ascomycot	thideomyce	Pleosporale	eosphaeria	Paraphoma
;Helotiacea	Ascomycot	eotiomycet	Helotiales	Helotiaceae	Tetracladium
Dothideom	Ascomycot	thideomyce	Pleosporale	Ascomycot	thideomycetes
ortierellales	rtierellomy	tierellomyc	Mortierellal	ortierellace	Mortierella
;Pleosporal	Ascomycot	thideomyce	Pleosporale	tosphaeria	eptosphaeria
etes;Heloti	Ascomycot	eotiomycet	Helotiales	Helotiales	Helotiaceae
es;Helotiac	Ascomycot	eotiomycet	Helotiales	Helotiacea	rticulospora
etes;Heloti	Ascomycot	eotiomycet	Helotiales	Helotiaceae	Tetracladium
rtierellales;	rtierellomy	tierellomyc	Mortierellal	ortierellace	Mortierella
es;Capnodi	Ascomycot	thideomyce	Capnodiale	Capnodiale	adosporiaceae
s;Bulleriba	asidiomyco	emellomyce	Tremellales	leribasidia	ishniacozyma
s;Pleospora	Ascomycot	thideomyce	Pleosporale	Pleosporale	idymellaceae
s;Pleospora	Ascomycot	thideomyce	Pleosporale	Pleosporale	idymellaceae
ortierellale	rtierellomy	tierellomyc	Mortierellal	ortierellace	Mortierella
les;Plectos	Ascomycot	rdariomyce	lomerellale	osphaerella	Lectera

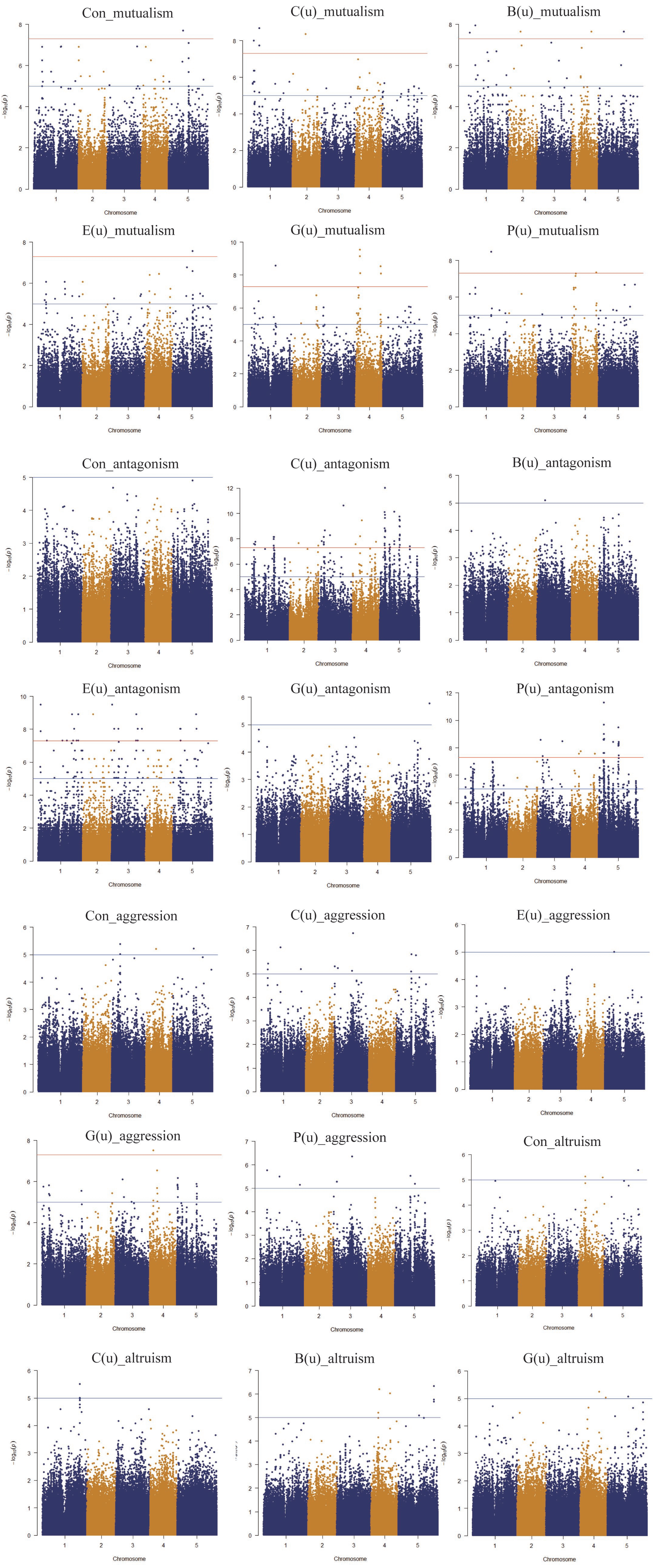
Con_mutualism
C(u)_mutualism
B(u)_mutualism
E(u)_mutualism
G(u)_mutualism
P(u)_mutualism
Con_antagonism
C(u)_antagonism
B(u)_antagonism
E(u)_antagonism
G(u)_antagonism
P(u)_antagonism
Con_aggression
C(u)_aggression
E(u)_aggression
G(u)_aggression
P(u)_aggression
Con_altruism
C(u)_altruism
B(u)_altruism
G(u)_altruism
Chromosome
$-\log_{10}(p)$

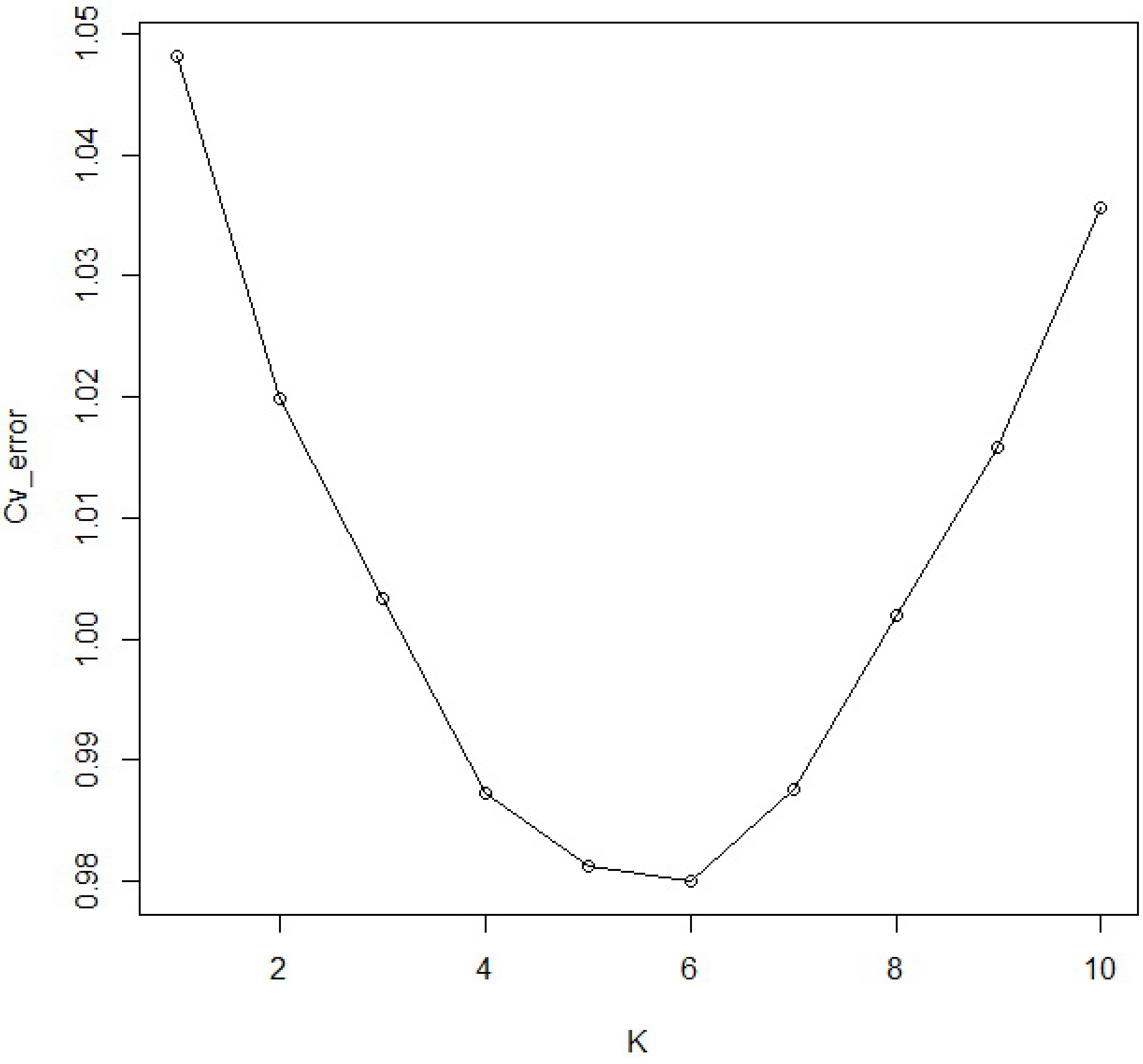

1.05
1.04
1.03
1.02
1.01
1.00
0.99
0.98
Cv_error
2
4
6
8
10
K

Structure

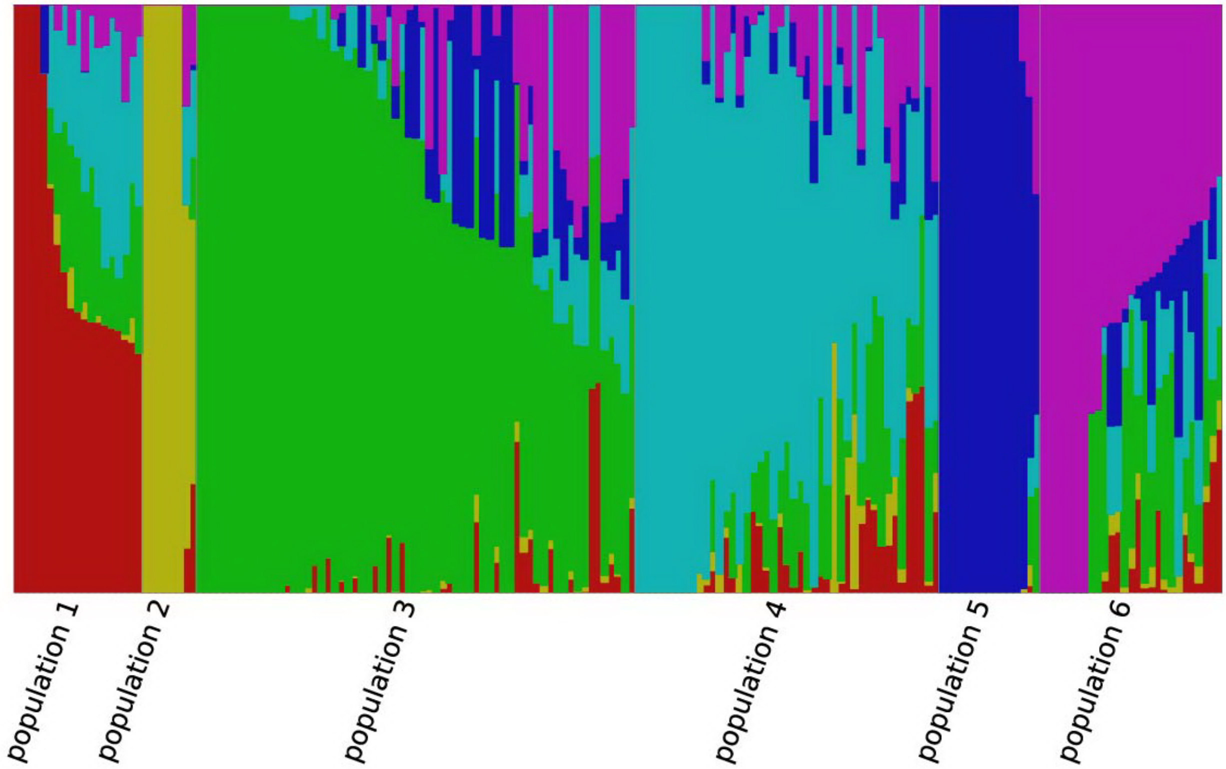
population 1
population 2
population 3
population 4
population 5
population 6

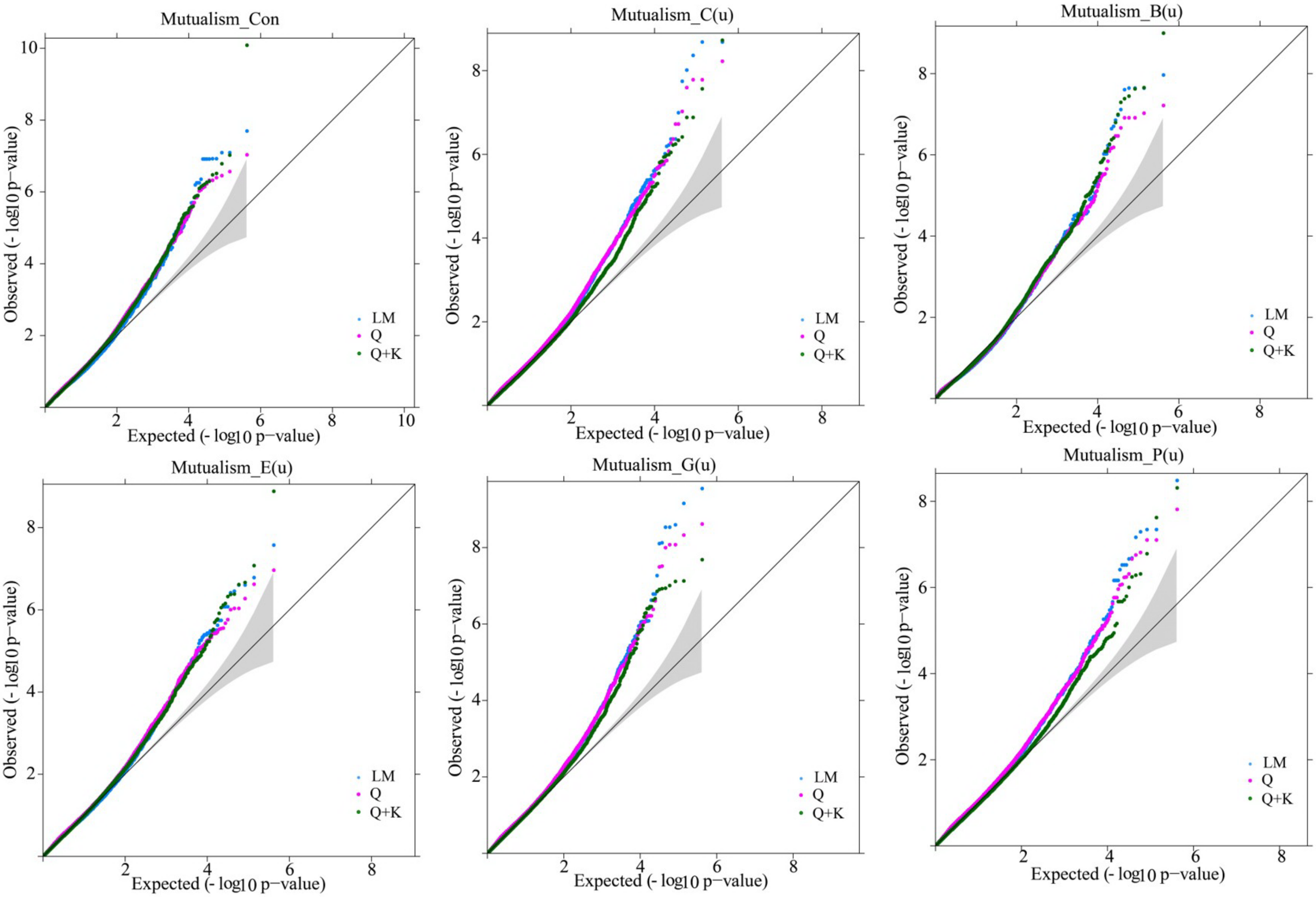

Mutualism_Con
Mutualism_C(u)
Mutualism_B(u)
Mutualism_E(u)
Mutualism_G(u)
Mutualism_P(u)
Observed (- log10 p−value)
Expected (- log10 p−value)
LM
Q
Q+K

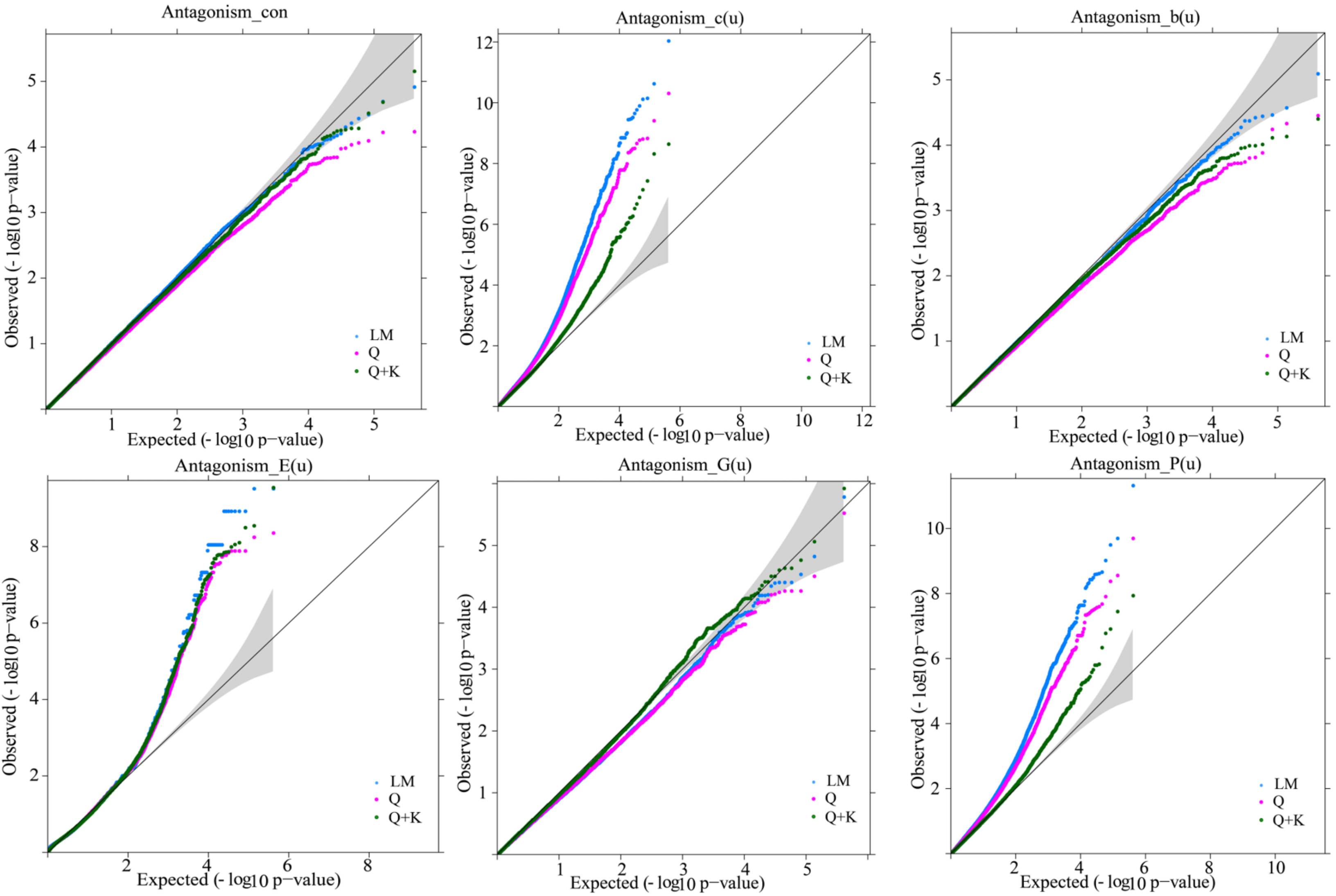
Antagonism_con
Antagonism_c(u)
Antagonism_b(u)
Antagonism_E(u)
Antagonism_G(u)
Antagonism_P(u)
Observed (- log10 p−value)
Expected (- log10 p−value)
LM
Q
Q+K

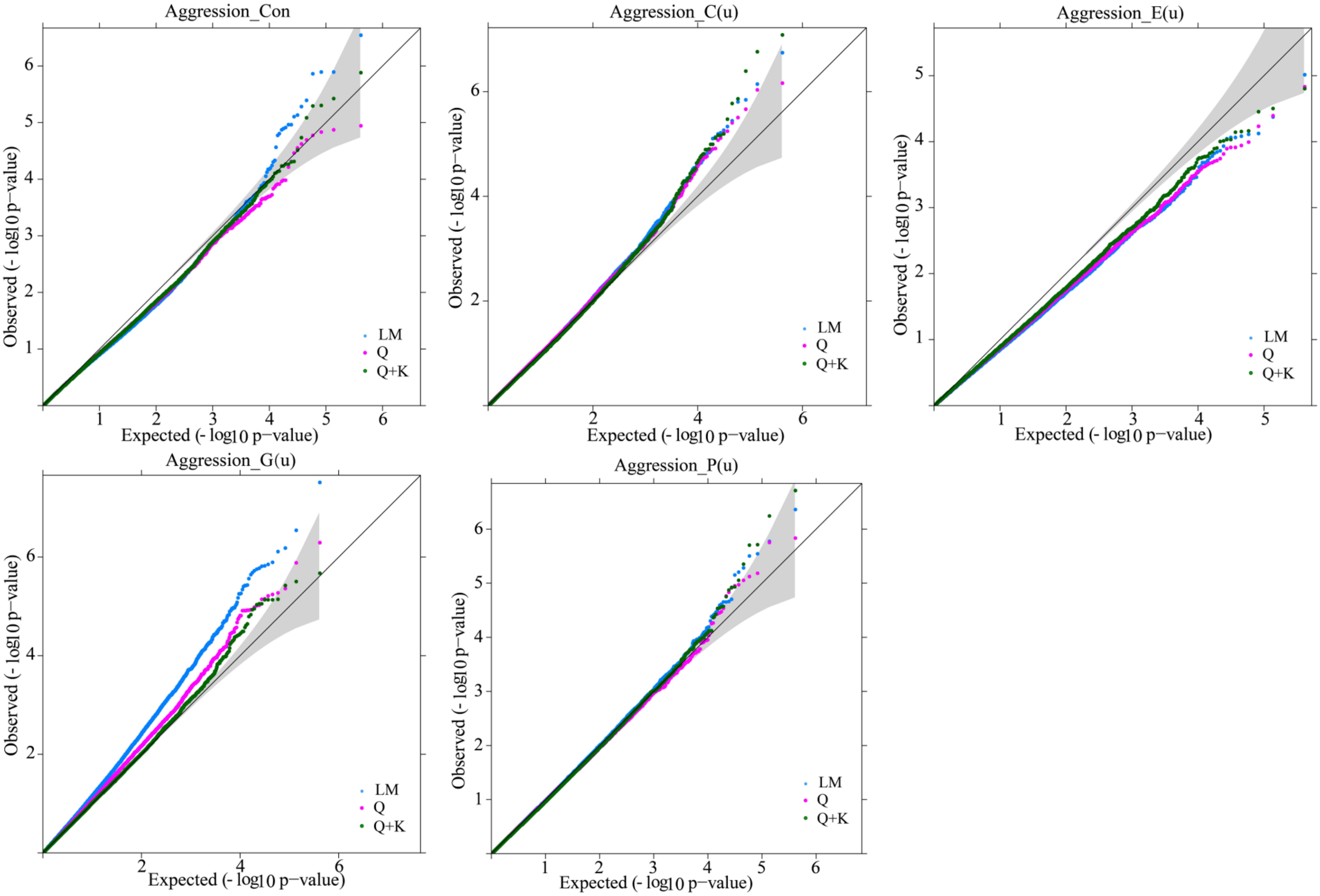
Aggression_Con
Aggression_C(u)
Aggression_E(u)
Aggression_G(u)
Aggression_P(u)
Observed (- log10 p−value)
Expected (- log10 p−value)
LM
Q
Q+K

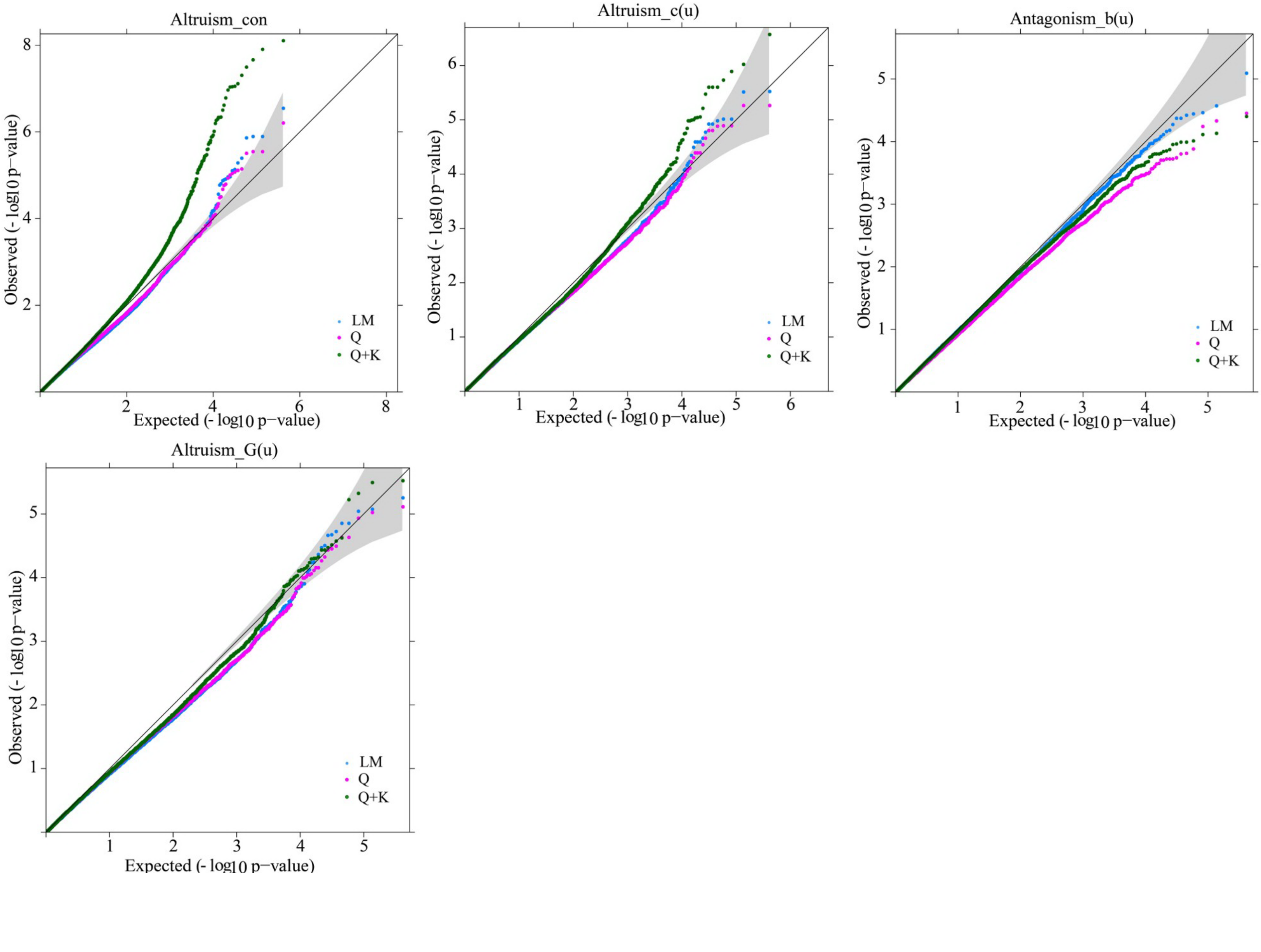
Altruism_con
Observed (- log10 p−value)
Expected (- log10 p−value)
LM
Q
Q+K
Altruism_c(u)
Observed (- log10 p−value)
Expected (- log10 p−value)
LM
Q
Q+K
Antagonism_b(u)
Observed (- log10 p−value)
Expected (- log10 p−value)
LM
Q
Q+K
Altruism_G(u)
Observed (- log10 p−value)
Expected (- log10 p−value)
LM
Q
Q+K
